# Non-targeted transcription factors motifs are a systemic component of ChIP-seq datasets

**DOI:** 10.1186/s13059-014-0412-4

**Published:** 2014-07-29

**Authors:** Rebecca Worsley Hunt, Wyeth W Wasserman

**Affiliations:** Centre for Molecular Medicine and Therapeutics, Child and Family Research Institute, University of British Columbia, Vancouver, BC Canada; Bioinformatics Graduate Program, University of British Columbia, Vancouver, BC Canada; Department of Medical Genetics, University of British Columbia, Vancouver, BC Canada

## Abstract

**Background:**

The global effort to annotate the non-coding portion of the human genome relies heavily on chromatin immunoprecipitation data generated with high-throughput DNA sequencing (ChIP-seq). ChIP-seq is generally successful in detailing the segments of the genome bound by the immunoprecipitated transcription factor (TF), however almost all datasets contain genomic regions devoid of the canonical motif for the TF. It remains to be determined if these regions are related to the immunoprecipitated TF or whether, despite the use of controls, there is a portion of peaks that can be attributed to other causes.

**Results:**

Analyses across hundreds of ChIP-seq datasets generated for sequence-specific DNA binding TFs reveal a small set of TF binding profiles for which predicted TF binding site motifs are repeatedly observed to be significantly enriched. Grouping related binding profiles, the set includes: CTCF-like, ETS-like, JUN-like, and THAP11 profiles. These frequently enriched profiles are termed ‘zingers’ to highlight their unanticipated enrichment in datasets for which they were not the targeted TF, and their potential impact on the interpretation and analysis of TF ChIP-seq data. Peaks with zinger motifs and lacking the ChIPped TF’s motif are observed to compose up to 45% of a ChIP-seq dataset. There is substantial overlap of zinger motif containing regions between diverse TF datasets, suggesting a mechanism that is not TF-specific for the recovery of these regions.

**Conclusions:**

Based on the zinger regions proximity to cohesin-bound segments, a loading station model is proposed. Further study of zingers will advance understanding of gene regulation.

**Electronic supplementary material:**

The online version of this article (doi:10.1186/s13059-014-0412-4) contains supplementary material, which is available to authorized users.

## Background

The mapping of the regulatory sequences in the human genome is proceeding rapidly. Large-scale chromatin immunoprecipitation coupled to high-throughput sequencing (ChIP-seq) experiments have been a central component of the mapping efforts, including both transcription factor (TF) target and histone target derivatives [1]. These mapping efforts are providing key insights into the properties of regulatory sequences, the interactions between TFs, and the mechanisms contributing to selective patterns of gene transcription. With the compilation of large and diverse ChIP-seq data collections, an opportunity has emerged to study the common characteristics of TF-bound regions revealed by ChIP-seq.

The characteristics of ChIP-seq data are shaped by both biological and technical influences [2–5]. As with every high-throughput technology, the community learns progressively more about the nuances of the data as they accumulate. Much effort has focused on the development of peak finding methods, which allow for the quantitative determination of TF-bound regions within the sequences recovered in a ChIP-seq experiment. In general, most methods take into account a background rate of sequence recovery and use this background to evaluate the significance of an observed number of mapped reads in the foreground ChIP experiment [2]. Most commonly background sequence data sources are generated from sheared input DNA or mock immunoprecipitation (mock-IP) using a non-specific antibody (for example, IgG). The comparison of the foreground against the background by peak finding software is often the basis for specifying the TF-bound regions, usually delineated with a start, stop, and local maximum read density position (that is, ‘peakMax’).

It is clear that the ChIP-seq procedure is working well for detecting regions bound by sequence-specific TFs. Analysis of ChIP-seq datasets reveals an enrichment of the expected TF binding site (TFBS) pattern close to the peakMax or, where no peakMax is determined, peak centre positions (hereafter also referred to as ‘peakMax’) [6,7]. *Ab initio* pattern discovery software applied to ChIP-seq data routinely recover the known TFBS pattern [8], and pattern enrichment methods confirm highly significant enrichment of the TFBS pattern of the ChIPped TF [9,10]. Additionally, a sufficient number of replicates have been performed to demonstrate general consistency between ChIP-seq datasets using the same cells and antibodies [11].

The properties of DNA in the nucleus have a strong influence on the results of diverse methods, including ChIP-seq and DNase I hypersensitivity mapping data [12]. Both input DNA and diverse ChIPped DNA reveal a strong tendency for the recovery of sequences from promoter regions [4,11], indicating that the DNA shearing process favors regions of open or less compact DNA. These open regions have been demonstrated to be enriched for TF binding and other indicators of accessible DNA such as key histone modifications [13].

One of the open questions about ChIP-seq results is the not infrequent recovery of peaks under which the target motif of the ChIPped TF is absent. Such observations might be attributable to an inadequate understanding of the TF binding specificity, the potential indirect tethering of a TF to a region through protein-protein interactions, or non-specific antibody pull-down. Based on this background, we sought to understand the properties of ChIP-seq TF binding data, with an emphasis on the identification of mechanisms to account for the past observations of peaks lacking the motif of the targeted TF. Based on our research, we report a striking property of TFBS enrichment around the peakMax for CTCF-like, JUN-like, ETS-like, and THAP11 motifs across a broad set of TF ChIP-seq data. The broadly enriched TFBS classes, which we term ‘zingers’ for their startling enrichment, can account for a substantial portion of TFBS ChIP-seq data. The zinger regions are observed to recur across ChIP-seq data from multiple cell lines and for multiple TFs. These recurring regions tend to be proximal to structural features defined by cohesin and polycomb group proteins. A model to account for the observed properties of zingers is introduced and discussed.

## Results

### Zingers are TF binding motifs enriched across multiple TF ChIP-seq datasets

A subset of TF ChIP-seq data has been reported to lack motifs for the ChIPped TF, suggesting that there may be additional proteins interacting in a sequence specific manner with these regions. Drawing together diverse TF-ChIP-seq data, we sought to determine if characterized TFs might account for a portion of the discrepancy. To measure the enrichment of TF motifs across the compiled TF ChIP-seq datasets we performed motif over-representation analyses, using the oPOSSUM 3.0 software [9]. We tested 165 position weight matrices (PWMs) selectively curated from the JASPAR development database (see methods), on 285 human datasets (33 cell-lines) for 101 TFs (ENCODE and other resources; see Materials and methods). A parallel analysis of mouse data was performed for 81 datasets (12 cell-lines) encompassing 43 TFs (ENCODE and other resources; see Materials and methods). For each oPOSSUM analysis we provided a set of background sequences of similar length and nucleotide composition relative to the ChIP-seq dataset (all peaks were constrained to 401 bp length). As there were two or more ChIP-seq datasets for many TFs, generated from different cell lines or conditions, we averaged the oPOSSUM enrichment scores across all datasets for a given ChIPped TF. The details of the statistical measures and assessed thresholds are presented in the methods. Briefly, two oPOSSUM enrichment scores were used to evaluate the datasets: a Fisher-log score (to assess enrichment of motifs across many ChIP-seq peaks) and a Kolmogorov-Smirnov (KS) centrality score (to assess enrichment of motifs in proximity to the peakMax position).

**Figure 1:**
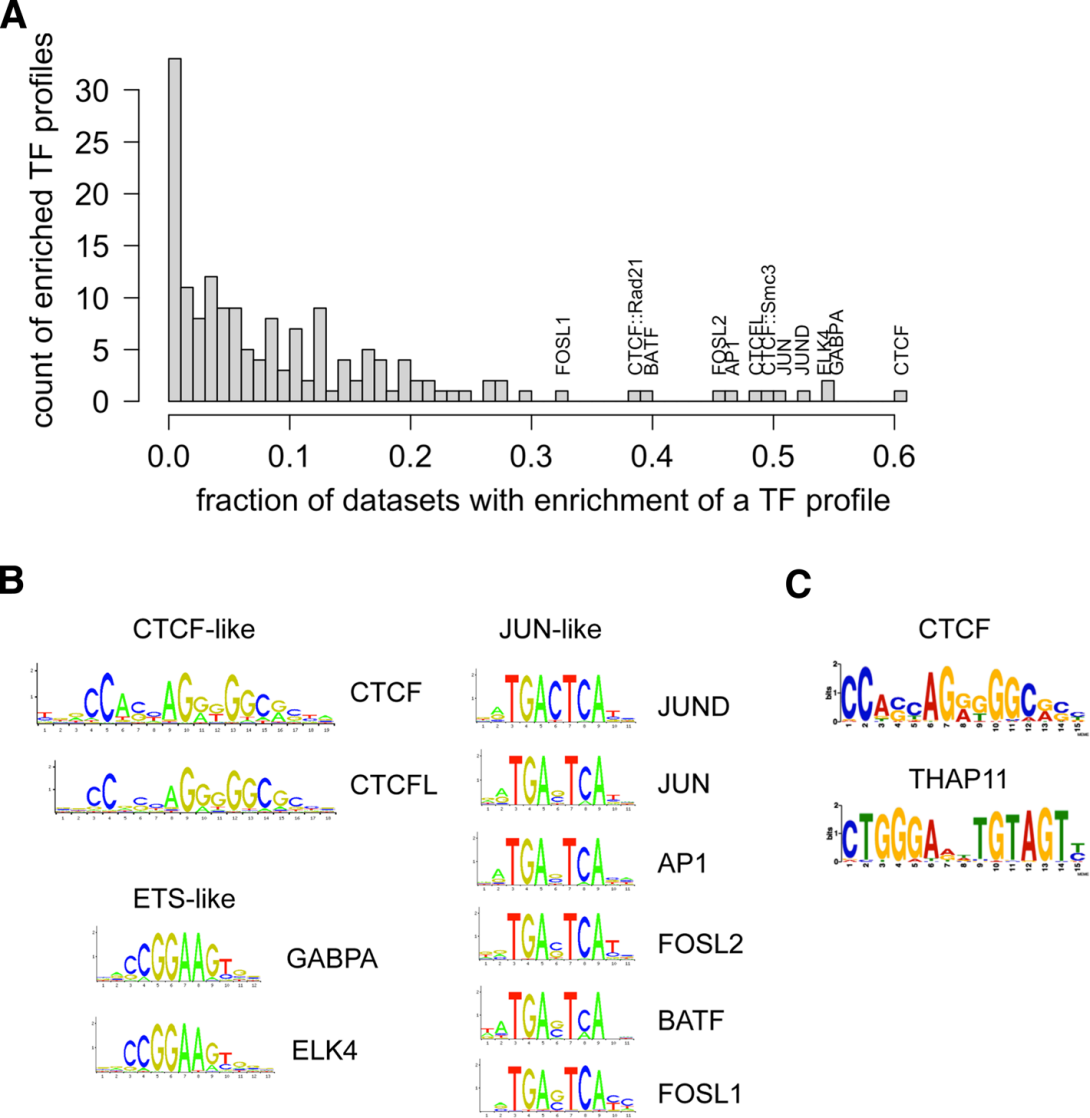
**Zinger binding motifs are enriched across multiple human ChIP-seq datasets. (A)** The histogram displays the results of TFBS motif enrichment analysis on 281 human ChIP-seq datasets generated with the oPOSSUM 3.0 software. Along the x-axis is the fraction of datasets that displayed enrichment near the peakMax for a TF profile. The y-axis is the number of TF profiles that were found enriched for a given fraction of datasets. The profiles most frequently observed to be enriched are labeled on the histogram. The likelihood (*P* values) of a PWM with the same width, information content, and GC composition as the CTCF, GABPA, or JUN PWMs to attain the enrichment frequency observed in the histogram follow: 2.5e-44 for CTCF, 2.8e-09 for GABPA, and 3.7e-08 for JUN. **(B)** The binding site logos of the 10 TF binding models with enriched motifs across the greatest number of datasets, manually grouped by motif similarity. Each logo depicts position along the x-axis and information content (that is, pattern strength) along the y-axis. **(C)** Motifs detected consistently by *ab initio* motif discovery across five datasets of 5,000 random sequences. The upper motif is similar to the CTCF logo in section B, while the lower motif is similar to the motif for the THAP11 TF.

Of 165 TF motifs analyzed, CTCF, ETS-like (for example, GABPA and ELK4), and JUN-like motifs were found to be both the most enriched and most proximal to the peakMax across the greatest number of both human (Figure 1A and binding site logos in 1B) and mouse (Additional file 1: Figure S1A and binding site logos in S1B) TFs’ datasets. We refer to such broadly enriched TF motifs as ‘zingers’, reflecting their potential to confound the analysis and interpretation of TF ChIP-seq results.

To assess if zinger enrichment is independent of the ChIPped TFs’ motifs (that is, not over-lapping the expected motif), we performed a second enrichment analysis on human ChIP-seq sequences in which the ChIPped TF motifs were masked (thus restricting the analysis to the subset of ChIP-seq datasets for which a TF binding profile is available). We again consider the two metrics of Fisher-log enrichment score and KS centrality score. The CTCF, ETS-like, and JUN-like zinger motifs remained enriched (Additional file 2: Figure S2A).

Short patterns, such as those found by PWMs, can occur by chance in the genome. To confirm the findings of zinger-specific enrichment, we shuffled the zinger PFMs and determined the likelihood of achieving the frequency of enriched datasets observed for the original profile (see Materials and methods). In all cases, the comparison against the frequency of enriched datasets obtained with the shuffled matrices confirmed that the true zinger motifs’ enrichment was extremely unlikely to occur by chance (*P* values are: 2.5e-44 for CTCF, 2.8e-09 for GABPA, and 3.7e-08 for JUN).

### *Ab initio* motif discovery of zinger profiles

We sought to determine if *ab initio* pattern discovery could recover either novel profiles or known TFBS profiles in pooled data, a process requiring a greater signal-to-noise ratio than the more noise-tolerant oPOSSUM motif enrichment testing above. Across all of the ChIP-seq data, we masked the motif of the ChIPped TF and repeat-masked the sequences (see Materials and methods), then drew five sets of 5,000 sequences from the ChIP-seq pool and subjected each set to pattern discovery analysis using the MEME system [8]. From the five replicate pools, MEME returned profiles for wide and high information content patterns. In all cases MEME detected a pattern consistent with the CTCF binding profile in the top six results (Figure 1C, top logo) and a profile unknown to MEME Suite’s TOMTOM pattern similarity scoring system [14] (Figure 1C, bottom logo). A report from Ngondo-Mbongo *et al.* [15] identified that THAP11 binds to a motif that matches the unknown profile, so we will hereafter refer to the MEME derived profile as the THAP11 profile. We reviewed oPOSSUM results for the enrichment of the THAP11 motif, and found that it is consistent with the zingers for the Fisher-log score enrichment frequency, but the motif is not frequently observed to be centrally positioned based on the oPOSSUM KS-score (although it is proximal to the peakMax by the heuristic motif enrichment method presented below in this report). Given the strength of evidence, we elected to classify THAP11 motif as an additional zinger.

### Zinger motif enrichment observed within open chromatin and genomic datasets

Using the oPOSSUM enrichment analysis procedure, we sought to determine if the zingers showed enrichment in other genomic data collections. ChIP-seq data are recognized to be highly enriched with open chromatin regions, and in particular ChIP-seq data for CTCF, one of the zinger TFs, are known to strongly overlap with DNase I hypersensitive sites [16,17]. We therefore analyzed ENCODE DNaseI-seq and Faire-seq data to assess the enrichment of the zinger motifs. Each region (average 150 bp) was extended to 401 bp for enrichment analysis using the oPOSSUM 3.0 software. oPOSSUM enrichment results revealed the zinger profiles to be the most frequently enriched within DNaseI-seq and Faire-seq datasets, showing enrichment near the region centre in 50% to 100% of the DNaseI-seq datasets, and 20% to 92% of Faire-seq datasets (Additional file 3: Figure S3). We further assessed the ratio of zinger motifs in DNase and Faire regions compared to flanking regions, providing an indication of the portion of each dataset that could be attributed to zingers: mean values of 47% for DNaseI-seq and 13% for Faire-seq were obtained (see Additional file 4: Text S1).

We have observed enrichment of zingers in other open chromatin associated data such as ChIP-seq data for helicase-related proteins or histone modifiers (Additional file 4: Text S1 and Additional file 5: Figure S4), and ChIP-seq control data (Additional file 4: Text S1 and Additional file 6: Figure S5). Thus zinger motifs are observed in multiple classes of genomic datasets.

### Visualizing the pattern of motif enrichment

**Figure 2:**
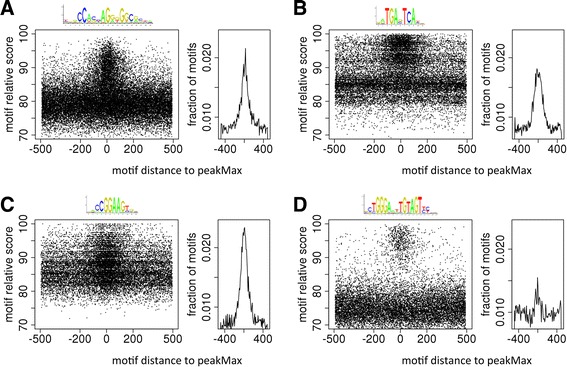
**Zinger motifs are enriched at the peak maximum of non-zinger ChIP-seq datasets.** The enrichment plots display the location of the top scoring motif for each peak relative to the peakMax (the peakMax is at 0) on the x-axis, while the score of the motif is plotted on the y-axis. The adjacent line plots display the fraction of motifs observed in 5 bp increments. The logo reflecting the binding specificity for each zinger appears above the related enrichment plot. **(A)** CTCF motif predictions from NRF1 ChIP-seq (GM12878 cells). **(B)** JUN motif predictions from TCF7L2 ChIP-seq (Hct116 cells). **(C)** GABPA motif predictions from NFKB ChIP-seq (GM19099 cells). **(D)** THAP11 motif predictions from IRF1 ChIP-seq (K562 cells).

We first used visualization approaches to examine the distribution of both the motif scores and peakMax proximity for the CTCF, JUN, GABPA, and THAP11 zinger motifs for several datasets using TFBS-landscape plots [18]. To visually assess the topological pattern of enrichment of zinger motifs using TFBS-landscape plots, we extended all analyzed sequences to 1,001 bp (peakMax position at 501 bp), and plotted the motif position relative to the peakMax (x-axis; upstream and downstream of peakMax) and the motif score (y-axis) of the top scoring zinger motif for each peak. As seen in Figure 2, the motif predictions of zinger PWMs are in general concentrated in motif score ranges across all positions relative to the peakMax, for example, motif scores 70 to 85 for CTCF (Figure 2A), or 80 to 87 for JUN (Figure 2B). However, proximal to the peakMax, there is a distinctive enrichment for the zinger motif, most strikingly seen for CTCF and THAP11 where almost all high scoring motifs (>85) are located proximal to the peakMax. The enrichment of JUN and particularly GABPA zinger motifs are less distinctive visually, due to the peakMax proximal enrichment overlapping the same score range as the background motifs. In control datasets and with shuffled matrices we do not see the distinct high scoring population of motif scores; we instead see a uniform distribution along the total 1,001 bp of sequence, which conveys, visually, the background rate of motif prediction for the PWM (Additional file 7: Figure S6). The distinctive zinger motif enrichment allowed for the selection of subsets of peaks that were enriched for the motif of a TF that was not specifically targeted by the ChIP-seq experiment.

### Defining a set of zinger motif containing peaks

**Figure 3:**
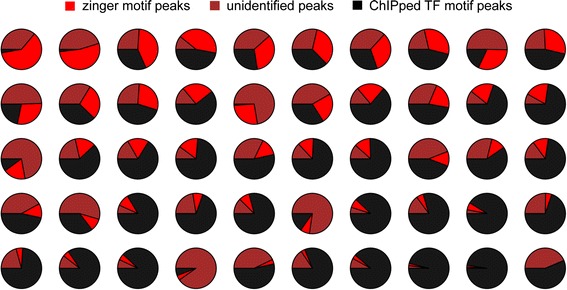
**The fraction of zinger motif peaks and ChIPped TF motif peaks varies across ChIP-seq datasets.** The pie charts present a random selection of 50 datasets for multiple TFs and cell-lines with zinger motifs present (>1% zinger). The charts are ordered by greatest zinger motif peak enrichment to the least. Black is the portion of peaks with the ChIPped TF’s motif, red is the portion of zinger motif peaks, and brown is the remaining portion of peaks that do not contain either the ChIPped TF nor zinger motifs.

Based on the visualization analysis we used a procedure for determining the range of motif enrichment relative to peakMax proximity and motif score enrichment [18]. The outer limits of these ranges of enrichment were then applied as thresholds that defined ‘enrichment zones’ for quantitative analysis of ChIP-seq dataset motif composition (Figure 3; see Materials and methods).

For ease of reference, we will hereafter use ‘zinger motifs’ to refer to the collection of CTCF, JUN-like, ETS-like, and THAP11 motifs within the enrichment zones and ‘zinger motif peaks’ to refer to those peaks within a dataset that have a zinger motif but not the ChIPped TF’s motif. Motif predictions outside the enrichment zones will be referred to as ‘distal-zinger’ motifs.

As anticipated, peaks with the ChIPped TF’s motif proximal to the peakMax comprised the majority of most datasets (up to 99% in the best case). After accounting for background ChIPped TF motif rates, the mean observed portion was 55% (the median was 59% with a median absolute deviation (MAD) of 27 pp). There are, however, extreme cases in which the ChIPped TF’s canonical binding motif is present in less than 10% of the peaks (Additional file 4: Text S1 and Additional file 8: Figure S7).

After accounting for background, and excluding two outliers, up to 45% of a ChIP-seq dataset are zinger motif peaks with a mean of 12% (median of 9% with a MAD of 3 pp) (Additional file 9: Figure S8A). The zinger motif peaks account for up to 69% of the set of peaks unexplained by the ChIPped TF’s motif, with a mean of 27% (median of 27% with MAD of 14 pp), in datasets with at least 1% zinger motif peak content (Additional file 9: Figure S8B); the zinger motif peak enrichment is visually depicted in a heat map format (Additional file 9: Figure S8C). For clarity, the portion of zinger motif peaks are anti-correlated with the portion of ChIPped TF motif peaks (Additional file 9: Figure S8D).

### No strong dependencies detected for zinger motif occurrence

As zinger motifs are present in peaks without the ChIPped TF’s motif we wanted to determine if there were any characteristics specific to or in common among this set of peaks. We found that neither the presence nor proportion of zinger motif peaks within a ChIP-seq dataset is dependent on cell type, as seen in Additional file 10: Figure S9A for the five most abundant cell lines. Neither, is the proportion of zinger motif-containing peaks consistent across multiple datasets for the same TF (Additional file 10: Figure S9B).

Next we asked if the zinger motifs have a strong tendency to co-occur in the same zinger motif peaks. We found that at most 11% of datasets show a positive association with a significant *P* value (Fisher exact *P* values <0.001 and log odds ratios >0) for any pairwise co-occurrence of two different zingers within a single peak (the most frequent pair of zingers being GABPA and THAP11). A few datasets (17%) show a negative association for zinger motif co-occurrence with a significant *P* value (Additional file 11: Figure S10A). Thus, the zinger motifs are not inter-dependent. We next evaluated the pairwise tendency for zinger motif peak enrichment within the same ChIP-seq datasets, finding unremarkable correlation values (correlation coefficients -0.0233 to 0.3803) (Additional file 11: Figure S10B).

Lastly we determined whether zinger motif peaks were consistently located near a feature in the genome. We evaluated the proximity of zinger-associated regions to genomic features such as transcription start sites (TSS), CpG islands, conserved regions, and repeat sequence regions. Comparing the set of zinger motif peaks to peaks with the ChIPped TF’s motif, we did not detect consistent enrichment tendencies that distinguished between the two sets of regions (Additional file 4: Text S1).

### Peaks containing a zinger motif but lacking the ChIPped TF motif have low scores

As zinger motifs are an unexpected presence across datasets we assessed the quality of the peaks they occur in, asking if the zinger motifs tended to be in the lower scoring peaks of the dataset. We compared the peak calling scores of peaks containing the ChIPped TF’s motif against peaks with a zingers’ motif. The peak scores for the zinger containing peaks are significantly poorer than for those peaks with the ChIPped TF’s canonical motif (Wilcoxon one-tailed test *P* values <5.0e-05).

### Peaks with a zinger motif may be *bona fide* targets of the zinger TF

**Figure 4:**
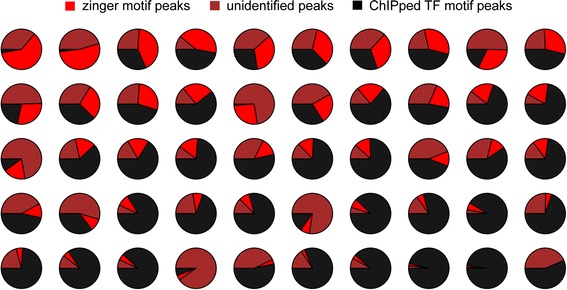
**ChIP-seq data for zinger TFs overlaps zinger motif peaks from other TF’s datasets.** For each plot, a selection of TF ChIP-seq datasets is alphabetically ordered by TF name horizontally. The y-axis represents the fraction of peaks that overlap with the zinger TF’s ChIP-seq peak in experiments performed with the same cell type. Two populations of peaks are plotted per dataset: solid circles represent the subset of peaks with a peakMax-proximal zinger motif, and open triangles represent the subset of peaks with a distal-zinger motif. **(A)** CTCF, **(B)** JUN, or **(C)** GABPA. The horizontal dashed line at 0.13 is a qualitatively selected visual aide.

Prediction of TFBSs can suffer from poor specificity, and as the enriched zinger motifs’ peaks were unexpectedly found in datasets for non-zinger TFs, we asked if the zinger motif peaks were actual binding locations for the zinger TF or not. Therefore we investigated the degree of agreement (co-occurrence within 100 bp) between zinger motif peaks with a strong motif score (score >85) and ChIP-seq data ChIPped for the zingers TF in the same cell type (Figure 4). On average 75% of zinger CTCF motif peaks overlapped CTCF ChIP-seq peaks (median 79% with a MAD of 15 pp); 38% of zinger JUN motif peaks overlapped JUN ChIP-seq peaks (median 38% with a MAD of 17 pp); and 28% of zinger GABPA motif peaks overlapped GABPA ChIP-seq peaks (median 27% with a MAD of 13 pp). In all cases the agreement was significant (Wilcoxon *P* values <3.4e-20) with respect to the distal-zinger control (see Additional file 4: Text S1), and indicated that many of the zinger regions may be *bona fide* binding regions for the zinger TF.

A comparison of the peak scores for the ChIP-seq peaks that overlapped the set of zinger motif peaks *versus* the set of distal-zinger peaks revealed a significant difference between the two groups (Wilcoxon one-tailed test significance threshold *P* value <0.001). The zinger motif peaks associated with stronger scoring ChIP-seq peaks than did the distal-zinger peaks for the majority of datasets (that is, 81%, 67%, and 79% of CTCF, JUN, and GABPA ChIP-seq datasets, respectively).

### Zinger motif peak regions recur across multiple TF datasets

As zinger motif peaks are enriched in numerous datasets for which the zinger is not the targeted TF, we asked whether the same zinger regions were occurring repeatedly across multiple datasets, that is, are the same zinger regions being ChIPped by many TFs. We pooled the zinger motif peaks, which by definition lacked the motif of the ChIPped TF, from across datasets (33 cell lines; 823,574 peaks), requiring that the zinger motif have a strong motif score of 85 or greater to reduce false positives. We assigned peaks whose peakMax were within 50 bp of each other into neighbourhoods (see Materials and methods), and then assessed the recurrence of each neighborhood, that is, the number of unique TFs whose datasets contributed a zinger motif peak to the neighbourhood.

**Figure 5:**
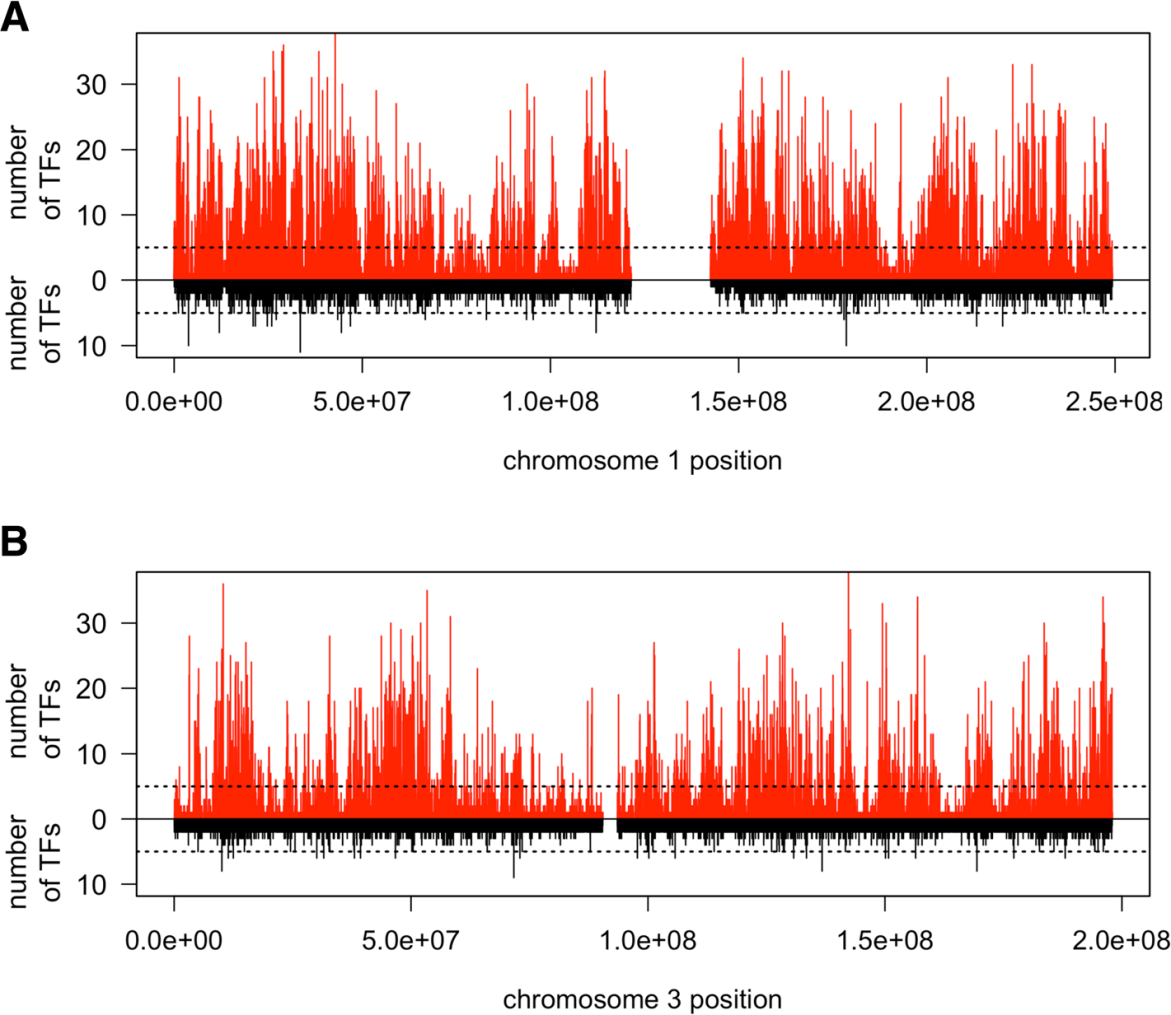
**Zinger motif peaks recur across datasets for multiple TFs.** The plots present two distinct neighbourhood sets (as defined in the text): one set derived from zinger motif peaks (red) and the other from ChIPped TF motif peaks without zinger motifs (black). The x-axis gives the neighborhood position on a chromosome: **(A)** chromosome 1, **(B)** chromosome 3. The y-axis is the number of unique TFs that ChIPped a peak in a neighborhood. A horizontal dotted line at y = 5 is given for visualization purposes, to highlight that there are many zinger neighborhood locations (red) that were ChIPped by multiple unique TFs.

We obtained 257,631 zinger neighbourhoods of which 92,244 neighbourhoods derived from regions ChIPped by two or more unique TFs. The neighborhoods ChIPped by two or more TFs are on average 167 bp in width (maximum 607 bp), and 77% derive from two or more cell lines. This amounts to approximately 15.4 Mbp of recurrently detected zinger motif associated sequence that was ChIPped by 2 to 41 non-zinger TFs in up to 21 cell lines. Figure 5 exemplifies the number of TFs that ChIPped zinger neighbourhoods across chromosomes 1 and 3 (zinger neighbourhood coordinates are provided in Additional file 12: Dataset S1).

We similarly generated neighborhoods from those regions with neither the ChIPped TF nor zinger motifs (unidentified motif neighborhoods - 536,546), and from the regions found to have a high scoring motif (score >85) for the ChIPped TF and no zinger motif (ChIPped TF neighborhoods - 408,677) (see Materials and methods). The zinger neighborhoods were found to be ChIPped by significantly more unique TFs than are the other two sets of neighborhoods (Wilcoxon one-tailed test *P* value = 0).

The recurrence of the zinger motif peaks across datasets prompted us to consider the motif content of HOT regions. HOT (high occupancy of transcription-related proteins) regions, as defined by Yip *et al.* [19], are ChIP-seq regions that within a single cell line (GM12878, HeLa, H1-hESC, HepG2, or K562) demonstrate binding co-occurrence among chromatin-related factors, general TFs, and sequence-specific TFs. Yip *et al*. noted that a substantial portion of a cell-line’s HOT regions are motif-less for the ChIPped factor, and associate with strong DNaseI signals. The HOT regions are present in two or more cell lines in 25% of cases according to Yip *et al*., while zinger neighborhoods were noted above to be 77% of cases. oPOSSUM over-representation analysis on the combined set of HOT regions found the zinger motifs to be 13 out of the 20 most enriched patterns, consistent with what was observed above for the DNaseI-seq/Faire-seq open chromatin datasets (Additional file 13: Table S1).

### Zinger neighbourhoods tend to occur close to regions occupied by cohesin

Recurring open chromatin enrichment across datasets suggested that structural properties of chromatin might contribute to zinger motif recovery across ChIP experiments [12]. Cohesin is a protein noted for both its role in gene regulation and DNA structure [20,21]. It is a multi-subunit complex, which is believed to form a ring like structure around DNA, and has been well documented in its role of sister chromatid interaction during the mitotic metaphase. Cohesin has also been implicated in promoting interaction between enhancers and core promoters of active genes in embryonic stem cells [21] and in chromosomal looping [22]. Chromosomal looping may be a structural element that is conducive to DNA shearing under the stress of sonication. Additionally, cohesin or associated proteins may function as a ‘loading station’ by bringing together proteins bound to remote regulatory elements and promoter regions that will in turn regulate transcription within the looped region [23].

We evaluated the proximity of the zinger neighborhoods to cohesin-interacting regions. Zinger neighborhoods are enriched for proximity (that is, within 500 bp) to cohesin regions (*via* RAD21 and SMC3 ChIP-seq) compared to the ChIPped TF neighborhoods or unidentified motif neighborhoods (Fisher exact one-tailed test *P* value of 0 for both comparisons; 77% of the zinger neighborhoods observed for multiple TFs are proximal, while 46% of the unidentified motif and 13% of the ChIPped TF neighborhoods are so positioned). The neighborhoods for unidentified motif peaks were also significantly more proximal to cohesin than neighborhoods from ChIPped TF peaks (Fisher exact one-tailed test *P* value of 0). As some of the neighborhoods contain CTCF zinger attributed regions, and cohesin is known to interact with CTCF [24,25], we removed neighborhoods within 500 bp of a CTCF ChIP-seq region and repeated the analysis. Regardless of the depletion of CTCF associated neighborhoods, the zinger neighborhoods remained significantly closer to cohesin (Fisher exact one-tailed test *P* value of 0 for all comparisons).

Another system noted to impact chromatin structure are the polycomb group proteins (including polycomb repressive complex 1 (PRC1) and polycomb repressive complex 1 (PRC2) forms), which are implicated in the remodeling of chromatin. In drosophila, PRC1 has been noted to interact with cohesin to co-regulate active genes [26]. We used ChIP-seq data for the constituent proteins CBX and EZH2 proteins to identify regions bound by the PRC1 and PRC2 complexes, respectively. We found that the zinger neighborhoods were significantly closer to CBX peaks and EZH2 peaks than are the neighborhoods derived from either ChIPped TF motif peaks, or from unidentified motif peaks (Fisher exact one-tailed test *P* value of 0). We observed that the PRC1 and PRC2 peaks proximal to the zinger neighborhoods, tend to be those that are also within 500 bp of cohesin (Fisher exact one-tailed test *P* value <7.6e-160 for PRC1, and *P* = 0 for PRC2). The unidentified motif neighborhoods are, in turn, significantly closer to PRC regions than the neighborhoods derived from peaks with the motif for the ChIP-seq experiment’s targeted TF.

Thus, the zinger neighborhoods, and to a lesser degree the unidentified motif neighborhoods, are associated with cohesin and polycomb repressive complex regions. This suggests that these diverse regions, which were initially identified as not containing the motif of the ChIPped TF, and yet in many cases enriched for an alternative motif (zingers), may be part of a structure involving cohesin. Such a structure could influence the tendency for these regions to be detected recurrently across diverse ChIP-seq data.

## Discussion

ChIP-seq experiments are increasingly used to investigate how sequence-specific DNA binding TFs regulate gene expression. In this report, we introduce ‘zingers’: four classes of TFBSs that display significant binding site enrichment, unexpectedly proximal to the peakMax, across ChIP-seq binding experiments for other TFs. Within individual TF ChIP-seq experiments, up to 45% of peaks are observed that lack the canonical TF binding motif and contain a zinger motif, with a mean of 12% (median 9%). While biased to the lower scoring peaks in other TF ChIP-seq data, the same zinger-associated regions tend to be high scoring peaks within datasets ChIPped for the zinger TF; indicating these regions are likely bound by the zinger TF. The zinger motif peaks derive from 257,631 regions (neighborhoods) in the genome, 36% of which are observed recurrently across datasets for diverse TFs, in sharp contrast to neighborhoods containing only the ChIPped TF’s motif, which recur relatively infrequently. Some regions lacking both the ChIPped TF’s motif and a zinger motif, are also recurrently observed. Both zinger motif and unidentified motif neighborhoods are positioned proximal to structural regions defined by the presence of cohesin and polycomb group complexes. Accounting for the contribution of zinger-associated regions to global studies of regulatory sequences will be a consideration for future analysis of ChIP-seq data.

**Figure 6:**
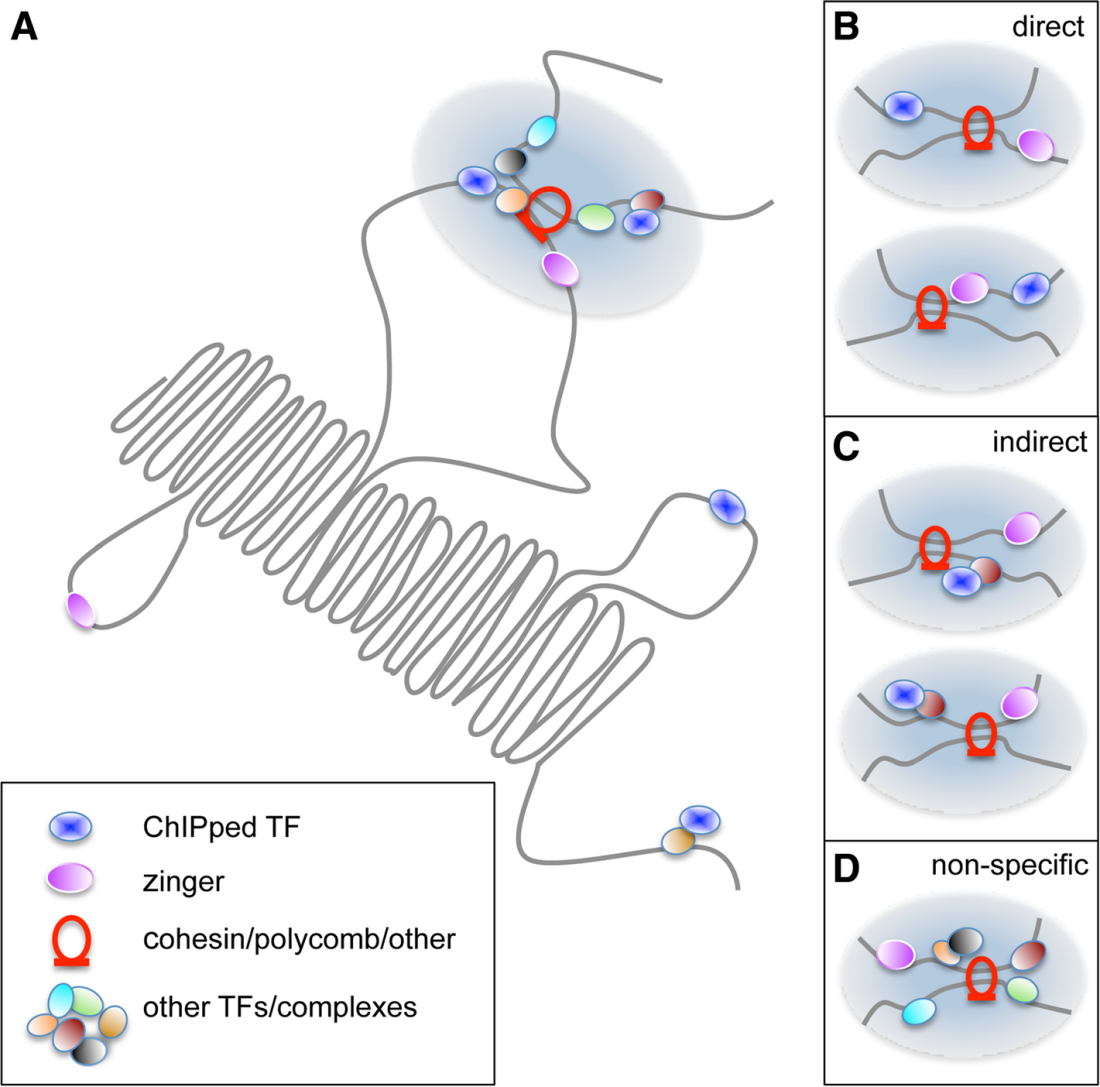
**A model to account for zinger motif enrichment across ChIP-seq datasets.** A TF loading station model is presented that is compatible with the observed enrichment of zinger motifs across diverse TF ChIP-seq data and cell lines. The dark blue oval represents the ChIPped TF, the magenta oval represents the zingers, the remaining coloured ovals represent TFs or other proteins or complexes that engage with the DNA, and the red loop represents cohesin and polycomb group proteins. The grey strands are chromatin. **(A)** Overview of a loading station. Multiple proteins may interact within a local region, from which TFs may disperse to search for other regulatory regions. Zingers and structural components such as cohesin and polycomb group proteins are key features. Panels B, C, and D present specific scenarios under which DNA loading station segments might be recovered in a ChIP-seq experiment. **(B)** Direct binding. The ChIPped TF directly binds to a TFBS, while a zinger motif is present in *trans* (upper) or in *cis* (lower). **(C)** Indirect binding. The ChIPped TF is present due to an indirect interaction, involving a mediating protein. The zinger motif is again present in *trans* (upper) or in *cis* (lower). **(D)** Non-specific events. Numerous proteins are present at the loading station, providing an abundance of epitopes, thus increasing the probability of being recovered in a ChIP-seq experiment.

Understanding the underlying biochemical mechanism by which the zinger-associated regions are observed across such diverse datasets remains to be resolved. However, based on the findings in this investigation, we present a ‘loading station’ model consistent with our state of understanding (Figure 6). Cohesin/polycomb and zinger proteins are proposed to participate in demarcation and stabilization of inter-segment interactions of DNA at which TFs bind. At these ‘stations’, the ChIPped TF may be present via direct (Figure 6B) or indirect (Figure 6C) interactions with the DNA, and either in *cis*- or *trans*- arrangements with a zinger TFBS. In a ChIP experiment, assuming covalent linking of the ChIPped TF and the cohesin-paired DNA, the patterns of motif enrichment observed in this report could emerge, including the presence or absence of motifs for both the ChIPped TF and a zinger. Alternatively, or possibly in combination, there may exist zinger-containing regions (Figure 6D) at which many proteins are present (at a cell population level). Such regions may contain a diverse range of epitopes and therefore be more likely to be recovered in ChIP-seq experiments, especially with polyclonal antibodies. Within this model, TFs may ‘visit’ cohesin and zinger marked regions, resulting in a low but consistent recovery of reads in a ChIP-seq experiment. The model accounts for recurring detection of zinger motif peaks, the proximity of the peaks to cohesion interacting regions, and why the zinger motifs may be present in the sequence even when the ChIPped TF’s motif is absent.

From a broader mechanistic perspective, a loading station mechanism is consistent with the ‘hop-skip-jump’ theory for how TFs efficiently search the nucleus to arrive at their TFBSs [27]. The proposed loading station model is supported in recent literature. Faure *et al.* [23] propose a role for cohesin in stabilizing large protein-DNA complexes. While this manuscript was under review, Taipale *et al.* [28] published a study using the LoVo cell line suggesting that cohesin participates in holding chromatin open during cell division to facilitate TFs relocating back to those regions once division is complete.

The zinger content of every ChIP-seq dataset should be evaluated, consistent with a growing effort to critically evaluate such data [12,29,30]. For instance, the STAT1 (GM12878) ChIP-seq dataset exceeds 30% of peaks with zinger motifs proximal to the peakMax, while STAT1 motifs occur only at the background frequency. We propose a general approach for the study of zinger content. For each ChIP-seq dataset, the peak regions should be scanned for the presence of the ChIPped TF motif in proximity to the peakMax. The peaks lacking a ChIPped TF motif should be compared to the recurring zinger neighborhoods (Additional file 12: Dataset S1). The portion of the dataset overlapping the neighborhoods gives insight into the overall specificity of the experiment.

## Conclusions

We have identified zinger motifs that are frequently enriched across a portion of TF ChIP-seq data, including CTCF-like, ETS-like, and JUN-like motif families, and THAP11. As high-throughput ChIP-seq data informs genome annotation, research into gene regulation may be impacted by zinger motif derived annotations. Moving forward it will be important to determine the prevalence of zinger-like motifs in ChIP-seq data in diverse organisms, probe the structural properties of the zinger regions, and develop computational approaches to systematically identify recurring zinger regions in large-scale genome annotation. Ultimately, understanding the biophysical processes that result in the zinger motif enrichment in ChIP-seq data may provide broader insight into the mechanisms of transcription regulation.

## Materials and methods

### Datasets

For our analyses, we used ENCODE ChIP-seq datasets (human and mouse), ENCODE DNaseI-seq and Faire-seq data, and human ChIP-seq controls [1] downloaded from the UCSC ENCODE database [31]. We also incorporated non-ENCODE ChIP datasets downloaded from GEO: (1) GSE11431 - 13 mouse ESC datasets [32]; (2) GSE25532 - mouse NFYA data in ES cells [33]; (3) GSE17917 and GSE18292 - human KLF4, POU5F1, cMYC, NANOG, and SOX2 data [34]; and (4) GSE22078 - human and mouse CEBPA and HNF4A [35]. Where only the mapped data were available, we used FindPeaks 4.0 [36] to call peaks using the following parameter options: dist_type 1 200 -subpeaks 0.6 -trim 0.2 -duplicatefilter. The ENCODE broadPeak datasets frequently occurred in replicate; to avoid duplication, only the replicate with the most peaks of a pair was used for analyses.

Where coordinates were provided as NCBI36/hg18 or NCBI36/mm8, they were first converted to GRCh37/hg19 or NCBI37/mm9, using a locally installed version of the UCSC lift-over tool [37]. We then used the Ensembl API to retrieve sequences from GRCh37/hg19 and NCBI37/mm9 assemblies.

The ENCODE ChIP-seq data are in one of two formats, narrowPeak and broadPeak. Both formats contain two columns pertaining to statistical significance of the peaks (also known as peak scores): one is a *P* value, the other a q value (bonferroni corrected). We used the q value field when it was assigned, and otherwise used the *P* value field.

As peaks are reported in a multitude of lengths, in the range of 1 bp to greater than 5,000 bp, we trimmed or extended all peaks to a constant length centered at the peak maximum for narrowPeak format datasets, or at the peak centre for broadPeak format and DNase-seq/Faire-seq datasets. For enrichment visualization and determining heuristic boundaries of enrichment we used 1,001 bp sequences, oPOSSUM TFBS enrichment analysis input was 401 bp sequences, and *ab initio* motif detection input was 201 bp sequences.

Position frequency matrices (PFMs) were obtained from the JASPAR [38] development 4.0_alpha database of transcription factor models (prior to 2013, April). Where the JASPAR PFM did not agree with the consensus in the literature we performed an *ab initio* analysis on the top 500 peaks (selected by peak score) of two or more ChIP-seq datasets for the given TF, using a locally installed version of the MEME software [8]. MEME results were then checked against the literature and for enrichment in a different ChIP-seq dataset for the given TF. MEME position specific probability matrices (PSPM) were converted to PFMs by transposing the PSPM and multiplying each letter (A, G, C, T) frequency in the matrix by the number of sites found by MEME. The PFMs were subsequently converted to position weight matrices (PWMs), using the TFBS Perl Module [39], only PWMs based on PFMs with information content (IC) greater than 8 bits were retained. The PFMs used in this study are provided in Additional file 14: Dataset S2.

For those analyses using datasets of shuffled matrices, the datasets were generated by random permutation of all columns of the originating PFMs, excluding the lower information content columns on the edges (2 columns on each side for all cases, except for the wider CTCF PWM for which 3 columns on each side were held constant).

### Motif over-representation analysis

Motif over-representation analyses were performed with a locally installed version of oPOSSUM 3.0 [9]. We used the sequence-based analysis option with default settings, except for specifying the use of the JASPAR development PFM matrices (Additional file 14: Dataset S2). We trimmed or extended all peaks to 401 bp. Backgrounds for the over-representation analyses came from the mappable portion of the genome, and were chosen to match the sequence length and mononucleotide GC composition distribution of each dataset.

The oPOSSUM Fisher-log enrichment score is derived from a one-tailed Fisher exact probability test, based on the hypergeometric distribution which compares the number of sequences that contain a motif for the TF of interest in the target and background datasets. The negative natural logarithm of the Fisher test probabilities is the reported Fisher-log score. Thus a Fisher-log score of 6.91 or higher is equivalent to a *P* value of 0.001 or lower. Fisher-log enrichment scores of ‘infinite’ value were set to either 500 or to 100 past the maximum non-infinite Fisher-log score.

The oPOSSUM KS centrality score is the negative logarithm of the probabilities from a Kolmogorov-Smirnov test. Thus a KS score of 6.91 or higher is equivalent to a *P* value of 0.001 or lower. The Kolmogorov-Smirnov tests whether a TF’s motifs are positionally enriched at the center of the target sequences relative to the motifs in the background set of sequences. KS ‘infinite’ enrichment scores were set to 100.

To calculate the number of datasets enriched for a motif we first obtained the average Fisher-log score and KS log score for datasets ChIPped for the same TF. Once we had a set of scores for each TF, we used a binary count of 1 or 0 to indicate whether both of the oPOSSUM enrichment scores passed a threshold based on the standard deviation (SD) of the scores or not (two SD for Fisher-log scores and one SD for KS log scores). This yielded the number of datasets with enrichment around the sequence midpoint for each of the 165 TFs. We then applied a further correction to compensate for the bias created by multiple datasets for families of TFs that recognize the same motif (for example, JUN, JUND, JUNB, AP1, FOS, FOSL1, FOSL2, and BATF PWMs all recognize a TGA(g/c)TCA consensus). The number of motif-family members, minus one, was subtracted from the count of datasets for each of the member TFs, for example, if JUNB were enriched in 20 TF datasets, and 9 of those datasets were ChIPped for a TF that recognizes the JUN-motif family consensus, then a count of eight would be subtracted from 20. The 165 TFs were then ranked according to this final number of associated datasets.

### Motif over-representation analysis with shuffled matrices

To assess the probability of a PWM’s predictions being enriched within as many datasets as observed with the zinger PWMs, we shuffled the PFMs of the zingers and fit a distribution to the results. We generated 100 shuffled matrices as described above. We performed oPOSSUM enrichment analyses with the shuffled PWMs, on the same human datasets as used to generate Figure 1. The oPOSSUM results were evaluated as outlined above. However, we applied the enrichment score thresholds for each dataset as was set for the original PWMs. We then counted the number of datasets within which each shuffled profile was enriched, and fit a zero-adjusted logarithmic distribution (ZALG) to the counts. The distribution was selected using the fitDist() function in the R statistical package GAMLSS 4.1-5 [40], and the parameters describing the distribution were obtained with gamlss family ZALG and the gamlss() function. We tested for goodness-of-fit of the distribution to the data by generating datasets from the random generation function, rZALG, and assaying the similarity of the generated distributions to our data using a chi-squared test. The fitted distribution function was then used to determine the probability of the shuffled PWMs obtaining a result as extreme as the original PWM. The probability was calculated with the density function for the zero-adjusted logarithmic distribution (dZALG).

### Motif prediction

Motif prediction was performed with C-code adapted from the TFBS Perl Module [39], reporting relative motif scores. Motifs predicted by a PFM are not permitted to overlap by more than one-fifth the PFM length (this setting is intended to equate to the low information content flanks of a PWM), for example, a 7 bp motif could only overlap a neighboring motif by 1 bp.

For post-oPOSSUM analyses, we predicted the presence of zinger motifs using one PWM per zinger TF motif family as proxy, to prevent redundancies. CTCF-like motifs were predicted with the CTCF PWM, ETS-like motifs with the GABPA PWM, JUN-like motifs with the JUN PWM, and THAP11 motifs with a THAP11 PWM.

### MEME suite tools

MEME [8] analyses were run using the following options: -dna -nmotifs 10 -minw 6 -maxw 15 -maxsize 2000000 -mod zoops -revcomp. TOMTOM [14] analyses were run with default values, aside from increasing the E-value threshold to 20, from the web server.

### Repeat-masking

Masking of repeat elements was performed using a local installation of RepeatMasker (RMBlast) [41] and RepBase [42], using default settings.

### Data processing and statistical analyses

Data processing and statistical analyses were done with a combination of in-house Unix and R scripts (R version 2.14.1) [40]. Throughout the manuscript we report the combination of median and the median absolute deviation (MAD), a measure of dispersion around the median. For a normal distribution the median and MAD are the same values as the mean and SD.

### TFBS-landscape visualization plots

To visualize peakMax proximal enrichment of TF motifs within ChIP-seq datasets, the top scoring predicted motif in each region for the given TF PWM, was plotted relative to its signed distance from the peakMax (using the R basic statistical package [40]). The dense horizontal ranges of motif scores spanning all positions relative to the peakMax, such as seen in the Figure 2 plots, are observed for the combination of most PWMs and ChIP-seq datasets, and are likely a mixture of both false and true TFBS predictions. Those motif matches that are distal to the peakMax are anticipated to be less reliable, as the observed frequency is consistent with background rates of motif prediction. If we take enrichment proximal to the peakMax as a measure of confidence for the predictions we can determine a distance threshold and motif score threshold (see next section) at a point where motif frequency proximal to the peakMax is greater than the flanking distal motif frequency. Using this threshold, we can select a sub-population of peaks that are less likely to have arisen by chance.

### Heuristic boundaries of enrichment

We assessed the enrichment of motif distance to the peakMax and motif score, using a heuristic method for topological motif enrichment [18], which we outline in brief here. To determine whether a motif was proximal to the peakMax, we used heuristic distance boundaries derived from the density of the top scoring motif for each 1,001 bp region. We identified the location, relative to the 501st bp, at which the density of motifs exceeds that of the distal region (approximately 175 to 500 bp distant from the peakMax). This change in density is observed in the TFBS-landscape plots of Figure 2, where there is a constant density of motif scores in the distal regions and an increase in the density of motif scores within approximately 100 bp of the peakMax. The heuristic distance boundaries were set at the transition point. A similar procedure was applied to determine a threshold for the motif score, where the motif score threshold was set at the point where the motif enrichment proximal to the peakMax was at least 20% higher than the flanking enrichment. The region defined by the distance boundaries and the motif score threshold, was termed the ‘enrichment zone’. The enrichment zone was subsequently used to specify peakMax enriched proximal motifs. On average, an enrichment boundary was ±90 bp from the peakMax, and the motif relative score threshold was 82.

The heuristic analysis of motif enrichment across datasets reports that on average a CTCF zinger motif is enriched above a motif score threshold of 79, while for JUN the average was 86, for GABPA it was 83, and for THAP11 it was 84. CTCF and THAP11 in particular consistently have enrichment above a motif score threshold of 85 that is strongly distinct from the flanking regions of similar score range, as seen in Figure 2A and D. The regions that flank the peakMax proximal enrichment in Figure 2 are representative of the background expectation of a PWM’s motif prediction. Thus, to reduce the presence of false positive predictions in subsets of peaks we analyzed, we selected, where noted in the main text, peaks with a motif scoring above the motif score threshold of 85. The use of a single threshold permits the processing of data as a single unit. A motif score of 85 is also the default threshold score in the oPOSSUM software.

### Background expectation of motif predictions

To estimate the proportion of regions in a given dataset in which motifs may result from background motif prediction, we compared the count of regions with motifs in the enrichment zone relative to the count of regions with motifs at least 50 bp outside the enrichment zone. The distal ‘zone’ from which counts were determined, was set to be the same length of sequence as the enrichment zone, that is, if the enrichment zone was 200 bp wide, then the distal zone was also 200 bp wide (100 bp from 5′ and 100 bp from 3′ of the region center). To estimate the proportion of regions in the enrichment zone with false positives, we divided the number of regions with motifs in the distal zone by the number of regions with motifs in the enrichment zone. See Additional file 4: Text S1 for the estimated overall background expectation of ChIPped TF and zinger motif prediction.

Calculating the background corrected estimates of ChIPped TF and zinger motif proportions within a dataset was done by subtracting the distal zone count from the enrichment zone count for the ChIPped TF or each zinger. For the ChIPped TF, the corrected count was divided by the size of the dataset. For the four zingers, the four corrected counts were first summed, and then divided by the size of the dataset.

### Heatmaps and correlation between zinger motifs

Heatmaps were created with the heatmap.2() function from the R statistical package: gplots, with the distance measure as ‘manhattan’ and the ‘ward’ agglomeration method for clustering.

The heatmap of zinger motif peak log_2_ fold enrichment was generated using the log_2_ fold enrichment of zinger motif peaks with motif score 85 or greater, relative to distal-zinger peaks of similar score range. Where the fold enrichment was below 1.5 we assigned a minimum value, represented as a grey colour in the heatmap, to facilitate visualization.

A heatmap of zinger motif inter-dependency within datasets was generated using the set of zinger motif peaks with motif scores equal to or greater than 85, and a 2×2 confusion matrix for each pair of zinger motifs. A Fisher exact *P* value <0.001 was taken to indicate significance and the sign of the log odds ratio to indicate whether a positive or negative association existed. The values used to generate the heatmap were 1-pvalue for positive associations, -1*(1-pvalue) for the negative associations, and 0 for the non-significant *P* values.

The pairwise correlation of zinger motif peaks for the different zingers, across datasets, was assessed using the log_2_ fold enrichment values generated for the above heatmap. The correlations were evaluated with both Pearson correlation and Spearman’s rank order correlation (R basic statistical package: cor() function).

### ChIP-seq controls

We obtained controls from a range of cell types and ENCODE consortium groups, and processed the mapped reads with FindPeaks. We used the peak height to rank the control peaks, and then selected the top 70,000 peaks. The number of peaks was chosen to match the average size of the ChIP-seq datasets. The peaks were then scored with the zinger PWMs and the enrichment of the motifs with respect to the peakMax was evaluated.

### Evaluating proximity of zinger motif peaks to genomic features

We compared the genomic feature proximity of zinger motif peaks, with those peaks containing the ChIPped TF’s motif and lacking zinger motifs. We measured the distance between the peakMax and the middle of the feature, which in the case of transcription start sites (TSSs) was simply the starting coordinate of the transcript. We used only those datasets for which we had at least 200 zinger motif peaks. The number of peaks that were within 500 bp, 1 kb or 5 kb of the TSS, or within 500 bp of CpG islands, conserved regions or repeat-masked regions were compared between the zinger motif peaks and the ChIPped TF peaks using a Fisher exact test. For the results of a zinger to be considered striking we required that at least 60% of the datasets with zinger motifs show statistical significance in one direction, that is, either 60% of datasets tend to be proximal to a feature, or 60% of datasets tend to be distal to a feature.

### Comparing zinger regions from non-zinger ChIP-seq datasets to peaks ChIPped by the zinger TF

We assessed the proximity of the zinger motif peaks with a high scoring zinger motif (score >85) to ChIP-seq peaks ChIPped by the zinger’s TF to determine whether the zinger motif peaks found in datasets for which the zinger is not the targeted TF, are potential *bona fide* binding regions for the zinger TF. In all cases we required that the zinger motif peaks and zinger TF’s ChIP-seq data be from the same cell line. To call a zinger motif peak in agreement with the zinger TF’s ChIP-seq data we required that the peakMax of the zinger motif peak be within 100 bp of a peakMax in the zinger TF’s dataset. This 100 bp distance reflects the average range of enrichment for a TF’s motif relative to the peakMax. The assessment of the distal-zinger peaks, that is, those peaks with motifs not proximal to the peakMax, relative to the zinger TF’s ChIP-seq dataset was performed in the same manner.

### Generation of ChIP-seq peak neighborhoods

To determine the degree of recurrence for a zinger motif peak region across multiple datasets we pooled all zinger motif peaks that had a high scoring (score >85) zinger motif from all datasets. We then calculated the inter-zinger distances between each zinger motif peak and its nearest neighbour in the 3′ direction on the plus strand. Consecutive peaks that were within 50 bp of their nearest neighbor were merged into a ‘zinger neighborhood’. The distance of 50 bp was chosen as a stringent measure of proximity between zinger motif peaks. For each neighborhood, we counted the number of unique TFs that ChIPped the zinger motif peaks and the number of unique cell lines. We provide the coordinates for the zinger neighborhoods in Additional file 12: Dataset S1.

We generated neighborhoods from the remaining two groups of peaks in a similar manner: those with the ChIPped TF motifs and lacking zinger motifs (‘ChIPped TF neighborhoods’), and those without either motif (‘unidentified motif neighborhoods’). For the ChIPped TF neighborhoods we required that there be a high scoring motif (score >85) for the ChIPped TF. Neighborhood widths were <150 bp on average. As stated in the main text, zinger motif peaks may be *bona fide* binding regions for the zinger TF. Thus, after generating the neighborhood sets, we removed from the ChIPped TF neighborhoods those regions that were within 300 bp (measured centre to centre of the zinger neighborhoods to ensure that comparisons were made between distinct neighborhood sets. We also removed from the ChIPped TF neighborhoods those regions that overlapped the unidentified motif neighborhoods in the same manner.

### Neighborhood proximity to cohesin and polycomb repressive complex

To assess whether a neighborhood is proximal to a region occupied by cohesin or the polycomb repressive complex (PRC) 1 or 2, we generated three datasets by combining the ENCODE ChIP-seq data for the cohesin proteins, RAD21 and SMC3, into a one dataset; combining the ENCODE ChIP-seq data for CBX to form a dataset for PRC1 occupancy, and lastly combining EZH2 ChIP-seq data into a dataset for PRC2 occupancy. We then assessed how many zinger neighborhoods were situated within 500 bp of one of the three protein complexes, measuring from the center of a neighborhood to the ChIP-seq peakMax, and compared this to the two other neighborhoods.

## Additional files

Additional file 1: Figure S1. Zinger motifs are enriched across multiple mouse ChIP-seq datasets. **(A)** The histogram displays the results of TFBS motif enrichment analysis on 81 mouse ChIP-seq datasets generated with the oPOSSUM 3.0 software. Along the x-axis is the fraction of datasets that displayed enrichment near the peakMax for a TF profile. The y-axis is the number of TF profiles that were found enriched for a given fraction of datasets. The profiles most frequently observed to be enriched are labeled on the histogram. **(B)** The binding site logos of the nine TF binding models with enriched motifs across the greatest number of datasets, manually grouped by motif similarity. Each logo depicts position along the x-axis and information content (that is, pattern strength) along the y-axis. Additional file 2: Figure S2. Zinger motifs are enriched across multiple human datasets after masking the ChIPped TF’s motif. **(A)** The histogram displays the results of TFBS motif enrichment analysis on 281 human ChIP-seq datasets in which the ChIPped TFs motifs were masked. Results were generated with the oPOSSUM 3.0 software. Along the x-axis is the fraction of datasets that displayed enrichment for a TF profile. The y-axis is the number of TF profiles that were found enriched near the peakMax for a given fraction of datasets. The profiles most frequently observed to be enriched are labeled on the histogram. **(B)** The binding site logos of the TF binding models with enriched motifs across the greatest number of datasets, manually grouped by motif similarity. Each logo depicts position along the x-axis and information content (that is, pattern strength) along the y-axis. Additional file 3: Figure S3. DNaseI-seq and Faire-seq datasets are enriched for zinger motifs. The histograms display the results of TFBS motif enrichment analysis on **(A)** DNaseI-seq datasets and **(B)** Faire-seq datasets. Results were generated with the oPOSSUM 3.0 software. Along the x-axis is the fraction of datasets that displayed enrichment for a TF profile. The y-axis is the number of TF profiles that were found enriched for a given fraction of datasets. The profiles most frequently observed to be enriched are labeled on the histogram. **(C)** The binding site logos of the TF binding models with enriched motifs across the greatest number of either DNaseI-seq or Faire-seq datasets. The logos are manually grouped by motif similarity, except for the bottom row. Each logo depicts position along the x-axis and information content (that is, pattern strength) along the y-axis. Additional file 4: Text S1. Additional observations regarding zinger motifs and zinger motif peaks. Additional file 5: Figure S4. ChIP-seq datasets for non-sequence-specific proteins are enriched for zinger motifs. The enrichment plots display the location of the top scoring motif for each peak relative to the peakMax (the peakMax is at 0) on the x-axis, while the score of the motif is plotted on the y-axis. The adjacent line plots display the fraction of motifs observed in 5 bp increments. The logo reflecting the binding specificity for each zinger appears above the related enrichment plot. **(A)** CTCF motif predictions on ChIP-seq data for WHIP, a helicase interacting protein. **(B)** JUN motif predictions on ChIP-seq data for p300, a histone acetyltransferase. **(C)** GABPA motif predictions on ChIP-seq data for CCNT2, a cyclin regulator of CDK9 kinase. **(D)** THAP11 motif predictions on ChIP-seq data for CHD2, a chromodomain helicase. Additional file 6: Figure S5. Input and mock-IP control data are enriched for zinger motifs. The enrichment plots display the location of the top scoring CTCF motif for each peak relative to the peakMax (the peakMax is at 0) on the x-axis, while the score of the motif is plotted on the y-axis. The adjacent line plots display the fraction of CTCF motifs observed in 5 bp increments. The logo reflecting the binding specificity for CTCF appears above the related enrichment plot. **(A)** Input regions from the HUVEC cell line. **(B)** IgG rabbit mock-IP regions from GM12878 cells. Additional file 7: Figure S6. Shuffled zinger PWMs are not enriched proximal to the peakMax. The enrichment plots display the location of the top scoring motif for each peak relative to the peakMax (the peakMax is at 0) on the x-axis, while the score of the motif is plotted on the y-axis. The adjacent line plots display the fraction of motifs observed in 5 bp increments. The logo reflecting the binding specificity for each zinger appears above the related enrichment plot. **(A)** Enrichment of CTCF motifs on the NRF1 (GM12878) dataset. **(B)** Enrichment of shuffled-CTCF motifs on the same NRF1 (GM12878) dataset. **(C)** Enrichment of JUN motifs on the TCF7L2 (Hct116) dataset. **(D)** Enrichment of a shuffled-JUN motif on the same TCF7L2 (Hct116) dataset. Additional file 8: Figure S7. Untreated STAT1 ChIP-seq data show strong zinger motif enrichment and not STAT1 motif enrichment. The enrichment plots display the location of the top scoring motif for each peak relative to the peakMax (the peakMax is at 0) on the x-axis, while the score of the motif is plotted on the y-axis. The adjacent line plots display the fraction of motifs observed in 5 bp increments. The logo reflecting the binding specificity for each zinger appears above the related enrichment plot. **(A)** STAT1 motif predictions on STAT1 ChIP-seq from untreated GM12878 cells. No STAT1 motif. **(B)** CTCF motif predictions on STAT1 ChIP-seq from untreated GM12878 cells. **(C)** STAT1 motif predictions on STAT1 ChIP-seq from IFNγ treated HeLa cells. STAT1 motif is present. **(D)** CTCF motif predictions on STAT1 ChIP-seq form IFNγ treated HeLa cells. Additional file 9: Figure S8. The distribution of zinger motif content varies across ChIP-seq datasets. **(A, B)** For those datasets with at least a 1% zinger component, the histograms present the distribution of observed zinger motif peak content. The x-axis reports the proportion of zinger motif peaks within an analyzed dataset, and the y-axis the frequency of such observations. The black vertical dashed line represents the mean, the blue vertical dashed line represents the median, and the red vertical dashed line represents the point where two-thirds of the datasets are to the right of the line. The asterisk indicates the maximum zinger proportion, excluding outliers. **(A)** Analysis performed on entire ChIP-seq datasets. **(B)** Analysis on the set of peaks unaccounted for by the ChIPped TF motif. **(C)** A heatmap of the individual zingers’ motif peaks log_2_ fold enrichment in the set of peaks unaccounted for by the ChIPped TF and with a strong motif score (score 85 or greater). Fold enrichment less than 1.5 is grey. The rows are individual datasets, the columns are the zingers. **(D)** A scatterplot of the proportions of zinger motif peaks (y-axis) and ChIPped TF motif peaks (x-axis) in each dataset. Additional file 10: Figure S9. The proportion of a dataset with zinger motifs is not dependent on cell-line nor the ChIPped TF. **(A)** The x-axis is the proportion of datasets composed of zinger motif peaks. The y-axis is a density value reflecting the fraction of datasets with zinger motifs. The five cell lines are K562 (black), GM12878 (blue), HeLa (red), H1-hESC (green), and HepG2 (magenta). There are no significant differences between the distributions per Wilcoxon test *P* values. **(B)** The TFs analyzed are listed on the horizontal access. The y-axis is the maximum difference of zinger proportions observed between two ChIP-seq datasets for the same TF. Additional file 11: Figure S10. Zinger motifs and zinger motif peaks are not strongly correlated. **(A)** A heatmap of significance for inter-dependence between pairs of zinger motifs in zinger motif peaks. Positive associations with a significant Fisher exact *P* value (*P* value <0.001) are yellow, negative associations with a significant Fisher exact *P* value are red, and non-significant *P* values are grey. The color density reflects *P* value significance, with the densest colors being *P* values closest to 0. The columns are individual datasets; the rows are the six possible zinger pairs. **(B)** A correlation matrix presenting both Spearman’s rank (lower diagonal) and Pearson (upper diagonal) correlation coefficients for the pairwise association of zinger motif peak enrichment within the same ChIP-seq datasets. Additional file 12: Dataset S1. Genomic coordinates for zinger neighborhoods (tab delimited). Additional file 13: Table S1. The top 20 motifs from motif over-representation analysis on HOT regions. Additional file 14: Dataset S2. Position frequency matrices.

## Abbreviations

ChIP-seq: Chromatin immunoprecipitation and high-throughput sequencing
peakMax: Peak local maximum
PFM: Position frequency matrix
PWM: Position weight matrix
TF: Transcription factor
TFBS: Transcription factor binding site
