# Supplementary figures and images for: Non-targeted transcription factors motifs are a systemic component of ChIP-seq datasets
Source: Genome Biol. 2014 Jul 29;15(7):412. doi: 10.1186/s13059-014-0412-4 (PMC4165360; doi:10.1186/s13059-014-0412-4)

**A**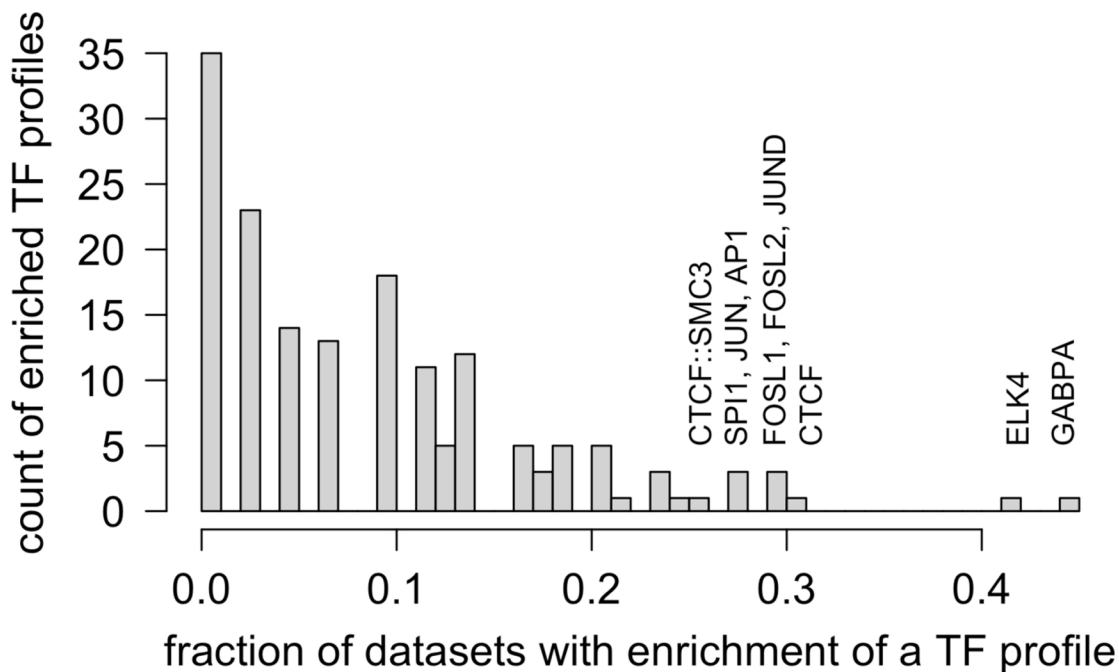**B**

ETS-like

JUN-like

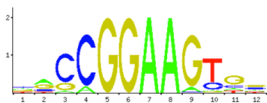

GABPA

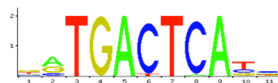

JUND

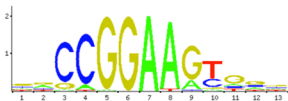

ELK4

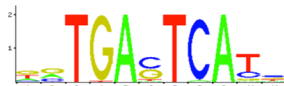

FOSL2

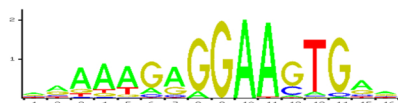

SPI1

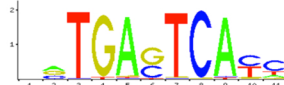

FOSL1

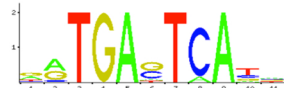

JUN

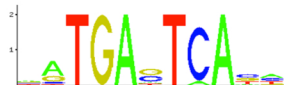

AP1

CTCF-like

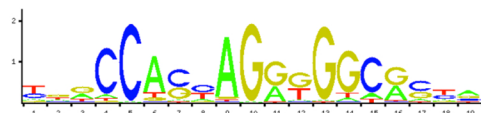

CTCF

Supplement: Additional file 1: Figure S1. — Zinger motifs are enriched across multiple mouse ChIP-seq datasets. (A) The histogram displays the results of TFBS motif enrichment analysis on 81 mouse ChIP-seq datasets generated with the oPOSSUM 3.0 software. Along the x-axis is the fraction of datasets that displayed enrichment near the peakMax for a TF profile. The y-axis is the number of TF profiles that were found enriched for a given fraction of datasets. The profiles most frequently observed to be enriched are labeled on the histogram. (B) The binding site logos of the nine TF binding models with enriched motifs across the greatest number of datasets, manually grouped by motif similarity. Each logo depicts position along the x-axis and information content (that is, pattern strength) along the y-axis. [file 13059_2014_412_MOESM1_ESM.pdf]

**A**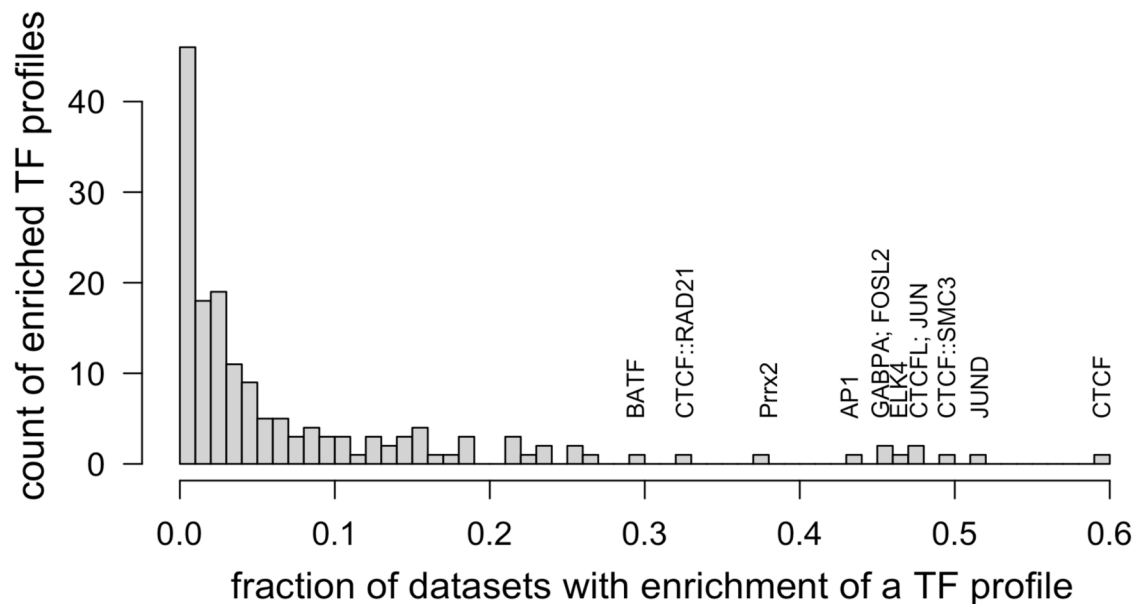**B**

CTCF-like

JUN-like

PRRX2

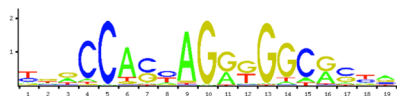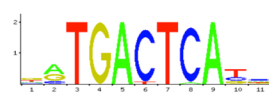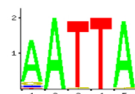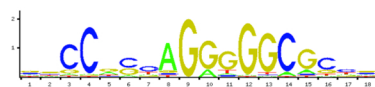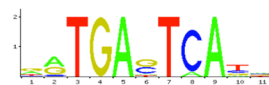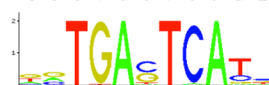

ETS-like

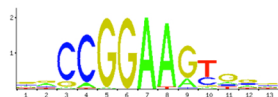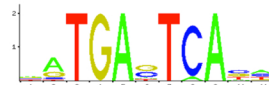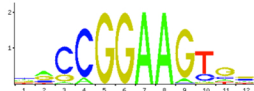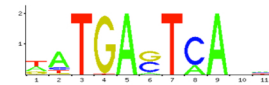

Supplement: Additional file 2: Figure S2. — Zinger motifs are enriched across multiple human datasets after masking the ChIPped TF’s motif. (A) The histogram displays the results of TFBS motif enrichment analysis on 281 human ChIP-seq datasets in which the ChIPped TFs motifs were masked. Results were generated with the oPOSSUM 3.0 software. Along the x-axis is the fraction of datasets that displayed enrichment for a TF profile. The y-axis is the number of TF profiles that were found enriched near the peakMax for a given fraction of datasets. The profiles most frequently observed to be enriched are labeled on the histogram. (B) The binding site logos of the TF binding models with enriched motifs across the greatest number of datasets, manually grouped by motif similarity. Each logo depicts position along the x-axis and information content (that is, pattern strength) along the y-axis. [file 13059_2014_412_MOESM2_ESM.pdf]

**A**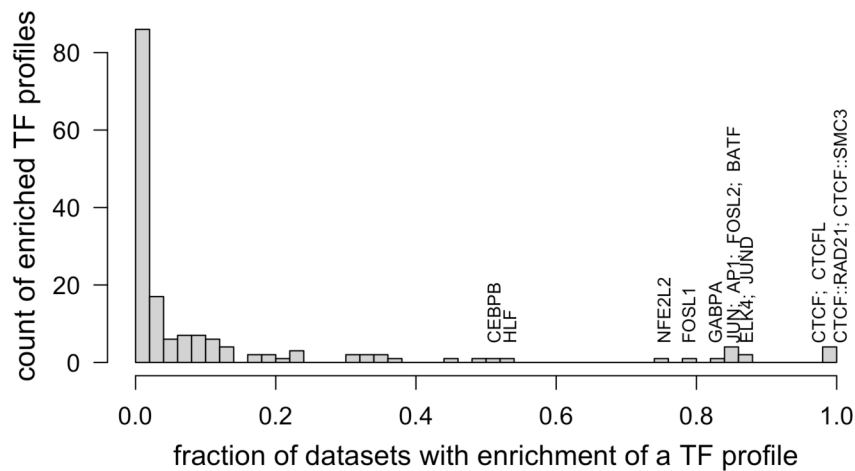**B**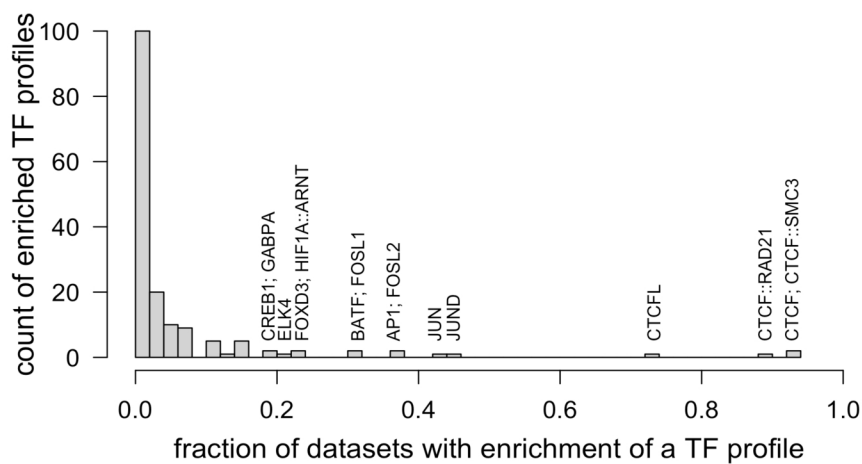**C**

CTCF-like

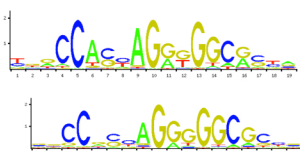

JUN-like

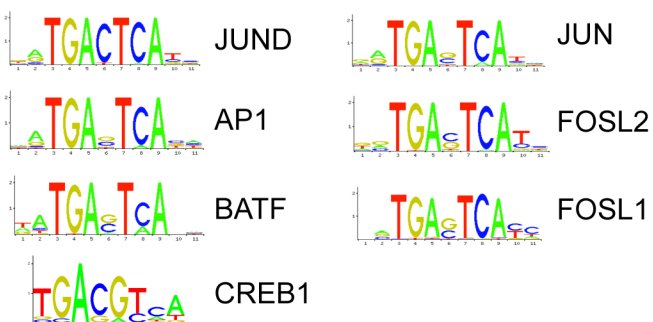

ETS-like

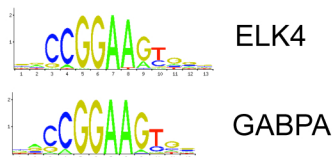

NFE2L2

HLF

CEBPB

FOXD3

HIF1A::ARNT

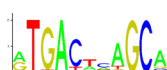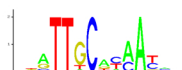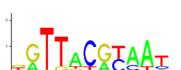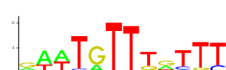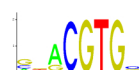

Supplement: Additional file 3: Figure S3. — DNaseI-seq and Faire-seq datasets are enriched for zinger motifs. The histograms display the results of TFBS motif enrichment analysis on (A) DNaseI-seq datasets and (B) Faire-seq datasets. Results were generated with the oPOSSUM 3.0 software. Along the x-axis is the fraction of datasets that displayed enrichment for a TF profile. The y-axis is the number of TF profiles that were found enriched for a given fraction of datasets. The profiles most frequently observed to be enriched are labeled on the histogram. (C) The binding site logos of the TF binding models with enriched motifs across the greatest number of either DNaseI-seq or Faire-seq datasets. The logos are manually grouped by motif similarity, except for the bottom row. Each logo depicts position along the x-axis and information content (that is, pattern strength) along the y-axis. [file 13059_2014_412_MOESM3_ESM.pdf]

**A**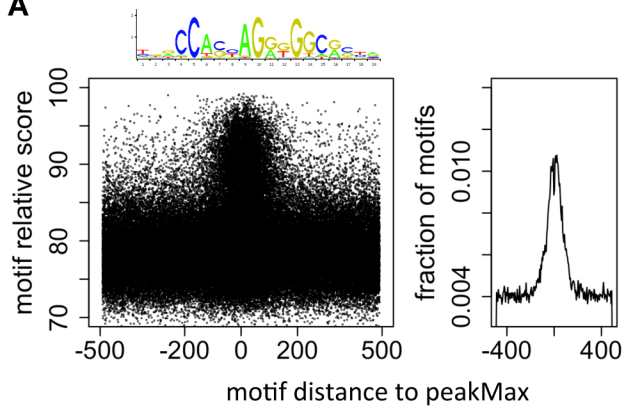**B**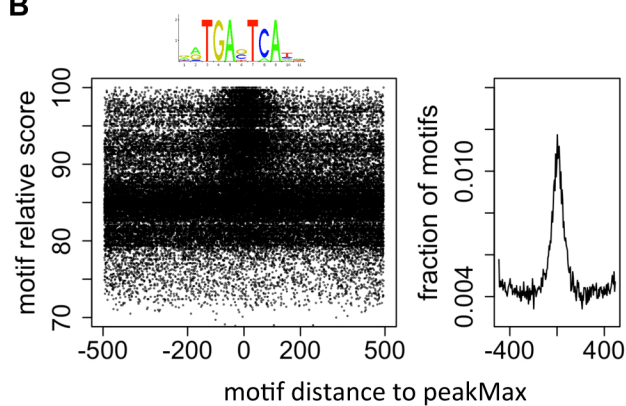**C**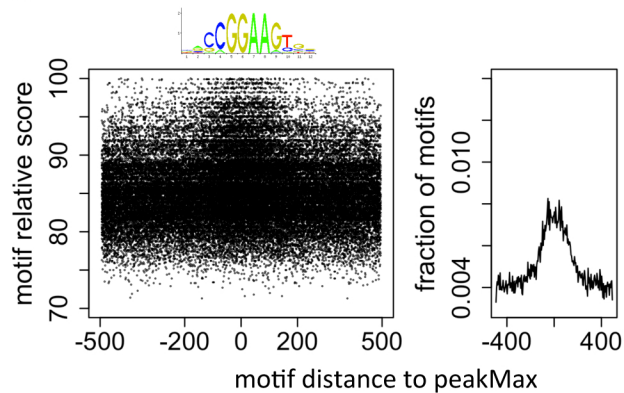**D**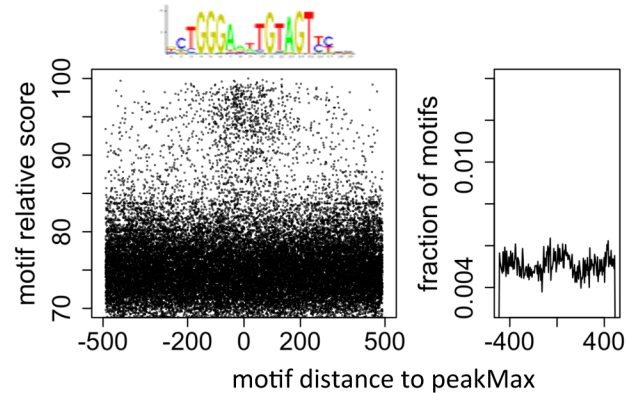

Supplement: Additional file 5: Figure S4. — ChIP-seq datasets for non-sequence-specific proteins are enriched for zinger motifs. The enrichment plots display the location of the top scoring motif for each peak relative to the peakMax (the peakMax is at 0) on the x-axis, while the score of the motif is plotted on the y-axis. The adjacent line plots display the fraction of motifs observed in 5 bp increments. The logo reflecting the binding specificity for each zinger appears above the related enrichment plot. (A) CTCF motif predictions on ChIP-seq data for WHIP, a helicase interacting protein. (B) JUN motif predictions on ChIP-seq data for p300, a histone acetyltransferase. (C) GABPA motif predictions on ChIP-seq data for CCNT2, a cyclin regulator of CDK9 kinase. (D) THAP11 motif predictions on ChIP-seq data for CHD2, a chromodomain helicase. [file 13059_2014_412_MOESM5_ESM.pdf]

**A**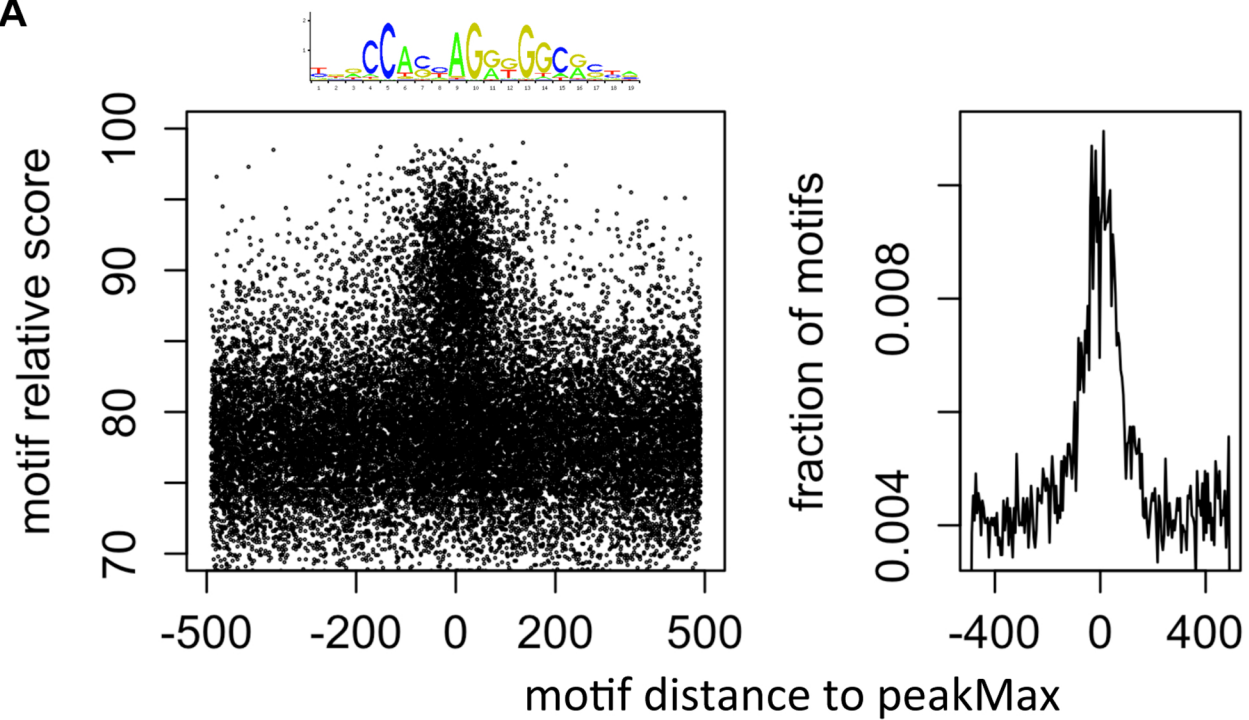**B**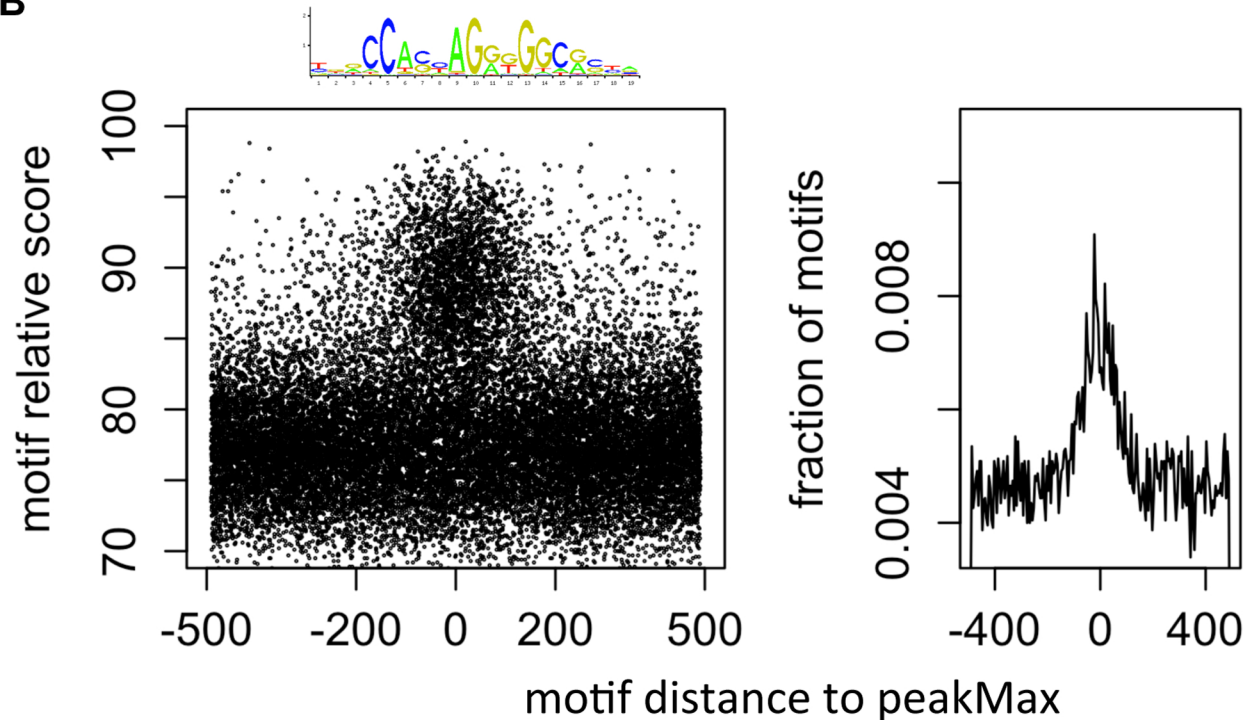

Supplement: Additional file 6: Figure S5. — Input and mock-IP control data are enriched for zinger motifs. The enrichment plots display the location of the top scoring CTCF motif for each peak relative to the peakMax (the peakMax is at 0) on the x-axis, while the score of the motif is plotted on the y-axis. The adjacent line plots display the fraction of CTCF motifs observed in 5 bp increments. The logo reflecting the binding specificity for CTCF appears above the related enrichment plot. (A) Input regions from the HUVEC cell line. (B) IgG rabbit mock-IP regions from GM12878 cells. [file 13059_2014_412_MOESM6_ESM.pdf]

**A**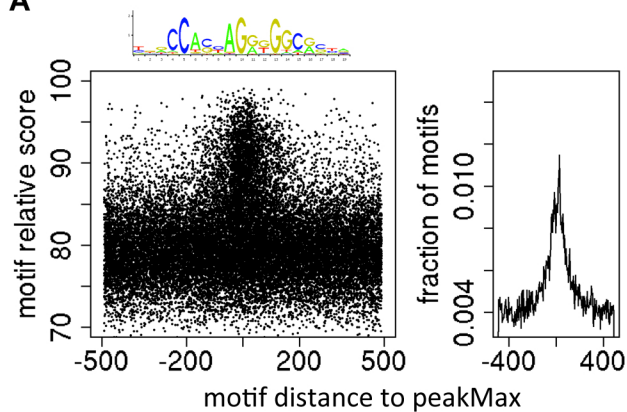**B**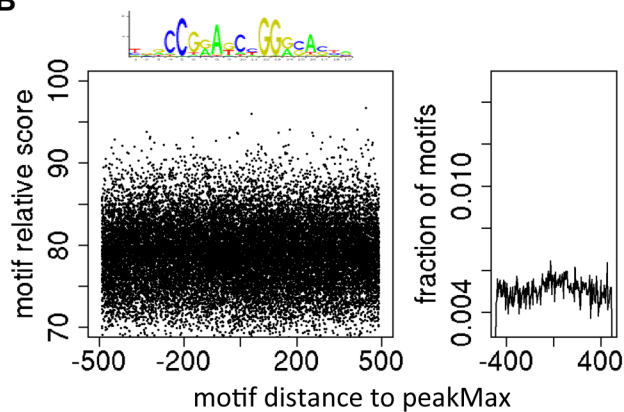**C**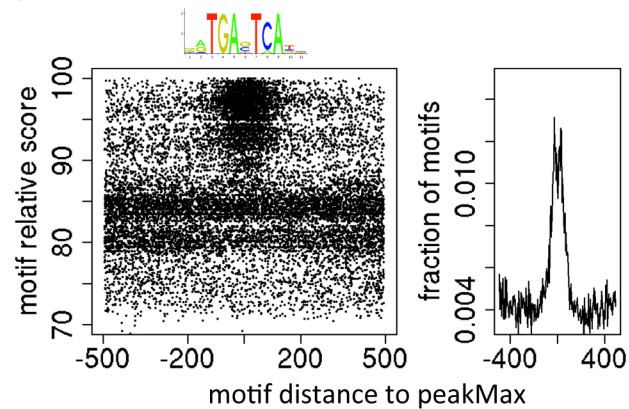**D**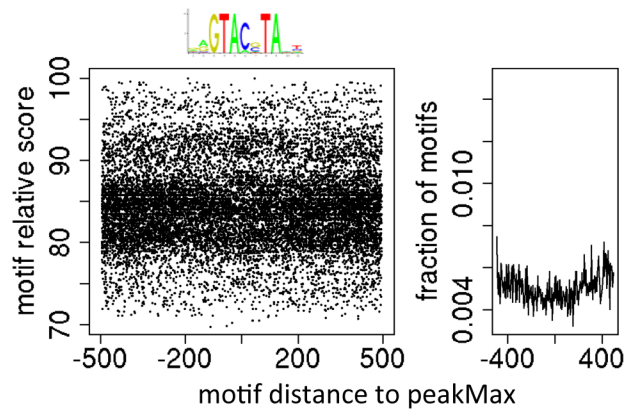

Supplement: Additional file 7: Figure S6. — Shuffled zinger PWMs are not enriched proximal to the peakMax. The enrichment plots display the location of the top scoring motif for each peak relative to the peakMax (the peakMax is at 0) on the x-axis, while the score of the motif is plotted on the y-axis. The adjacent line plots display the fraction of motifs observed in 5 bp increments. The logo reflecting the binding specificity for each zinger appears above the related enrichment plot. (A) Enrichment of CTCF motifs on the NRF1 (GM12878) dataset. (B) Enrichment of shuffled-CTCF motifs on the same NRF1 (GM12878) dataset. (C) Enrichment of JUN motifs on the TCF7L2 (Hct116) dataset. (D) Enrichment of a shuffled-JUN motif on the same TCF7L2 (Hct116) dataset. [file 13059_2014_412_MOESM7_ESM.pdf]

**A**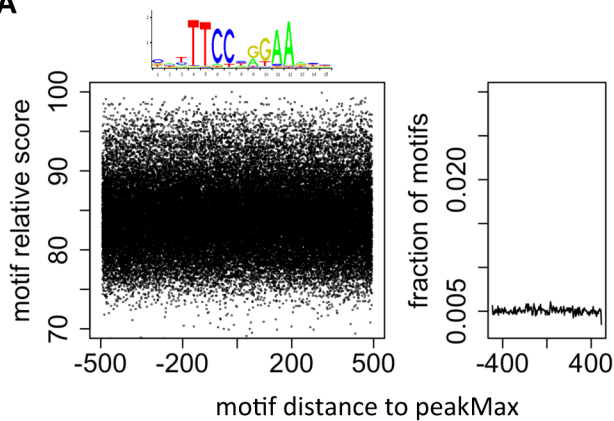**C**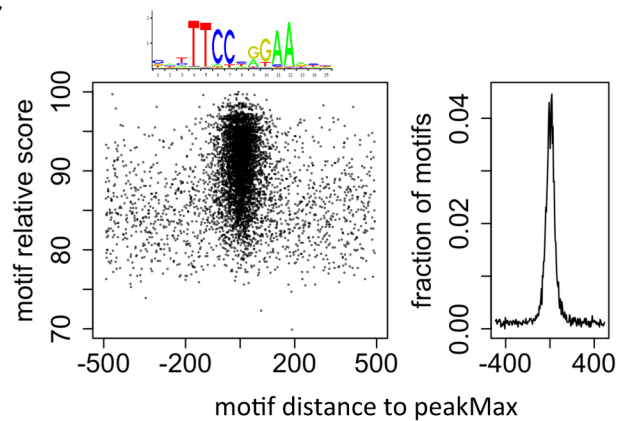**B**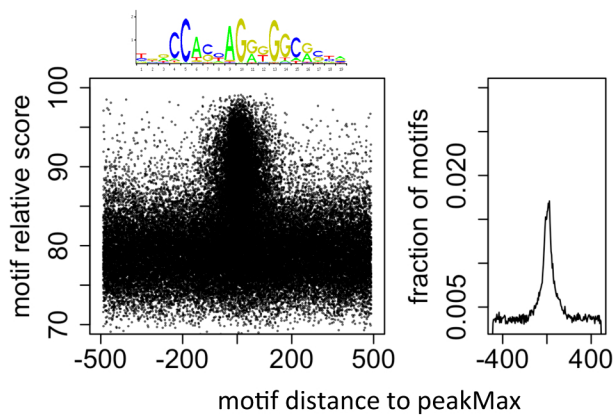**D**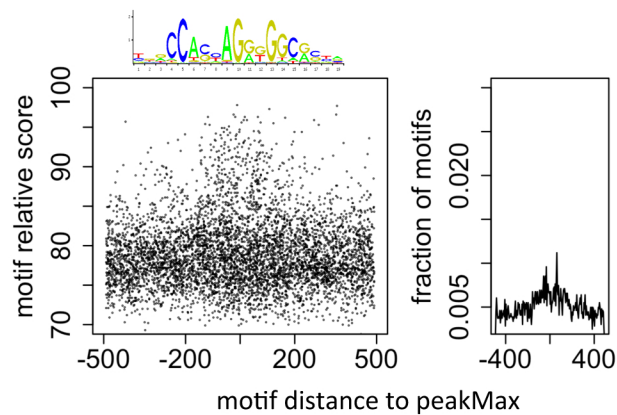

Supplement: Additional file 8: Figure S7. — Untreated STAT1 ChIP-seq data show strong zinger motif enrichment and not STAT1 motif enrichment. The enrichment plots display the location of the top scoring motif for each peak relative to the peakMax (the peakMax is at 0) on the x-axis, while the score of the motif is plotted on the y-axis. The adjacent line plots display the fraction of motifs observed in 5 bp increments. The logo reflecting the binding specificity for each zinger appears above the related enrichment plot. (A) STAT1 motif predictions on STAT1 ChIP-seq from untreated GM12878 cells. No STAT1 motif. (B) CTCF motif predictions on STAT1 ChIP-seq from untreated GM12878 cells. (C) STAT1 motif predictions on STAT1 ChIP-seq from IFNγ treated HeLa cells. STAT1 motif is present. (D) CTCF motif predictions on STAT1 ChIP-seq form IFNγ treated HeLa cells. [file 13059_2014_412_MOESM8_ESM.pdf]

**A**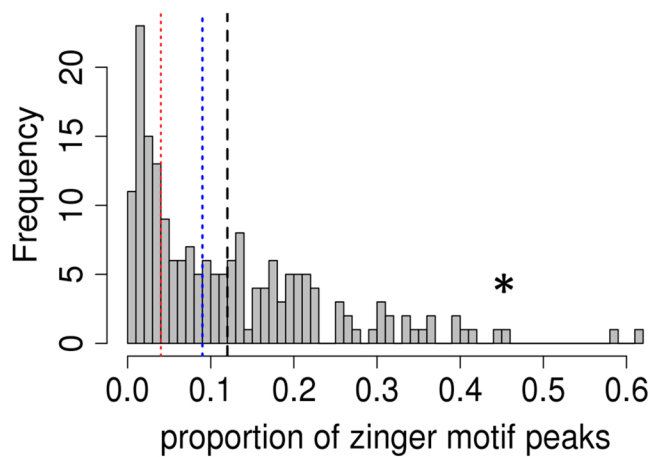**B**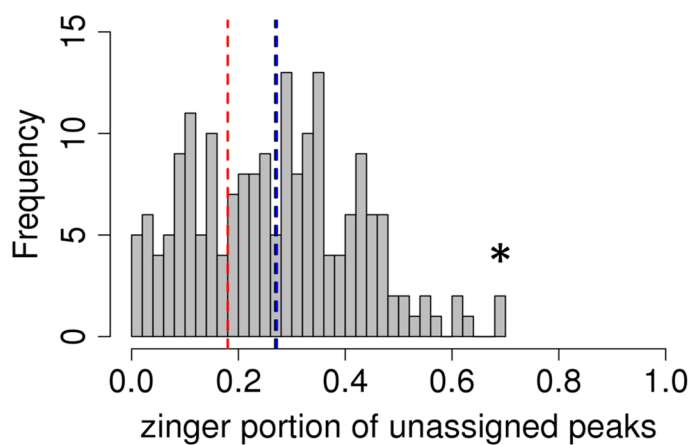**D**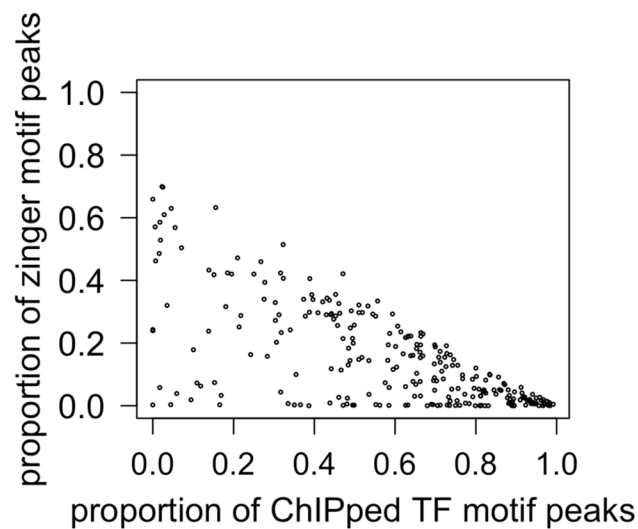**C**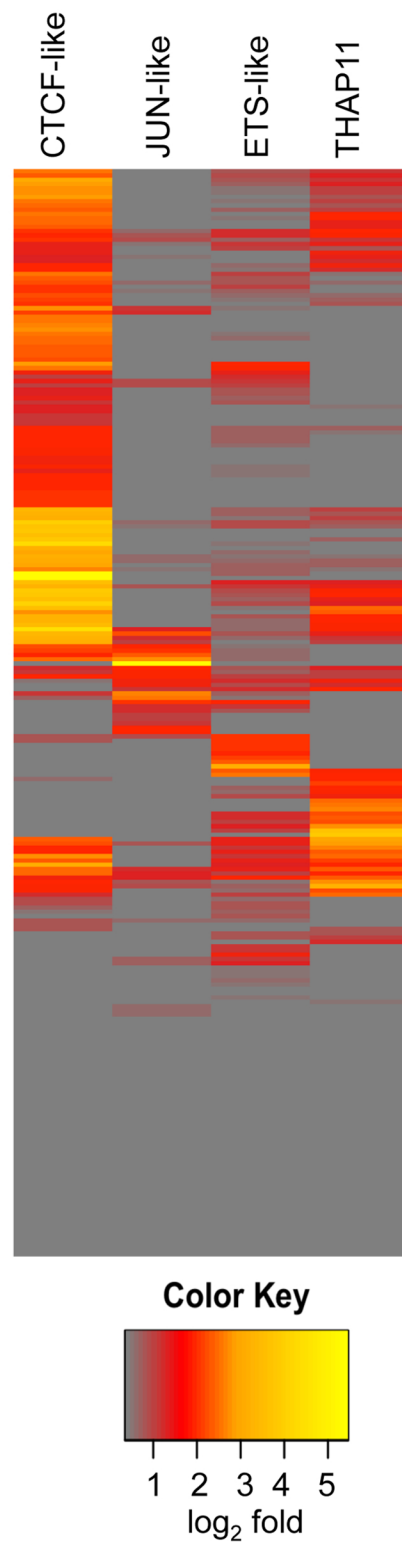

Supplement: Additional file 9: Figure S8. — The distribution of zinger motif content varies across ChIP-seq datasets. (A, B) For those datasets with at least a 1% zinger component, the histograms present the distribution of observed zinger motif peak content. The x-axis reports the proportion of zinger motif peaks within an analyzed dataset, and the y-axis the frequency of such observations. The black vertical dashed line represents the mean, the blue vertical dashed line represents the median, and the red vertical dashed line represents the point where two-thirds of the datasets are to the right of the line. The asterisk indicates the maximum zinger proportion, excluding outliers. (A) Analysis performed on entire ChIP-seq datasets. (B) Analysis on the set of peaks unaccounted for by the ChIPped TF motif. (C) A heatmap of the individual zingers’ motif peaks log2 fold enrichment in the set of peaks unaccounted for by the ChIPped TF and with a strong motif score (score 85 or greater). Fold enrichment less than 1.5 is grey. The rows are individual datasets, the columns are the zingers. (D) A scatterplot of the proportions of zinger motif peaks (y-axis) and ChIPped TF motif peaks (x-axis) in each dataset. [file 13059_2014_412_MOESM9_ESM.pdf]

**A**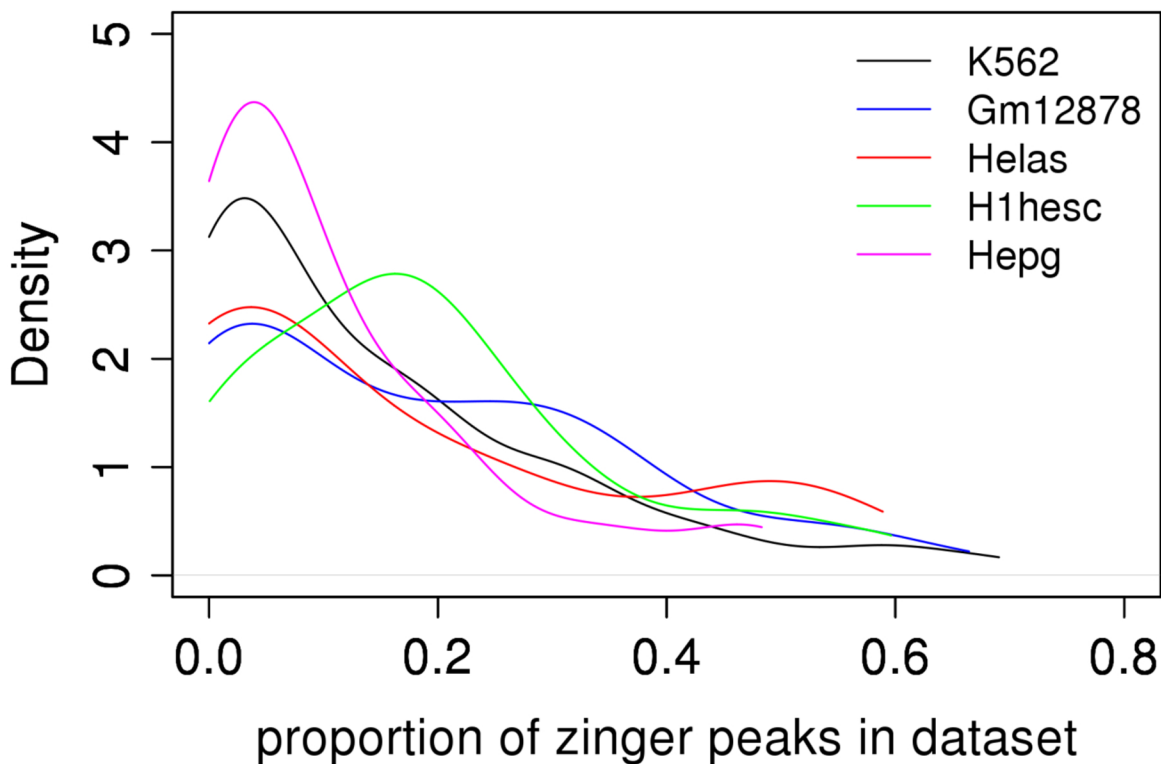**B**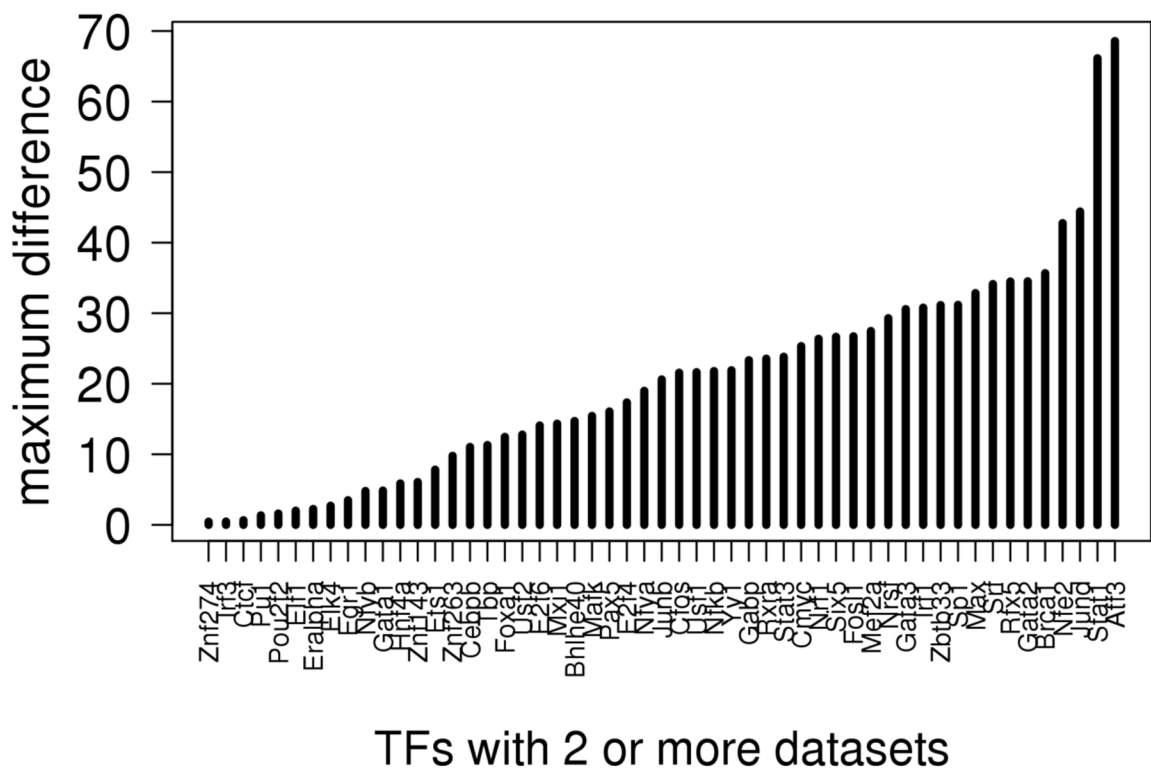

Supplement: Additional file 10: Figure S9. — The proportion of a dataset with zinger motifs is not dependent on cell-line nor the ChIPped TF. (A) The x-axis is the proportion of datasets composed of zinger motif peaks. The y-axis is a density value reflecting the fraction of datasets with zinger motifs. The five cell lines are K562 (black), GM12878 (blue), HeLa (red), H1-hESC (green), and HepG2 (magenta). There are no significant differences between the distributions per Wilcoxon test P values. (B) The TFs analyzed are listed on the horizontal access. The y-axis is the maximum difference of zinger proportions observed between two ChIP-seq datasets for the same TF. [file 13059_2014_412_MOESM10_ESM.pdf]
