# Supplementary material for: Non-targeted transcription factors motifs are a systemic component of ChIP-seq datasets
Source: Genome Biol. 2014 Jul 29;15(7):412. doi: 10.1186/s13059-014-0412-4 (PMC4165360; doi:10.1186/s13059-014-0412-4)
Supplement: Additional file 4: Text S1. — Additional observations regarding zinger motifs and zinger motif peaks. [file 13059_2014_412_MOESM4_ESM.pdf]

Supplemental data

Open chromatin data and non-TF ChIP-seq data have zinger enrichment

As stated in the main text we found that zinger motifs are enriched in DNaseI-Seq and Faire-Seq datasets. We extended the DNaseI-Seq and Faire-Seq regions from 150bp (on average) to 1001 bp to assess the proportion of regions within a dataset containing a zinger motif near the ~150bp region. After accounting for background rates of motif prediction, up to 67% of DNaseI-Seq regions contain a zinger, with a mean of 47% (median 48% with a median absolute deviation (MAD) of 6 pp (percent points)). The Faire-Seq regions had less zinger representation with a maximum of 32% of peaks and mean 13% (median 10% with a MAD of 7 pp).

As zinger motifs were enriched across numerous TF ChIP-seq datasets, and zinger motifs also have a strong presence in open chromatin regions, we wondered to what extent zinger motifs are present in any ChIP-seq based data. We assessed 31 datasets (18 proteins) for non-sequence specific proteins (histone modifiers, general TFs, elongation factors, co-activators *etc*.) and found zinger motifs enriched in half of these datasets. WHIP, a helicase interacting protein, CCNT2, a cyclin-T2 protein, CHD2, a helicase, and p300, a histone acetyl transferase, are examples of datasets displaying zinger motif enrichment proximal to the peakMax (Additional File 5 Figure S4).

Control data such as sheared DNA or mock immunoprecipitated DNA (mock-IP) for ChIP-seq is known to be enriched for open chromatin regions [1-3]. We assessed the enrichment of zinger motifs in the top ranking 70,000 peaks of 40 ChIP-seq controls, both Input and mock-IP. While all zingers were enriched in a subset of controls (GABPA in 26%, THAP11 in 16%, and JUN in 11%), only CTCF showed a strong and consistent pattern of enrichment (enriched in 66% of controls) (Additional File 6 Figure S5).

Extreme cases of datasets lacking the ChIPped TF's motif

There are extreme cases in which the ChIPped TF's binding motif occurs in less than 10% of the dataset and more than 30% of the dataset contains a zinger motif *e.g*. BRCA1 (HepG2), ZNF143 (GM12878, HeLa, K562), and unstimulated STAT1

(GM12878). The STAT1 (GM12878) data is notable. According to the literature, unstimulated STAT1 can act as a constitutive TF that may interact weakly with DNA or other proteins [4]. There were 49,261 peaks reported in the STAT1 unstimulated dataset, but the dataset was not enriched for weak sites of either the full or half canonical motif. At least 30% (after adjusting for background) of the peaks in the dataset are zinger motif peaks, primarily the CTCF motif (Additional file 8 Figure S7 A-B). In contrast, as a positive control, when STAT1 (HeLa) is stimulated with IFNγ, we observe clear enrichment for the STAT1 motif near the peakMax and little of the zinger motifs (Additional file 8 Figure S7 C-D).

Average background expectation of motif predictions

Determining the overall background expectation for the frequency of peaks with central motifs for either the ChIPped TF or the zingers can be assessed by considering the motif peak frequencies in the flanking regions of sequences, distal to the enrichment around the peakMax. The analysis was performed for all datasets displaying central enrichment for the assessed motif. For the ChIPped TF motif peaks, we estimate that, on average, 10% (median 7% with a median absolute deviation (MAD) of 9 percent points (pp)) of the cases arise by chance (~6% of the average ChIP-seq experiment). Likewise, 36% (median 40% with a MAD of 11 pp) of the enriched zinger motif peaks are expected to be present by chance (~7% of the average ChIP-seq experiment). Within the main text, each background expectation was determined specific to the datasets analyzed prior to reporting background corrected estimates of enrichment.

As indicated in the main text, we selected the subset of peaks with motifs that had a strong match to the PWM (motif score of 85 or greater). The background expectation using such high scoring motifs for the ChIPped TF dropped to a mean of 8% (median 5% with a MAD of 8 pp) of the predictions arising by chance. For the zinger motif peaks the mean dropped to 25% (median 21% with a MAD of 15 pp) of the predictions potentially arising by chance.

Zinger motif attributed peaks' proximity to genomic features

Using the zinger motif peaks, we assessed their proximity to promoter-related features such as the transcription start site (TSS), CpG islands, and conserved regions, in

addition to repeat sequence regions. We evaluated the proportion of peaks within 500bp, 1kb, 2kb, or 5kb of the TSS, or within 500bp for the other listed features, relative to the proportion of peaks more distant and counted those datasets meeting a significance threshold (Fisher's exact test p-values <0.001). For the majority of features, the number of datasets with the zinger motif peaks statistically further from the genomic feature than the ChIPped TF motif peaks was not striking. However, there were a few exceptions: 68% and 75% of datasets enriched for GABPA or THAP11 zinger motifs, respectively, displayed a tendency for the zinger motif peaks to be closer to the TSS (or CpG islands) than ChIPped TF motif peaks (Fisher test p-value <4e-05), while the reverse was true for JUN zinger motif enriched datasets (69% of datasets; Fisher test p-value <3e-05). When we examined GABPA and JUN ChIP-seq data we found that a greater proportion of GABPA ChIP-seq peaks are proximal to a TSS than are JUN ChIP-seq peaks (64% *versus* 17% relatively). Thus the zinger motif peaks show positional enrichment consistent with the enrichment observed for the ChIP-seq experiments specifically targeting the zinger TFs. This observation is supportive of the zinger motif peaks being *bona fide* targets of the zinger TFs.

Zinger motif peaks may be *bona fide* targets of the zinger TF

As indicated in the main text, we found good agreement between the zinger CTCF peaks with a strong motif score (score > 85) and CTCF ChIP-seq peaks from the same cell line (Figure 4A) – on average 75% of zinger CTCF peaks overlap CTCF ChIP-seq peaks (median 79%; MAD 15 pp). As control, we assessed those peaks with the CTCF motif distal to the peakMax (see Methods). On average, only 10% of the peakMax for the distal CTCF motifs were within 100bp of a CTCF ChIP-seq peakMax (median 9%; MAD 4 pp). The zinger CTCF motif peaks are significantly more likely (Wilcoxon one-tailed test p-values 5.5<e-50) to overlap CTCF ChIP-seq than distal-zinger CTCF peaks.

We evaluated JUN zinger motifs (Figure 4B) and GABPA zinger motifs (Figure 4C), in the same manner, using these two zinger motifs as proxy for the two families of motifs. Neither JUN nor GABPA ChIP-seq datasets and PWMs produce the same sharp delineation between high scoring peaks that are peakMax proximal compared to distal as occurs with CTCF (Figure 2A-C). We therefore expected a higher false positive rate and reduced agreement between the zinger motif peaks and the JUN or GABPA ChIP-seq datasets.

On average 38% of zinger JUN peaks are within 100bp of a JUN ChIP-seq peak within the same cell type (median 38%; MAD 17 pp), while only 6% of distal-zinger JUN peaks (distal to the peakMax) are in such agreement with JUN ChIP-seq peaks (median 4%; MAD 4 pp) (Figure 4B). The proportion of peaks with a JUN zinger motif proximal to the peakMax that agrees with a JUN ChIP-seq peak is significantly more than the distal-zinger JUN peaks (Wilcoxon one-tailed test p-value 3.4<e-20).

Although GABPA zinger peaks have higher background motif rates compared to CTCF or JUN, the overlap of GABPA zinger motif containing peaks with the GABPA ChIP-seq peaks is still significantly higher than the overlap observed for the distal-zinger motif containing regions (Wilcoxon one-tailed test p-value <1.1e-30) (Figure 4C). On average, 28% of GABPA zinger peaks in a dataset agree with GABPA ChIP-seq peaks from the same cell type (median 27%; MAD 13 pp), while there is only 8% average agreement for the non-zinger peaks (median 5%; MAD 5 pp).

In all datasets the zinger motif peak proportion of agreement with a ChIP-seq dataset was always greater than the distal-zinger proportion of agreement.

References

1. Dohm JC, Lottaz C, Borodina T, Himmelbauer H: **Substantial biases in ultra-short read data sets from high-throughput DNA sequencing.** *Nucleic Acids Res* 2008, **36:**e105.
2. Auerbach RK, Euskirchen G, Rozowsky J, Lamarre-Vincent N, Moqtaderi Z, Lefrancois P, Struhl K, Gerstein M, Snyder M: **Mapping accessible chromatin regions using Sono-Seq.** *Proc Natl Acad Sci U S A* 2009, **106:**14926-14931.
3. Teytelman L, Ozaydin B, Zill O, Lefrancois P, Snyder M, Rine J, Eisen MB: **Impact of chromatin structures on DNA processing for genomic analyses.** *PLoS ONE* 2009, **4:**e6700.
4. Yang J, Stark GR: **Roles of unphosphorylated STATs in signaling.** *Cell Res* 2008, **18:**443-451.
