# Supplementary material for: Non-targeted transcription factors motifs are a systemic component of ChIP-seq datasets
Source: Genome Biol. 2014 Jul 29;15(7):412. doi: 10.1186/s13059-014-0412-4 (PMC4165360; doi:10.1186/s13059-014-0412-4)
Supplement: Additional file 11: Figure S10. — Zinger motifs and zinger motif peaks are not strongly correlated. (A) A heatmap of significance for inter-dependence between pairs of zinger motifs in zinger motif peaks. Positive associations with a significant Fisher exact P value (P value <0.001) are yellow, negative associations with a significant Fisher exact P value are red, and non-significant P values are grey. The color density reflects P value significance, with the densest colors being P values closest to 0. The columns are individual datasets; the rows are the six possible zinger pairs. (B) A correlation matrix presenting both Spearman’s rank (lower diagonal) and Pearson (upper diagonal) correlation coefficients for the pairwise association of zinger motif peak enrichment within the same ChIP-seq datasets. [file 13059_2014_412_MOESM11_ESM.pdf]

A

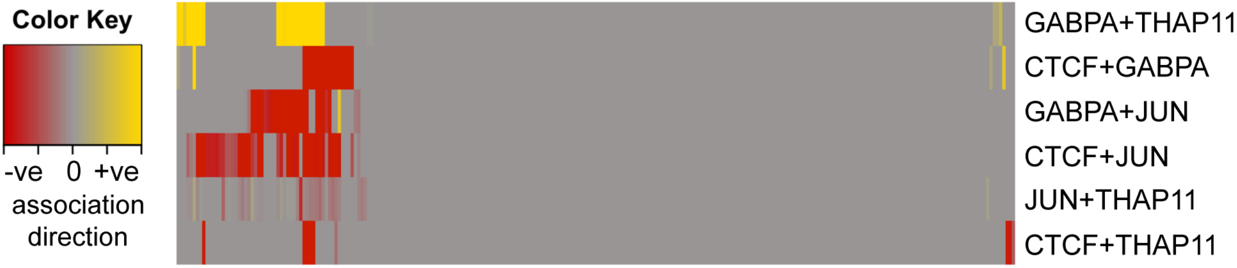

B

	CTCF-like	ETS-like	JUN-like	THAP11
CTCF-like	1	0.1346	0.0057	0.1263
ETS-like	0.3003	1	0.0123	0.2088
JUN-like	0.2937	0.3157	1	-0.0233
THAP11	0.3584	0.3803	0.1563	1

Pearson correlation coefficients

Spearman's rank correlation coefficients
