# Supplementary material for: Non-targeted transcription factors motifs are a systemic component of ChIP-seq datasets
Source: Genome Biol. 2014 Jul 29;15(7):412. doi: 10.1186/s13059-014-0412-4 (PMC4165360; doi:10.1186/s13059-014-0412-4)
Supplement: Additional file 12: Dataset S1. — Genomic coordinates for zinger neighborhoods (tab delimited). [file 13059_2014_412_MOESM12_ESM.rtf]

#chrom	left.coord	right.coord	number.of.TFschr12	53845528	53845908	40chr3	53381486	53381763	35chr11	62358908	62359214	36chr2	3622664	3622975	37chr3	52029768	52030047	30chr17	41561003	41561442	41chr5	40835203	40835526	34chr15	35261865	35262123	35chr20	31989260	31989521	39chr13	41345243	41345485	34chr17	29233106	29233436	34chr20	5093615	5093937	32chr21	46359708	46359983	35chr13	52768461	52768703	31chr1	42800903	42801204	38chr22	19419856	19420129	35chr21	26979706	26979937	31chr3	196014449	196014803	34chr5	133968371	133968645	31chr6	126277751	126278033	34chr9	130213556	130213810	31chr1	38455660	38455919	35chr2	232650937	232651262	34chr1	114447571	114448007	32chr17	8286418	8286805	32chr20	37063831	37064130	33chr2	197664282	197664536	34chr22	22337070	22337317	38chr4	71553851	71554280	36chr7	75920859	75921264	34chr10	43278074	43278365	31chr1	163291485	163291798	32chr11	67195749	67196015	30chr1	28655380	28655627	35chr13	113862865	113863157	35chr20	16710483	16710797	32chr3	142297473	142297789	40chr1	26324550	26324942	35chr19	11039299	11039534	31chr2	237994318	237994633	33chr7	141437980	141438295	28chr8	11141767	11142077	29chr11	62554677	62555001	34chr16	740296	740531	30chr18	47013495	47013852	30chr21	37692301	37692658	32chr6	170151558	170151901	35chr6	41755233	41755508	25chr7	32529844	32530170	29chr10	74927716	74928000	29chr11	47270275	47270608	31chr11	62420713	62420975	33chr1	227922905	227923139	33chr16	29816714	29817037	29chr16	75498455	75498863	32chr16	89626918	89627228	26chr17	49230704	49231007	32chr2	55496254	55496578	32chr5	138897560	138897859	31chr6	142468150	142468408	34chr7	148823274	148823656	31chr9	134000813	134001087	29chr9	140317552	140317819	37chr11	102962793	102963101	28chr1	222817392	222817702	33chr1	44678916	44679201	30chr16	87812699	87813005	31chr20	37075167	37075406	39chr20	57226105	57226364	32chr2	70369785	70370188	28chr3	10362672	10362951	36chr3	196295325	196295599	30chr4	13629313	13629614	35chr6	34855699	34855993	29chr7	127983788	127984084	30chr7	99679257	99679630	37chr9	140082976	140083235	30chr1	1310521	1310938	31chr1	151162559	151162788	34chr12	34175193	34175459	34chr12	56497943	56498378	25chr1	26496246	26496498	32chr1	29063052	29063430	36chr15	34517036	34517295	29chr15	38746217	38746479	32chr15	51200627	51200957	32chr15	76196085	76196388	30chr15	90437124	90437424	30chr16	2021816	2022087	33chr17	38083778	38084090	35chr20	1099067	1099325	28chr20	34542248	34542605	31chr2	178976919	178977288	31chr2	233415194	233415503	34chr2	24299168	24299413	29chr2	3605815	3606204	29chr4	74124754	74125060	28chr5	34915513	34915896	32chr6	30685180	30685560	28chr7	91509881	91510222	32chr1	110950232	110950545	31chr11	2421436	2421901	33chr11	65266393	65266607	30chr12	860366	860688	31chr15	34394072	34394393	30chr15	40401117	40401407	29chr17	42092248	42092492	29chr19	56165076	56165339	28chr20	3140369	3140660	34chr2	10829988	10830208	27chr22	39077808	39078099	33chr2	242041663	242041899	27chr2	74681925	74682261	30chr2	74710035	74710635	32chr3	149470169	149470437	33chr5	140998576	140998904	34chr5	37379229	37379516	34chr6	44280920	44281206	28chr7	134001619	134001964	32chrX	21857522	21857791	30chr10	75012230	75012571	33chr1	151138367	151138626	31chr11	64851428	64851738	25chr11	65655837	65656171	34chr12	38710349	38710584	29chr1	24018097	24018416	31chr1	36554330	36554611	31chr1	39456739	39457056	29chr16	1755930	1756236	31chr17	36981472	36981739	31chr2	74756696	74757112	28chr2	97304023	97304231	35chr5	102455640	102456000	27chr6	41747653	41747897	29chr7	66385899	66386232	33chr8	143751243	143751520	31chr8	33330560	33330873	37chr11	47429583	47430085	28chr11	47600404	47600674	29chr1	153643473	153643795	26chr1	156186306	156186526	31chr11	64885048	64885468	30chr11	6704414	6704899	28chr12	109490212	109490423	30chr14	39639428	39639746	31chr16	31085555	31085815	28chr16	48278058	48278389	33chr18	19192136	19192454	29chr19	1438207	1438498	32chr2	234160096	234160396	32chr3	156892632	156892967	34chr3	183602554	183602851	30chr5	21459404	21459720	31chr7	75943645	75943898	30chr9	130879960	130880206	32chr11	111749709	111750092	28chr1	114472021	114472275	27chr11	77348717	77349007	27chr12	57632854	57633140	30chr15	65809943	65810266	30chr16	70380635	70380909	29chr17	73008567	73008945	28chr18	33552466	33552705	28chr19	4400484	4400806	33chr19	47103933	47104369	27chr19	50083716	50084016	26chr19	58789940	58790444	25chr2	85152957	85153244	27chr3	128399530	128399885	30chr3	15140525	15140833	27chr3	156877899	156878201	32chr5	132202122	132202448	31chr5	134094252	134094545	32chr6	143771762	143771980	31chr7	1543902	1544291	31chr7	23221407	23221713	27chr9	139258137	139258443	33chr1	114354851	114355290	31chr11	17099129	17099475	26chr11	59578104	59578373	29chr16	2653213	2653499	33chr16	28857568	28857864	26chr16	30886525	30886847	26chr1	93811264	93811571	30chr19	56146192	56146473	29chr20	62289528	62289840	30chr2	122288280	122288795	27chr22	36520012	36520314	28chr22	38082194	38082522	30chr22	39715520	39715854	33chr22	50683166	50683597	27chr2	28615444	28615863	30chr3	119217152	119217470	26chr5	74062835	74063111	27chr6	108582277	108582721	26chr6	30640709	30641017	27chr6	31926757	31927109	30chr7	73668621	73668942	29chr7	89975842	89976183	30chr9	131534142	131534414	31chr9	79186627	79186843	34chr9	97488857	97489135	28chr10	1034187	1034474	28chr10	27792989	27793308	28chr10	6130669	6130956	29chr10	80828479	80828791	27chr10	88699175	88699474	29chr1	155904091	155904472	25chr15	67546794	67547167	29chr16	21610674	21610965	29chr16	2732313	2732581	34chr17	55927342	55927612	27chr2	231577353	231577629	28chr22	39925205	39925481	29chr3	101280515	101280841	26chr3	58291812	58292074	31chr5	80597248	80597527	27chr6	144416662	144416965	25chr6	25992714	25993035	30chr6	28192848	28193173	30chr6	34393737	34394062	23chr8	145550369	145550724	28chr8	37707336	37707603	27chr9	35103024	35103332	28chr1	109642633	109642989	29chr11	34126916	34127243	24chr11	57424924	57425312	28chr11	64863512	64863724	29chr1	179851737	179852069	26chr11	82782766	82783171	28chr12	118813975	118814332	28chr12	56617863	56618257	27chr13	31191789	31192076	28chr13	45563496	45563764	27chr14	104387770	104388045	27chr15	100105985	100106305	31chr15	56538250	56538505	31chr17	16120377	16120583	27chr17	26662397	26662763	30chr20	2451371	2451672	30chr20	35233867	35234154	26chr2	118572007	118572323	31chr21	30365100	30365546	26chr21	47706065	47706298	27chr2	24149889	24150151	32chr22	43583543	43583887	27chr2	74699685	74699969	28chr3	10028404	10028653	24chr3	10067909	10068246	26chr4	123844051	123844356	28chr5	137688102	137688350	33chr5	41925147	41925498	27chr6	31515172	31515436	26chr6	42185507	42185724	25chr7	1609593	1609829	32chr7	44925100	44925457	29chr7	96746795	96747070	29chr8	125551197	125551511	23chr8	54934849	54935136	30chr9	127624096	127624380	26chr9	33025035	33025392	32chr9	37800621	37800822	28chr10	75490121	75490413	26chr10	88159607	88159934	25chr10	95462259	95462534	30chr11	33182894	33183288	31chr1	19536726	19536987	21chr1	203830517	203830907	28chr13	111805801	111806136	24chr14	105282317	105282586	29chr14	21945118	21945451	28chr14	55518149	55518522	33chr14	69658059	69658320	28chr14	75348439	75348683	24chr1	53704106	53704344	29chr15	63340233	63340729	31chr16	87799472	87799736	25chr17	17991090	17991300	28chr17	43212731	43212983	30chr17	56429410	56429762	27chr17	80023628	80023839	27chr19	19302848	19303096	26chr1	94344614	94344925	26chr20	36661666	36661981	25chr20	49545968	49546190	29chr20	61569129	61569410	25chr2	101618535	101618889	24chr2	201753911	201754399	30chr3	47866313	47866599	29chr3	50264879	50265196	27chr4	4249879	4250132	26chr5	153418340	153418640	30chr5	36151927	36152297	29chr6	28367420	28367716	26chr6	90529379	90529718	24chr7	102985072	102985381	26chr7	138818328	138818598	29chr7	35734620	35734927	24chr7	6144130	6144439	29chr7	75831081	75831289	29chr8	110346272	110346529	27chr8	74884402	74884832	25chr9	127177628	127177946	27chr9	139838962	139839447	32chr1	10003257	10003658	24chr11	10772568	10772946	28chr11	3078525	3078845	26chr11	46958116	46958372	29chr1	149982561	149982808	25chr1	155278253	155278661	23chr11	62606943	62607258	25chr1	1655689	1655968	25chr11	86013097	86013413	30chr11	8710337	8710694	27chr12	31902097	31902469	25chr1	234908109	234908483	26chr12	62860402	62860690	26chr14	77428121	77428437	26chr15	57998680	57998973	26chr16	67850497	67850738	28chr17	26684454	26684765	26chr17	80606222	80606468	26chr19	12807303	12807617	27chr19	16683273	16683544	24chr19	50879510	50879721	28chr2	114514111	114514461	26chr3	142720155	142720485	29chr5	140027205	140027542	28chr5	145562104	145562504	28chr6	170893598	170893885	27chr6	28104485	28104745	27chr6	49430930	49431181	25chr7	105172503	105172758	20chr7	156742213	156742557	26chr7	66146906	66147198	29chr9	127703288	127703574	26chr9	37592437	37592737	26chrX	119077620	119077840	28chr11	6502545	6502877	28chr1	173683880	173684148	26chr1	205601024	205601314	31chr12	109531160	109531520	28chr12	120105415	120105940	26chr12	58176363	58176633	28chr1	27070622	27070876	28chr14	39644153	39644516	27chr14	55737840	55738155	27chr15	63796630	63796923	27chr15	83419117	83419412	21chr16	1470674	1470929	25chr16	71496035	71496293	26chr17	40925338	40925526	22chr17	48474768	48475019	31chr17	62915515	62915832	29chr17	79670244	79670555	27chr19	17325997	17326335	27chr19	34850022	34850586	27chr19	56186321	56186646	29chr19	7694424	7694751	27chr19	9546129	9546400	24chr2	162164793	162165147	29chr2	219260740	219261100	32chr22	24951768	24952046	22chr2	240964708	240964956	26chr2	27294364	27294751	26chr3	101292772	101293083	27chr3	125094127	125094379	25chr4	39367885	39368138	25chr5	53813345	53813588	31chr5	77590436	77590734	29chr6	111195791	111196088	30chr6	134274153	134274407	24chr6	29932677	29932930	25chr7	43768984	43769239	25chr7	66057242	66057565	23chr8	141467760	141468015	27chr8	145133333	145133701	25chr9	136283056	136283346	25chr9	140473264	140473564	30chr1	110527142	110527517	22chr11	116643605	116643844	27chr11	31391244	31391469	28chr1	1590424	1590666	25chr1	160312940	160313259	26chr11	65382662	65382989	25chr1	204485358	204485661	24chr12	124086485	124086809	24chr12	51441599	51442111	28chr12	7079741	7080040	29chr12	95611222	95611643	27chr12	98910105	98910450	27chr13	46626760	46627064	26chr13	48669126	48669448	26chr1	40723539	40723890	31chr1	45965787	45966096	22chr15	44955763	44956116	24chr15	64388034	64388295	26chr16	1401780	1402014	29chr16	31153718	31154013	28chr1	6614488	6614793	28chr16	69599656	69599929	28chr1	6761795	6762128	28chr16	87984492	87984845	24chr17	10600731	10601048	24chr17	45144741	45145033	28chr17	73901014	73901327	26chr18	54318334	54318626	23chr19	12721468	12721738	27chr19	19626861	19627132	23chr19	38826247	38826478	27chr19	5690086	5690412	28chr20	30200716	30200930	26chr21	27107127	27107397	23chr2	20850793	20851139	26chr2	32390764	32391030	23chr2	74153825	74154146	24chr3	45730438	45730808	30chr4	190861811	190862114	26chr4	8442270	8442578	23chr5	81147707	81147953	26chr6	15691527	15691792	30chr6	52859973	52860418	28chr7	102937715	102938019	23chr7	128116636	128116928	23chr7	134855391	134855731	28chr8	144363725	144363993	23chr8	17780050	17780371	24chr9	114393380	114393663	32chr9	123555589	123555952	26chr9	136202965	136203229	26chr9	139294561	139294825	28chr9	139622552	139622822	29chr9	140100020	140100279	23chr9	4679375	4679737	27chr9	88969184	88969512	27chr10	122610690	122611070	31chr10	18940384	18940704	24chr10	96122562	96122871	25chr1	109806251	109806546	24chr1	145507358	145507690	24chr1	150551902	150552231	26chr1	151227041	151227320	24chr1	155145649	155145901	25chr1	156629836	156630293	27chr1	161509955	161510248	32chr11	63741872	63742219	25chr1	193090929	193091242	27chr11	9385851	9386180	24chr12	120907448	120907726	27chr12	122232112	122232440	30chr12	123237000	123237428	23chr12	45342549	45342774	29chr12	69080595	69080854	24chr14	24657969	24658220	31chr14	67826475	67826977	26chr15	55489008	55489350	23chr15	83735839	83736163	19chr16	23607517	23607789	22chr16	23652593	23652858	27chr16	4475440	4475922	29chr16	57496422	57496685	23chr16	66907040	66907456	23chr16	790844	791166	25chr16	81069205	81069433	27chr16	89787252	89787572	28chr17	25680721	25681034	26chr17	33288350	33288683	23chr17	34135963	34136451	22chr17	3749496	3749740	26chr18	33077744	33078110	30chr19	4182407	4182761	25chr19	42829344	42829773	22chr20	33264883	33265190	25chr20	35917908	35918248	26chr2	10575091	10575339	26chr22	20104642	20104996	30chr2	45878091	45878374	25chr3	150264304	150264630	30chr3	16306321	16306689	24chr3	9005022	9005353	24chr4	109541575	109541907	23chr5	110427865	110428172	28chr5	122758998	122759376	27chr5	37371104	37371380	26chr6	149867095	149867471	24chr6	42531459	42531996	25chr7	135347052	135347345	27chr7	150755010	150755361	26chr7	30634313	30634556	29chr7	44240428	44240754	24chr7	75807483	75807742	23chr7	92076572	92076866	26chr8	33342506	33342797	23chr9	108006538	108006754	26chr9	116037686	116038059	27chr9	131084650	131084965	26chr9	132404365	132404670	27chr9	132597405	132597749	26chr10	105156216	105156560	23chr10	13628790	13629070	25chr10	75571390	75571717	26chr1	114889201	114889467	26chr11	30344395	30344727	30chr1	1406954	1407210	20chr11	43902183	43902435	26chr11	65149642	65150112	26chr11	9587554	9587914	24chr12	104350884	104351208	26chr12	122710474	122710769	25chr1	228353291	228353844	27chr12	49110361	49110758	25chr12	53574246	53574552	29chr12	75905270	75905588	27chr12	94070978	94071289	24chr13	77566027	77566377	23chr14	61447705	61448025	26chr15	41186401	41186726	26chr15	64679876	64680082	28chr1	59012282	59012623	27chr16	20817677	20817962	25chr16	2301508	2301808	29chr16	68344665	68344998	26chr16	71879642	71879921	28chr16	89307288	89307642	31chr17	30771272	30771579	27chr18	11851229	11851465	26chr18	11947238	11947523	24chr18	48556375	48556727	23chr19	58816500	58816845	22chr19	58898427	58898728	22chr19	9695082	9695339	22chr20	32398845	32399122	25chr20	47835703	47836078	20chr2	191184439	191184836	27chr2	203130304	203130577	21chr22	32145988	32146295	26chr22	46731488	46731793	28chr2	70120938	70121175	22chr3	184080987	184081319	27chr3	64009037	64009356	23chr5	175875120	175875497	25chr5	60458172	60458450	26chr6	107349228	107349531	24chr6	28109471	28109740	24chr6	42946824	42947105	19chr7	144533036	144533310	29chr7	35840458	35840795	25chr7	45151207	45151553	25chr7	6616936	6617199	27chr7	72722723	72723003	28chr7	75677219	75677504	24chr8	56685860	56686204	24chrX	70288237	70288512	26chr10	94351318	94351679	25chr1	109618491	109618803	22chr1	151371855	151372082	21chr11	75110371	75110699	24chr12	110486281	110486538	29chr12	31881831	31882250	23chr1	234509007	234509331	22chr1	235324415	235324753	25chr1	32573421	32573742	24chr13	53024648	53024961	25chr14	105956968	105957510	27chr14	70233640	70233967	26chr14	77422998	77423320	24chr16	53088730	53089010	25chr16	67260812	67261107	25chr16	89283911	89284199	22chr17	40169321	40169668	22chr17	47647320	47647684	24chr17	65821370	65821708	28chr17	72199634	72199867	31chr18	3297395	3297662	24chr18	55297427	55297663	30chr18	67872920	67873400	29chr19	38397583	38397951	23chr19	55791369	55791676	25chr1	9687053	9687388	26chr20	49407278	49407503	28chr20	5986581	5986906	25chr21	30374976	30375284	24chr2	217363366	217363705	25chr22	38004303	38004857	27chr22	47158303	47158566	24chr2	3383340	3383847	20chr2	85788561	85788838	29chr2	9695770	9696135	25chr3	11888241	11888516	25chr3	131221681	131221949	21chr3	5163757	5164032	23chr5	115177142	115177450	23chr5	140090674	140090952	29chr6	3069028	3069383	27chr6	32806456	32806788	28chr8	117778561	117778872	24chr8	124253462	124253799	27chr8	145980880	145981136	25chr10	103815870	103816195	25chr1	150459639	150459889	29chr11	66112456	66112719	24chr1	172413115	172413410	28chr11	73882081	73882464	25chr12	107167799	107168192	26chr12	121124467	121124850	27chr12	12764550	12764870	23chr1	218458461	218458735	23chr1	226595579	226595928	27chr12	62653927	62654255	28chr13	103497968	103498300	26chr13	25875528	25875851	27chr14	105512059	105512380	27chr15	42783271	42783579	23chr15	45879371	45879677	24chr15	75917976	75918271	24chr16	31519636	31519885	24chr16	67515019	67515341	26chr16	89766568	89766859	22chr17	1359390	1359708	26chr17	27077110	27077383	22chr17	33905483	33905786	22chr19	10230472	10230724	25chr19	36618708	36618959	27chr19	56092235	56092482	25chr1	95699548	95699898	28chr19	57751813	57752117	22chr19	57901038	57901433	23chr19	9879277	9879549	24chr20	2644738	2645043	20chr20	277868	278171	22chr20	34824241	34824493	24chr20	5591521	5591808	24chr2	113341820	113342173	25chr21	45759199	45759448	20chr2	170430268	170430522	22chr22	36925116	36925426	23chr2	61244196	61244516	22chr2	66662060	66662361	24chr2	96971164	96971468	24chr3	127842663	127842905	25chr3	128369581	128369886	22chr3	128880030	128880414	28chr3	141030463	141030767	24chr3	179322277	179322609	24chr3	3221301	3221535	28chr3	46530221	46530501	24chr4	103940693	103941028	25chr4	1857896	1858168	22chr5	125936475	125936720	23chr5	139936907	139937208	25chr5	171615683	171615921	22chr5	172571327	172571597	26chr5	68389576	68389996	26chr6	13615340	13615654	25chr6	17707080	17707343	25chr6	28219960	28220186	28chr6	36853494	36853749	25chr6	693029	693207	23chr7	123389066	123389384	28chr7	134331320	134331703	23chr7	66119299	66119608	25chr7	91763853	91764154	26chr7	99149604	99149904	25chr7	99647218	99647599	25chr8	119124008	119124318	22chr9	123964072	123964580	25chrX	100645806	100646090	26chrX	70752634	70752907	27chr10	32734921	32735238	24chr10	93683359	93683649	23chr1	110036462	110036832	22chr11	18127482	18127857	23chr11	18548324	18548627	27chr11	43380282	43380643	24chr11	67273500	67273850	23chr11	8704093	8704435	21chr11	93517273	93517539	21chr12	106751345	106751672	23chr12	111834782	111835065	27chr12	124118156	124118419	28chr12	42719778	42720105	21chr12	4386171	4386419	21chr12	49245861	49246165	27chr12	57881639	57881927	23chr1	26758653	26758942	22chr12	8234626	8234940	25chr12	95397387	95397614	25chr12	98909176	98909481	21chr13	37573375	37573656	25chr14	23451811	23452068	24chr14	73493718	73494115	21chr16	21311810	21312188	24chr16	2390840	2391113	27chr16	25118339	25118661	25chr16	30825488	30825911	23chr16	31191214	31191560	23chr16	4664776	4665111	22chr17	13972660	13972941	21chr17	1945172	1945489	23chr17	2239642	2239959	21chr17	25708080	25708332	26chr17	40086532	40086868	21chr17	41150233	41150420	22chr17	41277411	41277750	23chr17	48785182	48785467	22chr17	78549185	78549454	26chr19	41140450	41140681	26chr1	94374923	94375373	26chr19	47291718	47291992	23chr19	51925220	51925491	25chr19	984188	984422	23chr19	9938399	9938693	26chr20	17949368	17949747	30chr20	30539688	30540086	23chr20	46130421	46130739	28chr2	11884247	11884504	22chr2	160568763	160569116	24chr2	21022687	21022965	22chr2	220110049	220110358	20chr22	26908275	26908628	23chr22	39898173	39898427	24chr2	43653619	43653904	25chr2	73461308	73461557	26chr3	101395888	101396183	25chr3	13493263	13493576	24chr3	158362161	158362477	25chr3	183852985	183853245	25chr3	3168468	3168781	22chr3	32822961	32823282	28chr3	50297449	50297716	28chr4	130014495	130014866	21chr4	187112468	187112804	26chr4	48832902	48833268	24chr4	56814800	56815160	26chr5	1633992	1634289	26chr5	43515140	43515434	26chr5	64858865	64859160	25chr6	119215139	119215393	23chr6	70506677	70507168	24chr7	102987990	102988263	23chr7	150869689	150869935	25chr7	64838523	64838908	23chr7	65958416	65958736	22chr8	126103906	126104148	25chr8	144373388	144373694	28chr8	27631707	27632146	24chr8	28479755	28480015	26chr8	48873276	48873538	24chr8	67976382	67976705	24chr9	19049093	19049439	22chr9	2015050	2015443	21chr9	98638052	98638397	26chrX	153763059	153763290	24chr10	124713638	124713982	26chr10	38265453	38265820	28chr10	73808359	73808643	23chr10	74080513	74080783	22chr10	99496742	99497062	22chr11	118230138	118230436	19chr11	36531676	36531998	21chr1	145096269	145096545	23chr1	145208885	145209171	21chr1	156710742	156711154	23chr1	167684716	167684908	28chr11	94706621	94706887	24chr1	21985652	21986017	27chr1	224301659	224301919	24chr12	25539041	25539503	25chr1	231114610	231114955	26chr1	24151772	24151992	23chr1	246729473	246729808	21chr12	88535800	88536159	23chr1	28879271	28879570	21chr12	92539514	92539807	24chr12	96252532	96252854	19chr13	29233060	29233371	23chr13	45915222	45915499	20chr14	73524922	73525303	24chr14	74226746	74227237	27chr15	101835398	101835730	25chr15	66790031	66790329	25chr15	74753386	74753756	26chr15	85259236	85259500	23chr15	89010522	89010891	21chr15	93352368	93352726	29chr16	23568527	23569019	25chr16	2771010	2771311	25chr16	48644009	48644310	20chr16	56485120	56485566	23chr16	67313265	67313511	25chr16	69345086	69345326	21chr16	717933	718129	23chr16	81110716	81111008	22chr16	8891444	8891790	21chr17	35969365	35969774	24chr17	42147903	42148162	28chr1	89357083	89357437	26chr19	18499276	18499562	24chr19	18682374	18682701	25chr19	32896355	32896628	20chr19	39888357	39888604	24chr19	45582357	45582606	24chr19	54023944	54024337	22chr19	58892041	58892331	19chr20	3801164	3801540	21chr20	60877650	60878004	24chr20	62612270	62612507	25chr2	129079385	129079666	22chr21	30445871	30446156	20chr2	202096980	202097231	20chr22	19132102	19132457	27chr2	219134761	219135270	21chr22	42062707	42063011	23chr2	27304134	27304386	23chr2	88990921	88991370	23chr3	139048238	139048480	22chr3	43732217	43732522	28chr3	45429956	45430186	22chr4	24586057	24586367	21chr4	492912	493128	23chr4	77997245	77997609	26chr5	112196784	112197107	21chr5	149380101	149380328	23chr5	72143767	72144106	24chr6	119558544	119558713	24chr6	170102037	170102332	20chr6	170863245	170863528	23chr6	26286596	26286881	25chr6	26341863	26342103	24chr6	28317590	28317893	24chr7	140179080	140179356	24chr7	23338668	23339030	25chr7	44084052	44084324	21chr7	6629423	6629736	22chr8	126442369	126442672	19chr8	144623607	144623906	27chr8	27630015	27630341	23chr8	82754694	82754936	27chr9	127533487	127533863	22chr9	139377372	139377676	21chr9	26892696	26892956	24chr9	37904234	37904509	26chr9	94877535	94877836	21chr9	96328643	96329005	25chr10	35379388	35379723	22chr10	71267638	71267851	19chr10	72575503	72575774	23chr11	111957338	111957668	21chr11	118888981	118889313	25chr1	160162984	160163180	22chr11	62413947	62414211	22chr11	64037332	64037655	19chr11	64455943	64456209	26chr11	65190143	65190436	27chr11	65625530	65625857	25chr1	204380941	204381281	21chr1	208136822	208137204	25chr1	209921007	209921424	25chr12	104359459	104359846	23chr12	122124842	122125190	30chr12	122985424	122985727	22chr12	132434335	132434577	25chr1	228688661	228688978	22chr1	230778001	230778360	22chr1	235267537	235267806	27chr12	46122579	46122835	28chr12	46384413	46384682	20chr12	56510216	56510528	23chr12	56511852	56512137	24chr12	57824748	57824984	22chr12	76742011	76742366	24chr1	28414987	28415294	24chr12	95867719	95868013	22chr13	100089205	100089484	26chr1	32403975	32404342	25chr14	24630285	24630533	23chr14	57735455	57735781	22chr14	69619779	69620022	24chr14	77564280	77564583	23chr14	88237571	88237848	27chr14	93673290	93673616	21chr15	34635200	34635549	22chr15	40615469	40615919	23chr16	15188029	15188316	22chr16	28956917	28957193	24chr16	30816726	30817034	23chr16	56998519	56998757	28chr16	58163196	58163574	25chr16	67189282	67189541	20chr17	28804176	28804513	22chr17	5372247	5372525	22chr17	55038246	55038595	23chr17	63119328	63119720	24chr17	78428387	78428707	24chr17	79075588	79075818	25chr1	894485	894743	19chr19	17416231	17416550	26chr19	3971143	3971464	19chr19	44617276	44617670	23chr20	19997787	19998049	25chr20	32900563	32900771	25chr20	44420413	44420669	23chr20	49126550	49127002	24chr21	33651274	33651643	21chr21	36423596	36423885	18chr2	220408461	220408786	23chr22	20861678	20862013	22chr22	22901558	22901785	23chr22	42084753	42085019	18chr22	42353605	42353912	23chr2	24270135	24270493	21chr2	32852983	32853336	21chr2	3381521	3381861	25chr2	85581573	85581846	22chr2	85843119	85843448	25chr2	99224867	99225155	25chr3	196669272	196669543	24chr3	50396936	50397194	25chr3	50606748	50607051	21chr4	106395102	106395351	28chr4	160024064	160024345	21chr4	2420460	2420713	23chr5	176881461	176881745	26chr5	178286750	178287239	25chr5	43064911	43065204	25chr5	72794101	72794375	19chr5	93954201	93954470	23chr6	106773461	106773759	23chr6	111580317	111580599	24chr6	26313256	26313520	22chr6	44042243	44042541	24chr7	100303463	100303810	21chr7	138915737	138916067	25chr7	148762915	148763195	26chr7	19748596	19748882	22chr7	74489498	74489794	22chr7	99006305	99006611	21chr7	99214392	99214637	24chr8	22102453	22102759	25chr8	95731912	95732171	24chr9	126964730	126965059	22chr9	130700008	130700323	18chr9	131644241	131644502	23chr9	132222654	132222989	22chr9	132586274	132586554	23chr9	138392348	138392608	20chr9	19380143	19380452	29chr9	33001513	33001752	19chr9	91925613	91925997	22chr10	104159248	104159533	26chr10	116697823	116698081	17chr10	124739873	124740209	25chr10	7860268	7860538	23chr10	85899158	85899414	20chr1	100435372	100435638	22chr11	124492579	124492892	23chr11	130786259	130786548	27chr1	155220521	155220756	22chr1	156163669	156163987	21chr11	65183896	65184095	25chr11	8008531	8008866	23chr12	120524731	120525041	27chr12	122326342	122326757	22chr12	125401706	125401928	18chr12	125412292	125412591	27chr1	231376773	231377037	20chr12	31812061	31812371	26chr1	236767685	236767998	26chr12	49961745	49962059	19chr12	98884781	98885132	21chr1	32421673	32421947	24chr13	33160371	33160664	23chr13	49821891	49822173	22chr1	35325280	35325602	22chr14	24685177	24685419	23chr1	45205286	45205618	22chr14	77494960	77495312	22chr15	40660278	40660576	22chr15	44092600	44092931	17chr15	45694360	45694639	20chr15	55611183	55611514	27chr15	65426109	65426344	20chr15	74284530	74284812	21chr15	80216094	80216419	23chr16	2059814	2060065	25chr1	6305192	6305403	23chr16	67906810	67907046	22chr16	69419711	69420040	22chr16	71929093	71929400	23chr16	89160124	89160389	23chr17	30228585	30228935	25chr17	36507367	36507745	21chr17	65362559	65362812	20chr17	73663223	73663519	20chr17	79633596	79633897	23chr18	47901142	47901485	21chr19	1174168	1174435	19chr19	30097025	30097284	18chr19	51611519	51611873	25chr19	54960134	54960411	20chr19	57922349	57922668	20chr20	25677297	25677676	23chr20	30326785	30327152	23chr20	34287175	34287424	21chr20	34638827	34639039	23chr20	60961930	60962193	23chr20	62339220	62339473	23chr2	11970369	11970707	21chr2	162016815	162017096	19chr2	183988935	183989184	19chr2	187454527	187454855	17chr2	190525840	190526326	21chr2	198299662	198300083	19chr22	21271343	21271708	22chr22	37252418	37252715	25chr22	40742348	40742660	23chr2	39005189	39005452	17chr2	9939819	9940066	23chr3	124449083	124449370	22chr3	15419714	15419982	22chr3	180319761	180320073	25chr3	44690080	44690299	21chr3	51422313	51422644	24chr3	51975982	51976390	24chr4	185655178	185655516	21chr4	69215675	69215953	18chr4	83812265	83812562	27chr5	167913305	167913576	25chr5	172386270	172386530	23chr5	43066958	43067237	25chr5	892809	893101	23chr6	26330442	26330673	24chr6	33547924	33548203	25chr6	41888710	41889011	21chr6	43603367	43603671	23chr6	44026160	44026549	23chr6	56819977	56820230	22chr7	100076673	100076998	21chr7	111846294	111846753	25chr7	121036230	121036618	26chr7	127225487	127225838	21chr7	5821214	5821451	21chr7	75157380	75157708	26chr8	37593985	37594256	22chr8	53626923	53627201	20chr9	140130935	140131208	23chr9	35603905	35604314	24chr9	4741070	4741362	21chrX	47052814	47053283	24chr10	70715716	70716036	22chr10	75544971	75545410	18chr10	79789213	79789503	24chr11	108093561	108093919	22chr11	129234093	129234325	20chr11	16759865	16760298	21chr1	150266127	150266415	19chr1	154155581	154155870	21chr1	156631134	156631392	21chr11	64794971	64795441	21chr11	65819560	65819946	24chr11	6624694	6624974	24chr11	68039418	68039739	23chr11	77705645	77705980	23chr11	82612572	82612894	19chr12	110337879	110338165	19chr12	25403774	25404012	22chr1	247536060	247536298	24chr12	4758069	4758387	23chr12	54121270	54121638	23chr12	69863970	69864268	21chr1	28832366	28832692	18chr12	96883150	96883421	24chr13	25496941	25497383	26chr1	32687812	32688112	21chr1	33592798	33593107	26chr13	60738030	60738298	22chr1	36615067	36615392	24chr14	24740679	24740966	26chr14	35761412	35761722	20chr14	54976404	54976639	21chr14	68066851	68067148	22chr14	95731879	95732104	23chr15	43477458	43477745	19chr15	49338560	49338867	23chr15	74988289	74988569	20chr16	1823029	1823243	24chr16	19535019	19535333	23chr16	3162410	3162620	21chr16	3450875	3451223	22chr16	57682977	57683208	25chr16	58426226	58426438	24chr16	66968208	66968505	21chr16	67219335	67219550	20chr16	734243	734546	22chr16	84220577	84220829	19chr16	85045021	85045312	23chr17	1303369	1303642	19chr17	40713998	40714226	25chr17	73285425	73285867	23chr17	79451303	79451592	22chr17	79479704	79479959	24chr19	10676536	10676838	22chr19	13044429	13044724	20chr19	2783310	2783611	22chr19	34268966	34269183	22chr19	45942758	45943327	25chr20	32700068	32700351	20chr20	60757872	60758146	22chr2	113484320	113484589	20chr2	15731832	15732091	21chr2	158732798	158733173	21chr2	174830023	174830384	21chr2	178029345	178029619	21chr2	178128215	178128453	24chr2	187350747	187351089	24chr2	191878920	191879198	24chr2	217276996	217277497	19chr22	21921814	21922126	22chr2	32264771	32265040	20chr2	55844753	55845134	27chr3	119182436	119182786	24chr3	186288027	186288370	21chr3	195808864	195809179	26chr4	41992367	41992675	22chr4	83295162	83295485	27chr5	148724745	148725093	26chr6	138725137	138725525	27chr6	144164274	144164582	19chr6	149969671	149969949	23chr6	26596020	26596316	26chr6	27440720	27441135	25chr6	53036750	53036972	22chr6	8064458	8064811	26chr7	126341154	126341369	22chr7	2281700	2281973	20chr7	92157882	92158182	25chr7	98923339	98923672	23chr8	67341040	67341293	26chr8	71520543	71520814	26chr8	95565712	95565953	26chr9	100174072	100174301	23chr9	102861179	102861491	24chr9	125693728	125694029	20chr9	132565283	132565544	22chr9	140149585	140149812	24chr9	19102829	19103097	21chr9	37784926	37785247	20chrX	12964961	12965230	24chr10	102133049	102133446	18chr10	124768240	124768598	21chr10	27530978	27531358	25chr10	46222499	46222717	25chr10	70660864	70661225	23chr10	88470865	88471126	23chr10	99894157	99894569	20chr1	10490333	10490586	21chr1	110091043	110091329	21chr11	47376806	47377039	23chr1	150241323	150241752	23chr1	155057265	155057538	21chr11	62389446	62389772	25chr1	165737974	165738329	22chr11	85339441	85339765	19chr12	104682493	104682802	20chr12	120739964	120740268	26chr1	224517991	224518269	21chr1	226374307	226374625	21chr12	31835164	31835358	25chr12	49412458	49412704	22chr12	49504467	49504794	20chr12	498374	498592	21chr12	54608508	54608825	23chr12	58329818	58330076	22chr1	26947182	26947545	24chr1	28908272	28908594	22chr1	32429761	32430013	24chr13	42535129	42535416	21chr14	90422077	90422331	26chr14	90722762	90722999	22chr15	40674972	40675262	23chr15	65903446	65903690	25chr16	22308456	22308719	27chr16	30569652	30569943	24chr16	30996275	30996548	20chr16	56642246	56642566	23chr16	66864738	66865018	18chr16	67880566	67880895	21chr16	69364373	69364703	22chr16	69788732	69788992	22chr16	74700644	74700974	21chr16	74847062	74847290	25chr16	771011	771327	22chr16	81515020	81515271	24chr17	21219877	21220207	21chr17	48238853	48239190	23chr17	76356874	76357184	25chr18	43753850	43754138	20chr19	10514086	10514379	20chr19	11205739	11206048	19chr19	12902183	12902430	21chr19	13991011	13991239	23chr19	40854253	40854516	24chr19	41768215	41768590	24chr19	4246873	4247198	22chr19	4457804	4458028	24chr19	44669018	44669263	17chr19	6862830	6863076	24chr2	114647300	114647580	27chr2	136499063	136499377	23chr2	169746824	169747164	22chr2	170550751	170551106	27chr2	178417390	178417916	25chr2	203103180	203103464	21chr2	220507248	220507584	24chr22	41032713	41032992	23chr2	29117304	29117630	24chr2	65454609	65454932	24chr2	85173391	85173799	22chr2	88355266	88355483	20chr3	14220113	14220428	21chr3	38206358	38206720	20chr3	9404567	9404887	18chr4	140216869	140217116	22chr4	153457218	153457477	21chr4	74088708	74088932	22chr5	121297435	121297688	25chr5	140044228	140044540	24chr5	140070786	140071126	22chr5	43105836	43106082	23chr5	76326018	76326300	23chr6	151773253	151773577	25chr6	26568979	26569182	24chr6	28048575	28048841	26chr6	28891587	28891940	21chr6	36842480	36842768	24chr6	37321604	37321938	18chr6	56954631	56954954	22chr6	83777184	83777464	22chr7	103848326	103848622	22chr7	56174175	56174441	24chr7	86781495	86781849	22chr7	99613059	99613302	23chr8	103823104	103823349	22chr8	110552153	110552464	23chr9	130497511	130497849	25chr9	131133461	131133787	25chr9	135230206	135230653	23chr9	33076554	33076877	23chr9	33264781	33265102	22chrX	10087576	10087805	24chrX	110924216	110924521	21chr10	21784681	21784954	23chr10	53459238	53459567	22chr10	65028898	65029161	22chr10	72124216	72124509	20chr10	977542	977821	18chr1	109289012	109289325	21chr11	108338058	108338350	22chr11	32605169	32605471	20chr11	450122	450375	21chr1	154193006	154193288	20chr1	160509974	160510243	26chr1	16302471	16302740	17chr11	64099124	64099566	20chr11	65244956	65245262	22chr11	65337772	65338007	16chr1	166845408	166845700	25chr11	67085083	67085362	19chr1	16767032	16767270	20chr1	186344567	186344940	24chr11	94801766	94802052	23chr1	205180748	205180964	22chr12	102513805	102514129	21chr12	108079364	108079630	21chr12	110888090	110888304	21chr12	112563151	112563476	19chr12	120933699	120934008	19chr1	213188975	213189290	18chr1	225662641	225662954	19chr1	235116455	235117062	20chr1	235530577	235530896	24chr1	236260401	236260714	24chr1	244462712	244463056	25chr12	56519974	56520221	22chr12	89919790	89920106	19chr13	114144992	114145396	20chr13	41706841	41707139	20chr1	3566506	3566827	25chr1	36396597	36396869	24chr1	40780977	40781200	19chr14	21131257	21131535	25chr14	24664764	24665045	21chr14	55493782	55494102	23chr1	46813787	46814043	25chr14	90865898	90866140	23chr15	49447699	49447971	22chr15	63449490	63449828	23chr15	77925859	77926149	21chr15	89182000	89182317	25chr16	11945263	11945583	19chr16	146956	147188	22chr16	1979719	1979965	26chr16	22217357	22217611	23chr16	30669293	30669679	23chr16	30759366	30759666	21chr16	30856055	30856317	21chr16	57450953	57451298	21chr17	15602781	15603080	17chr17	18684400	18684613	22chr17	42767002	42767336	19chr17	5342297	5342673	22chr17	60501102	60501360	23chr17	66201399	66201724	24chr17	73775631	73775926	24chr17	75084647	75084868	20chr17	79829203	79829437	20chr17	8279891	8280254	21chr18	13726485	13726779	24chr18	19180527	19180913	21chr18	32820816	32821168	21chr18	45275556	45275788	22chr18	48494205	48494553	21chr18	54305784	54306124	23chr1	8938638	8938909	20chr19	12886411	12886656	23chr19	23869837	23870188	22chr19	2819681	2819921	20chr19	33571908	33572202	18chr19	39971279	39971533	18chr20	33999634	33999956	21chr20	47950509	47950738	23chr20	55043505	55043846	23chr2	114341488	114341697	22chr21	34752856	34753127	20chr22	21213045	21213321	20chr22	46431821	46432109	19chr2	44314401	44314656	19chr2	55509262	55509565	21chr2	63815851	63816138	18chr2	71295404	71295778	19chr2	85647773	85648102	21chr3	150320836	150321221	23chr3	183966600	183966844	22chr3	52321886	52322169	19chr4	170678883	170679213	18chr4	185395713	185396034	25chr4	44680285	44680647	19chr5	134073987	134074207	22chr6	117996365	117996674	20chr6	163148734	163149065	19chr6	166755960	166756247	24chr6	20534574	20534875	22chr6	26104130	26104440	22chr6	26987938	26988250	24chr6	27094903	27095203	21chr6	27858357	27858709	25chr6	33239555	33239892	23chr6	35310069	35310450	23chr6	42018232	42018482	20chr6	43111278	43111552	22chr7	112758522	112758854	18chr7	150929404	150929698	21chr7	22862363	22862613	19chr7	93633599	93633863	19chr8	101162707	101162975	21chr8	107282237	107282612	20chr8	125462929	125463206	20chr8	99057637	99057953	20chr9	116163496	116163929	23chr9	128469333	128469681	20chr9	135819943	135820199	27chr9	136242811	136243090	21chrX	103401346	103401621	22chr10	113943430	113943685	24chr10	27443256	27443495	20chr10	6244597	6244915	18chr10	73610927	73611162	20chr10	74114326	74114767	24chr10	76995729	76995953	25chr10	94050718	94051041	20chr10	97849839	97850132	22chr11	10329351	10329787	22chr11	111472791	111473107	23chr11	118560786	118561108	23chr11	27528201	27528510	24chr1	151031923	151032226	20chr1	153541018	153541323	21chr1	155532816	155533156	21chr1	155826851	155827152	22chr1	166808442	166808749	23chr11	70049162	70049528	18chr1	171454442	171454691	21chr1	179846814	179847126	21chr11	82904555	82904815	19chr11	93394662	93395008	17chr1	201865109	201865368	21chr12	123459105	123459364	19chr12	125391366	125391572	20chr12	132413648	132413947	21chr12	1799921	1800194	21chr1	225632601	225632896	22chr1	235292031	235292344	22chr12	39300426	39300743	21chr1	24127265	24127506	21chr12	48099705	48099998	19chr12	56546117	56546373	21chr12	57940924	57941187	18chr12	64237649	64237880	19chr12	64797914	64798293	23chr12	69979071	69979539	21chr1	27216648	27216896	18chr1	29508468	29508752	21chr13	103451276	103451533	16chr13	25861524	25861771	19chr13	26796289	26796610	23chr13	33112838	33113174	20chr13	34392036	34392391	23chr13	41768469	41768878	19chr1	36863383	36863664	20chr1	3712990	3713234	21chr14	105947231	105947496	22chr14	23590000	23590272	20chr14	50583184	50583564	22chr1	46153698	46153989	23chr14	65453419	65453675	21chr14	74769575	74769918	19chr14	75179700	75179960	18chr15	34502234	34502507	21chr15	58624400	58624688	17chr15	66161348	66161747	22chr15	75939995	75940529	21chr15	91473246	91473507	22chr16	11038208	11038556	23chr16	12009827	12010181	24chr16	2053854	2054151	21chr1	62190681	62190956	21chr16	30366548	30366803	21chr16	30661756	30662081	19chr16	30961341	30961648	19chr16	81771658	81771879	20chr17	17494849	17495244	23chr17	40171943	40172155	20chr17	42287734	42288060	23chr17	56595483	56595788	19chr17	62340674	62340960	18chr17	80452487	80452785	22chr19	13056466	13056757	22chr19	13858550	13858872	22chr19	14682808	14683030	21chr19	16653142	16653449	19chr19	16770796	16771252	16chr19	19477644	19477902	20chr19	19516115	19516519	19chr19	28284606	28285038	19chr19	36485919	36486197	21chr19	36630799	36631076	20chr19	39322299	39322695	21chr19	39832529	39832800	17chr19	42612728	42612956	21chr19	47852300	47852610	18chr19	57831674	57831968	17chr19	58090224	58090526	20chr19	58427851	58428189	17chr19	58987252	58987563	20chr20	1450678	1451051	21chr20	18568471	18568755	22chr20	33872484	33872729	22chr20	33891698	33891936	19chr20	47804897	47805229	23chr20	49252478	49252753	19chr20	61371543	61371907	24chr2	101179185	101179548	20chr2	109065449	109065744	20chr2	113017099	113017315	22chr2	128051594	128051900	22chr2	134875904	134876269	26chr21	44344076	44344306	21chr2	171785332	171785598	23chr22	19718197	19718520	22chr2	220159629	220159824	22chr22	24191761	24192013	23chr2	238875471	238875671	20chr2	54013986	54014233	24chr2	54950364	54950741	22chr2	85822549	85822914	20chr2	96931632	96931986	20chr3	11313937	11314228	19chr3	130465555	130465831	23chr3	148943033	148943296	20chr3	193310885	193311099	20chr3	195641897	195642215	19chr3	39448034	39448356	20chr3	45698295	45698544	21chr3	50329818	50330071	25chr4	100009786	100010169	20chr4	120221663	120221949	23chr4	2936512	2936733	20chr5	172234241	172234499	21chr5	176883331	176883630	23chr5	60704420	60704736	20chr6	130005188	130005456	23chr6	139309224	139309653	22chr6	35435975	35436228	24chr6	41703236	41703534	24chr7	102004087	102004408	22chr7	108166482	108166831	24chr7	148663888	148664196	26chr7	148725465	148725940	20chr7	4815111	4815314	17chr7	5526519	5526825	21chr7	91875055	91875371	18chr7	92439069	92439307	20chr9	130477883	130478176	17chr9	131038261	131038581	17chr9	136009192	136009437	23chr9	71650522	71650805	22chr9	91933161	91933533	26chrX	108297650	108297862	21chrX	71496955	71497235	16chr10	106051221	106051449	20chr10	18948045	18948345	23chr10	22292544	22292890	21chr10	32635099	32635460	22chr10	46951316	46951583	18chr10	74856490	74856729	21chr11	15671645	15671884	18chr11	34195963	34196156	19chr11	47574654	47574899	24chr1	150898663	150898967	21chr1	154989993	154990257	21chr11	58347005	58347342	24chr11	60897312	60897521	23chr11	61584377	61584579	23chr11	64655380	64655715	22chr11	65239582	65239854	20chr11	65652177	65652396	20chr1	16947569	16947905	22chr1	17036331	17036571	21chr1	181111618	181111889	20chr12	118454353	118454646	21chr12	121019024	121019316	15chr12	21590461	21590744	19chr12	21654409	21654755	20chr1	226111724	226112065	21chr1	231558191	231558492	22chr1	235667785	235668152	25chr12	50794423	50794743	24chr12	72057515	72057844	22chr1	27648583	27648864	20chr12	93323073	93323461	21chr12	94496009	94496303	22chr1	29529351	29529613	19chr1	32409982	32410323	24chr13	50698453	50698730	22chr1	36023122	36023471	24chr1	37945276	37945559	18chr14	100659157	100659512	19chr14	23322300	23322569	19chr1	46806322	46806555	22chr1	47644853	47645092	22chr14	78174276	78174550	18chr15	40987153	40987467	21chr15	62682523	62682803	18chr15	64386088	64386325	20chr15	86383819	86384057	21chr15	90808697	90809051	21chr15	99791321	99791560	18chr16	21566204	21566463	18chr16	29801809	29802126	23chr16	30546219	30546505	19chr16	57481250	57481489	15chr16	67596032	67596315	21chr16	88729425	88729702	21chr17	1552150	1552351	22chr17	39819005	39819192	19chr17	40831676	40832029	21chr17	48796744	48797034	20chr17	76374585	76374799	19chr17	79935282	79935545	23chr19	2273701	2273978	24chr19	24184002	24184190	24chr19	33793343	33793649	21chr19	34919094	34919380	16chr19	36036297	36036617	21chr19	36239171	36239481	20chr19	41945648	41946012	18chr19	42954533	42954860	21chr19	44455172	44455417	20chr19	46320452	46320665	18chr1	948642	948907	17chr19	57862401	57862671	18chr19	58962724	58963094	19chr20	30198438	30198783	20chr20	40322079	40322320	24chr20	4153141	4153390	14chr20	44485904	44486235	21chr20	61283455	61283703	19chr20	62495529	62495807	19chr2	111435595	111435947	19chr21	30671077	30671356	19chr2	131130045	131130329	23chr2	170440729	170440963	20chr2	204103507	204103861	21chr2	219157152	219157405	20chr22	20918640	20918873	26chr22	31741840	31742092	23chr2	231921194	231921452	20chr2	241525601	241525914	22chr22	43263782	43264035	21chr22	43539325	43539632	20chr22	50946518	50946751	23chr2	32582018	32582310	25chr2	38152507	38152782	19chr2	55920866	55921186	17chr2	61763808	61764098	17chr2	99771356	99771646	19chr3	13036432	13036810	24chr3	133380665	133380942	19chr3	155588303	155588543	19chr3	172468179	172468551	18chr3	179065330	179065594	21chr3	186334831	186335098	16chr3	23847358	23847609	18chr3	37217718	37218007	18chr3	42003585	42003882	17chr3	44770968	44771173	20chr4	110354574	110354984	23chr4	113152427	113152824	18chr4	152682050	152682346	18chr4	169931285	169931670	22chr4	667983	668319	19chr5	140098334	140098583	21chr5	140943806	140944034	17chr5	141736565	141736824	21chr5	157170565	157170788	20chr5	175223896	175224136	15chr5	177540346	177540672	24chr5	177557822	177558065	22chr5	32585467	32585777	22chr5	68855849	68856154	20chr6	111303110	111303393	17chr6	159240332	159240575	18chr6	26285598	26285885	23chr6	33267060	33267429	22chr6	80254742	80255046	20chr7	100183638	100184002	21chr7	1032818	1033053	21chr7	104624225	104624567	23chr7	12726228	12726587	21chr7	2903314	2903567	20chr7	5553306	5553574	23chr7	75795770	75796078	20chr7	99517070	99517351	19chr7	99716840	99717174	21chr8	103250940	103251215	20chr8	117886930	117887225	19chr8	142318322	142318576	19chr8	22941733	22942007	16chr8	27695268	27695549	23chr8	57124214	57124453	20chr8	67837645	67837833	22chr9	115480251	115480561	19chr9	124082423	124082682	22chr9	129567107	129567429	20chr9	129622603	129622859	20chr9	130953736	130954021	24chr9	139702098	139702461	20chr9	140117950	140118300	21chrX	16737507	16737756	20chr10	102827624	102827856	15chr10	102973454	102973732	18chr10	134133740	134133952	16chr10	16859612	16859897	19chr10	71930164	71930447	20chr10	75700421	75700694	22chr11	129939637	129939875	18chr11	130731905	130732151	20chr11	18610133	18610480	20chr11	19092817	19093018	23chr11	20408701	20409244	21chr11	207303	207692	22chr11	4115765	4116033	21chr1	145610783	145611116	21chr11	62446427	62446712	16chr11	64614648	64614897	19chr11	66025740	66026024	23chr1	167772619	167772888	18chr11	71791592	71791925	21chr11	88070747	88071025	19chr1	20208635	20208947	20chr1	203595716	203595988	20chr1	209957863	209958166	24chr12	129308493	129308790	18chr1	21348835	21349117	17chr12	26427642	26427906	16chr1	23670691	23671002	21chr12	42631292	42631490	19chr1	247095126	247095411	20chr12	53374678	53374936	20chr1	28585689	28586155	19chr12	97300847	97301136	19chr13	24463438	24463729	20chr1	33177836	33178118	18chr13	34116796	34117146	18chr1	35318316	35318554	21chr1	36348569	36348832	21chr14	102829079	102829414	22chr14	21560712	21560925	17chr14	94547397	94547711	16chr15	73075983	73076247	18chr15	74286851	74287165	18chr15	74908008	74908297	23chr15	75092523	75092744	19chr1	61515591	61515853	19chr16	15684761	15685033	20chr16	231395	231695	25chr16	3333292	3333588	17chr16	3507855	3508137	19chr16	4817142	4817406	20chr16	85454414	85454639	20chr16	9221497	9221699	23chr1	714034	714228	18chr17	16438981	16439312	20chr17	19912429	19912744	22chr17	37778280	37778522	19chr17	38115286	38115493	20chr17	38231814	38232058	18chr17	42188577	42188800	20chr17	43275257	43275505	20chr17	48228399	48228676	22chr17	56769775	56770104	20chr17	57920535	57920811	20chr17	617978	618251	21chr17	72744478	72744849	21chr18	51750981	51751277	21chr1	85725214	85725537	19chr18	9475218	9475500	18chr19	13261085	13261363	18chr19	1897611	1897907	21chr19	2475978	2476239	23chr19	36207714	36208133	20chr19	38893634	38893958	20chr19	41035022	41035314	20chr19	51014426	51014823	22chr19	510682	510914	20chr1	95538355	95538660	20chr19	55629036	55629299	18chr19	6863582	6863818	21chr20	30102099	30102350	20chr20	48687851	48688052	19chr20	49434922	49435284	18chr20	61531631	61531941	19chr2	110962465	110962777	18chr2	118845743	118846046	20chr21	45553191	45553545	21chr21	46221626	46221906	15chr2	172543815	172544028	22chr2	201936549	201936840	19chr2	219166041	219166330	22chr2	219264303	219264607	23chr22	29949631	29949925	20chr2	232826259	232826557	19chr22	35772979	35773286	21chr22	36847830	36848051	23chr22	38245252	38245569	20chr2	239197488	239197794	21chr22	46692454	46692778	23chr2	42360191	42360475	22chr2	75937693	75938057	23chr3	113233817	113234137	21chr3	11685260	11685526	18chr3	127872134	127872354	21chr3	12909526	12909748	22chr3	146262242	146262552	21chr3	155572138	155572426	17chr3	185661321	185661590	20chr3	195577772	195578022	21chr3	197894474	197894747	20chr3	39093348	39093573	20chr3	50310365	50310634	20chr3	57541681	57542128	19chr4	77069528	77069799	24chr4	83350487	83350796	16chr5	139348423	139348653	20chr5	139554472	139554775	22chr5	175156892	175157154	21chr5	175815887	175816150	20chr5	6378511	6378823	18chr5	93447198	93447539	19chr5	95194502	95194775	19chr6	131949367	131949582	20chr6	136571355	136571621	23chr6	137540375	137540713	19chr6	158980956	158981193	21chr6	159125267	159125597	20chr6	170615647	170615959	20chr6	27560526	27560741	23chr6	28072434	28072703	21chr6	3024098	3024339	22chr6	3126670	3126919	18chr6	4021286	4021634	21chr6	41691185	41691527	17chr6	43149753	43149969	20chr6	43655391	43655721	23chr6	44205687	44205992	21chr6	53412787	53413026	24chr6	99873110	99873379	18chr7	102105216	102105524	24chr7	148936529	148936754	23chr7	150777812	150778088	19chr7	152373192	152373456	17chr7	27779575	27779839	18chr7	39605799	39606128	21chr7	39662995	39663278	17chr7	44896551	44896843	18chr7	5013528	5013773	20chr7	5085267	5085594	24chr7	5569210	5569549	18chr7	6098608	6098954	20chr7	73081974	73082235	21chr8	117767952	117768233	17chr8	124780565	124780838	19chr8	144911417	144911696	20chr8	56792191	56792502	16chr8	80997358	80997618	18chr9	125675396	125675753	22chr9	127952092	127952351	20chr9	131936359	131936591	21chr9	132175932	132176187	25chr9	140122007	140122236	18chr9	6412949	6413288	19chrX	100075223	100075474	17chrX	115004614	115004869	21chrX	129116362	129116672	21chrX	13752616	13752963	16chrX	152127338	152127709	19chrX	55187237	55187649	22chrX	64754701	64754952	16chr10	104182450	104182715	18chr10	112257470	112257726	21chr10	127407957	127408372	19chr10	131934480	131934769	15chr10	51565019	51565281	17chr10	65801017	65801263	19chr10	69609109	69609409	18chr10	88281466	88281742	17chr1	100731643	100731891	19chr11	119039252	119039476	19chr11	119204981	119205372	19chr1	115259279	115259555	22chr1	1334757	1335056	19chr11	448245	448571	19chr1	146556148	146556465	20chr1	149899608	149899892	19chr11	518640	519058	22chr1	153919121	153919370	22chr11	62432606	62432863	20chr1	162467459	162467751	21chr11	66049978	66050173	22chr11	66175955	66176170	21chr11	6628162	6628416	19chr11	67250404	67250779	16chr1	167597344	167597614	21chr1	174998883	174999196	18chr1	178062612	178062895	17chr11	85376045	85376350	20chr11	87063814	87064107	18chr11	96122941	96123291	20chr1	201924438	201924754	21chr1	202088139	202088364	22chr1	202858014	202858323	19chr1	20834525	20834792	20chr12	110906986	110907247	19chr12	11324052	11324321	18chr12	117175920	117176172	20chr12	120632362	120632654	19chr1	212208447	212208855	18chr12	122235017	122235295	20chr12	2113600	2113866	19chr1	22379055	22379277	20chr1	244615347	244615718	22chr1	246862480	246862758	20chr12	56583983	56584203	19chr12	685340	685591	20chr1	28499244	28499515	19chr12	9803390	9803615	15chr13	111617738	111617958	21chr13	114992235	114992504	19chr13	48612031	48612314	19chr13	92001145	92001517	21chr13	99128743	99129015	18chr14	24711754	24711999	15chr14	69283416	69283683	17chr14	73603005	73603287	19chr1	51425862	51426189	22chr15	41523105	41523454	18chr1	54303991	54304437	21chr15	68155790	68156089	21chr15	68569939	68570310	21chr15	72978348	72978679	19chr15	89600821	89601032	18chr15	89786994	89787294	20chr16	27279986	27280243	18chr16	2977073	2977320	21chr16	31454336	31454606	19chr16	66550727	66551048	20chr16	67875751	67876100	22chr16	68074531	68074829	20chr16	88803768	88804088	22chr17	1588016	1588362	15chr17	27181800	27182188	21chr17	40706637	40706891	22chr17	40976177	40976430	17chr17	43225633	43226039	18chr17	48450373	48450651	15chr17	6917847	6918295	18chr17	72787681	72787898	20chr17	73267261	73267542	19chr19	10613345	10613586	21chr19	1113316	1113627	21chr19	12888426	12888642	23chr19	12904310	12904624	22chr19	1383642	1383930	22chr19	13885109	13885309	18chr19	17970374	17970775	17chr19	3155126	3155486	20chr19	3505888	3506178	20chr19	35464149	35464382	21chr19	36139024	36139313	19chr19	3761566	3761903	20chr19	40502713	40503065	18chr19	46146398	46146650	21chr19	488913	489226	19chr19	53606563	53606825	16chr19	55727845	55728120	21chr19	58038565	58038893	17chr19	58361042	58361397	18chr19	58919717	58920105	24chr19	6361361	6361699	19chr19	7990101	7990381	18chr19	8645692	8645917	19chr20	18268706	18269011	16chr20	21283815	21284058	16chr20	25371600	25371860	18chr20	30311620	30311934	21chr20	44044613	44044881	18chr20	46012796	46013059	21chr2	11724331	11724644	15chr21	30396862	30397167	17chr21	40760500	40760753	18chr21	44527648	44527935	19chr21	45663849	45664186	18chr2	170681259	170681573	22chr2	174904570	174904892	20chr2	198364645	198364936	21chr2	201374662	201374936	22chr2	216973894	216974235	18chr2	219137403	219137752	17chr22	24552923	24553141	18chr22	41079058	41079402	19chr2	30934538	30934763	17chr2	43823038	43823368	21chr2	44001005	44001306	18chr2	48667592	48667883	17chr2	73089690	73089939	21chr3	120277737	120278020	18chr3	141087050	141087442	20chr3	169755565	169755914	20chr3	171178089	171178468	21chr3	186524141	186524478	20chr3	44666348	44666587	18chr3	47563667	47563897	16chr3	48646968	48647290	20chr3	53164298	53164613	18chr3	93781592	93781952	19chr3	9932196	9932467	18chr4	129375440	129375726	17chr4	154354304	154354590	20chr4	159644440	159644738	20chr4	17812233	17812558	20chr4	184328267	184328514	19chr4	184365601	184365898	22chr4	185435552	185435795	19chr5	10284178	10284416	18chr5	130599622	130599919	20chr5	137799750	137800009	19chr5	59995864	59996129	20chr5	86708610	86708862	22chr5	95296919	95297204	21chr6	135644363	135644668	20chr6	147178691	147178927	19chr6	170862249	170862485	19chr6	26156468	26156761	19chr6	33756804	33757125	19chr6	34725119	34725357	17chr6	36409198	36409445	18chr6	88466644	88466939	17chr6	96969507	96969768	18chr7	102036605	102036861	18chr7	104585529	104585799	22chr7	127032673	127032911	15chr7	128864307	128864704	20chr7	132937601	132937888	17chr7	23636838	23637170	21chr7	38375226	38375540	18chr7	43878414	43878732	19chr7	6120977	6121327	18chr7	64126323	64126654	19chr7	6523670	6523962	23chr7	66205467	66205791	21chr7	6769144	6769489	19chr7	72349707	72350007	21chr7	99756234	99756494	23chr8	101912670	101912943	19chr8	103875106	103875514	19chr8	11626967	11627296	21chr8	144349434	144349682	20chr8	146277768	146277981	17chr8	23104008	23104382	22chr8	30013621	30013967	18chr8	37619958	37620247	21chr8	82598413	82598650	19chr9	128023825	128024124	18chr9	130965470	130965745	24chr9	137029924	137030219	16chr9	95432323	95432625	17chr9	98980468	98980756	18chrX	118699284	118699541	18chrX	1710307	1710562	18chrX	46404655	46405047	21chr10	102295504	102295791	18chr10	103929211	103929489	21chr10	121356422	121356862	23chr10	3239635	3240145	23chr10	73137055	73137366	18chr1	109505919	109506221	17chr1	109825792	109826130	16chr11	105892991	105893327	22chr11	118798700	118799092	16chr11	119537202	119537422	16chr11	126173808	126174153	16chr1	112933514	112933724	19chr1	113008759	113009113	21chr1	114301941	114302346	18chr1	116681717	116681947	21chr1	117369900	117370135	18chr11	18655797	18656096	16chr1	12079212	12079509	16chr1	12221908	12222200	19chr11	46368794	46369067	18chr1	150039610	150039944	18chr1	151431877	151432147	18chr1	153931043	153931315	20chr1	155163298	155163593	19chr1	155210974	155211198	17chr1	156024349	156024668	19chr11	65729020	65729266	18chr11	73372017	73372296	20chr1	202927324	202927564	18chr12	104609421	104609651	18chr1	211848954	211849303	19chr1	211871900	211872157	21chr12	12966106	12966405	16chr12	14956331	14956680	19chr1	21503149	21503436	17chr12	16035162	16035435	16chr12	27167175	27167473	20chr1	228270254	228270546	15chr1	229694537	229694878	21chr1	234748413	234748718	18chr1	236958243	236958695	18chr1	23866301	23866623	21chr1	24194732	24195048	18chr12	53689138	53689402	17chr12	54773461	54773688	19chr12	6982445	6982779	20chr12	7260984	7261276	15chr13	101327140	101327448	16chr13	103452514	103452861	17chr13	113951238	113951491	22chr1	32645137	32645483	19chr13	31773929	31774210	15chr13	48877730	48878008	15chr14	31028208	31028494	17chr1	43855344	43855612	19chr14	50329549	50329848	24chr14	75593636	75593918	18chr14	81407999	81408263	19chr15	64445788	64446067	16chr15	77835729	77835999	20chr15	83518395	83518651	19chr15	83680368	83680603	17chr15	85874297	85874699	22chr15	89089820	89090104	20chr15	90708938	90709293	21chr16	14394755	14394978	17chr16	279229	279595	22chr16	30406549	30406841	20chr16	3109262	3109584	19chr16	3156405	3156616	20chr16	57278724	57279084	19chr16	69220841	69221108	19chr16	75681451	75681773	16chr16	84764100	84764333	18chr16	85644104	85644390	19chr16	857713	857982	21chr16	85833132	85833384	18chr17	27475859	27476136	18chr17	37886653	37886949	16chr17	41176462	41176780	19chr17	43025065	43025354	16chr17	43462946	43463146	19chr17	48246675	48246954	21chr17	48556045	48556281	19chr17	57863917	57864229	20chr17	62493068	62493397	20chr17	7183888	7184214	17chr17	7218614	7218852	21chr18	21032966	21033301	17chr1	840014	840297	22chr19	10216857	10217141	19chr19	12750960	12751234	22chr19	12917223	12917522	16chr19	14185891	14186218	17chr19	14685124	14685311	21chr19	16222072	16222376	17chr19	18475791	18476046	20chr19	18632983	18633336	19chr19	19774375	19774683	19chr1	93297416	93297665	20chr19	34992450	34992743	18chr19	36184732	36184982	23chr19	3706120	3706372	20chr19	39390330	39390645	16chr19	39993458	39993738	18chr19	41076552	41076909	16chr19	44506901	44507183	21chr19	46386626	46386863	18chr19	47354043	47354350	21chr19	47747484	47747697	19chr19	48837214	48837463	21chr19	49339070	49339299	18chr19	52074315	52074569	22chr19	57874632	57874961	18chr19	58280859	58281136	20chr19	58694443	58694844	17chr20	1447483	1447699	19chr20	30697325	30697618	21chr20	33460551	33460870	21chr20	34035041	34035325	20chr20	35724387	35724595	21chr20	37101178	37101519	22chr20	43991554	43991955	23chr20	48429037	48429511	18chr20	48763500	48763759	19chr2	102857290	102857547	20chr2	109252868	109253086	17chr2	114644090	114644446	22chr2	120770375	120770715	17chr2	128848490	128848807	21chr2	171829927	171830247	21chr2	178257317	178257599	21chr2	179387741	179388031	19chr2	207024151	207024480	18chr22	20067316	20067582	18chr2	231280733	231281049	18chr22	31503663	31503902	19chr22	31556012	31556248	18chr2	232578555	232578826	17chr22	41252960	41253226	19chr22	41487532	41487858	21chr22	45080631	45080899	20chr2	28974245	28974589	21chr2	47214199	47214452	19chr2	67624246	67624560	22chr2	70056561	70056886	18chr2	87035531	87035775	23chr3	100119867	100120170	16chr3	123679981	123680276	14chr3	128839420	128839683	18chr3	14693088	14693323	17chr3	179040620	179040823	15chr3	194054526	194054824	21chr3	194304583	194304876	17chr3	49893865	49894175	19chr3	52443953	52444158	15chr3	87276222	87276531	18chr4	100484808	100485269	22chr4	148605199	148605497	22chr4	159593211	159593549	24chr5	133375280	133375486	21chr5	1345156	1345382	19chr5	141258765	141259038	22chr5	147763341	147763566	19chr5	157158251	157158556	17chr5	179245921	179246245	18chr5	41904185	41904524	19chr5	74532667	74532979	18chr5	94982378	94982702	21chr6	11093813	11094089	17chr6	154996745	154997015	22chr6	160147863	160148123	19chr6	17600378	17600643	18chr6	30646833	30647060	21chr6	33176113	33176344	17chr6	41286597	41286850	22chr6	42981683	42982054	18chr6	43197088	43197369	21chr6	46816711	46816955	20chr6	6547328	6547614	19chr6	7313432	7313766	15chr6	83902866	83903067	18chr7	101331568	101331903	21chr7	1199669	1199987	20chr7	128731637	128731874	19chr7	129845057	129845378	16chr7	138802975	138803224	15chr7	140217768	140218063	18chr7	23312743	23313107	16chr7	6388566	6388820	20chr7	65670084	65670372	19chr7	97601530	97601847	20chr8	100025324	100025587	22chr8	125985342	125985656	20chr8	141522054	141522326	18chr8	145582058	145582385	20chr8	145597390	145597749	18chr8	21867623	21867966	17chr8	59714057	59714317	20chr9	127474274	127474476	19chr9	35665085	35665315	16chr9	6681156	6681616	19chr9	6757552	6757917	16chrX	108297523	108297655	21chrX	130037072	130037446	17chrX	70585649	70585938	19chr10	101491741	101492053	17chr10	105127508	105127798	14chr10	105668088	105668337	19chr10	112533978	112534217	17chr10	123734571	123734905	16chr10	13203300	13203538	19chr10	33247232	33247494	16chr10	43913551	43913812	20chr10	4891886	4892151	17chr10	51827526	51827764	21chr10	65225611	65225863	19chr10	71152310	71152587	18chr1	109102437	109102720	20chr11	10388197	10388443	20chr11	10814080	10814299	18chr11	114271066	114271382	18chr11	126138601	126138959	15chr1	113162090	113162465	21chr1	120190875	120191158	20chr1	12664375	12664659	18chr1	145438313	145438615	18chr1	145516172	145516449	16chr11	46260757	46260999	22chr11	47789017	47789324	15chr1	150293825	150294141	18chr11	506671	506945	17chr1	154934132	154934431	18chr11	61100438	61100745	19chr11	63753710	63754048	18chr11	64008258	64008569	17chr11	6633390	6633650	18chr11	73018347	73018626	19chr11	74952726	74952930	20chr11	85358819	85359120	17chr1	206663469	206663776	18chr1	207925201	207925534	23chr12	112123651	112123956	20chr1	211555985	211556270	19chr12	117348590	117348894	21chr12	125424333	125424657	21chr12	132195413	132195727	19chr1	22110041	22110280	21chr1	24285505	24285777	20chr12	50101285	50101570	22chr12	53764903	53765245	22chr12	54140490	54140728	18chr12	56223317	56223626	22chr1	26437972	26438330	18chr12	6833081	6833377	23chr12	7033513	7033754	18chr12	72332571	72332764	17chr1	29562780	29563087	16chr13	108870674	108870918	21chr13	111064543	111064849	19chr13	111358403	111358691	20chr13	45491998	45492358	19chr1	36839407	36839734	15chr13	79233137	79233422	22chr1	38019791	38020020	17chr1	38478144	38478532	17chr14	105553170	105553456	17chr1	41469431	41469691	23chr14	24025221	24025633	18chr14	24898966	24899269	17chr14	31091308	31091616	18chr14	35008739	35008981	19chr14	51706831	51707112	15chr14	74353345	74353620	19chr15	40074871	40075183	17chr15	40566938	40567176	21chr15	41149907	41150176	20chr15	59949591	59949877	17chr15	73928835	73929091	16chr15	74838547	74838793	21chr15	75339445	75339731	18chr15	75497124	75497378	19chr15	75628242	75628460	22chr15	85174494	85174736	23chr15	91565713	91566028	17chr15	93447346	93447648	21chr15	99602259	99602494	17chr16	15149941	15150242	18chr16	1524973	1525201	23chr16	3074098	3074429	19chr16	30786651	30786937	16chr16	3179539	3179827	17chr16	4784160	4784476	19chr1	65329838	65330120	21chr16	577316	577775	20chr16	70332933	70333317	19chr1	67966162	67966400	17chr17	17942421	17942714	17chr17	19281105	19281455	18chr17	40474819	40475056	19chr17	41837785	41838065	17chr17	45908769	45909099	19chr17	4693211	4693488	16chr17	56494824	56495093	17chr17	57906621	57906965	20chr17	73042916	73043251	17chr17	74381872	74382221	19chr17	79995186	79995466	17chr18	51884094	51884356	16chr1	856499	856711	20chr18	60382117	60382437	20chr19	10044859	10045094	18chr19	1240301	1240524	22chr19	13076170	13076437	17chr19	18117866	18118078	20chr19	1848616	1848934	17chr19	1876060	1876339	16chr19	21512046	21512348	16chr19	41222700	41223032	20chr19	45596035	45596392	20chr19	46898878	46899086	21chr19	58666399	58666667	19chr19	633538	633793	18chr19	6674907	6675190	17chr20	16555397	16555712	18chr20	18547835	18548127	20chr20	30795474	30795698	20chr20	32251423	32251695	17chr20	43238634	43238864	15chr20	48595583	48595898	18chr20	48732432	48732647	16chr20	49411290	49411570	19chr20	56784935	56785154	18chr20	62694230	62694497	18chr20	62917344	62917554	20chr2	113299865	113300144	21chr2	130939510	130939883	23chr21	45358976	45359266	20chr21	46560319	46560592	18chr2	149267041	149267309	17chr2	153032357	153032687	19chr2	174828541	174828862	21chr2	190627330	190627610	19chr2	203499363	203499676	20chr2	20861274	20861493	18chr2	220082558	220082837	19chr22	21457109	21457365	20chr22	22307108	22307488	19chr22	45371500	45371732	14chr22	46646115	46646411	18chr22	50765475	50765760	18chr2	27593256	27593510	18chr2	47499499	47499828	16chr2	48338386	48338647	18chr2	64751236	64751500	21chr2	65144824	65145109	22chr2	65659558	65659895	21chr2	68694383	68694711	19chr2	73404479	73404765	18chr2	86116127	86116453	18chr3	101443245	101443718	15chr3	120068052	120068388	20chr3	170587802	170588088	16chr3	197237053	197237288	18chr3	197632966	197633179	19chr3	32509322	32509598	19chr3	33839940	33840217	18chr3	46037186	46037480	17chr3	47554837	47555187	19chr3	50126147	50126429	16chr3	53925852	53926212	22chr3	56528795	56528970	17chr3	57741825	57742084	20chr3	88198936	88199199	20chr3	99979458	99979823	18chr4	128801787	128802094	19chr4	2010872	2011174	17chr4	3108365	3108600	18chr4	39640516	39640870	16chr4	76598637	76598914	15chr4	76912066	76912264	18chr5	10628019	10628262	19chr5	137785699	137785941	17chr5	140105589	140105851	21chr5	142784792	142785123	18chr5	148930999	148931353	22chr5	150880313	150880604	17chr5	159435987	159436253	19chr5	179720539	179720775	15chr5	32174342	32174601	16chr5	60627902	60628149	17chr5	68665265	68665652	20chr5	81267724	81268001	17chr5	85913612	85913836	19chr5	94890605	94890972	18chr6	109416485	109416770	23chr6	27125786	27126035	23chr6	27144852	27145137	22chr6	27300694	27300971	18chr6	31548641	31548932	19chr6	31648531	31648832	17chr6	34191883	34192127	16chr6	37139961	37140200	20chr6	41649926	41650151	19chr6	79944255	79944571	15chr6	88299561	88299909	16chr7	1105413	1105694	19chr7	134671233	134671649	20chr7	138037406	138037675	21chr7	44530109	44530451	19chr7	64466938	64467283	16chr7	766151	766414	21chr7	96339046	96339381	19chr8	104427423	104427676	20chr8	10785985	10786263	16chr8	10873925	10874218	20chr8	145911188	145911469	22chr8	22526491	22526780	16chr8	22613621	22613997	21chr8	26240264	26240555	15chr8	27219637	27219927	18chr8	27950465	27950743	19chr8	41998177	41998406	16chr8	86132861	86133174	20chr9	104160796	104161121	18chr9	114658857	114659111	20chr9	131902298	131902554	15chr9	134553915	134554170	20chr9	134615474	134615698	20chr9	135282131	135282345	15chr9	136019813	136020101	16chr9	140513080	140513406	23chr9	33290309	33290552	19chr9	35812118	35812388	16chrX	131352146	131352404	17chrX	64815351	64815673	21chr10	103578065	103578376	18chr10	103892602	103892883	19chr10	3852371	3852685	21chr10	60144928	60145298	18chr10	64564283	64564633	19chr10	71892275	71892736	19chr10	80955234	80955473	17chr11	10757357	10757606	18chr11	107879445	107879720	15chr11	108368365	108368654	15chr11	118661866	118662108	16chr1	112289950	112290148	16chr1	1167266	1167578	19chr1	117099257	117099491	17chr11	19262358	19262737	18chr1	1209101	1209354	19chr11	43702148	43702457	16chr1	145059026	145059261	18chr1	150952025	150952249	18chr1	153539040	153539270	20chr1	156571149	156571455	19chr1	160001642	160001937	20chr11	60542905	60543110	17chr1	161283976	161284260	17chr1	161719411	161719686	19chr1	16279288	16279538	15chr11	64109890	64110166	18chr11	65585710	65585981	15chr11	69489942	69490281	20chr1	172903091	172903332	19chr11	75918649	75918906	20chr11	925586	925960	20chr1	204732915	204733185	17chr1	208418595	208418830	15chr12	105501321	105501616	17chr12	111325895	111326068	18chr12	113859933	113860297	15chr12	122492322	122492526	17chr12	123465094	123465347	19chr12	124068864	124069084	21chr1	212588133	212588505	21chr12	127650459	127650642	20chr1	224701399	224701703	21chr1	231347150	231347415	21chr12	48551153	48551437	16chr12	51157568	51157785	19chr1	25174465	25174745	18chr12	53718584	53718845	17chr12	56709830	56710218	20chr1	2574538	2574818	18chr12	6888103	6888380	18chr1	28969462	28969780	18chr1	33115991	33116259	17chr13	32889421	32889722	19chr1	38061395	38061674	17chr13	97762197	97762419	18chr1	40997100	40997409	15chr14	103851539	103851842	15chr14	105442537	105442744	19chr14	105948302	105948521	21chr14	23527302	23527540	18chr14	23764268	23764524	20chr1	43232756	43233066	19chr14	52456015	52456284	18chr1	45285477	45285816	18chr14	54908032	54908442	17chr14	64010117	64010478	16chr14	68162384	68162641	19chr14	68283239	68283555	19chr14	69291537	69291816	19chr14	77425703	77426033	15chr15	22461021	22461320	21chr15	55790497	55790719	12chr15	67143591	67143809	18chr15	67196062	67196321	18chr15	67383613	67383891	20chr15	90863283	90863516	15chr1	59249528	59249837	12chr16	19566586	19566860	20chr16	19729417	19729700	16chr16	30420805	30421036	19chr1	63249556	63249887	17chr16	47494999	47495282	21chr16	68027223	68027456	17chr16	68271448	68271737	23chr16	70462027	70462483	16chr16	71518394	71518654	16chr16	74640826	74641140	17chr16	75018956	75019307	14chr16	85649573	85649809	20chr16	89233857	89234090	17chr16	89571932	89572155	16chr17	16289984	16290333	19chr17	18528957	18529213	19chr17	27053933	27054181	18chr17	30677001	30677335	16chr17	36714410	36714646	19chr17	37844128	37844534	17chr17	38210568	38210861	16chr17	40336176	40336442	20chr17	40729578	40729961	19chr17	41471374	41471641	20chr17	43971628	43971901	20chr17	5015115	5015344	19chr17	63133220	63133549	20chr17	73389922	73390193	17chr17	74117520	74117805	18chr17	74519216	74519497	21chr17	76247021	76247264	17chr17	79818790	79819117	17chr18	33709712	33709982	16chr18	61089669	61089938	16chr18	72163250	72163519	16chr18	9136411	9136691	18chr19	12035720	12036047	14chr19	15288354	15288570	19chr19	2051201	2051507	17chr19	2474668	2474895	16chr19	36103523	36103716	18chr1	937237	937460	17chr19	37861779	37862058	14chr19	39926585	39926896	23chr19	42463399	42463778	19chr19	42748830	42749139	20chr19	42806588	42806889	18chr19	45927042	45927288	13chr19	46367333	46367624	16chr19	49496993	49497248	13chr19	55671944	55672184	18chr19	9785612	9785957	16chr20	20032950	20033271	15chr20	30135006	30135286	17chr20	31240685	31240982	18chr20	34252767	34253026	16chr20	36322209	36322513	21chr20	44563198	44563472	18chr20	45825071	45825347	16chr20	49574934	49575274	15chr20	52824254	52824548	20chr20	61904013	61904288	16chr20	62587702	62587959	21chr20	62605258	62605465	18chr2	110371603	110371916	21chr21	15755345	15755720	15chr2	128568700	128568989	18chr21	33942115	33942385	19chr2	172778771	172779102	20chr22	35746799	35747069	19chr22	36851017	36851322	21chr2	239148513	239148821	15chr22	39378204	39378481	18chr22	41985920	41986290	19chr22	50364442	50364707	20chr2	43447025	43447348	15chr2	62266521	62266748	21chr2	69951830	69952044	17chr2	73114998	73115199	18chr2	74692392	74692712	18chr2	75873763	75874080	17chr2	85839110	85839392	17chr2	9282867	9283156	19chr2	97534236	97534495	17chr3	115503297	115503590	17chr3	121264752	121264986	20chr3	13691936	13692303	19chr3	155523962	155524227	19chr3	15642978	15643303	17chr3	156799440	156799783	17chr3	196594641	196594884	16chr3	32612146	32612465	18chr3	38537626	38537941	17chr3	39329909	39330199	16chr3	46120388	46120589	18chr3	49577172	49577448	18chr3	5017883	5018150	19chr3	52188682	52189011	18chr3	57969525	57969774	18chr3	88108165	88108483	16chr3	9773005	9773311	18chr4	119606312	119606677	16chr4	144105933	144106219	19chr4	152020587	152020925	14chr4	185185376	185185649	17chr4	185276339	185276615	19chr4	2443418	2443694	20chr4	57844898	57845175	19chr4	76439502	76439814	17chr4	84031579	84031849	18chr5	131892487	131892753	20chr5	149185429	149185671	20chr5	153989960	153990182	19chr5	172192340	172192638	19chr5	173043639	173043912	18chr5	176778531	176778870	17chr5	36876672	36876896	20chr5	5422249	5422570	18chr5	79703517	79703867	23chr5	80490873	80491166	16chr5	89825265	89825574	18chr6	107435465	107435735	19chr6	110501142	110501451	18chr6	149753173	149753407	18chr6	153323740	153324062	19chr6	2932468	2932821	20chr6	29617793	29618089	14chr6	34164071	34164339	19chr6	36953771	36954089	18chr6	37759208	37759507	19chr6	4018843	4019115	19chr6	43543671	43543988	20chr6	8102596	8102926	15chr6	89673195	89673491	19chr6	91078806	91079062	21chr7	100720903	100721164	20chr7	117824065	117824358	20chr7	128095579	128095895	22chr7	149119431	149119710	19chr7	150725665	150725969	17chr7	27200599	27200852	18chr7	42971690	42972061	16chr7	5279924	5280128	18chr7	87563321	87563698	19chr7	913034	913316	16chr7	97735975	97736226	17chr7	99724408	99724749	20chr8	128746277	128746547	18chr8	141647517	141647778	18chr8	144512969	144513197	17chr8	21894355	21894596	17chr8	30670236	30670494	17chr8	48920855	48921113	20chr8	74205722	74205944	13chr8	94766949	94767218	17chr9	131464606	131464932	19chr9	131625969	131626168	18chr9	133922166	133922416	20chr9	19408789	19409115	18chr9	37913638	37913914	16chr9	4662080	4662398	15chr9	95640243	95640448	18chrX	118602910	118603224	19chrX	149106668	149106951	18chrX	48397707	48397961	18chrX	52004634	52004988	16chrX	53461199	53461527	14chrX	73770188	73770414	20chr10	103113484	103113808	17chr10	103824984	103825244	16chr10	104153727	104154164	19chr10	112261042	112261336	17chr10	17270369	17270685	16chr10	38383061	38383322	17chr10	58120896	58121161	15chr10	60094611	60094996	14chr10	74870102	74870345	16chr10	88854514	88854755	15chr10	96943403	96943644	18chr1	100598527	100598809	17chr11	118938318	118938598	18chr11	118972681	118973003	20chr1	112298192	112298472	19chr1	118301471	118301772	17chr11	3400232	3400522	18chr11	43665623	43665848	17chr11	44642731	44643026	17chr11	46722062	46722420	19chr1	155022989	155023208	16chr1	155243266	155243528	18chr1	161015651	161016004	19chr1	161087677	161087959	17chr11	61197422	61197677	19chr11	62559316	62559612	16chr1	16382642	16382842	15chr11	64071840	64072204	15chr1	16514314	16514601	18chr11	65236350	65236610	18chr11	65430338	65430778	19chr11	65479344	65479567	17chr11	66462267	66462487	18chr1	169075424	169075696	17chr11	76510264	76510611	19chr11	76802232	76802517	16chr11	8350427	8350657	19chr1	185126144	185126427	18chr11	85904547	85904847	19chr11	9482277	9482620	19chr1	200589776	200590133	15chr1	200609126	200609442	16chr1	211307462	211307753	20chr12	114404020	114404342	16chr12	123380540	123380848	17chr1	227015772	227016044	20chr1	22743117	22743359	16chr12	30907806	30908155	20chr12	39836880	39837143	17chr12	44199969	44200298	18chr12	48357149	48357432	20chr12	51477290	51477524	20chr12	54585346	54585602	20chr12	56109726	56109983	21chr12	56521726	56522145	16chr12	56615560	56615951	16chr12	65563193	65563475	17chr12	66696145	66696477	18chr12	69201737	69202077	19chr12	69202582	69202844	18chr12	69633147	69633499	15chr12	76478247	76478648	18chr12	80328898	80329239	20chr1	28052106	28052392	16chr12	95467330	95467543	21chr1	29557326	29557560	19chr13	111108320	111108554	17chr13	53542268	53542487	17chr13	77901287	77901504	19chr1	37980294	37980572	18chr1	38273740	38273989	18chr14	102990822	102991173	17chr14	106873510	106873781	17chr14	23341816	23342092	17chr14	23755382	23755727	15chr14	24682553	24682897	18chr14	50053033	50053338	19chr14	55658205	55658524	18chr14	68141310	68141810	18chr14	77787121	77787563	17chr1	52870134	52870427	18chr1	53662328	53662625	19chr15	41234044	41234334	20chr15	45455291	45455556	19chr15	64648325	64648606	19chr15	67066754	67067009	20chr15	72668290	72668516	18chr15	76050716	76051002	20chr15	77789170	77789424	18chr15	82554932	82555234	17chr15	82824825	82825049	13chr16	19097870	19098129	20chr16	19918840	19919104	15chr16	2009352	2009625	14chr16	2570264	2570447	21chr1	6265267	6265511	16chr16	2954157	2954448	16chr16	3020099	3020428	20chr16	3053814	3054127	17chr16	30609546	30609782	16chr16	31724416	31724708	15chr16	31885844	31886121	16chr16	4524544	4524827	16chr16	58283739	58283953	18chr16	67051862	67052061	16chr16	69373486	69373765	13chr16	69760259	69760540	19chr16	74330557	74330892	17chr16	75032408	75032792	20chr16	75551279	75551537	17chr16	83841448	83841719	17chr16	8617751	8617984	15chr16	8974147	8974377	18chr17	21117392	21117702	17chr17	26821812	26822017	18chr17	27046840	27047174	18chr17	33416388	33416656	16chr17	38170032	38170297	17chr17	45967337	45967584	18chr17	4848526	4848814	17chr17	61819218	61819484	16chr17	61850972	61851266	17chr17	61904303	61904649	15chr17	7387653	7387902	17chr17	73975510	73975844	19chr17	74733316	74733646	18chr17	7591501	7591753	22chr17	76210158	76210418	17chr17	7755141	7755337	15chr17	78075110	78075444	18chr17	8057532	8057821	17chr18	21083096	21083470	18chr18	32870205	32870436	14chr18	33647438	33647758	17chr19	10138419	10138644	17chr19	1095387	1095618	18chr19	11616523	11616854	14chr19	11669964	11670262	19chr19	17716887	17717136	19chr19	3435150	3435441	17chr19	37157651	37157896	18chr19	37958307	37958499	19chr19	40949045	40949330	16chr19	44809001	44809325	20chr19	45988585	45988825	15chr19	46234101	46234334	17chr19	46272616	46272882	17chr19	47288016	47288269	14chr19	47759591	47759858	17chr19	49314099	49314360	21chr19	51041037	51041267	19chr19	53238171	53238451	17chr19	54296277	54296520	19chr19	5680396	5680817	19chr19	58330921	58331187	21chr20	1757833	1758178	22chr20	23331142	23331449	16chr20	24973347	24973570	18chr20	43588935	43589216	18chr20	52532334	52532635	18chr20	5931058	5931349	18chr20	61640455	61640732	19chr2	10183990	10184239	18chr2	128458891	128459212	16chr2	153191437	153191790	16chr2	177134058	177134313	16chr2	202316114	202316384	17chr2	219712455	219712644	21chr22	20748210	20748557	19chr22	29168514	29168764	15chr2	231712666	231712913	17chr22	35767960	35768222	19chr22	36635528	36635797	18chr2	241505175	241505468	13chr2	241543800	241544097	18chr2	27255645	27255886	18chr2	27545783	27546102	14chr2	27603557	27603811	20chr2	27906445	27906668	20chr2	27994412	27994777	19chr2	46768949	46769227	15chr2	48647876	48648117	18chr2	54799488	54799707	18chr2	64994893	64995226	17chr2	70336005	70336236	19chr2	73964483	73964726	20chr2	8442171	8442450	18chr2	96782743	96783071	20chr2	97778662	97778974	23chr2	99797476	99797686	16chr2	99952829	99953142	16chr3	112395698	112395913	17chr3	128336523	128336839	21chr3	13521392	13521751	16chr3	136471145	136471426	20chr3	184053480	184053774	16chr3	196756563	196756906	18chr3	44574399	44574606	18chr3	47517397	47517617	19chr3	48342857	48343110	21chr3	49377521	49377846	16chr3	50263577	50263811	18chr3	52035578	52035798	20chr4	110736473	110736774	17chr4	129438953	129439212	17chr4	25378734	25379109	16chr4	26859039	26859468	20chr4	38321501	38321781	18chr4	39183725	39184075	16chr4	95372860	95373229	16chr5	138677335	138677610	17chr5	140904387	140904609	18chr5	142149771	142150022	17chr5	149924305	149924581	20chr5	177707058	177707240	15chr5	7869082	7869317	16chr6	110012223	110012549	15chr6	126307458	126307776	16chr6	15299828	15300135	18chr6	157695757	157696008	19chr6	159420821	159421105	18chr6	16771247	16771480	17chr6	30070918	30071101	16chr6	30796287	30796538	18chr6	31628394	31628723	17chr6	31704011	31704377	16chr6	31869830	31870154	17chr6	37400743	37401043	16chr6	42858445	42858763	13chr6	44140456	44140756	20chr6	73121975	73122182	19chr7	100609496	100609729	18chr7	102212929	102213275	17chr7	102715168	102715682	17chr7	128337393	128337635	17chr7	135661918	135662287	19chr7	150118171	150118396	19chr7	39988965	39989239	16chr7	44613436	44613710	18chr7	5603247	5603501	17chr7	64023288	64023641	17chr7	74071660	74071981	19chr7	77166033	77166290	19chr7	92861451	92861727	20chr8	11593897	11594172	17chr8	11720086	11720339	18chr8	120844930	120845222	16chr8	131054551	131054868	15chr8	144717992	144718281	17chr8	145051241	145051543	14chr8	22431927	22432139	20chr8	27172905	27173097	23chr8	64081007	64081320	14chr8	67525659	67525860	19chr9	115248820	115249047	17chr9	137335241	137335475	19chr9	138853103	138853426	19chr9	71199409	71199655	21chr9	98224890	98225150	23chrX	23801017	23801364	19chrX	48815398	48815801	16chr10	12238002	12238309	15chr10	130010259	130010469	19chr10	134385479	134385756	21chr10	43633748	43634039	17chr10	49487153	49487496	18chr10	49864347	49864597	17chr10	72163707	72163943	13chr10	73078746	73079092	15chr10	75639427	75639674	16chr10	89264405	89264731	16chr10	93058073	93058357	14chr10	97054954	97055270	18chr1	1051477	1051728	18chr11	107328445	107328660	17chr11	119076516	119076838	14chr1	112050865	112051204	19chr11	124823746	124824089	19chr1	118472133	118472404	15chr11	1874122	1874356	18chr11	2554038	2554275	16chr1	145455371	145455737	18chr11	45864712	45865029	16chr1	152020552	152020827	20chr1	155225155	155225439	12chr1	156695604	156695853	19chr11	58938937	58939209	19chr1	160983539	160983896	16chr11	62473593	62473906	15chr11	64068548	64068751	19chr11	64901936	64902202	19chr11	65547461	65547723	17chr11	67030227	67030434	16chr1	169863180	169863516	15chr11	73675392	73675718	18chr11	74959774	74959965	14chr11	75863243	75863523	17chr11	77899595	77899906	16chr1	181159081	181159284	16chr1	182931751	182931994	17chr1	192775055	192775293	15chr11	9406040	9406288	18chr11	9634729	9635026	18chr11	9635603	9635971	20chr1	203242291	203242527	16chr1	205304817	205305008	17chr1	208042463	208042681	19chr1	20987837	20988124	20chr12	108154815	108155083	19chr12	109125269	109125562	17chr12	110318185	110318488	18chr12	110869223	110869442	18chr1	211687354	211687609	19chr12	120666157	120666407	16chr12	122232887	122233108	18chr12	123344828	123345070	16chr1	212740479	212740749	14chr12	13069172	13069429	16chr12	133757921	133758148	15chr12	1905293	1905546	18chr1	222763143	222763409	17chr1	22351833	22352130	20chr12	25347990	25348333	17chr12	31226925	31227267	18chr1	231473560	231473924	20chr1	235093173	235093493	17chr1	24117418	24117722	14chr12	4430213	4430499	17chr12	49208443	49208684	18chr12	52557777	52558009	14chr12	53343574	53343842	15chr1	25566324	25566649	15chr12	58026839	58027128	15chr12	83079536	83079812	15chr12	89746295	89746630	18chr1	29208650	29208894	18chr12	94853615	94853868	17chr12	96589337	96589568	15chr13	111567564	111567798	20chr1	31769520	31769862	15chr13	34253412	34253766	18chr13	80917006	80917318	18chr1	38157855	38158236	19chr1	39339026	39339334	17chr14	102430659	102431049	19chr14	21979428	21979673	15chr14	32406974	32407219	17chr14	34512942	34513274	14chr1	43637806	43638114	18chr14	50087237	50087524	17chr14	71067214	71067441	15chr14	74195473	74195715	17chr14	74416796	74417114	16chr14	81636937	81637178	13chr14	90849600	90849907	15chr15	43398189	43398499	16chr15	43802966	43803252	16chr1	54955087	54955340	19chr15	72523567	72523787	20chr15	72529800	72530057	15chr15	78423724	78423981	16chr15	90944146	90944394	16chr15	91429305	91429523	18chr16	11351146	11351436	15chr16	27190880	27191090	14chr16	30076508	30076881	15chr16	30441457	30441697	16chr16	31884970	31885231	17chr16	67011623	67011864	18chr16	75267824	75268076	17chr16	77270012	77270243	18chr16	85279323	85279566	17chr16	85646689	85647028	20chr17	18585517	18585809	16chr17	25572338	25572598	17chr17	27279276	27279555	19chr17	29151584	29151942	18chr17	33469216	33469460	17chr17	34838880	34839094	16chr17	34957837	34958173	22chr17	37607415	37607681	17chr17	40306963	40307227	17chr17	43354633	43354886	19chr17	44271244	44271673	17chr17	45078567	45078786	18chr17	48217335	48217580	17chr17	49027759	49028021	17chr17	59489814	59490076	16chr17	66243812	66244193	17chr17	66288068	66288479	17chr17	71228297	71228645	14chr17	7123078	7123343	18chr17	73105857	73106187	15chr18	12991062	12991316	14chr18	20811294	20811686	19chr18	47340052	47340329	17chr1	84972196	84972443	12chr18	72264927	72265181	14chr18	8609198	8609501	16chr19	11457043	11457464	16chr19	12868422	12868669	15chr19	13204678	13204898	17chr19	14142996	14143290	14chr19	14192099	14192465	21chr19	1445214	1445456	17chr19	19285899	19286106	16chr19	2543562	2543827	16chr19	30251020	30251299	16chr19	36422426	36422736	18chr19	41312740	41313086	18chr19	45303764	45304000	16chr19	46696478	46696710	14chr19	4791209	4791574	15chr19	48246844	48247109	19chr19	50145128	50145351	17chr19	50183997	50184210	16chr19	53400767	53401015	14chr19	54605887	54606228	16chr19	55987739	55987968	16chr19	57998905	57999239	17chr19	5804071	5804276	19chr19	584434	584689	18chr19	58873733	58874058	14chr19	59031092	59031343	19chr19	8070535	8070856	16chr19	9731761	9732132	17chr20	1373673	1373962	16chr20	2632971	2633290	19chr20	37274843	37275087	15chr20	37678747	37678938	15chr20	46292581	46292862	17chr20	48545262	48545525	19chr20	48770148	48770563	12chr20	49604295	49604511	14chr20	57607198	57607592	19chr20	62496405	62496714	17chr20	814187	814439	16chr2	122407491	122407812	17chr2	132431593	132431855	17chr2	170655293	170655589	15chr2	20779066	20779297	19chr2	220394942	220395215	16chr22	23624030	23624260	17chr2	228336724	228336925	18chr22	29137634	29137948	18chr22	31607968	31608287	14chr2	232571688	232572031	19chr2	232646203	232646497	15chr2	239165241	239165520	17chr22	41799866	41800074	18chr2	242448039	242448274	15chr2	24583227	24583616	17chr22	50468757	50469022	15chr2	28789644	28789871	18chr2	45870839	45871145	18chr2	46844151	46844456	16chr2	47550307	47550509	17chr2	54342813	54343138	16chr2	9983385	9983785	18chr3	10018738	10018966	17chr3	108308192	108308535	18chr3	113557498	113557790	19chr3	121553736	121554056	15chr3	13689824	13690104	18chr3	139108348	139108651	17chr3	177063480	177063737	17chr3	195681327	195681556	18chr3	196411813	196412117	17chr3	32726549	32726847	17chr3	42922536	42922738	17chr3	47020996	47021296	18chr3	48699189	48699375	15chr3	49709176	49709431	18chr3	58477571	58477815	16chr4	146540386	146540712	17chr4	1722844	1723135	17chr4	40623781	40624015	17chr4	56212165	56212631	19chr4	57410744	57411056	18chr5	110074540	110074869	14chr5	145826480	145826832	17chr5	14809896	14810107	19chr5	150827015	150827293	19chr5	153784796	153785059	20chr5	154320468	154320763	17chr5	43040431	43040666	18chr5	55008012	55008424	18chr5	67730053	67730417	19chr5	72251623	72251949	15chr6	122792832	122793142	16chr6	130031229	130031569	18chr6	147523621	147524024	16chr6	149886714	149887053	18chr6	21593792	21594085	17chr6	2634719	2635050	18chr6	27356621	27356828	16chr6	27740270	27740542	14chr6	27763606	27763864	17chr6	2842010	2842295	15chr6	41336697	41336947	15chr6	42420844	42421103	20chr6	43484561	43484893	18chr6	43742572	43742772	16chr6	58287615	58287880	15chr6	88032149	88032340	19chr6	88411870	88412299	16chr6	99841895	99842192	19chr7	100781759	100782067	18chr7	100861238	100861489	16chr7	100895441	100895749	14chr7	105683297	105683558	17chr7	1126541	1126831	16chr7	127291879	127292203	17chr7	134966318	134966500	18chr7	139184796	139185042	18chr7	148844345	148844667	19chr7	148959065	148959386	17chr7	149535333	149535592	18chr7	20278357	20278686	20chr7	47514740	47515016	13chr7	4793557	4793779	16chr7	56032004	56032369	19chr7	64363481	64363747	17chr7	66594125	66594424	19chr7	73140097	73140336	16chr8	124286405	124286634	16chr8	143484565	143484786	16chr8	143820816	143821112	17chr8	1703806	1704135	15chr8	41559306	41559568	20chr8	42010100	42010422	18chr8	8860259	8860539	20chr8	90996210	90996568	20chr9	112083318	112083623	16chr9	123883875	123884174	16chr9	132499942	132500256	19chr9	132815977	132816274	18chr9	134955204	134955386	15chr9	138859469	138859732	18chr9	139305020	139305263	14chr9	139760696	139760972	17chr9	139940997	139941249	17chr9	95922278	95922529	17chrX	104166921	104167114	18chrX	129039928	129040200	15chrX	48748984	48749270	17chrX	70331357	70331623	14chr10	102193587	102193812	16chr10	118976189	118976416	16chr10	12110624	12110948	17chr10	126377524	126377787	17chr10	44101734	44101996	18chr10	73620237	73620448	14chr10	75385697	75385989	16chr10	75503936	75504232	16chr10	80733191	80733508	21chr10	91461046	91461386	16chr10	95256195	95256479	15chr10	97453741	97454056	16chr10	99160954	99161157	15chr1	10092623	10092989	17chr1	10270275	10270597	18chr11	10427787	10427990	16chr11	109817041	109817243	18chr11	113644384	113644666	15chr11	123940144	123940358	16chr1	112912311	112912555	17chr11	129620769	129621006	18chr11	16627814	16628092	18chr1	11845204	11845491	17chr11	2923345	2923583	16chr1	144932009	144932369	15chr11	47395479	47395718	19chr11	48131474	48131722	14chr1	155140327	155140613	17chr1	155231953	155232349	17chr1	156052232	156052451	12chr1	156100027	156100352	15chr11	59521533	59521751	20chr1	159880287	159880534	17chr11	61602066	61602342	17chr11	64622465	64622713	19chr11	64627051	64627272	18chr11	64661765	64662011	14chr11	65488171	65488357	16chr11	65990187	65990440	15chr11	67007409	67007658	15chr11	67033666	67033906	16chr11	67275489	67275846	15chr11	67350992	67351350	17chr11	67396775	67397174	16chr11	69065976	69066270	20chr11	72145509	72145781	17chr1	17231490	17231885	21chr11	75417351	75417595	15chr1	183559621	183559829	16chr11	85565885	85566255	17chr11	95657097	95657380	13chr1	205454642	205454963	14chr12	101801734	101801986	15chr12	105629915	105630194	16chr12	123011631	123011920	14chr12	26985975	26986247	11chr1	234743841	234744106	16chr1	24136288	24136506	14chr1	2457589	2457781	18chr12	49351132	49351414	17chr12	49628224	49628500	18chr12	49736251	49736573	17chr12	510555	510857	19chr12	53553240	53553447	15chr12	53902407	53902619	16chr12	54762483	54762734	19chr12	56320608	56321041	18chr12	57030498	57030795	17chr1	26221965	26222183	16chr12	69139722	69140058	15chr12	7052979	7053303	19chr12	75728139	75728451	20chr12	76371779	76371992	20chr1	27718853	27719117	17chr13	30706743	30706979	16chr13	49323884	49324206	16chr13	51486166	51486423	17chr1	37939977	37940265	18chr13	80055314	80055662	17chr14	20881445	20881773	16chr14	21077511	21077785	18chr14	21457990	21458230	15chr14	23426156	23426498	19chr14	23770681	23770998	16chr14	24701604	24701993	17chr1	43311986	43312302	17chr14	35591062	35591319	16chr14	45604842	45605151	18chr1	45139938	45140283	17chr1	46645225	46645470	19chr14	71786877	71787163	17chr1	47184775	47185118	18chr14	73393060	73393310	16chr1	47903397	47903697	15chr14	96027369	96027583	17chr14	96829557	96829815	16chr1	51762559	51762774	18chr15	22436132	22436341	19chr15	41099575	41099882	18chr15	45571305	45571596	18chr1	55680924	55681271	16chr15	64189037	64189265	18chr15	67834796	67835083	18chr15	69351532	69351790	17chr15	69706412	69706754	12chr15	74419954	74420139	18chr15	74674799	74674995	17chr15	75315781	75316021	15chr15	75660201	75660535	17chr15	78441373	78441662	17chr15	85197400	85197694	19chr15	89901865	89902201	18chr15	90686451	90686744	17chr15	91260376	91260729	15chr1	59165596	59165878	15chr15	93461158	93461432	15chr16	2097762	2098073	16chr16	25269511	25269762	17chr16	2551243	2551549	15chr16	28289044	28289299	15chr16	30709353	30709574	16chr16	30798410	30798641	13chr16	30933771	30934014	15chr16	47071758	47071957	14chr16	48387576	48387793	19chr16	56390361	56390647	15chr16	57844227	57844476	18chr16	67225906	67226163	17chr16	69458474	69458675	17chr16	84538340	84538582	15chr16	86597501	86597724	17chr17	17184475	17184791	15chr17	25659893	25660158	18chr17	27070836	27071124	17chr17	27546434	27546746	19chr17	30334207	30334479	17chr17	36762660	36762893	17chr17	37009901	37010219	16chr17	40761482	40761812	17chr17	43505498	43505758	19chr17	48860607	48860810	12chr17	55320572	55320759	17chr17	57642763	57643025	15chr17	61045861	61046097	19chr17	61556263	61556554	16chr17	66097697	66097936	17chr17	66508411	66508705	16chr17	7155661	7155955	15chr17	73936935	73937349	20chr17	74260871	74261116	19chr17	76108753	76108960	14chr17	77005757	77006043	16chr17	77813431	77813862	16chr17	78400737	78401012	16chr17	78830337	78830551	18chr18	28786747	28787088	16chr18	3448227	3448510	17chr18	55288982	55289213	19chr18	66382235	66382505	13chr19	11455139	11455392	17chr19	11849720	11850063	17chr19	12404676	12404867	15chr19	14800711	14800965	16chr19	15490499	15490786	13chr19	18263320	18263634	15chr19	1864809	1865041	15chr19	19887167	19887466	16chr19	35264007	35264256	16chr19	38270022	38270426	15chr19	44763947	44764210	17chr19	45256652	45256829	17chr19	45348012	45348280	17chr19	45542179	45542445	15chr19	46366436	46366667	14chr19	50093494	50093789	13chr19	50175577	50175827	15chr19	5048329	5048541	15chr19	50918047	50918259	16chr19	53935059	53935411	15chr19	58144310	58144658	16chr19	6516953	6517199	17chr19	7459850	7460101	16chr20	23402013	23402291	18chr20	2505061	2505293	16chr20	32580811	32581162	18chr20	33680559	33680872	17chr20	37554635	37554959	14chr20	44600454	44600752	17chr20	49077308	49077569	17chr20	60876962	60877210	16chr2	102972107	102972374	18chr2	11272765	11272998	15chr2	113238642	113238958	16chr2	131090143	131090461	15chr21	34755448	34755837	16chr21	44313180	44313520	15chr2	148778320	148778566	17chr2	158695035	158695286	14chr2	160143278	160143604	15chr2	18741843	18742155	11chr2	191513320	191513651	14chr2	198317954	198318358	15chr22	19705526	19705824	17chr2	223836509	223836701	17chr22	25843831	25844086	13chr22	38448333	38448625	17chr22	42486830	42487144	16chr2	242549579	242549852	16chr22	45664896	45665138	14chr22	50311828	50312142	16chr22	50356008	50356369	15chr2	26214515	26214733	14chr2	27008541	27008859	17chr2	27851737	27851932	17chr2	32502747	32503042	16chr2	38829931	38830230	15chr2	39664306	39664600	17chr2	72110128	72110388	15chr2	85934451	85934684	16chr2	95831284	95831550	13chr2	98423288	98423477	16chr2	9843892	9844132	14chr3	123303968	123304282	18chr3	125803005	125803294	16chr3	126630295	126630573	18chr3	130612646	130612894	16chr3	140911201	140911392	16chr3	14166273	14166573	14chr3	152094555	152094799	13chr3	15468912	15469158	15chr3	16592353	16592622	15chr3	191047017	191047274	20chr3	193807405	193807647	17chr3	39194261	39194552	18chr3	40350973	40351271	15chr3	40566292	40566516	16chr3	49058787	49059042	16chr3	49840050	49840305	17chr3	58163452	58163740	18chr3	8811079	8811326	18chr3	98312372	98312744	16chr4	140187833	140188128	19chr4	154265685	154265963	18chr4	178363407	178363693	15chr4	40311312	40311609	18chr4	41218442	41218689	14chr4	57333559	57333868	18chr4	79698105	79698346	13chr5	10353549	10353898	15chr5	112043033	112043417	15chr5	122180953	122181275	17chr5	130970963	130971269	15chr5	131722307	131722560	16chr5	132165684	132165948	16chr5	138739596	138739936	15chr5	139944161	139944499	18chr5	148747146	148747377	13chr5	157286038	157286303	17chr5	169758247	169758536	15chr5	173315148	173315417	13chr5	176087841	176088074	15chr5	176882370	176882680	18chr5	39424911	39425302	18chr5	40755998	40756238	16chr5	43121417	43121711	18chr5	56469625	56469907	19chr5	68530524	68530779	15chr5	71604231	71604509	17chr6	105907051	105907287	15chr6	117002009	117002228	13chr6	157936093	157936331	17chr6	158461379	158461669	19chr6	159290959	159291162	16chr6	31334706	31335000	17chr6	33359014	33359290	14chr6	33422170	33422461	17chr6	34577420	34577682	18chr6	37027105	37027399	20chr6	42989174	42989472	17chr6	44119658	44119862	16chr6	44189365	44189603	15chr6	44225146	44225610	18chr6	47015296	47015526	14chr6	75953527	75953692	13chr6	80714095	80714447	17chr6	83775457	83775692	16chr7	100157482	100157667	17chr7	100449668	100449954	13chr7	100887999	100888471	20chr7	108210069	108210352	18chr7	112090897	112091092	12chr7	1209532	1209769	16chr7	128695028	128695322	16chr7	142912264	142912422	19chr7	1452478	1452778	15chr7	152161084	152161449	17chr7	154705399	154705718	14chr7	157129536	157129775	16chr7	21466877	21467193	17chr7	35577100	35577345	19chr7	44678773	44679051	18chr7	5565975	5566281	17chr7	73269877	73270185	19chr7	73482247	73482457	19chr7	73620956	73621248	17chr7	7606492	7606700	19chr7	76751572	76751865	17chr7	90893554	90893747	18chr7	99102713	99102961	16chr8	142441680	142441864	16chr8	144465779	144466032	15chr8	144766481	144766697	16chr8	21994917	21995166	19chr8	22462075	22462313	16chr8	37962903	37963237	17chr8	42128697	42128912	15chr8	8923322	8923560	15chr8	97247672	97247926	15chr9	111882248	111882511	15chr9	127631251	127631552	15chr9	130007394	130007735	16chr9	131769079	131769347	18chr9	132096460	132096813	18chr9	135897752	135897984	16chr9	136022975	136023228	15chr9	137035946	137036143	16chr9	138304482	138304690	19chr9	5832948	5833246	15chr9	69065303	69065527	15chr9	86322764	86323043	19chrX	108779959	108780259	16chrX	118826997	118827348	16chrX	123094062	123094396	16chrX	153626391	153626814	13chrX	44732093	44732361	18chrX	70503299	70503629	16chr10	101989287	101989524	12chr10	104238772	104238984	19chr10	30287327	30287549	15chr10	43048205	43048530	16chr10	43892673	43892968	18chr10	5665132	5665387	18chr10	69644126	69644411	19chr10	82422520	82422785	16chr10	88137035	88137236	17chr10	97321123	97321424	16chr1	109234767	109235025	17chr11	102188014	102188249	15chr11	116658631	116658889	15chr11	118122795	118123154	14chr11	118758460	118758734	17chr1	113249969	113250253	16chr11	17752646	17752875	17chr1	12112106	12112376	20chr11	313871	314166	14chr11	34072993	34073293	16chr11	44117053	44117361	18chr1	150586047	150586314	15chr1	154130255	154130538	16chr1	155034044	155034251	15chr1	155063954	155064159	13chr11	57103300	57103516	15chr11	58903428	58903650	15chr11	61739280	61739537	18chr11	62328104	62328428	16chr11	62379966	62380174	14chr11	62528971	62529174	15chr11	64691525	64691768	14chr11	64764355	64764661	16chr11	6640518	6640778	17chr11	67150077	67150278	15chr1	168147950	168148299	16chr11	728037	728269	15chr1	17452299	17452543	16chr11	76494018	76494303	18chr1	178511552	178511938	15chr1	183439290	183439559	14chr1	183604707	183605041	13chr11	8985829	8986157	16chr1	19239598	19239827	14chr1	193028495	193028733	16chr11	95976467	95976685	15chr11	96076387	96076673	15chr1	201482131	201482341	15chr1	202938169	202938416	12chr1	206643615	206643915	18chr1	208357743	208357971	17chr12	102455766	102456057	16chr12	111264505	111264732	20chr12	112037288	112037565	16chr12	120638958	120639223	15chr1	222886531	222886730	15chr1	224544433	224544639	15chr1	226163749	226163945	18chr1	234738536	234738767	13chr1	242011204	242011593	18chr12	42538579	42538795	16chr1	246859765	246860068	18chr1	247553485	247553771	17chr12	49284468	49284729	12chr12	50339353	50339616	13chr12	50898528	50898788	17chr12	51318156	51318439	16chr12	52386973	52387171	15chr12	53661848	53662268	16chr12	53715311	53715511	16chr12	54754677	54754904	16chr12	6641703	6642017	15chr1	28844595	28844866	15chr12	92945680	92945997	11chr12	95497005	95497254	14chr1	31274682	31274895	15chr1	31867505	31867810	17chr13	22178223	22178501	15chr13	31377076	31377300	14chr1	33238628	33238880	15chr1	33352229	33352441	19chr13	41837594	41837777	15chr1	38156124	38156403	17chr1	38412588	38412863	15chr13	99097608	99097912	13chr1	40075317	40075555	17chr14	100149127	100149396	16chr14	23235713	23236004	13chr14	23398795	23399033	18chr14	23538164	23538359	14chr1	43147745	43148039	16chr14	50234649	50234953	17chr1	45769631	45769879	17chr14	58862505	58862770	18chr1	45987417	45987643	17chr14	74252610	74252859	17chr14	77279174	77279438	17chr1	47902196	47902466	20chr14	93651131	93651470	14chr15	26327322	26327646	15chr15	40453019	40453290	17chr15	42103643	42103894	14chr15	42565462	42565800	16chr15	44038537	44038728	14chr15	67095509	67095775	15chr15	78832533	78832892	15chr15	85278279	85278618	17chr15	91446388	91446634	15chr16	11057981	11058195	18chr16	18995080	18995341	15chr16	23516120	23516345	17chr16	25026585	25026879	16chr16	2509948	2510207	13chr16	2802158	2802456	15chr16	28503308	28503583	18chr16	31470585	31470787	15chr16	420504	420704	16chr16	48657163	48657467	16chr16	56459380	56459624	14chr16	56763941	56764207	15chr16	639106	639449	12chr16	639710	639952	14chr1	6639925	6640203	14chr16	68562612	68562839	14chr16	70473110	70473388	18chr16	70834982	70835267	16chr16	81437193	81437466	18chr16	87422042	87422382	13chr16	87811510	87811737	13chr16	89723974	89724284	17chr17	12921409	12921729	13chr17	1477775	1478016	16chr17	1620769	1621024	14chr17	17140537	17140992	15chr17	17739509	17739808	18chr17	18086539	18086843	17chr17	26925919	26926234	15chr17	33307305	33307529	15chr17	34091125	34091429	19chr17	34891188	34891487	15chr17	36886262	36886578	14chr17	37309129	37309460	17chr17	38518947	38519158	16chr17	39822169	39822370	13chr17	39823295	39823561	18chr17	42357088	42357411	16chr17	42580920	42581173	16chr17	42610947	42611215	16chr17	43138366	43138630	14chr17	46671305	46671522	20chr17	53828352	53828604	16chr17	56415349	56415628	13chr17	58220252	58220532	15chr17	66168547	66168812	17chr17	73452510	73452779	17chr17	73511888	73512117	17chr17	78234017	78234293	14chr18	21656208	21656474	14chr18	3037439	3037686	13chr18	3247638	3247908	15chr18	33196905	33197130	18chr18	36520818	36521187	18chr18	39534995	39535298	15chr18	43271241	43271559	15chr18	51795635	51795897	15chr18	57504828	57505064	17chr1	85773883	85774139	15chr1	87169991	87170314	18chr19	11201049	11201399	16chr19	12606068	12606395	14chr19	12907379	12907591	18chr19	14184188	14184517	17chr19	14328137	14328454	17chr19	18049103	18049325	18chr19	2069753	2069979	14chr19	2962150	2962326	14chr19	3163340	3163620	16chr1	93645985	93646233	12chr19	3985323	3985597	18chr19	41029262	41029469	15chr19	44439334	44439659	16chr19	44598295	44598563	18chr19	45273926	45274177	17chr19	45458310	45458602	15chr19	46087915	46088254	16chr19	47582701	47582920	13chr19	47939943	47940197	15chr19	49999463	49999795	17chr19	53445671	53445907	17chr19	58740108	58740358	14chr20	1484371	1484539	14chr20	21106468	21106822	17chr20	23342349	23342609	15chr20	3026448	3026784	18chr20	335182	335394	14chr20	35515611	35515926	15chr20	3776424	3776744	16chr20	39597433	39597730	14chr20	4065949	4066223	14chr20	44065584	44065820	14chr20	4741261	4741585	18chr20	52486357	52486574	18chr20	52543124	52543357	17chr20	61454158	61454390	14chr2	128615551	128615908	16chr2	133015140	133015291	18chr2	135675643	135675935	15chr2	136633883	136634198	14chr21	45567072	45567361	18chr2	171729751	171730028	16chr2	172272620	172272845	15chr2	20189756	20190076	14chr2	208195612	208195871	17chr2	216176609	216176851	13chr2	219773672	219773990	17chr22	20144535	20144814	17chr2	220163495	220163725	17chr22	22292472	22292798	16chr22	24093243	24093488	14chr22	24921425	24921643	16chr22	27041210	27041529	18chr22	29784450	29784752	14chr22	30752680	30753069	17chr2	231851863	231852200	16chr22	37638428	37638778	17chr2	241075648	241075885	14chr22	41488327	41488716	15chr22	42078328	42078575	14chr2	26101334	26101671	19chr2	27309334	27309559	16chr2	27805720	27806093	12chr2	43520978	43521225	13chr2	7005837	7006113	16chr2	85134071	85134501	15chr2	97175148	97175420	15chr2	97220135	97220373	17chr2	98262302	98262621	18chr3	113464044	113464323	15chr3	126191148	126191443	16chr3	128997453	128997796	18chr3	158390350	158390648	16chr3	167452566	167452894	16chr3	184429694	184430003	15chr3	190040102	190040393	18chr3	23958372	23958746	16chr3	28390421	28390686	18chr3	45079237	45079488	18chr3	47462601	47462817	14chr3	48506891	48507351	14chr3	48754616	48754822	17chr3	49977267	49977619	15chr3	50653455	50653651	16chr4	1037373	1037665	13chr4	104020922	104021192	16chr4	13655798	13656039	17chr4	15683246	15683521	16chr4	38162555	38162831	19chr4	41937002	41937270	12chr4	71705437	71705765	15chr4	77870703	77871011	13chr4	926070	926315	17chr5	133304282	133304541	17chr5	133839041	133839360	16chr5	137090116	137090415	16chr5	139012951	139013133	15chr5	1645089	1645286	17chr5	173959579	173959843	18chr5	176817552	176817773	15chr5	177591217	177591462	19chr5	90576308	90576556	18chr6	143247907	143248165	19chr6	151711575	151711868	18chr6	169284746	169284962	13chr6	18155502	18155809	16chr6	26026561	26026822	15chr6	28129432	28129679	16chr6	30312806	30313070	15chr6	31633496	31633781	17chr6	3259064	3259318	16chr6	33378053	33378346	18chr6	37659831	37660054	15chr6	39270990	39271219	16chr6	43021537	43021788	15chr6	43737265	43737510	19chr6	91189038	91189290	17chr7	100080884	100081195	17chr7	102031201	102031438	13chr7	120627767	120628008	16chr7	134117540	134117731	14chr7	148224774	148224993	14chr7	150715224	150715513	15chr7	150821095	150821316	15chr7	150924331	150924617	14chr7	1513724	1513928	14chr7	36406757	36406994	16chr7	48124624	48124885	16chr7	5534312	5534561	14chr7	56142035	56142301	14chr7	5938191	5938454	17chr7	73149048	73149315	16chr7	75932779	75933092	17chr8	103580219	103580486	17chr8	103826922	103827110	15chr8	105677573	105677816	17chr8	127888875	127889154	17chr8	141601541	141601767	14chr8	144536827	144537049	14chr8	22225310	22225613	16chr8	22932211	22932479	13chr8	25316421	25316692	13chr8	28558935	28559155	14chr8	29952798	29953054	14chr8	30413373	30413730	17chr8	38033906	38034220	15chr8	54857532	54857814	15chr8	67036199	67036551	18chr8	91013043	91013317	15chr9	104295941	104296326	15chr9	131218173	131218430	15chr9	132539491	132539818	17chr9	133873775	133874023	18chr9	139014745	139015055	15chr9	7799614	7799927	14chrX	133941004	133941319	14chrX	1365748	1365927	18chrX	18443410	18443686	12chrX	38186765	38187001	19chrX	44402038	44402444	16chrX	47441603	47441906	14chrX	48776173	48776442	15chrX	48980103	48980381	15chrX	52963913	52964228	15chrX	54466609	54466824	17chrX	73163925	73164181	19chrX	77359317	77359667	17chr10	102321795	102322041	16chr10	103600484	103600703	16chr10	104210079	104210493	18chr10	105678228	105678447	16chr10	112114101	112114386	16chr10	112431889	112432137	17chr10	114206556	114206896	15chr10	121652033	121652297	14chr10	126077830	126078093	16chr10	129948335	129948586	16chr10	14647341	14647580	16chr10	5855435	5855733	16chr10	73533211	73533454	14chr10	75645905	75646164	17chr10	79471221	79471453	16chr10	81077483	81077745	12chr10	88963234	88963487	16chr10	91020186	91020424	16chr10	99185765	99186015	15chr1	10571040	10571252	16chr1	109655361	109655546	14chr11	118016086	118016374	13chr11	118436560	118436854	18chr11	123612259	123612538	15chr11	124746201	124746485	16chr1	113423609	113423832	17chr1	115681615	115681860	10chr1	116961084	116961337	18chr1	117909923	117910204	15chr11	18034448	18034762	15chr1	11866023	11866290	19chr11	36171308	36171582	16chr11	36615876	36616146	15chr11	3876125	3876400	19chr1	145589247	145589527	15chr11	45826444	45826765	13chr11	45921347	45921596	12chr11	46391000	46391292	14chr1	149860005	149860205	17chr1	151882032	151882191	16chr1	154377644	154377908	14chr1	154955525	154955805	16chr1	156195246	156195499	15chr11	61666659	61666853	16chr11	63993532	63993864	15chr11	65756471	65756758	16chr11	66247727	66248064	17chr11	66659681	66659922	16chr1	16693425	16693648	17chr11	67374165	67374450	14chr11	68024978	68025215	15chr11	72436326	72436578	13chr11	72504599	72505045	15chr11	74733999	74734192	17chr11	76758725	76759008	16chr1	179334938	179335196	15chr11	92930520	92930762	16chr1	203764549	203764820	16chr12	109058606	109058848	13chr12	112856487	112856755	14chr12	119882359	119882553	15chr12	123868192	123868456	14chr12	123942545	123942812	15chr1	226312869	226313159	15chr1	226850502	226850898	18chr12	27676726	27677094	14chr1	23730466	23730723	15chr12	39298684	39298915	15chr12	3982485	3982739	13chr1	24397375	24397590	15chr1	25015060	25015372	16chr12	53493155	53493458	17chr12	53693325	53693659	16chr1	25558880	25559169	14chr12	56390840	56391139	16chr12	57395266	57395515	15chr12	58299165	58299390	15chr1	26146309	26146588	14chr12	7055441	7055817	15chr12	7074259	7074498	17chr1	27721916	27722144	13chr1	29211483	29211708	13chr13	22051184	22051425	16chr1	32405389	32405697	17chr13	27936204	27936422	14chr1	32986091	32986324	15chr13	40516962	40517174	16chr13	60970370	60970623	11chr14	102197564	102197875	15chr14	102783200	102783448	18chr14	103542007	103542248	14chr14	105556868	105557139	14chr14	21439624	21439816	15chr14	21924153	21924417	10chr14	23938639	23938983	17chr14	24058264	24058538	15chr14	31677258	31677520	14chr14	50328793	50329093	18chr14	50787856	50788135	17chr14	50999227	50999454	12chr14	55239971	55240257	16chr14	55568922	55569224	20chr1	46502768	46503009	16chr14	74111481	74111768	14chr14	74225073	74225317	17chr14	91885138	91885392	15chr15	30113643	30113894	15chr15	34875692	34875969	16chr15	40337358	40337597	14chr15	41408648	41408987	16chr15	45018876	45019142	14chr15	59592569	59592847	17chr15	75063447	75063694	14chr15	75242794	75243006	17chr15	76483119	76483376	14chr15	85956747	85956987	15chr15	89560823	89561130	16chr15	89959589	89959807	16chr1	59281998	59282205	15chr15	93425533	93425865	16chr16	2033959	2034269	16chr16	2073172	2073402	17chr16	2203608	2203837	16chr16	46406524	46406629	13chr16	47177346	47177613	12chr16	55499056	55499288	14chr16	56735982	56736226	13chr16	57553701	57553977	16chr16	58231974	58232226	15chr1	67896035	67896352	15chr16	82203624	82203980	15chr16	85855098	85855319	17chr16	87526525	87526796	15chr16	87943021	87943250	18chr16	88522475	88522777	15chr17	1018399	1018630	13chr17	16189296	16189624	16chr17	19896124	19896419	14chr17	21183397	21183628	14chr17	21191219	21191454	17chr17	37027765	37028096	12chr17	38375332	38375547	14chr17	38716553	38716789	17chr17	40273291	40273504	13chr17	4458650	4458888	11chr17	47492188	47492538	15chr17	57784813	57785153	17chr17	7142952	7143215	18chr17	72350209	72350466	15chr17	73760629	73760955	16chr17	74178343	74178551	18chr17	7482690	7483012	16chr17	79071251	79071504	16chr17	80111814	80112007	16chr17	80309744	80309972	16chr18	19284638	19284913	14chr18	32559103	32559417	17chr18	77139039	77139343	14chr1	8908984	8909273	17chr19	12163548	12163842	15chr19	16295956	16296224	18chr19	16606964	16607170	15chr19	1853370	1853605	15chr19	1885393	1885656	15chr19	18942550	18942832	17chr19	2456934	2457187	13chr19	38865210	38865512	15chr19	39137927	39138322	18chr19	39881835	39882144	12chr19	41814467	41814700	13chr19	42942658	42942891	15chr19	44576083	44576395	17chr19	44716499	44716834	15chr19	4639006	4639260	12chr19	47818621	47818873	14chr1	94883579	94883826	15chr19	49087542	49087760	16chr19	49403222	49403489	14chr19	50529181	50529404	12chr19	53465941	53466251	14chr19	56028323	56028558	13chr19	57946495	57946744	12chr19	58459001	58459309	17chr19	6087905	6088188	19chr19	7197189	7197456	15chr19	7717002	7717250	18chr19	7872457	7872714	13chr19	8418884	8419142	15chr1	9927602	9927809	15chr20	35201581	35201896	17chr20	36013133	36013351	14chr20	37522506	37522691	12chr20	39318699	39318932	15chr20	39620104	39620346	17chr20	43594907	43595145	17chr20	44002515	44002780	17chr20	48785946	48786157	16chr20	48909109	48909431	17chr20	50808180	50808429	15chr2	102844246	102844438	15chr2	10442596	10442815	15chr2	114147904	114148189	17chr2	135208481	135208731	15chr21	45527028	45527356	14chr21	47563475	47563727	14chr2	172430647	172430848	17chr2	177005249	177005555	16chr2	179279445	179279744	11chr2	181971612	181971840	17chr2	202507269	202507574	14chr2	20335452	20335654	16chr2	20646622	20646905	18chr2	209119938	209120243	14chr22	18507286	18507539	15chr22	19929220	19929472	14chr2	231917621	231917847	14chr22	36727526	36727822	16chr22	37957749	37958013	13chr22	38003110	38003366	17chr22	41601099	41601418	15chr2	24307182	24307566	17chr22	45705679	45705952	16chr2	27392782	27392986	16chr2	30387952	30388160	16chr2	33824205	33824572	16chr2	3698637	3698844	18chr2	43406125	43406398	15chr2	44271402	44271630	14chr2	65780412	65780654	15chr2	71192055	71192312	16chr2	73298709	73299052	16chr2	91815039	91815415	15chr2	9796576	9796799	19chr3	100427986	100428304	16chr3	111697655	111697960	16chr3	131753794	131754057	18chr3	13503121	13503358	15chr3	15106686	15106988	16chr3	15374005	15374239	14chr3	154042144	154042439	14chr3	184529784	184530056	15chr3	185255244	185255494	18chr3	44481312	44481540	19chr3	48936236	48936570	16chr3	52007912	52008250	13chr3	52570450	52570752	12chr3	57583068	57583354	15chr3	58457084	58457359	16chr3	66271271	66271550	14chr3	67164341	67164531	18chr4	104119461	104119719	15chr4	183846530	183846777	14chr4	184104539	184104764	17chr4	1900987	1901260	17chr4	36287790	36288114	18chr4	56719669	56719951	16chr4	83821672	83822046	16chr4	95128837	95129199	16chr5	106907788	106908080	14chr5	107718062	107718314	16chr5	111756032	111756274	20chr5	114970347	114970624	15chr5	126011904	126012119	14chr5	139624132	139624313	15chr5	139737295	139737542	14chr5	139742781	139743120	14chr5	141742374	141742593	16chr5	149815664	149815932	17chr5	151031630	151031832	13chr5	159772360	159772614	16chr5	159848707	159848965	14chr5	171906039	171906283	16chr5	172261029	172261349	15chr5	173193423	173193654	18chr5	176744906	176745183	15chr5	179223176	179223427	16chr5	179247852	179248051	15chr5	32444707	32445071	13chr5	36690470	36690749	14chr5	61601603	61601901	18chr5	6633009	6633345	14chr5	78810315	78810561	15chr5	87564583	87564849	17chr6	10404718	10404961	17chr6	109761861	109762156	17chr6	112364864	112365169	17chr6	13874074	13874342	17chr6	143267428	143267738	17chr6	20877670	20877908	15chr6	27573342	27573631	15chr6	27804833	27805153	16chr6	29933818	29934141	14chr6	30594482	30594771	16chr6	30655158	30655376	15chr6	31126045	31126379	17chr6	32158067	32158274	16chr6	34205832	34206130	15chr6	36726982	36727197	16chr6	36997133	36997459	17chr6	41040135	41040484	12chr6	42694371	42694616	15chr6	99968783	99969036	12chr7	100066966	100067140	16chr7	100472437	100472683	17chr7	104563417	104563594	14chr7	1215787	1216078	13chr7	127849838	127850080	18chr7	128858453	128858698	14chr7	130794564	130794874	15chr7	131231434	131231748	15chr7	137686938	137687295	18chr7	142491048	142491422	17chr7	150705831	150706077	15chr7	157095113	157095350	13chr7	22539738	22539972	15chr7	23586631	23586861	17chr7	26707215	26707466	12chr7	44297531	44297839	17chr7	48031384	48031613	15chr7	48149810	48150059	16chr7	50700662	50700892	13chr7	54826907	54827110	15chr7	6556066	6556273	15chr7	75115507	75115760	16chr7	92462325	92462514	17chr7	99698242	99698447	18chr7	99869746	99870010	17chr8	118532789	118533117	17chr8	124054371	124054858	18chr8	124408555	124408841	14chr8	140718111	140718378	15chr8	29107509	29107731	16chr8	38243743	38244068	18chr8	42029179	42029394	15chr8	81260180	81260414	14chr8	98608104	98608355	15chr9	101553464	101553795	14chr9	115818969	115819236	15chr9	128170346	128170577	15chr9	131069526	131069792	11chr9	131182553	131182789	15chr9	132650716	132651016	17chr9	133741834	133742101	16chr9	134225451	134225737	17chr9	139652875	139653101	15chr9	139682192	139682396	17chr9	15307090	15307552	15chr9	26947050	26947369	14chr9	27529670	27529930	15chr9	32552191	32552453	14chr9	34178783	34179034	15chr9	34379374	34379688	15chr9	35079939	35080186	15chr9	37120352	37120677	16chr9	95882222	95882513	13chrX	106954277	106954517	13chrX	109018034	109018242	16chrX	14047893	14048146	18chrX	20009215	20009459	14chrX	20284969	20285220	14chrX	39719609	39719850	16chrX	47863245	47863576	13chrX	49056564	49056764	15chr10	102758904	102759217	16chr10	103540852	103541132	12chr10	103543028	103543308	13chr10	104404636	104404907	14chr10	105881427	105881740	14chr10	112116375	112116616	14chr10	119806287	119806592	15chr10	124134001	124134261	13chr10	124892573	124892790	14chr10	1463088	1463342	17chr10	15902415	15902684	14chr10	32428329	32428620	12chr10	45496257	45496494	14chr10	6443797	6444038	15chr10	64577696	64578000	15chr10	64892887	64893198	15chr10	73454370	73454606	14chr10	74056965	74057303	19chr10	74100228	74100449	14chr10	82258572	82258840	10chr10	94831579	94831887	16chr10	97989313	97989539	13chr10	98561340	98561522	17chr1	108113020	108113160	14chr1	110026586	110026935	14chr1	110163579	110163813	13chr11	107992102	107992360	14chr11	120381813	120382047	16chr11	121338756	121338986	13chr11	124670136	124670429	12chr11	125439117	125439328	19chr11	1714503	1714755	14chr11	17229399	17229703	14chr11	35684149	35684543	16chr11	45361593	45361855	14chr1	146373433	146373676	14chr11	46559432	46559652	14chr1	146644128	146644428	15chr11	47736782	47737090	14chr11	47869770	47870062	15chr1	153483424	153483662	14chr11	535581	535865	15chr1	155948186	155948423	14chr1	155959229	155959479	14chr1	155990654	155990860	15chr11	60928771	60929091	17chr1	161359564	161359796	13chr11	61515175	61515459	13chr11	62554051	62554248	13chr11	68214755	68215002	14chr11	69500673	69500895	16chr11	70601696	70601950	15chr1	171283054	171283328	12chr1	171750476	171750792	17chr11	72526738	72526984	15chr11	73088184	73088424	14chr11	75514339	75514543	15chr11	76757136	76757355	19chr11	77757255	77757514	17chr1	201451255	201451532	14chr1	201798058	201798313	15chr1	205253847	205254275	14chr12	100660739	100661007	13chr12	1058950	1059210	12chr12	109085514	109085706	15chr12	109232202	109232394	17chr12	110283123	110283411	16chr12	111136093	111136429	17chr12	120703582	120703899	14chr12	122211169	122211401	14chr1	2126632	2126897	15chr1	212732250	212732546	15chr12	127765282	127765578	15chr12	132638467	132638689	12chr1	220445683	220445998	19chr1	22290492	22290739	18chr1	224622445	224622686	13chr12	31556104	31556381	14chr1	236135073	236135405	13chr1	245026287	245026606	18chr12	49628972	49629205	16chr12	50236725	50237015	16chr12	51632498	51632797	14chr12	53746428	53746728	16chr12	57915871	57916167	13chr1	26560291	26560584	14chr12	66524441	66524715	12chr1	26690364	26690551	15chr12	68759036	68759352	13chr12	69550894	69551088	17chr12	7261759	7261975	15chr1	27324211	27324473	17chr1	27627361	27627584	17chr1	27684040	27684293	14chr12	8085877	8086124	17chr12	81171941	81172212	15chr12	93793004	93793208	14chr13	114894488	114894732	13chr1	31643665	31643941	14chr1	32014502	32014781	14chr13	21278198	21278464	14chr13	22245059	22245280	15chr13	31318527	31318794	16chr1	33921443	33921700	16chr13	41593331	41593560	16chr13	49106751	49107056	14chr13	50181218	50181457	13chr1	40506193	40506507	17chr1	40804443	40804726	13chr14	100534918	100535269	17chr14	103571356	103571614	16chr14	105116684	105116917	16chr14	105451975	105452213	14chr14	106765229	106765480	15chr1	41898406	41898627	15chr1	41981829	41982049	13chr14	24900957	24901252	15chr1	42612639	42612850	14chr14	31698994	31699219	16chr14	35805947	35806217	15chr1	44400497	44400702	14chr14	50320582	50320817	14chr14	52118155	52118385	12chr14	59215821	59216204	14chr14	69208581	69208884	17chr14	75774544	75774794	14chr14	92573020	92573229	16chr1	52499329	52499575	15chr15	31283730	31284047	16chr15	41953973	41954264	14chr15	43622632	43622972	14chr15	45722491	45722733	17chr15	63481303	63481690	11chr15	64752453	64752710	15chr15	65020446	65020712	15chr15	68966417	68966586	18chr15	74243479	74243765	12chr15	74265348	74265550	13chr15	74666035	74666285	16chr15	75135411	75135701	15chr15	85114215	85114533	16chr15	90895313	90895575	15chr16	115722	115957	17chr16	11626070	11626328	17chr16	12070347	12070632	14chr16	21964253	21964596	12chr16	2273493	2273724	11chr16	23464381	23464626	15chr1	6265953	6266258	16chr16	30470156	30470418	16chr16	31106123	31106360	15chr16	31119577	31119849	14chr16	31711741	31712055	15chr16	3661580	3661874	16chr1	63782666	63782922	14chr16	4304022	4304287	12chr16	4338388	4338730	14chr16	50300277	50300549	13chr1	6546606	6546854	13chr16	58545841	58546130	14chr16	67969590	67969939	15chr16	72127437	72127763	15chr16	81348269	81348489	17chr1	71546796	71547107	14chr17	16894775	16895011	12chr17	26220285	26220622	16chr17	27907416	27907648	16chr17	29764357	29764614	15chr17	33446763	33447046	14chr17	35306090	35306427	17chr17	37401027	37401234	16chr17	3817236	3817439	14chr17	38511361	38511540	17chr17	38689671	38690006	18chr17	40688027	40688278	16chr17	40770632	40770886	18chr17	41955968	41956208	15chr17	4613237	4613454	13chr17	47865820	47866133	14chr17	55980640	55980912	15chr17	58217211	58217406	16chr17	62103143	62103423	15chr17	62772939	62773291	16chr17	66755602	66755863	13chr17	6925360	6925623	14chr17	7199734	7199975	13chr17	75082664	75082880	16chr17	75243190	75243379	13chr17	75426180	75426454	14chr17	75877721	75877942	15chr17	76183104	76183408	17chr17	80255977	80256303	14chr17	9478984	9479238	15chr18	12934572	12934778	14chr18	3585865	3586074	13chr18	44676735	44677056	14chr18	48636798	48637051	13chr18	55710764	55711030	15chr18	57585731	57585973	14chr18	9899076	9899339	13chr19	10535010	10535347	15chr19	1194509	1194773	14chr19	1241536	1241802	14chr19	1244435	1244706	15chr19	13957373	13957660	15chr19	17420091	17420353	15chr19	17607104	17607369	14chr19	1826174	1826415	12chr19	19431229	19431536	13chr19	2056398	2056623	13chr19	21324624	21324972	15chr19	24269798	24270126	14chr19	35503951	35504182	15chr19	35558077	35558310	15chr19	35696234	35696469	19chr19	37825398	37825680	16chr19	41168726	41168979	15chr19	44289481	44289735	15chr19	45004459	45004831	14chr19	45829501	45829740	12chr19	46196631	46196830	16chr19	4870202	4870431	13chr19	48823323	48823564	15chr19	49378540	49378818	15chr19	49467090	49467335	15chr19	49977284	49977542	15chr19	49982094	49982333	14chr19	52598886	52599151	12chr19	54735570	54735821	15chr1	9555357	9555645	11chr19	56904823	56905107	17chr19	58257985	58258268	15chr19	664988	665180	14chr19	6753389	6753717	12chr19	8067301	8067600	15chr19	909227	909482	15chr19	9186333	9186600	19chr19	982712	982980	15chr20	18477735	18478037	14chr20	2489628	2489964	16chr20	29591629	29591838	16chr20	29611607	29612054	17chr20	30410636	30410940	16chr20	34582992	34583207	18chr20	36192843	36193093	15chr20	37262335	37262558	12chr20	39637029	39637272	14chr20	4089384	4089582	15chr20	48532104	48532441	16chr20	49094862	49095078	14chr20	49548115	49548420	16chr20	52210615	52210885	14chr20	60813142	60813416	13chr20	61075285	61075550	12chr20	62284570	62284810	12chr20	937149	937353	16chr2	10212759	10212993	17chr2	109150200	109150521	15chr2	109200486	109200743	14chr2	113931381	113931625	14chr21	15918574	15918787	15chr21	18985075	18985386	18chr2	128991129	128991417	16chr2	131113559	131113917	16chr2	132439934	132440206	16chr2	133104797	133105155	13chr21	35288056	35288276	15chr21	38362157	38362446	16chr21	45138759	45138983	12chr21	46712421	46712641	16chr2	196933074	196933266	16chr2	203879150	203879440	14chr2	208490033	208490305	17chr2	217642470	217642721	15chr22	18268042	18268290	14chr22	18560579	18560826	17chr2	219271245	219271468	15chr2	219922750	219922989	16chr22	19935326	19935485	14chr2	220024861	220025125	12chr22	22020143	22020474	19chr22	28315235	28315508	12chr2	232542420	232542674	17chr2	234763131	234763397	14chr22	37178101	37178307	15chr22	45831647	45831840	14chr2	26220673	26220907	16chr2	28817232	28817453	11chr2	38763256	38763556	14chr2	38892912	38893175	14chr2	47143036	47143337	16chr2	55746544	55746824	18chr2	69135640	69135913	13chr2	69405408	69405700	16chr2	71558673	71558951	14chr2	74413141	74413339	16chr2	85154151	85154393	14chr2	9563541	9563857	16chr3	124339492	124339681	15chr3	127293784	127294074	17chr3	14473782	14473999	13chr3	156272883	156273283	15chr3	156534703	156534975	19chr3	177888490	177888738	14chr3	185701354	185701577	16chr3	186857453	186857689	13chr3	196255664	196255968	15chr3	50365538	50365820	11chr3	53106664	53106955	15chr3	57261530	57261793	13chr3	8671168	8671446	17chr3	9791459	9791759	16chr4	127771521	127771739	15chr4	148538378	148538631	11chr4	17616066	17616378	13chr4	2243686	2243972	16chr4	2470664	2470977	13chr4	3294356	3294612	16chr4	38732808	38733042	15chr4	52709009	52709306	12chr4	52710817	52711103	16chr4	54232160	54232380	17chr4	54243603	54243923	12chr4	6976195	6976465	16chr4	84376828	84377172	15chr4	84469608	84469890	14chr5	116102269	116102540	15chr5	126113853	126114148	14chr5	132155851	132156131	15chr5	134527106	134527326	14chr5	137878986	137879337	15chr5	138598392	138598651	16chr5	145061266	145061487	13chr5	14664622	14664931	15chr5	149208049	149208275	15chr5	162932383	162932764	16chr5	172359456	172359665	17chr5	173472521	173472772	16chr5	176513978	176514289	14chr5	176918517	176918746	14chr5	176928020	176928288	12chr5	179125602	179125893	18chr5	179517484	179517713	16chr5	32613250	32613441	14chr5	33440676	33440895	14chr5	42583408	42583673	15chr5	56988973	56989201	15chr5	65439952	65440247	17chr5	78281489	78281823	16chr5	79543102	79543391	15chr6	107996629	107996799	12chr6	111279691	111280012	17chr6	125474759	125474961	15chr6	129993082	129993322	19chr6	135505034	135505331	15chr6	138188183	138188449	15chr6	139094474	139094747	15chr6	14877398	14877656	18chr6	159274372	159274757	18chr6	27113724	27114014	17chr6	27219569	27219799	15chr6	2846582	2846887	12chr6	30539059	30539364	16chr6	30689005	30689348	15chr6	30690423	30690713	14chr6	30923365	30923606	17chr6	31566422	31566622	17chr6	31783272	31783492	18chr6	32223006	32223284	15chr6	33247868	33248074	18chr6	35490653	35490970	13chr6	36328342	36328587	17chr6	36591281	36591499	14chr6	37019239	37019524	14chr6	37070306	37070625	17chr6	3912219	3912394	16chr6	40995725	40995993	13chr6	41651376	41651608	15chr6	43142306	43142665	15chr6	43351542	43351790	11chr6	43474477	43474756	12chr6	43597702	43597992	11chr6	53213686	53213966	14chr6	57182323	57182601	15chr6	88182417	88182792	15chr7	100048132	100048410	16chr7	100964958	100965249	12chr7	105449737	105449995	18chr7	131198314	131198566	13chr7	138144982	138145216	12chr7	139025108	139025484	15chr7	139762713	139762920	15chr7	151479960	151480227	17chr7	158769748	158770010	14chr7	17274131	17274412	16chr7	5405748	5405983	14chr7	6746523	6746774	12chr7	73686975	73687181	15chr7	75368665	75368969	14chr7	76178435	76178649	18chr7	98050351	98050578	15chr8	101571916	101572134	14chr8	11422054	11422385	13chr8	130694338	130694552	14chr8	134461736	134461963	14chr8	143808420	143808654	16chr8	22452758	22453022	16chr8	23267846	23268168	15chr8	41685885	41686154	14chr8	42698435	42698695	17chr8	48173244	48173515	14chr8	48651542	48651759	15chr8	67579509	67579927	16chr8	681105	681347	14chr8	74086620	74086889	15chr8	80677519	80677726	17chr8	87520777	87521095	16chr8	92052945	92053209	13chr9	1009201	1009411	14chr9	101018049	101018274	11chr9	116420293	116420501	13chr9	123638688	123639031	14chr9	123836937	123837262	19chr9	126980991	126981249	17chr9	130341267	130341534	16chr9	130683897	130684136	14chr9	131200943	131201169	15chr9	131266812	131267133	16chr9	132488998	132489176	13chr9	132546104	132546327	14chr9	135675560	135675810	16chr9	139686165	139686457	16chr9	139965004	139965280	17chr9	140095319	140095627	14chr9	14353539	14353729	14chr9	34458473	34458718	15chr9	34989487	34989729	13chr9	68726252	68726503	17chr9	80646806	80647031	13chr9	93799534	93799776	14chrX	118708387	118708642	12chrX	13671159	13671537	16chrX	2614762	2615016	16chrX	30907426	30907700	13chrX	47400583	47400846	15chrX	48534617	48534922	14chrX	48937462	48937724	13chrX	61694644	61694818	19chrX	77154690	77155045	15chr10	100206566	100206858	15chr10	102046305	102046617	15chr10	102772648	102772934	16chr10	102778748	102778992	13chr10	103989611	103989830	12chr10	112295813	112296125	15chr10	118764569	118764805	11chr10	125318930	125319151	13chr10	16478836	16479156	15chr10	30024736	30024973	14chr10	30648696	30648960	15chr10	556983	557262	11chr10	6128389	6128656	14chr10	6317241	6317516	13chr10	72338201	72338476	18chr10	72968876	72969094	16chr10	75608825	75609102	14chr10	75716373	75716588	14chr10	76871020	76871372	13chr10	80015879	80016111	15chr10	86088004	86088244	16chr10	89577818	89578085	12chr10	95653545	95653871	13chr10	98400740	98400986	15chr1	10010479	10010710	14chr1	100715227	100715433	11chr1	109373143	109373473	16chr1	110186019	110186208	15chr11	10476476	10476794	16chr11	1051131	1051386	15chr11	111317284	111317549	13chr11	116662028	116662283	15chr11	116967259	116967493	15chr11	117103169	117103372	13chr11	117198395	117198726	14chr11	118955476	118955764	16chr11	119252261	119252558	12chr1	113258960	113259214	12chr1	113261305	113261561	16chr1	115300639	115300851	14chr1	116711040	116711293	12chr1	117027907	117028133	14chr11	19138532	19138818	14chr11	1968448	1968748	14chr1	12679935	12680142	14chr11	2909626	2909889	14chr11	31832597	31832897	14chr11	3846052	3846229	14chr1	148556649	148556912	18chr1	151253671	151254010	14chr1	151512687	151512977	11chr1	153505664	153505906	14chr1	153571378	153571641	17chr1	154971579	154971844	14chr1	155293134	155293319	14chr1	156066507	156066705	13chr11	59317990	59318218	14chr1	15943868	15944123	12chr1	159861379	159861601	13chr11	62673499	62673707	13chr11	63706141	63706391	13chr11	64013261	64013526	12chr11	64073450	64073714	15chr11	64084948	64085255	14chr11	64877834	64878067	14chr11	65667752	65668017	14chr11	65670595	65670883	18chr11	66104151	66104484	15chr1	167237110	167237349	15chr1	167905267	167905512	12chr11	71736232	71736440	13chr11	73309508	73309780	13chr1	173400378	173400629	16chr11	74109752	74110053	15chr11	747181	747469	13chr11	75156401	75156639	13chr11	76029907	76030067	13chr11	77530418	77530677	14chr1	17828899	17829081	15chr1	178986224	178986485	14chr1	182583990	182584410	12chr1	182808444	182808701	14chr11	910677	910954	15chr1	201404933	201405179	16chr1	201673375	201673594	14chr1	204367704	204367907	14chr1	20820323	20820594	11chr1	210757764	210758012	13chr12	111843170	111843383	10chr12	113536183	113536507	14chr1	211688750	211689061	17chr12	116997608	116997824	12chr12	118312707	118312948	13chr12	124938033	124938339	16chr12	125251640	125251855	16chr12	132314968	132315247	16chr1	220267299	220267610	15chr1	226309257	226309551	16chr1	228318760	228318973	14chr1	228594416	228594706	14chr1	2313241	2313455	14chr12	31789613	31789925	14chr1	2343914	2344157	17chr1	23823656	23823841	14chr1	23963996	23964176	16chr1	24069804	24070068	14chr1	245134054	245134316	14chr1	2479487	2479733	15chr12	48173136	48173322	15chr12	50135282	50135482	12chr12	52417498	52417762	15chr12	53264277	53264550	12chr12	53278652	53278909	14chr12	54399748	54400052	11chr12	54688042	54688285	17chr12	56552080	56552345	13chr12	56583340	56583567	14chr12	56660538	56660791	14chr12	57521012	57521256	10chr12	6532789	6533047	13chr1	26648055	26648355	17chr12	67662513	67662867	13chr12	6934805	6935017	12chr12	6961552	6961758	15chr12	6976612	6976838	14chr12	70132324	70132588	15chr12	7047194	7047441	16chr12	75723882	75724174	16chr12	76017639	76017923	11chr12	8012547	8012800	14chr12	8147886	8148108	16chr12	93835582	93835831	15chr12	96017439	96017668	15chr13	107188805	107189078	14chr13	111124188	111124413	16chr13	114048347	114048608	14chr1	32420340	32420537	14chr13	27559473	27559749	13chr13	40229710	40229953	14chr13	41019640	41019932	20chr13	45151903	45152177	11chr13	50069942	50070206	14chr13	50422110	50422355	14chr1	35331942	35332203	15chr1	36834669	36834928	15chr13	80623035	80623261	15chr1	39325466	39325717	11chr13	96705532	96705754	14chr14	24423235	24423466	13chr1	43123905	43124171	15chr14	34529398	34529703	13chr14	35515212	35515477	15chr14	36295248	36295547	17chr14	45431030	45431361	13chr14	55569970	55570211	12chr1	45805620	45805986	13chr14	62129264	62129506	13chr14	64805514	64805761	11chr14	75745234	75745492	13chr14	90701713	90701966	14chr14	91224838	91225079	15chr15	23034314	23034580	16chr15	23810603	23810865	14chr15	31727565	31727807	16chr1	53387271	53387568	16chr1	53480514	53480684	16chr15	41196272	41196469	14chr15	42376633	42376890	14chr15	44829225	44829423	13chr15	45145592	45145877	13chr15	52548577	52548838	16chr1	55487774	55487997	14chr15	60771287	60771565	15chr15	64540211	64540433	16chr15	65127952	65128209	10chr15	68599007	68599328	11chr15	70391815	70392061	11chr15	72072653	72073085	14chr15	74676144	74676491	12chr15	90304026	90304261	15chr15	91412977	91413193	14chr16	102921	103214	14chr16	11422431	11422691	11chr16	11490176	11490361	14chr16	14013916	14014165	12chr16	20753015	20753227	16chr16	21831643	21831913	17chr16	24740776	24741022	16chr16	27442322	27442633	11chr16	30124170	30124401	12chr16	30538102	30538318	13chr16	31044604	31044901	14chr16	3213410	3213653	14chr1	6403585	6403808	13chr16	4452664	4452942	14chr16	53737700	53738019	13chr16	57791205	57791386	12chr16	629141	629371	15chr16	66981159	66981399	14chr16	67753279	67753492	14chr16	67840508	67840840	15chr16	69166368	69166617	15chr16	70510518	70510761	14chr16	71392434	71392708	12chr16	72866540	72866745	15chr16	761770	762000	13chr16	81785889	81786106	15chr16	85089063	85089412	12chr16	85120397	85120608	15chr16	85148572	85148803	15chr16	86609400	86609642	16chr16	87495105	87495364	16chr17	15862516	15862767	16chr17	16322454	16322705	14chr17	1958228	1958453	13chr17	20224146	20224437	14chr17	26578702	26578944	15chr17	26989066	26989334	15chr17	27918690	27918943	14chr17	29058539	29058840	13chr17	29885773	29886044	16chr17	36648358	36648587	15chr17	39804232	39804489	15chr17	39894341	39894562	12chr17	40176870	40177201	17chr17	40776230	40776487	17chr17	41755354	41755522	14chr17	42245837	42246064	11chr17	43099324	43099561	14chr17	43472477	43472781	12chr17	44026427	44026651	14chr17	46026682	46026957	14chr17	46102436	46102692	14chr17	46103859	46104107	15chr17	46969917	46970238	15chr17	47049759	47050024	15chr17	47633719	47633946	16chr17	57930631	57930915	14chr17	60186877	60187104	17chr17	61912256	61912460	13chr17	67323176	67323524	17chr17	70536491	70536739	13chr17	7232662	7232904	15chr17	72375619	72375887	16chr17	72740834	72741055	15chr17	7338514	7338688	14chr17	73422465	73422678	16chr17	74099750	74100051	13chr17	76123325	76123625	15chr17	76130497	76130750	14chr17	76588355	76588791	15chr17	79268968	79269181	13chr1	8014173	8014455	15chr18	11732982	11733255	13chr18	12727109	12727312	13chr18	3624013	3624286	18chr18	46201691	46201924	12chr18	46303515	46303724	14chr18	56209005	56209267	15chr18	60251599	60251846	15chr18	61034861	61035070	14chr18	74534711	74534914	14chr18	77404106	77404353	15chr18	9147958	9148220	15chr19	10360780	10361010	16chr19	10755486	10755685	14chr19	14517026	14517288	15chr19	14530040	14530314	11chr19	17393492	17393708	14chr19	18284498	18284808	14chr19	19030303	19030606	15chr19	2087085	2087386	17chr19	21579724	21580056	14chr19	23578116	23578443	13chr19	30207457	30207676	15chr19	3076026	3076221	15chr19	37019241	37019736	13chr19	3762531	3762803	13chr19	37701279	37701618	15chr19	39127582	39127868	14chr19	39421405	39421713	11chr19	3987847	3988066	14chr19	39900092	39900275	16chr19	40596676	40596987	12chr19	41859976	41860171	16chr19	42637277	42637545	13chr19	42721932	42722237	15chr19	46184814	46185025	14chr19	46850651	46850826	15chr19	46916764	46917025	17chr19	47729346	47729624	16chr19	48948922	48949329	14chr19	49092141	49092448	16chr19	49149829	49150021	14chr19	49242192	49242477	17chr19	50009910	50010148	13chr19	52097605	52097884	15chr19	52551962	52552210	12chr19	53104289	53104666	14chr19	55813218	55813531	13chr19	56592019	56592336	18chr19	57791157	57791418	15chr19	6126432	6126700	15chr19	7600405	7600639	14chr19	7633362	7633572	15chr19	8509621	8509952	12chr19	9250928	9251195	12chr1	9970583	9970801	15chr1	999603	999822	17chr20	30679773	30679985	12chr20	311259	311577	13chr20	31366298	31366513	12chr20	31797652	31797866	13chr20	3189994	3190292	14chr20	33735031	33735263	12chr20	35184957	35185188	14chr20	3799055	3799317	14chr20	39968642	39968931	15chr20	42745386	42745565	14chr20	42939770	42940021	14chr20	45989442	45989772	14chr20	46093234	46093448	14chr20	55926153	55926389	13chr20	56964023	56964308	13chr20	62372165	62372485	14chr20	626192	626463	14chr20	62751778	62752028	12chr21	26859598	26859847	12chr21	27173889	27174134	12chr2	128165613	128165809	17chr2	129494246	129494481	12chr2	131100286	131100543	14chr21	36218104	36218336	16chr21	36262368	36262635	16chr21	45432019	45432233	16chr21	46293498	46293701	13chr21	46707688	46708006	16chr21	46974819	46975054	16chr2	174267195	174267423	16chr2	175206305	175206531	14chr2	206790579	206790891	15chr2	219081684	219081871	14chr2	219762978	219763181	14chr22	20226115	20226381	15chr22	20255876	20256137	15chr22	22011664	22012003	14chr2	228270914	228271186	14chr2	232410224	232410488	16chr2	232545868	232546127	13chr2	232765261	232765441	12chr22	36783938	36784300	16chr22	38070979	38071268	15chr2	238421494	238421672	14chr2	242088743	242089026	16chr22	43058033	43058322	14chr22	47194552	47194804	10chr2	26986997	26987254	15chr2	27357357	27357609	13chr2	44395131	44395447	13chr2	57987052	57987310	12chr2	62684140	62684414	15chr2	64977892	64978259	14chr2	68589252	68589421	13chr2	69534554	69534763	14chr2	88899668	88899945	13chr2	97616956	97617203	15chr2	98207301	98207555	14chr3	119041809	119042139	18chr3	122291845	122292156	13chr3	125239085	125239341	14chr3	127174474	127174677	15chr3	128135689	128135961	14chr3	129830208	129830480	14chr3	133524468	133524788	15chr3	148847253	148847442	14chr3	152856523	152856794	18chr3	156392207	156392495	9chr3	156543872	156544152	15chr3	156855264	156855515	14chr3	16090219	16090463	13chr3	182879448	182879752	16chr3	184032228	184032452	15chr3	196370043	196370359	13chr3	196696765	196696996	17chr3	197807510	197807739	15chr3	32989283	32989543	14chr3	45209046	45209253	14chr3	45252128	45252310	16chr3	45266708	45266907	12chr3	47323967	47324269	17chr3	47422296	47422588	14chr3	48282486	48282691	15chr3	48672861	48673138	15chr3	48777447	48777684	12chr3	49941271	49941535	16chr3	5065052	5065323	15chr3	72897452	72897776	13chr3	8664627	8664885	15chr3	9757501	9757787	14chr4	10020446	10020736	16chr4	101014554	101014889	14chr4	10118611	10118812	13chr4	110624129	110624450	16chr4	113445013	113445358	17chr4	113558163	113558503	12chr4	140586797	140587120	15chr4	169752969	169753345	15chr4	18023683	18023966	12chr4	190825652	190825821	12chr4	4337022	4337254	16chr4	68411046	68411282	11chr4	7072492	7072736	14chr4	77222566	77222824	14chr4	8244033	8244240	14chr5	108804556	108804751	14chr5	126083765	126084091	15chr5	138940649	138940955	17chr5	139781032	139781410	15chr5	140699802	140700046	13chr5	141392527	141392848	13chr5	148520895	148521141	16chr5	149829172	149829519	15chr5	150505800	150506015	16chr5	170171539	170171758	17chr5	170845689	170845908	14chr5	172124510	172124792	14chr5	172143864	172144105	13chr5	173000190	173000379	14chr5	178157636	178157915	15chr5	31532212	31532550	14chr5	39217958	39218192	13chr5	55290711	55291034	13chr5	68630627	68630863	14chr5	73980827	73981162	14chr6	111265891	111266124	15chr6	13336814	13336983	14chr6	143381478	143381855	14chr6	161695115	161695383	15chr6	166795300	166795581	13chr6	19804931	19805217	17chr6	27106909	27107135	15chr6	27860862	27861181	16chr6	29596421	29596698	14chr6	30845588	30845796	15chr6	31509610	31509848	14chr6	31795482	31795741	14chr6	32121678	32121982	15chr6	33290671	33290912	13chr6	35208904	35209157	14chr6	36091654	36091886	16chr6	38670839	38671138	11chr6	4136160	4136418	16chr6	42013408	42013751	14chr6	42869288	42869455	17chr6	42897792	42898080	13chr6	43138724	43139059	13chr6	43894460	43894688	13chr6	4647864	4648122	11chr6	47102487	47102716	16chr6	7239124	7239350	14chr6	7389870	7390159	15chr6	74290077	74290351	12chr6	7590292	7590537	15chr6	80487737	80488000	13chr7	100743467	100743722	14chr7	104524192	104524477	16chr7	105987146	105987349	17chr7	107886777	107887053	15chr7	129592718	129592958	17chr7	129691024	129691284	18chr7	130353403	130353756	15chr7	150020072	150020415	14chr7	151065244	151065478	15chr7	151451229	151451498	17chr7	22149940	22150164	14chr7	22893719	22893980	15chr7	2491037	2491339	13chr7	30066373	30066563	13chr7	32535014	32535288	17chr7	35077463	35077750	13chr7	36155171	36155441	14chr7	38948665	38948910	14chr7	43689432	43689656	15chr7	44517027	44517294	12chr7	4869784	4870017	14chr7	5598552	5598809	13chr7	6865818	6866054	16chr7	75623836	75624159	15chr7	7680208	7680520	18chr7	87849238	87849611	15chr7	99588582	99588812	13chr8	131540644	131540906	15chr8	136935419	136935641	13chr8	144416673	144416941	15chr8	144976711	144976955	14chr8	22085330	22085594	14chr8	22876761	22877003	14chr8	26148772	26149094	14chr8	29197790	29198063	16chr8	356814	357082	14chr8	37749441	37749664	15chr8	42547704	42547936	13chr8	42948447	42948728	14chr8	47829364	47829607	15chr8	75262556	75262808	14chr8	8130816	8130999	17chr9	101077897	101078136	15chr9	112203052	112203310	14chr9	117147802	117148028	13chr9	123975557	123975797	15chr9	128137035	128137234	14chr9	130632217	130632463	16chr9	130681710	130681955	12chr9	130717741	130717956	15chr9	131495593	131495788	13chr9	136114504	136114773	14chr9	138360820	138361145	13chr9	139923898	139924081	14chr9	139977877	139978163	15chr9	140306189	140306407	14chr9	33722160	33722345	14chr9	37415243	37415475	14chr9	38672403	38672719	16chr9	74340942	74341152	13chr9	97021461	97021718	14chrX	13687412	13687639	13chrX	153707383	153707722	16chrX	47420727	47421040	13chrX	57021947	57022144	15chrX	69653714	69653948	12chr10	100174845	100175090	14chr10	100227272	100227533	12chr10	102822362	102822646	12chr10	103347901	103348119	11chr10	112064712	112064939	14chr10	114143176	114143383	14chr10	114847209	114847373	14chr10	119202469	119202737	12chr10	126490173	126490464	12chr10	31607227	31607523	11chr10	32049170	32049437	14chr10	33269484	33269722	12chr10	35283602	35283870	13chr10	44806538	44806795	15chr10	45360321	45360611	13chr10	45470310	45470636	14chr10	45504111	45504365	10chr10	65467110	65467305	18chr10	70982481	70982710	12chr10	71389895	71390147	15chr10	71674455	71674678	11chr10	74014922	74015195	14chr10	74124138	74124362	13chr10	76859779	76859947	13chr10	79807127	79807323	13chr10	92631549	92631853	12chr1	109419467	109419727	13chr1	109969032	109969384	14chr11	102212602	102212850	13chr11	10477405	10477643	10chr1	111162072	111162228	13chr11	112160777	112160989	10chr11	114030761	114030986	15chr11	119352079	119352326	12chr11	130304400	130304645	14chr1	113615438	113615786	13chr1	115124272	115124514	14chr11	1715243	1715489	11chr11	19463862	19464120	11chr11	2326667	2326846	13chr1	12589472	12589703	18chr11	3113064	3113308	14chr11	32914006	32914295	15chr1	1337427	1337720	13chr11	356269	356477	13chr11	3829178	3829421	10chr11	4208801	4209064	14chr1	144918061	144918311	16chr1	145713605	145713871	14chr1	149658443	149658685	15chr1	151168793	151169060	13chr1	151707557	151707801	14chr1	153959264	153959578	13chr1	154244732	154245008	16chr1	1550586	1550974	16chr1	155197162	155197444	13chr1	156326758	156326954	13chr1	159906294	159906483	14chr1	16067890	16068145	13chr11	60682861	60683223	17chr1	161162388	161162569	13chr11	61447676	61447877	15chr11	62369245	62369577	16chr11	63246331	63246576	14chr11	63639543	63639760	13chr11	64122626	64122891	13chr11	66206431	66206667	15chr1	167028375	167028675	14chr11	67381540	67381873	15chr11	68934066	68934316	14chr11	695727	696028	12chr11	71511453	71511676	12chr11	72853348	72853653	17chr11	73000534	73000785	15chr11	73006504	73006752	12chr11	73062310	73062537	14chr11	73694122	73694375	10chr11	75236796	75237050	11chr1	180991948	180992215	13chr1	182112360	182112575	15chr11	8932626	8932864	12chr11	95438753	95438965	13chr1	19812305	19812583	13chr1	19980253	19980500	14chr1	20042129	20042373	12chr1	201417273	201417591	15chr1	202558412	202558682	14chr1	203643260	203643566	15chr1	204131625	204131891	16chr1	205225328	205225598	14chr1	205242695	205242903	15chr1	205257414	205257628	14chr1	207752768	207753013	12chr1	210001180	210001443	15chr12	102224698	102224923	12chr12	104026413	104026616	14chr12	108955020	108955307	13chr12	121647754	121648054	16chr12	125425274	125425468	16chr1	212650584	212650754	13chr12	132991573	132991778	14chr1	226084886	226085165	15chr1	230240479	230240760	17chr1	235012088	235012353	14chr12	46867470	46867739	16chr12	47055106	47055458	15chr12	498709	498971	13chr12	54582769	54583051	16chr12	54793169	54793353	14chr12	56401047	56401244	15chr12	57028833	57029048	13chr12	57607175	57607417	14chr12	58150625	58150809	13chr1	26137424	26137592	14chr12	6277013	6277262	13chr12	6726917	6727205	13chr12	7068333	7068568	13chr1	27932927	27933191	14chr1	28099571	28099906	11chr12	90150553	90150828	14chr1	29203748	29203977	17chr12	92537902	92538140	13chr12	94495212	94495462	12chr12	9822159	9822402	12chr13	20207542	20207857	14chr13	30046558	30046776	16chr13	31439486	31439774	13chr1	33430397	33430717	14chr1	33722464	33722757	13chr13	41363382	41363708	13chr1	3541229	3541521	11chr1	36713967	36714231	15chr13	73356092	73356398	12chr1	38325823	38326085	15chr1	40167568	40167809	14chr14	100844194	100844417	15chr14	102094812	102095050	17chr14	102427479	102427745	14chr14	102780560	102780777	10chr14	105864690	105864959	15chr14	105956041	105956322	12chr14	22802510	22802719	14chr14	23005908	23006127	14chr14	23039215	23039393	17chr14	24036837	24037107	14chr14	24578129	24578348	11chr14	24802855	24803097	15chr14	25148167	25148469	12chr14	35344402	35344797	12chr1	45118597	45118784	12chr14	55369411	55369760	14chr14	58637865	58638127	13chr1	46152654	46152999	13chr14	67999678	67999915	10chr14	68734976	68735161	11chr14	69255707	69255935	14chr14	70193719	70193972	14chr1	47069649	47069957	13chr1	47641117	47641315	12chr14	99984821	99985023	13chr15	101629066	101629273	14chr15	36871605	36871897	13chr15	41836339	41836592	14chr15	44580733	44581027	15chr15	50647299	50647501	12chr15	63569286	63569507	13chr15	65325490	65325693	13chr15	65590107	65590314	13chr15	70390253	70390469	13chr15	75335393	75335664	13chr15	83654784	83655114	13chr15	85131320	85131554	15chr15	88790048	88790322	11chr15	90602852	90603074	12chr15	90701192	90701434	11chr15	91475690	91475977	15chr15	99619972	99620196	15chr16	10603490	10603711	14chr16	1350927	1351195	13chr16	14622992	14623226	15chr16	1463769	1463970	13chr16	15521550	15521787	14chr16	27468512	27468697	14chr16	2924907	2925129	16chr16	29937347	29937722	12chr16	3011764	3012005	11chr1	6306804	6307048	13chr16	3078235	3078471	14chr16	30905443	30905724	12chr16	3493412	3493671	11chr16	4401218	4401430	10chr16	4674286	4674599	13chr1	65431922	65432172	14chr16	57610357	57610530	13chr16	58521680	58521937	13chr16	66558749	66559022	14chr16	66642542	66642790	13chr16	68544115	68544327	16chr16	72698874	72699058	12chr1	67519542	67519861	14chr1	68152354	68152631	14chr16	84641446	84641636	16chr16	85309302	85309487	11chr16	86698142	86698427	14chr16	8754858	8755080	15chr16	9232972	9233251	12chr17	15554973	15555259	17chr17	29152842	29153095	12chr17	2952084	2952337	15chr17	31264009	31264290	12chr17	38497417	38497708	11chr17	38673072	38673323	14chr17	40669521	40669784	14chr17	40679303	40679606	13chr17	40718940	40719192	15chr17	40915478	40915709	15chr17	40950594	40950897	14chr17	42441112	42441321	15chr17	44913341	44913534	15chr17	54857648	54857926	13chr17	60781130	60781403	14chr17	6554739	6555019	13chr17	66432548	66432786	12chr17	70417399	70417633	18chr17	71251809	71252070	14chr17	72653613	72653854	13chr1	7727681	7727931	12chr17	73512379	73512613	18chr17	74553702	74553922	14chr17	7464372	7464617	13chr17	75319194	75319446	15chr17	75327588	75327865	15chr17	75491081	75491267	15chr17	76418181	76418354	16chr17	7646961	7647171	14chr17	77785117	77785391	11chr17	77982282	77982472	14chr17	79116599	79116840	14chr17	79215535	79215752	13chr17	80477290	80477559	14chr17	80509736	80509946	14chr17	8062138	8062333	15chr18	10787404	10787601	16chr18	12039006	12039224	14chr18	12948196	12948541	16chr18	21166600	21166958	15chr18	30050747	30050995	13chr18	32811054	32811209	14chr18	47807822	47808044	14chr1	84944758	84945062	13chr18	77006580	77006816	11chr18	77772886	77773055	17chr18	839386	839643	14chr18	9869858	9870140	12chr19	10025347	10025638	13chr19	11146644	11146862	14chr19	11202491	11202750	13chr19	11646929	11647183	13chr19	1269170	1269450	15chr19	12958439	12958682	15chr19	14606974	14607214	13chr19	14667565	14667776	15chr19	16181284	16181551	12chr1	9170825	9171172	13chr19	17791001	17791268	14chr19	18279042	18279262	14chr19	18362667	18362898	16chr19	18415340	18415600	13chr19	19335943	19336242	14chr19	19643095	19643285	12chr19	2085312	2085632	16chr19	21203286	21203572	12chr19	23456923	23457215	14chr1	93426007	93426287	14chr19	34745281	34745509	14chr19	35605562	35605862	15chr19	37064042	37064331	13chr19	39109662	39109919	10chr19	40372253	40372437	14chr19	41104502	41104740	15chr19	42783919	42784246	14chr19	44488150	44488404	14chr19	44529339	44529556	13chr19	44711465	44711768	17chr19	45393738	45393979	13chr19	45406913	45407208	14chr19	46195061	46195365	12chr19	4724590	4724755	15chr19	48216474	48216734	14chr19	48825055	48825364	16chr19	4911906	4912201	12chr19	49465615	49465886	13chr19	50871899	50872143	12chr19	556031	556292	13chr19	55657502	55657723	14chr19	55718549	55718812	15chr19	57791691	57791906	12chr19	59066270	59066521	15chr19	6022309	6022563	16chr19	6393070	6393371	14chr19	7069534	7069814	13chr19	7746597	7746880	16chr19	7918225	7918470	14chr19	8634048	8634278	13chr19	863941	864167	15chr20	23618459	23618704	14chr20	25291014	25291256	12chr20	25604685	25604939	12chr20	361445	361750	15chr20	37590676	37591000	15chr20	57227282	57227596	13chr20	60641032	60641225	12chr20	62733423	62733631	12chr2	102637123	102637352	15chr2	105953819	105954091	13chr2	10972456	10972716	14chr2	113638844	113639085	13chr2	114384702	114384905	13chr21	19191531	19191857	13chr2	12003815	12004128	15chr2	120301800	120301971	12chr2	127822333	127822734	13chr21	30568724	30568940	15chr21	38349339	38349590	14chr2	139259112	139259327	14chr21	43378244	43378531	14chr21	45148562	45148837	10chr21	45930472	45930675	13chr21	46859345	46859578	15chr21	47573203	47573491	13chr2	172968607	172968872	12chr2	179269181	179269396	18chr2	200320401	200320647	17chr2	201577854	201578089	15chr2	204192781	204193059	14chr2	205889845	205890084	14chr2	20725000	20725354	15chr2	20842241	20842412	12chr22	20960570	20960838	14chr22	21336368	21336607	13chr22	22001038	22001240	13chr22	22221973	22222249	12chr22	25631897	25632106	12chr22	27068811	27069091	14chr2	232770823	232771043	14chr22	37595497	37595813	15chr2	238969295	238969576	12chr22	39151963	39152233	13chr22	39707007	39707216	13chr22	39928723	39928980	15chr22	41418580	41418869	15chr2	242892980	242893275	14chr22	43355804	43356022	14chr22	43659863	43660061	17chr22	45093343	45093646	11chr22	50354220	50354466	14chr2	25584885	25585144	13chr2	25599701	25599925	13chr2	27233705	27233934	14chr2	28675368	28675602	14chr2	37423449	37423756	13chr2	42722150	42722390	12chr2	43054801	43055086	13chr2	43202111	43202453	15chr2	54198068	54198324	15chr2	54760200	54760437	13chr2	64870439	64870651	13chr2	65267882	65268092	15chr2	70141552	70141899	16chr2	70914036	70914300	14chr2	71098700	71098934	11chr2	71228230	71228496	14chr2	74619081	74619353	13chr2	85645445	85645778	13chr2	86041398	86041614	12chr2	9346339	9346624	14chr2	97197811	97198060	14chr3	10266573	10266838	15chr3	113871381	113871610	14chr3	123005241	123005413	16chr3	123442482	123442717	13chr3	124283867	124284102	12chr3	12447320	12447528	15chr3	126471132	126471391	14chr3	127794469	127794719	17chr3	128968298	128968520	13chr3	130745477	130745774	11chr3	132071447	132071721	13chr3	134027306	134027553	14chr3	134093189	134093440	16chr3	136751317	136751582	11chr3	138634282	138634517	15chr3	14302695	14302950	16chr3	151022900	151023151	13chr3	15244285	15244537	14chr3	158519665	158519886	16chr3	169491003	169491295	14chr3	169899303	169899583	12chr3	178979096	178979362	16chr3	185655726	185655957	16chr3	194403413	194403619	17chr3	194992226	194992492	13chr3	197439187	197439446	16chr3	40498665	40498957	14chr3	43810976	43811200	15chr3	4534832	4535105	15chr3	47823292	47823613	16chr3	48545105	48545351	13chr3	49203692	49203911	13chr3	49481527	49481708	13chr3	49823937	49824235	14chr3	5022091	5022308	12chr3	52254536	52254736	14chr3	52481098	52481310	12chr3	58518532	58518745	14chr4	108641441	108641759	19chr4	114468681	114468950	15chr4	140374804	140375074	12chr4	151936836	151937144	14chr4	171180799	171181034	14chr4	174173517	174173770	14chr4	1747999	1748201	14chr4	177116684	177116969	15chr4	185458599	185458876	12chr4	185734526	185734829	13chr4	186347013	186347334	13chr4	186495003	186495324	13chr4	26199066	26199282	14chr4	3445261	3445477	14chr4	39471082	39471292	12chr4	56412870	56413160	14chr4	6722052	6722268	16chr4	71767922	71768227	13chr4	75607985	75608236	14chr4	99388770	99389002	15chr5	102201367	102201781	15chr5	130506313	130506586	13chr5	131755362	131755632	13chr5	132576229	132576482	14chr5	133474125	133474310	11chr5	134801848	134802154	13chr5	138533769	138534087	13chr5	138727416	138727687	12chr5	140700167	140700521	14chr5	150603586	150603778	12chr5	156569738	156570058	13chr5	172410692	172410981	13chr5	172710880	172711118	13chr5	172759017	172759319	15chr5	173191525	173191736	12chr5	177547949	177548222	12chr5	179159419	179159710	12chr5	271563	271836	14chr5	32542792	32543015	12chr5	368793	369067	13chr5	54040242	54040504	15chr5	56946175	56946477	12chr5	57756573	57756893	17chr5	64064393	64064719	12chr5	75012869	75013152	11chr5	75668467	75668704	13chr5	7816394	7816644	13chr5	79551927	79552178	15chr5	90610153	90610371	16chr5	95048182	95048402	14chr6	11044699	11044963	14chr6	119468571	119468799	16chr6	130069542	130069738	15chr6	130342185	130342446	14chr6	1335703	1335988	14chr6	13795399	13795599	14chr6	151645412	151645695	13chr6	1604649	1604830	15chr6	16761810	16762097	14chr6	25962898	25963155	11chr6	27721905	27722104	10chr6	28092209	28092450	14chr6	28234834	28235145	13chr6	30098872	30099058	16chr6	30421465	30421690	14chr6	30509313	30509569	12chr6	30698630	30698945	16chr6	31697195	31697460	13chr6	31737581	31737843	16chr6	33244736	33245047	15chr6	34122560	34122825	13chr6	35705385	35705609	13chr6	36308755	36308996	13chr6	36514969	36515327	17chr6	36723480	36723805	17chr6	37786635	37786943	13chr6	39134286	39134482	10chr6	42012014	42012203	16chr6	42515366	42515606	10chr6	43231234	43231474	13chr6	43422319	43422602	12chr6	44046924	44047192	11chr6	47210295	47210509	14chr6	52463076	52463346	12chr6	6750279	6750503	13chr6	8435643	8435842	13chr6	88433036	88433247	14chr6	88441577	88441880	15chr7	11013365	11013630	14chr7	115850406	115850725	14chr7	129588732	129589010	13chr7	129598547	129598798	13chr7	143534406	143534616	15chr7	155137599	155137905	15chr7	157066296	157066521	12chr7	2710656	2710841	15chr7	32982728	32982913	12chr7	33168902	33169291	13chr7	37488360	37488717	16chr7	44645969	44646165	13chr7	44677762	44678021	13chr7	47305889	47306188	12chr7	50535764	50536009	13chr7	5125102	5125319	14chr7	5465196	5465435	13chr7	54841928	54842131	13chr7	5609731	5610024	13chr7	5744141	5744358	13chr7	73021432	73021677	14chr7	73130056	73130285	15chr7	73815673	73815893	13chr7	87555988	87556276	13chr7	89783618	89783890	16chr7	97650916	97651173	14chr7	97881479	97881721	12chr7	98048257	98048517	13chr7	98910584	98910819	14chr7	99156098	99156345	15chr8	110656890	110657178	13chr8	116439901	116440152	13chr8	125605234	125605474	15chr8	12613134	12613375	15chr8	130548056	130548296	15chr8	135844597	135844835	17chr8	142094440	142094664	17chr8	142396911	142397138	13chr8	144614290	144614516	13chr8	145022675	145022925	15chr8	145049050	145049314	12chr8	145137381	145137634	12chr8	145693088	145693317	12chr8	146017668	146017934	14chr8	21966820	21967040	12chr8	22454951	22455226	15chr8	27449769	27450019	15chr8	37756874	37757119	14chr8	42995332	42995542	15chr8	56798223	56798471	14chr8	6264016	6264221	14chr8	95274455	95274750	12chr9	110045260	110045485	16chr9	115512708	115512939	14chr9	123659508	123659740	18chr9	127181900	127182078	11chr9	127615321	127615594	16chr9	130706062	130706244	15chr9	131102644	131102855	14chr9	136223307	136223530	14chr9	137341499	137341748	13chr9	139525906	139526113	13chr9	140172020	140172311	12chr9	15422585	15422859	15chr9	19161747	19161993	17chr9	33167509	33167741	13chr9	34620400	34620684	14chr9	34675125	34675365	13chr9	35489759	35490046	16chr9	36190558	36190934	16chr9	36400166	36400395	12chr9	5839318	5839561	12chr9	72873809	72874115	12chr9	77567226	77567432	13chr9	91933784	91933996	11chr9	93910079	93910281	14chr9	96928955	96929175	14chr9	97316723	97317008	12chr9	98189053	98189400	12chrX	103410903	103411177	14chrX	11129137	11129463	12chrX	153597959	153598210	14chrX	40440021	40440299	12chrX	40594972	40595167	14chrX	41301563	41301845	13chrX	49091814	49092058	13chrX	70315480	70315732	12chrX	9319911	9320134	14chr10	102102183	102102476	13chr10	102802701	102802947	14chr10	104613993	104614264	15chr10	105677752	105678081	13chr10	111985507	111985714	12chr10	121412033	121412247	15chr10	122702420	122702624	13chr10	126429921	126430174	14chr10	129924581	129924842	12chr10	131988427	131988698	15chr10	133836669	133836876	14chr10	17258277	17258529	14chr10	25349046	25349285	13chr10	29947981	29948215	17chr10	4705395	4705609	13chr10	52381758	52382022	15chr10	73648622	73648954	14chr10	74096949	74097162	12chr10	74436228	74436416	13chr10	74451620	74451933	11chr10	81963243	81963507	12chr10	97134478	97134683	16chr10	97190220	97190406	13chr10	99408781	99409009	16chr1	100315487	100315755	12chr1	10488136	10488396	12chr1	110316319	110316565	13chr11	107611725	107612030	13chr11	115093234	115093454	14chr11	117817619	117817856	12chr11	118530289	118530462	13chr11	11870875	11871117	13chr11	118777800	118777991	12chr11	120206858	120207071	13chr11	126081456	126081747	12chr1	113351926	113352146	13chr1	113683255	113683458	13chr1	117664711	117664934	15chr11	18265385	18265610	14chr11	22647296	22647534	12chr11	35028337	35028521	9chr11	46373784	46373986	12chr11	46401972	46402498	11chr1	150997088	150997303	14chr1	151284091	151284329	11chr1	151566705	151566930	13chr1	151584297	151584547	14chr1	152413419	152413621	14chr1	154307569	154307808	13chr1	155017409	155017616	11chr1	155176701	155176934	14chr1	155881099	155881408	15chr1	156076074	156076279	13chr1	156542271	156542477	14chr11	57192283	57192586	14chr11	57260982	57261223	9chr11	59333692	59333913	15chr1	160070906	160071097	15chr1	160346340	160346573	16chr11	60386246	60386457	15chr1	161067410	161067691	14chr11	61103774	61103954	13chr1	161172025	161172278	11chr11	61544039	61544226	11chr11	61559925	61560272	12chr11	64510361	64510615	13chr11	65341676	65341914	15chr11	66035750	66036090	12chr11	66360432	66360731	11chr11	67122033	67122355	15chr11	67124420	67124592	15chr11	68065262	68065557	13chr1	168195099	168195375	14chr1	169337043	169337423	16chr11	70042263	70042456	15chr11	72533155	72533499	15chr11	73036112	73036398	11chr11	77850611	77850868	15chr1	179262727	179263026	14chr1	182585239	182585551	16chr11	95522728	95523019	12chr1	201083289	201083508	12chr1	202162014	202162228	14chr1	203236432	203236701	13chr1	203258708	203258931	15chr1	204332816	204333046	14chr1	20539420	20539757	12chr1	205498145	205498384	13chr1	208305626	208305807	14chr1	210580626	210580831	12chr12	110035560	110035769	15chr12	111020977	111021362	12chr12	117354631	117354862	13chr1	212003983	212004288	12chr12	125165958	125166164	12chr1	212838662	212838891	11chr12	131355471	131355674	14chr1	21581780	21582048	15chr12	1714403	1714659	13chr12	2144141	2144359	14chr12	27175378	27175609	12chr1	228327808	228328064	13chr1	231664120	231664419	15chr12	31899814	31899996	12chr1	2323031	2323323	13chr1	234820029	234820225	14chr1	241774376	241774665	13chr1	24306862	24307067	13chr12	48213766	48214030	13chr12	49730848	49731077	13chr12	50222204	50222498	12chr12	50469055	50469267	13chr12	50505673	50505893	12chr12	52541207	52541459	14chr12	52721348	52721606	15chr12	53815352	53815649	13chr12	56122793	56123131	14chr12	56652088	56652392	14chr12	57381346	57381640	13chr12	57852939	57853158	12chr12	57856505	57856711	14chr12	57914298	57914597	14chr1	26362456	26362725	13chr12	656126	656387	13chr1	26633062	26633293	14chr1	26743573	26743774	13chr1	27004001	27004262	12chr12	74931476	74931738	11chr12	77459363	77459622	11chr1	27930595	27930862	14chr1	28696032	28696326	10chr12	96608971	96609262	12chr1	31192141	31192418	14chr13	24152824	24153125	11chr13	31248120	31248492	16chr1	33446767	33447081	14chr1	33833424	33833648	13chr1	34098741	34098931	11chr13	49875382	49875594	13chr13	50367039	50367394	12chr13	52157896	52158104	11chr1	36616425	36616664	11chr1	36626534	36626815	14chr1	36866688	36866960	14chr1	38230037	38230211	12chr13	91519629	91519862	11chr1	40626859	40627143	11chr14	100704289	100704507	15chr14	100704991	100705278	16chr14	105175057	105175241	13chr14	105234873	105235208	14chr14	105837195	105837438	14chr1	41174705	41175042	13chr1	41418187	41418466	12chr14	23525450	23525724	11chr14	24610847	24611149	11chr14	50528499	50528710	11chr14	57046342	57046659	11chr14	74829113	74829319	11chr14	75413144	75413328	12chr14	75535918	75536171	11chr1	47799836	47800141	14chr14	78227359	78227689	15chr14	96566964	96567222	14chr15	101548640	101548871	16chr15	31507684	31507962	11chr15	40391020	40391245	11chr15	40408053	40408231	13chr15	40799291	40799538	13chr15	40971333	40971568	14chr1	54411093	54411407	10chr15	52432110	52432403	12chr15	63189227	63189472	16chr15	63385842	63386022	17chr15	64183298	64183512	12chr15	64455115	64455400	11chr15	65186071	65186434	14chr15	65677978	65678183	13chr15	67155277	67155515	15chr15	67316767	67317011	14chr15	67394548	67394842	11chr15	71407597	71407853	13chr15	75165513	75165751	12chr15	75182217	75182482	13chr15	75494176	75494461	13chr15	76603657	76603922	11chr15	84811655	84811849	15chr15	85197985	85198235	14chr15	90836669	90836855	16chr15	91203795	91204045	14chr16	11036260	11036545	12chr16	15993514	15993780	14chr16	22199917	22200191	12chr16	2264905	2265235	15chr16	23681517	23681712	14chr16	27561290	27561584	13chr16	28936919	28937277	13chr16	2918059	2918364	13chr16	30007300	30007600	14chr16	30032944	30033110	12chr16	30087124	30087305	14chr16	30483811	30484070	13chr16	31045542	31045792	13chr16	31888859	31889048	13chr16	4380368	4380601	12chr16	53469372	53469583	11chr16	56903520	56903747	11chr16	575612	575852	14chr16	57662258	57662588	14chr16	58529054	58529296	15chr16	67194813	67195063	12chr16	67499759	67499934	13chr16	67867701	67867966	13chr16	70438249	70438498	17chr1	67390735	67391070	15chr16	74743312	74743626	9chr16	75182207	75182437	16chr16	84706025	84706183	12chr16	89557178	89557449	14chr16	89641071	89641346	13chr1	70671191	70671393	15chr17	17400193	17400387	13chr17	17715650	17715869	14chr17	18218406	18218749	15chr17	1992819	1993004	13chr17	2206890	2207179	12chr17	2300165	2300493	14chr17	28443730	28443977	14chr17	29852433	29852654	13chr17	34274132	34274412	17chr17	36571040	36571238	13chr17	42295813	42296144	14chr17	43449100	43449458	15chr17	45918491	45918800	13chr17	46680005	46680217	13chr17	46696020	46696190	16chr17	46713939	46714142	14chr17	48559955	48560166	14chr17	48739237	48739501	14chr17	48986688	48986907	12chr17	5389990	5390309	15chr17	59494234	59494522	15chr17	61689624	61689846	14chr17	62982012	62982226	16chr17	63096674	63097005	13chr17	64902818	64903083	14chr17	66196951	66197207	14chr17	71088723	71089012	12chr17	71425591	71425801	13chr17	72772370	72772597	12chr17	72978321	72978567	14chr17	73031231	73031494	14chr17	73746037	73746215	14chr17	74502534	74502740	14chr17	77979683	77979846	10chr17	782862	783133	13chr17	79779657	79779909	15chr17	8198699	8199062	15chr18	11908241	11908480	11chr18	12271704	12272132	13chr18	13823919	13824187	15chr18	21002777	21003034	14chr18	29302386	29302669	13chr18	3129320	3129577	13chr18	3177966	3178221	14chr18	32987937	32988130	14chr1	85248202	85248435	14chr18	5868279	5868513	13chr18	596968	597208	14chr18	60092909	60093170	10chr18	76916773	76916994	13chr18	864894	865118	11chr19	10215490	10215701	15chr19	10426485	10426811	14chr19	1103723	1104010	14chr19	11546265	11546557	9chr19	11593448	11593718	14chr19	12551718	12551907	12chr19	13094417	13094702	14chr19	13275354	13275595	16chr19	13958500	13958728	15chr19	16177405	16177771	13chr19	17516299	17516606	11chr19	1767429	1767682	11chr19	18107745	18107970	15chr19	19496421	19496703	13chr19	19754669	19754956	12chr19	2042234	2042496	14chr19	2529607	2529799	13chr19	2950528	2950837	13chr19	34532924	34533184	10chr19	3545149	3545359	13chr19	37178229	37178500	14chr19	40476915	40477176	14chr19	44269439	44269730	14chr19	45681509	45681826	10chr19	46110545	46110796	13chr19	46389978	46390228	12chr19	47141823	47142150	12chr19	47808643	47808873	14chr19	48610686	48610889	15chr19	48866388	48866617	15chr19	49178691	49178921	12chr19	50179507	50179743	12chr19	50440991	50441183	15chr19	51111338	51111557	12chr19	52110362	52110630	13chr19	53970846	53971172	12chr1	95509053	95509218	12chr19	56152218	56152471	13chr19	5773799	5773988	14chr19	58978218	58978574	13chr19	59010805	59011001	14chr19	6280140	6280456	15chr1	9648569	9648773	9chr19	6670073	6670369	14chr19	6737869	6738091	10chr19	8223314	8223611	15chr19	8571022	8571340	9chr1	9934993	9935187	13chr20	18774549	18774803	11chr20	25228709	25228903	13chr20	30157448	30157645	12chr20	30310536	30310845	13chr20	30619311	30619555	10chr20	31350137	31350416	13chr20	32950924	32951172	13chr20	37504154	37504379	12chr20	3762010	3762337	12chr20	44615999	44616250	13chr20	46044190	46044399	15chr20	48225506	48225782	12chr20	49196560	49196780	15chr20	49923352	49923590	12chr20	524299	524564	11chr20	62689192	62689457	13chr20	709044	709293	14chr2	100907632	100907903	12chr2	101769276	101769571	14chr2	10423442	10423705	14chr2	113450177	113450367	13chr2	11828619	11828835	13chr21	30848867	30849056	12chr2	131555114	131555375	17chr2	132250169	132250468	16chr21	34100161	34100427	15chr21	34733602	34733807	13chr21	35296391	35296581	13chr2	135676246	135676560	15chr21	35734167	35734446	15chr21	37802378	37802616	12chr21	40105850	40106124	12chr2	143791912	143792172	12chr21	43916318	43916584	16chr21	45616234	45616514	14chr2	157292680	157292937	13chr2	169868712	169868993	13chr2	170335977	170336369	15chr2	173420285	173420611	15chr2	175113397	175113704	13chr2	197504198	197504408	15chr2	20063232	20063498	14chr2	209118920	209119333	11chr22	17539099	17539359	11chr22	18312850	18313041	10chr22	19166980	19167201	11chr22	19419215	19419416	14chr2	219536580	219536811	13chr22	19710816	19711033	15chr22	20008338	20008640	12chr22	20772518	20772782	13chr22	20850759	20851077	10chr22	21239882	21240111	11chr22	21356296	21356565	12chr22	23284976	23285228	16chr22	24384160	24384403	13chr2	231191769	231192044	15chr22	31365042	31365333	13chr2	232477213	232477539	13chr2	232574987	232575213	13chr22	36026003	36026240	14chr22	36673797	36673999	13chr22	36682622	36682826	13chr22	37374115	37374327	13chr22	38240325	38240650	15chr22	38349486	38349789	11chr22	39266320	39266568	15chr22	41048004	41048286	14chr22	41940402	41940640	14chr22	43011127	43011459	11chr22	46409386	46409605	11chr2	25524377	25524614	12chr2	3522628	3522978	13chr2	38735531	38735789	15chr2	39102922	39103219	14chr2	43456734	43456962	13chr2	46542386	46542793	13chr2	47995125	47995298	13chr2	48133942	48134211	15chr2	61698271	61698643	13chr2	65058511	65058777	13chr2	68073597	68073847	10chr2	71175626	71175893	12chr2	71357665	71357840	14chr2	73312763	73313064	13chr2	85512673	85512892	13chr2	85648498	85648759	13chr2	85906347	85906631	14chr2	85956152	85956332	14chr2	8825938	8826201	12chr2	96658578	96658846	12chr3	122084443	122084658	13chr3	122661729	122662068	12chr3	128745903	128746104	14chr3	128964634	128964821	14chr3	129209139	129209374	12chr3	137893589	137893877	10chr3	141179048	141179230	15chr3	14186097	14186343	13chr3	150945589	150945845	13chr3	169482841	169483090	13chr3	170027984	170028208	13chr3	171678762	171678993	13chr3	183903581	183903902	16chr3	184089378	184089626	14chr3	185000573	185000771	12chr3	185977382	185977618	13chr3	195590004	195590225	12chr3	195923521	195923733	14chr3	196564737	196565067	12chr3	19988470	19988644	11chr3	38035791	38036125	12chr3	39188550	39188769	16chr3	4344901	4345218	15chr3	45883585	45883826	13chr3	46977015	46977314	16chr3	48956094	48956391	14chr3	49256061	49256292	13chr3	50311760	50312042	12chr3	51716126	51716368	13chr3	52162204	52162498	14chr3	53229949	53230218	13chr3	53288907	53289161	9chr3	58035007	58035308	12chr3	98241803	98242001	15chr4	107237553	107237776	10chr4	122632777	122632997	14chr4	129112547	129112788	14chr4	15151536	15151793	13chr4	1750445	1750667	15chr4	184020057	184020337	12chr4	184427324	184427614	13chr4	186392530	186392747	13chr4	2538017	2538253	15chr4	40327483	40327679	13chr4	40674984	40675217	15chr4	54774877	54775147	11chr4	677831	678054	13chr4	7805781	7806041	13chr4	78739317	78739546	14chr4	80979959	80980163	15chr4	8305368	8305629	14chr4	83660557	83660803	10chr4	89744834	89744993	13chr5	10635453	10635677	12chr5	1140430	1140758	13chr5	125874889	125875118	13chr5	137371697	137371988	11chr5	137911006	137911320	13chr5	137915047	137915269	15chr5	137977551	137977824	14chr5	138893196	138893396	13chr5	139536753	139536961	12chr5	141071679	141071945	15chr5	141928354	141928663	13chr5	143550118	143550391	12chr5	148761496	148761694	11chr5	159626422	159626681	14chr5	159866160	159866467	14chr5	16465789	16465971	14chr5	173249466	173249750	14chr5	173754380	173754619	15chr5	176001089	176001283	14chr5	176038458	176038610	9chr5	176831412	176831664	14chr5	1792975	1793182	15chr5	179334731	179335044	12chr5	39283480	39283716	15chr5	60954444	60954621	13chr5	70882975	70883238	13chr5	76070437	76070655	14chr5	8457404	8457654	13chr5	87986843	87987029	14chr5	95992848	95993103	15chr5	96271554	96271761	14chr6	105850817	105851146	11chr6	106094321	106094576	12chr6	108883890	108884113	13chr6	111926975	111927254	13chr6	133120097	133120416	15chr6	135144117	135144326	14chr6	136610874	136611170	13chr6	13814449	13814639	10chr6	148880986	148881220	11chr6	150255374	150255629	13chr6	157041012	157041313	11chr6	160940901	160941240	14chr6	16332350	16332589	15chr6	20202247	20202433	13chr6	27577101	27577330	14chr6	28249197	28249453	12chr6	30034837	30035061	14chr6	30737413	30737638	12chr6	30875734	30875959	14chr6	31364717	31364958	15chr6	31744944	31745210	12chr6	32055447	32055635	13chr6	32160552	32160766	12chr6	34214354	34214617	13chr6	36270294	36270513	13chr6	36410573	36410792	14chr6	3742691	3742917	14chr6	41904078	41904304	13chr6	43635041	43635267	14chr6	44443512	44443727	13chr6	52284804	52285110	15chr6	53590688	53590913	14chr6	73972813	73973097	13chr6	79786566	79786860	12chr6	86379612	86379877	12chr6	87864918	87865242	15chr7	100161610	100161822	13chr7	100289382	100289643	11chr7	100425423	100425726	14chr7	100660780	100661018	12chr7	101915552	101915753	13chr7	105711949	105712237	14chr7	112063037	112063306	12chr7	122794373	122794581	11chr7	128523682	128523996	15chr7	130125957	130126167	9chr7	134143841	134144120	14chr7	137583946	137584125	14chr7	150783169	150783375	14chr7	150808202	150808428	12chr7	154997256	154997558	12chr7	24329924	24330191	14chr7	25132987	25133194	15chr7	25605541	25605827	13chr7	26331317	26331582	13chr7	37071544	37071831	14chr7	39773009	39773313	13chr7	39785839	39786046	14chr7	4837811	4838064	14chr7	5588926	5589280	13chr7	5862558	5862822	12chr7	63774220	63774503	15chr7	6475339	6475565	14chr7	65187320	65187553	13chr7	66836817	66837036	12chr7	73236870	73237173	12chr7	76145321	76145577	14chr7	90032678	90032938	13chr7	99160596	99160796	15chr8	102149468	102149753	14chr8	114449966	114450213	14chr8	11711507	11711744	14chr8	130951933	130952167	13chr8	141477381	141477571	12chr8	142737184	142737420	13chr8	145735122	145735336	14chr8	22775933	22776219	14chr8	27584813	27584995	12chr8	29939819	29940051	14chr8	30601681	30602000	12chr8	38662856	38663096	18chr8	48872577	48872837	11chr8	52921094	52921275	14chr8	61936115	61936368	15chr8	66557280	66557547	15chr8	68256103	68256366	12chr8	73920856	73921109	12chr8	99129322	99129622	14chr9	101946801	101947149	15chr9	102195931	102196140	14chr9	11273	11474	14chr9	123698885	123699076	13chr9	130186468	130186790	16chr9	130980999	130981318	15chr9	131103705	131103924	15chr9	131419080	131419366	11chr9	132250873	132251134	13chr9	132524895	132525126	11chr9	137000881	137001155	12chr9	139962254	139962500	14chr9	35096145	35096413	13chr9	36166589	36166833	14chr9	37079723	37080047	11chr9	4985116	4985301	12chr9	5437793	5438038	13chr9	5450373	5450592	16chr9	72374874	72375173	15chr9	77567724	77568041	18chr9	94902648	94902881	11chr9	99329132	99329359	9chr9	99382126	99382348	10chrX	11776557	11776805	12chrX	119619859	119620223	11chrX	129194483	129194760	14chrX	13110764	13111039	12chrX	152804981	152805212	11chrX	153639611	153639931	11chrX	24072613	24072912	12chrX	47382818	47383015	15chrX	48901255	48901514	12chrX	54556628	54556884	12chrX	62571132	62571419	14chrX	65266816	65267021	17chr10	102747135	102747386	12chr10	102855353	102855604	13chr10	102902041	102902277	12chr10	104163636	104163835	11chr10	11312579	11312828	15chr10	116581312	116581632	13chr10	120850580	120850801	12chr10	120863564	120863804	13chr10	120966882	120967042	11chr10	15139074	15139287	12chr10	17065459	17065710	13chr10	25210335	25210606	16chr10	26986406	26986719	13chr10	31015820	31016088	14chr10	43133853	43134236	14chr10	45428165	45428455	14chr10	49678231	49678445	13chr10	5931882	5932140	16chr10	75255293	75255576	14chr10	81134419	81134609	13chr10	94516922	94517195	13chr10	96305516	96305776	11chr10	99519214	99519452	13chr10	99674592	99674779	13chr1	101361506	101361749	15chr1	10532492	10532766	13chr1	107599212	107599410	11chr1	109820105	109820356	12chr1	110074968	110075233	13chr11	10326563	10326759	11chr1	110546555	110546811	16chr11	110300660	110300964	12chr11	113906886	113907086	11chr1	111506571	111506870	16chr11	118069043	118069277	12chr11	126310799	126311085	12chr1	112938495	112938819	11chr1	113585270	113585582	12chr1	116138491	116138714	10chr11	180222	180419	13chr11	1859027	1859268	13chr1	11954050	11954240	12chr11	20389177	20389454	14chr1	12107314	12107574	11chr1	1259884	1260196	11chr11	3238478	3238656	12chr11	33037269	33037547	13chr11	33087240	33087397	13chr11	34255657	34255972	14chr11	34460364	34460588	12chr11	35052271	35052541	13chr11	36768667	36768936	12chr11	46148754	46148977	11chr11	46911258	46911492	14chr1	149870977	149871280	13chr1	150949814	150950045	12chr1	151507175	151507409	14chr1	152025124	152025352	12chr1	154452320	154452608	13chr1	154531222	154531421	11chr1	155916079	155916319	15chr1	156659366	156659620	12chr1	156698082	156698340	17chr1	156862085	156862326	12chr11	57227809	57228053	9chr11	576301	576585	12chr11	58984303	58984587	13chr11	60681313	60681668	12chr1	160921244	160921437	10chr1	160960162	160960330	14chr1	161147347	161147671	15chr11	61463490	61463774	13chr11	62192262	62192441	13chr11	62477006	62477300	13chr11	63920583	63920801	13chr11	65307703	65308064	14chr1	16563607	16563809	13chr11	65666768	65666998	13chr11	65679229	65679548	9chr11	65789945	65790143	12chr11	66738330	66738583	13chr11	67771504	67771751	13chr11	69485037	69485260	12chr1	16951486	16951681	10chr11	70049987	70050286	12chr11	71350290	71350537	14chr11	72354065	72354322	10chr1	17287195	17287450	11chr11	73490614	73490856	12chr1	175124100	175124320	10chr11	75243411	75243655	16chr1	1790157	1790347	13chr1	181081667	181081857	13chr1	1840373	1840618	12chr1	186051494	186051691	12chr11	86304050	86304277	15chr1	1891541	1891731	9chr11	9770064	9770344	13chr1	200452716	200452908	14chr1	201431062	201431335	10chr1	201762209	201762417	11chr1	201978684	201978918	11chr1	202153866	202154141	10chr1	202522795	202523101	14chr1	204096813	204097084	10chr1	204176279	204176463	11chr1	206234488	206234669	11chr1	206310399	206310600	14chr1	20901756	20901977	11chr1	209846606	209846953	11chr12	104752249	104752473	10chr12	108908800	108909037	13chr12	109248267	109248564	16chr12	110481058	110481280	9chr12	110562077	110562320	13chr1	211828575	211828810	12chr12	122155043	122155254	11chr12	125198250	125198523	18chr12	125477485	125477660	14chr12	12808874	12809149	15chr12	131310918	131311177	11chr1	213224420	213224706	12chr12	132380602	132380884	12chr12	13408742	13408926	14chr1	214502249	214502478	13chr1	227730276	227730513	14chr12	2921625	2921899	13chr1	234634909	234635116	12chr1	235003067	235003335	14chr1	23871241	23871492	12chr1	243419193	243419517	13chr1	248100252	248100519	15chr12	50056821	50057029	15chr12	50419292	50419563	12chr12	52550664	52550832	13chr12	53607175	53607380	11chr12	53739013	53739289	13chr12	55378420	55378696	12chr12	56040044	56040282	14chr12	56334032	56334290	11chr12	56435490	56435715	13chr12	56858132	56858357	11chr12	58334892	58335197	14chr12	6304911	6305137	12chr12	64173645	64173953	13chr12	65950588	65950754	14chr1	26796880	26797089	12chr1	26857683	26857939	12chr12	7013803	7014172	14chr1	28764498	28764745	10chr12	8801292	8801539	11chr12	89918430	89918702	14chr1	29111626	29111854	12chr12	92933089	92933317	14chr12	96429339	96429674	12chr12	98791346	98791565	13chr12	98987283	98987497	13chr13	100150779	100151016	13chr13	100258966	100259255	12chr13	103046821	103047070	14chr13	109568091	109568351	10chr13	113260710	113260962	13chr1	31384254	31384474	12chr13	20175907	20176200	12chr13	21286824	21287021	12chr1	32129530	32129690	14chr13	21635647	21635941	12chr13	22010044	22010263	16chr1	32479281	32479598	10chr13	27825333	27825555	13chr1	32800749	32801057	10chr1	32914014	32914248	14chr1	3341335	3341515	13chr13	49136519	49136712	11chr13	50571335	50571653	15chr1	3827456	3827695	12chr1	38325142	38325424	13chr13	95014753	95014959	13chr13	96032963	96033231	13chr1	40204370	40204664	12chr1	40367916	40368180	14chr14	100784622	100784842	14chr14	22025684	22026020	11chr14	23292042	23292259	10chr14	23298933	23299206	13chr14	23564629	23564897	14chr1	44229212	44229398	10chr1	44412180	44412486	14chr1	44471416	44471597	11chr14	59655190	59655544	14chr1	46010724	46010895	10chr14	64108181	64108420	7chr14	68038055	68038270	12chr14	69130263	69130479	15chr14	69310681	69310918	10chr14	76120523	76120750	15chr15	20561660	20561886	11chr15	23032881	23033135	14chr15	34630185	34630477	13chr15	34659766	34660091	13chr15	39109990	39110242	12chr15	40331265	40331513	14chr15	40882006	40882305	12chr15	41198595	41198804	13chr15	42264849	42265108	14chr1	54355314	54355641	12chr15	44719177	44719460	14chr15	45747091	45747343	11chr15	45748868	45749088	10chr15	52395718	52396016	14chr15	58813696	58814041	13chr15	59042028	59042385	16chr15	63902441	63902654	11chr15	65282161	65282439	15chr15	65477846	65478127	12chr15	65579123	65579410	14chr15	70047982	70048277	13chr15	70053312	70053528	12chr15	70805203	70805380	15chr15	72492114	72492380	11chr15	74913486	74913689	11chr15	75162430	75162633	15chr15	75930758	75930970	11chr15	76443350	76443561	12chr15	79296844	79297075	15chr15	85421838	85422040	12chr15	85839875	85840089	15chr15	90514826	90515036	13chr15	91388455	91388689	12chr15	93374920	93375188	16chr1	59347268	59347486	13chr1	60225188	60225424	13chr16	10837532	10837852	11chr16	16115959	16116202	15chr16	22692088	22692309	13chr16	24855903	24856117	14chr16	25059953	25060297	15chr16	28519423	28519660	12chr16	30103869	30104133	13chr16	30382320	30382589	15chr16	3070833	3071078	15chr16	30953330	30953553	12chr16	46427652	46427773	10chr16	48443962	48444206	10chr16	5083827	5084015	11chr16	5147537	5147795	13chr16	56945751	56945978	14chr16	58277421	58277656	13chr16	58370674	58370849	14chr16	66440388	66440621	14chr16	66901815	66901972	13chr16	67345623	67345927	16chr1	66749443	66749729	11chr16	68297931	68298161	11chr16	68738300	68738499	12chr16	71644930	71645174	13chr1	68001169	68001374	10chr16	83986596	83986929	12chr16	85350052	85350332	13chr16	88509476	88509743	9chr16	88593864	88594165	13chr16	88628438	88628688	10chr16	88851926	88852147	12chr16	88924182	88924453	13chr16	89043458	89043668	14chr16	9102654	9102878	12chr17	11900578	11900941	13chr17	1626398	1626633	10chr17	16356597	16356818	11chr17	16492647	16492884	11chr17	16868042	16868258	11chr17	17349561	17349811	15chr17	17614242	17614482	10chr17	18128600	18128830	13chr17	25896382	25896584	14chr17	28257204	28257422	14chr17	30263854	30264172	11chr17	30580181	30580339	10chr17	30646532	30646753	12chr17	32527014	32527215	10chr17	36600424	36600619	13chr17	36833372	36833546	10chr17	36891446	36891643	14chr17	36908734	36908950	14chr17	37363310	37363534	9chr17	38347943	38348199	14chr17	3868468	3868669	11chr17	4118699	4118923	12chr17	41411597	41411854	13chr17	41633615	41633924	13chr17	4269725	4269983	12chr17	4316517	4316748	13chr17	43226574	43226787	14chr17	43296342	43296572	13chr17	43717794	43717990	13chr17	44331273	44331498	14chr17	48194617	48194884	11chr17	48227252	48227482	10chr17	4870977	4871260	12chr17	49021986	49022225	12chr17	57696829	57697074	13chr17	62409406	62409642	10chr17	65386835	65387102	14chr17	65713803	65713987	11chr17	65959446	65959645	11chr17	70982304	70982555	13chr17	71306799	71307118	13chr17	71308173	71308428	12chr17	72172836	72173099	13chr17	72206616	72206804	14chr17	73083688	73083932	10chr17	73110177	73110456	13chr17	73597360	73597660	12chr17	73851438	73851796	16chr17	7476925	7477256	14chr17	74864343	74864544	11chr17	79212932	79213245	13chr17	7977018	7977239	13chr17	79791107	79791333	14chr17	79875859	79876102	14chr17	80174519	80174849	13chr1	7962861	7963060	14chr18	21756763	21756983	13chr18	51811452	51811685	15chr18	5237913	5238171	13chr18	55989271	55989534	13chr18	56161101	56161353	10chr18	56806821	56807114	14chr18	57572087	57572299	9chr18	61637125	61637343	11chr18	67956139	67956413	10chr18	68101166	68101390	11chr18	71959251	71959601	12chr1	87597505	87597710	15chr1	90228616	90228813	12chr1	90372257	90372527	16chr19	11266469	11266863	14chr19	11607216	11607457	12chr19	12662219	12662524	12chr19	1266982	1267273	9chr19	1279809	1279975	12chr19	12834656	12834891	13chr19	13172257	13172445	13chr19	13265070	13265310	13chr19	13278297	13278504	17chr19	13371945	13372254	13chr19	14459334	14459503	12chr19	14583033	14583299	13chr19	18439233	18439467	12chr19	18699486	18699678	13chr19	1929314	1929562	12chr19	21950269	21950535	11chr19	2236650	2236927	10chr19	2648573	2648779	16chr19	267397	267630	12chr19	33634903	33635097	14chr19	35168326	35168678	15chr19	36605733	36605954	14chr19	3700528	3700748	13chr19	37569093	37569451	13chr19	39612717	39612966	13chr19	40926631	40926845	11chr19	41082610	41082860	13chr19	41710267	41710447	15chr1	94279616	94279806	12chr1	94309901	94310105	12chr19	4374581	4374893	12chr19	44205569	44205825	12chr19	44331320	44331588	13chr19	45245171	45245420	14chr19	45257647	45257960	14chr19	46026927	46027223	16chr19	47016832	47017051	14chr19	47539049	47539313	12chr19	49474219	49474452	14chr19	50050266	50050533	14chr19	50828927	50829175	12chr19	50844421	50844674	12chr19	51320780	51321070	14chr19	51898725	51898937	9chr19	52643011	52643261	11chr19	53426481	53426739	12chr19	54712022	54712343	17chr1	9551174	9551417	12chr19	56150466	56150677	13chr19	58111031	58111357	10chr19	58125455	58125694	12chr19	58193160	58193441	13chr19	6633264	6633458	14chr19	7405991	7406234	14chr19	7701829	7702145	11chr19	9906872	9907117	14chr20	17661793	17662058	12chr20	23128534	23128828	13chr20	26189932	26190072	15chr20	30091008	30091201	10chr20	30150716	30150980	12chr20	30160597	30160870	13chr20	32045042	32045278	15chr20	32717542	32717731	12chr20	33894798	33895132	10chr20	35806726	35806915	14chr20	35807529	35807819	13chr20	36156131	36156469	15chr20	36793723	36793920	12chr20	36849527	36849785	13chr20	37676741	37676977	13chr20	42378429	42378644	13chr20	43017175	43017424	12chr20	43150577	43150860	14chr20	44761312	44761546	12chr20	44935750	44935969	12chr20	4573245	4573533	12chr20	45962949	45963186	12chr20	48402382	48402618	12chr20	48802849	48803074	11chr20	49057326	49057692	13chr20	50211632	50211834	16chr20	52228117	52228353	12chr20	52239931	52240172	10chr20	54967388	54967634	11chr20	56812173	56812364	12chr20	60663583	60663801	11chr20	61425404	61425672	12chr20	62066241	62066465	12chr20	633885	634196	15chr2	109744535	109744699	12chr2	11484740	11485033	12chr2	122513131	122513354	14chr2	128155880	128156123	15chr2	128284063	128284301	11chr2	128349124	128349298	12chr21	35882905	35883143	11chr21	36260322	36260594	15chr2	136996371	136996694	14chr21	43729817	43730071	9chr21	43757977	43758242	14chr21	43870116	43870390	13chr21	44013403	44013620	11chr21	44596825	44597011	13chr21	45066556	45066787	15chr2	145115618	145115829	14chr21	45557879	45558139	12chr21	46975906	46976225	14chr21	47240534	47240777	14chr2	159975172	159975371	11chr2	171786789	171787101	12chr2	173892272	173892439	15chr2	174313985	174314194	10chr2	175352001	175352266	13chr2	177356013	177356269	12chr2	178077821	178078083	15chr2	191885075	191885323	12chr22	18558837	18559118	14chr2	218933566	218933790	16chr2	219860833	219861099	11chr22	20193746	20193981	13chr2	220406507	220406774	15chr22	22067518	22067779	13chr22	22384994	22385286	15chr2	223555371	223555565	13chr22	25858557	25858755	12chr22	31030497	31030696	13chr2	231454241	231454472	14chr22	31886543	31886796	9chr2	232422640	232422875	13chr22	33698087	33698277	9chr2	238480702	238480910	13chr22	38614636	38614880	12chr22	40766461	40766721	14chr22	41972935	41973166	11chr22	42093121	42093418	13chr22	42196484	42196734	12chr22	42337307	42337543	13chr22	46959305	46959495	12chr2	2617197	2617433	15chr2	26173925	26174223	14chr2	27926704	27926889	11chr2	32288481	32288730	16chr2	44588870	44589134	12chr2	60809016	60809265	10chr2	64873503	64873824	12chr2	65233748	65233980	15chr2	68978961	68979123	14chr2	69786927	69787161	12chr2	69870987	69871236	11chr2	69941872	69942051	12chr2	70331776	70332016	12chr2	70529107	70529385	13chr2	70781237	70781490	15chr2	70875069	70875260	10chr2	73053027	73053361	14chr2	84542031	84542281	11chr2	96926224	96926549	15chr2	97173803	97174039	13chr2	97215764	97216037	10chr2	97626190	97626408	13chr2	98313814	98314008	13chr3	10234675	10234939	13chr3	113465464	113465708	11chr3	119513996	119514239	15chr3	122252739	122253025	14chr3	124531707	124531934	12chr3	129345600	129345823	11chr3	132378993	132379254	13chr3	135915208	135915520	17chr3	140950431	140950680	13chr3	14244987	14245252	13chr3	148990995	148991194	14chr3	14962715	14962950	13chr3	149939111	149939317	15chr3	150863875	150864146	13chr3	15149631	15149848	16chr3	15309571	15309759	15chr3	15689218	15689492	12chr3	160167335	160167653	13chr3	172390978	172391221	10chr3	184261839	184262030	12chr3	185378296	185378474	12chr3	189679401	189679614	13chr3	194033446	194033697	15chr3	194354088	194354388	12chr3	195621604	195621783	12chr3	196230535	196230745	14chr3	32147846	32148108	13chr3	34053617	34053902	13chr3	38178749	38179073	10chr3	39231379	39231560	10chr3	39424666	39425016	16chr3	40622082	40622311	14chr3	42141338	42141580	12chr3	45175370	45175579	14chr3	47029311	47029529	13chr3	48477481	48477647	12chr3	4872822	4873154	14chr3	49027152	49027410	13chr3	50159058	50159297	13chr3	52178384	52178577	13chr3	61235691	61235936	13chr3	67704785	67705093	13chr3	69355577	69355837	10chr4	108658209	108658481	13chr4	122618209	122618528	13chr4	1345724	1345907	11chr4	13550613	13550802	13chr4	140097595	140097865	14chr4	146101607	146101902	13chr4	1630916	1631144	12chr4	16918423	16918636	13chr4	183727524	183727853	11chr4	29246	29457	13chr4	2965094	2965286	14chr4	38677173	38677449	13chr4	40201771	40202043	13chr4	48965154	48965388	12chr4	6676457	6676766	13chr4	6717719	6718018	10chr4	6919434	6919678	15chr4	74570273	74570525	11chr5	131762224	131762559	15chr5	132446971	132447195	14chr5	132759557	132759766	13chr5	138775078	138775397	11chr5	140420782	140421009	12chr5	142975885	142976111	14chr5	156755463	156755721	14chr5	157375858	157376039	12chr5	159935902	159936068	11chr5	162864294	162864611	14chr5	167696384	167696608	12chr5	171533858	171534119	13chr5	172720967	172721219	12chr5	177019134	177019390	10chr5	43008725	43008910	13chr5	52095894	52096138	9chr5	58182512	58182785	13chr5	61699687	61699954	13chr5	657748	657963	13chr5	66462056	66462289	13chr5	74807581	74807771	11chr5	74964918	74965186	13chr5	78053979	78054288	13chr5	9544648	9544897	13chr6	106808335	106808592	15chr6	108169511	108169794	11chr6	108615949	108616323	14chr6	111912720	111912933	13chr6	128841681	128841931	13chr6	136965374	136965643	13chr6	137910593	137910798	11chr6	139446115	139446359	15chr6	150070529	150070913	13chr6	154623346	154623524	12chr6	160211289	160211483	11chr6	160389717	160390081	14chr6	167188758	167188992	9chr6	167702172	167702478	13chr6	24775183	24775471	12chr6	26924640	26924865	15chr6	29815894	29816116	11chr6	30658803	30659018	10chr6	3251778	3251951	13chr6	32764831	32765123	11chr6	33216130	33216393	14chr6	34131646	34131846	11chr6	36648247	36648460	11chr6	37178266	37178519	13chr6	37293588	37293840	14chr6	44187197	44187443	10chr6	52929520	52929804	11chr6	53302445	53302655	10chr6	6677138	6677350	13chr6	71665623	71665892	13chr6	8085310	8085541	14chr7	101186187	101186367	12chr7	104909418	104909725	11chr7	105817142	105817398	9chr7	1083780	1084047	12chr7	116164712	116164986	14chr7	128379234	128379554	13chr7	130581782	130582017	12chr7	134853890	134854166	12chr7	140098202	140098417	11chr7	148702936	148703196	12chr7	150595651	150595905	11chr7	154958517	154958732	12chr7	16707700	16707925	14chr7	20811	21069	13chr7	23053552	23053826	15chr7	2508590	2508805	12chr7	25934985	25935298	12chr7	30387664	30387958	13chr7	32886305	32886570	15chr7	44280612	44280846	12chr7	45299677	45299895	14chr7	4681713	4681954	16chr7	47294007	47294307	13chr7	47976976	47977193	13chr7	55323297	55323502	14chr7	6116828	6117087	12chr7	64772701	64772968	14chr7	65445849	65446106	13chr7	66461621	66461818	12chr7	73115874	73116115	12chr7	75988592	75988888	14chr7	99102165	99102379	14chr8	10696433	10696713	10chr8	123807302	123807546	12chr8	126346330	126346615	15chr8	126444294	126444514	10chr8	126446906	126447164	16chr8	126525343	126525599	14chr8	141598923	141599196	12chr8	142036944	142037179	12chr8	143528925	143529105	14chr8	144594835	144595025	12chr8	144629454	144629689	13chr8	144816182	144816417	12chr8	145669443	145669762	11chr8	22480459	22480692	12chr8	22766580	22766757	12chr8	27224102	27224373	12chr8	29580574	29580777	14chr8	37557877	37558059	11chr8	38239806	38240051	14chr8	40059339	40059577	13chr8	49838197	49838489	11chr8	49862160	49862384	13chr8	59323643	59323985	12chr8	61325118	61325403	14chr8	74659254	74659468	13chr8	85538445	85538642	12chr8	8594457	8594735	13chr8	8775263	8775529	12chr8	9688558	9688790	12chr8	99953272	99953492	13chr9	100149338	100149551	10chr9	103115158	103115473	11chr9	116172869	116173109	14chr9	116341268	116341520	13chr9	116917573	116917850	10chr9	117373393	117373668	11chr9	119311691	119311899	15chr9	124041126	124041317	9chr9	124312856	124313063	13chr9	130266321	130266517	10chr9	130548816	130549034	11chr9	131930394	131930621	12chr9	132388391	132388573	13chr9	132646663	132646987	11chr9	133557979	133558158	13chr9	134153480	134153697	13chr9	138396543	138396795	10chr9	140339838	140340081	12chr9	35646780	35647001	10chr9	37411364	37411700	13chr9	38088039	38088287	15chr9	44227383	44227542	11chr9	4762590	4762899	15chr9	66458416	66458597	13chr9	67309305	67309640	13chr9	69381587	69381815	16chr9	74383320	74383524	12chr9	97365622	97365871	11chrX	107020498	107020711	13chrX	117479714	117479976	13chrX	128977734	128978037	9chrX	129095329	129095580	11chrX	129473736	129473981	12chrX	133931091	133931335	12chrX	134186110	134186359	13chrX	149369203	149369501	12chrX	150565467	150565667	14chrX	199906	200179	12chrX	23774342	23774571	12chrX	39614876	39615222	14chrX	57147849	57148149	12chrX	67718735	67718956	12chr10	101605633	101605827	11chr10	102638295	102638536	12chr10	102757440	102757672	10chr10	102974557	102974781	12chr10	103362571	103362752	12chr10	105230998	105231246	10chr10	1102577	1102848	14chr10	114074700	114074886	11chr10	114571614	114571928	14chr10	11726815	11727059	15chr10	11799969	11800181	12chr10	118441899	118442129	10chr10	118934422	118934696	13chr10	121028551	121028791	8chr10	121075172	121075375	12chr10	121464095	121464326	11chr10	126150260	126150564	10chr10	134036655	134036866	12chr10	134143386	134143671	14chr10	14644019	14644287	11chr10	22610275	22610534	12chr10	24931930	24932204	13chr10	25369910	25370176	11chr10	43904298	43904551	13chr10	44144183	44144476	13chr10	45960537	45960737	11chr10	45966018	45966404	15chr10	52419933	52420137	10chr10	6193440	6193698	13chr10	6342109	6342397	12chr10	63510722	63510958	9chr10	70821388	70821660	10chr10	72309704	72309882	14chr10	73571789	73572018	12chr10	73656268	73656444	11chr10	76947741	76947979	14chr10	97205709	97205934	11chr10	98047520	98047782	11chr10	98479575	98479909	12chr1	101360276	101360569	10chr1	101701120	101701409	10chr11	102261434	102261680	13chr1	110420095	110420353	10chr11	113176535	113176715	11chr11	114131777	114131999	13chr11	116706728	116707017	13chr11	117014837	117015012	12chr11	117924165	117924392	13chr11	118800980	118801226	11chr11	118827328	118827535	13chr11	118928091	118928378	13chr11	119067654	119067894	13chr11	123117971	123118208	14chr11	12745974	12746214	10chr11	12910354	12910579	11chr11	14443916	14444141	15chr1	117047027	117047308	14chr11	17620608	17620782	11chr11	1846314	1846520	12chr11	18797342	18797531	13chr11	18824285	18824485	10chr11	28129591	28129823	10chr11	2913359	2913626	12chr11	32197831	32198020	14chr11	3476158	3476401	15chr11	36310884	36311069	12chr11	43890534	43890823	16chr1	1447181	1447426	13chr11	44749466	44749661	11chr11	46318498	46318762	12chr1	150540674	150540949	8chr1	150980673	150980990	12chr1	153590152	153590346	10chr1	154580494	154580793	12chr1	154851055	154851221	14chr1	156060586	156060816	11chr11	5706122	5706345	12chr1	157108126	157108373	11chr11	57434827	57435106	10chr11	57479696	57479956	14chr11	58910077	58910360	12chr11	59590508	59590795	12chr1	159893505	159893702	11chr1	159915356	159915650	13chr1	160055471	160055710	13chr1	160651358	160651571	12chr11	60673861	60674108	15chr1	161179680	161179893	13chr1	16118561	16118763	14chr11	61405937	61406131	11chr11	62264635	62264844	10chr1	16339211	16339561	15chr11	63448839	63449087	9chr11	64217091	64217265	14chr11	64511128	64511327	11chr11	65083125	65083306	13chr11	65640967	65641258	13chr1	165667423	165667727	10chr11	65812667	65812930	11chr11	67397797	67398093	11chr1	168409073	168409316	14chr1	170043824	170044048	12chr11	71802306	71802587	13chr11	71823722	71824004	11chr11	71894506	71894793	12chr11	71949214	71949539	12chr11	72193375	72193595	13chr11	72889113	72889364	11chr11	73587839	73588130	13chr1	17380473	17380750	11chr11	73849696	73849892	11chr11	75095590	75095774	12chr11	76571587	76571899	13chr11	7687061	7687267	12chr1	17746437	17746643	11chr1	178501511	178501737	10chr1	182993082	182993310	12chr1	183250712	183250923	12chr1	183885215	183885473	11chr1	185606033	185606278	13chr11	93451444	93451655	14chr11	9556711	9556968	13chr11	95889353	95889631	9chr1	197744464	197744717	12chr1	198638226	198638502	13chr1	201139781	201140135	11chr1	20478881	20479077	12chr1	205888193	205888379	11chr1	206652905	206653141	13chr1	206730415	206730627	13chr1	206736400	206736654	14chr1	206957858	206958152	11chr1	207378450	207378730	13chr1	210506207	210506431	12chr12	111180550	111180820	13chr1	211786709	211786938	14chr12	120668377	120668695	12chr12	121084808	121085086	14chr12	121454200	121454443	11chr1	212185357	212185551	12chr12	122214143	122214386	12chr12	125399353	125399586	12chr12	125404674	125404895	12chr1	212769703	212769993	13chr12	127865689	127865880	10chr12	15101706	15101925	13chr12	1692719	1692909	14chr1	221881473	221881687	14chr1	225960081	225960279	12chr1	226250365	226250643	14chr1	226496679	226496916	11chr1	231493630	231493909	12chr12	32213594	32213773	10chr1	236162781	236163002	12chr1	236445187	236445474	12chr1	246378304	246378510	10chr12	46465980	46466223	11chr12	49482096	49482314	12chr12	49974358	49974576	11chr12	50038399	50038641	11chr12	50464329	50464558	12chr12	50561146	50561373	10chr12	51762803	51763067	13chr12	54378703	54378925	12chr12	64845734	64845991	13chr12	65996363	65996591	12chr12	68768009	68768239	12chr12	6978632	6978842	11chr12	70446805	70447012	11chr1	27423964	27424160	11chr12	9102433	9102766	13chr12	96792994	96793268	13chr13	101240957	101241225	11chr13	103249167	103249411	10chr13	113528890	113529116	11chr13	28195868	28196178	11chr1	33077790	33078063	12chr13	31736456	31736617	13chr1	33342332	33342487	12chr13	36920839	36921064	13chr13	37633622	37633981	12chr13	46756171	46756430	12chr13	50570736	50570965	14chr13	52025563	52025884	13chr13	52389355	52389520	10chr1	36565257	36565512	10chr13	77554338	77554508	11chr13	95953619	95953900	12chr1	40839304	40839504	10chr14	100574737	100574920	11chr14	105399750	105399957	11chr14	105994761	105994965	12chr1	41961881	41962192	12chr14	21571716	21572014	14chr14	22940625	22940809	12chr14	23057985	23058287	12chr14	34697236	34697468	14chr14	35754655	35754966	8chr14	39572492	39572820	12chr14	39583337	39583601	12chr14	44786172	44786382	13chr14	55272573	55272801	11chr1	46016376	46016599	11chr1	46943103	46943358	14chr14	76451945	76452200	11chr1	47658596	47658814	13chr14	76618743	76619019	11chr14	77251221	77251396	10chr14	89882469	89882710	13chr14	91752802	91753105	11chr14	93153324	93153523	13chr14	93605547	93605836	13chr15	101137081	101137335	15chr1	52520859	52521068	12chr1	52831712	52831946	10chr15	41849895	41850139	11chr15	41896164	41896399	10chr15	42196762	42196980	13chr15	43733148	43733367	11chr15	44119008	44119184	12chr1	54518933	54519224	13chr15	50646969	50647346	13chr15	51973570	51973769	11chr15	52274475	52274715	13chr15	52313405	52313590	11chr15	52519691	52519938	11chr15	52971500	52971761	11chr15	55700459	55700668	14chr15	58749736	58750010	13chr15	59063090	59063368	11chr15	59981387	59981711	11chr15	63758969	63759197	11chr15	65677255	65677424	10chr15	67058159	67058416	15chr15	67368573	67368864	13chr15	67413460	67413722	12chr15	74257125	74257391	10chr15	74614976	74615181	12chr15	74782155	74782402	13chr15	75127669	75127936	13chr15	75280424	75280639	12chr15	75287665	75287858	13chr15	79476754	79477003	11chr15	81594270	81594471	12chr15	85201542	85201775	14chr15	89973987	89974180	11chr15	90190438	90190644	12chr15	90283356	90283575	13chr1	59039466	59039714	12chr15	90844451	90844764	13chr15	98964863	98965115	13chr16	11012679	11012849	13chr16	12130939	12131186	13chr16	14726590	14726817	12chr16	15576177	15576554	11chr16	157110	157286	13chr16	1772222	1772461	12chr16	1993124	1993413	11chr16	21289509	21289739	12chr16	25122888	25123108	12chr16	2563693	2563946	12chr16	29859860	29860083	12chr16	30596973	30597156	14chr16	30913445	30913710	12chr16	30965009	30965285	12chr16	4233169	4233453	14chr16	4363918	4364172	12chr16	4666164	4666420	13chr16	47526881	47527135	15chr1	6498172	6498342	10chr16	5006218	5006411	11chr16	50315925	50316196	13chr16	5142304	5142562	13chr16	54227603	54227840	11chr16	56352251	56352465	10chr16	66349192	66349397	12chr16	66914271	66914529	12chr1	6673590	6673774	14chr16	67678604	67678826	12chr16	68002519	68002747	14chr1	66840318	66840494	11chr16	68573006	68573251	12chr16	68794780	68795030	12chr16	69564604	69564907	14chr16	75600154	75600411	11chr16	75798146	75798328	12chr16	81130004	81130306	11chr16	84544964	84545198	10chr16	84655795	84656034	12chr16	84759746	84759971	10chr16	87096345	87096526	12chr16	87670021	87670244	9chr16	88503846	88504053	12chr16	88748216	88748476	14chr16	88882455	88882671	10chr16	89976744	89976947	12chr16	90182623	90182858	12chr17	1418934	1419223	13chr17	16593288	16593531	10chr17	17258282	17258489	12chr17	17817322	17817511	15chr17	17936805	17937052	15chr17	18908000	18908309	14chr17	21189768	21189986	13chr17	21360446	21360721	11chr17	27916645	27916930	12chr17	28258379	28258633	12chr17	32688572	32688764	15chr17	3539716	3540089	14chr17	3627018	3627189	13chr17	37391982	37392218	11chr17	40346334	40346591	12chr17	4046290	4046512	15chr17	43394715	43395009	10chr17	45898986	45899282	11chr1	74663856	74664166	10chr17	46908203	46908456	13chr17	48075684	48075886	12chr17	5140829	5141139	13chr17	55749346	55749537	12chr17	55910730	55910970	12chr17	56327025	56327285	11chr17	56863765	56863978	12chr17	60555803	60556015	12chr17	62207367	62207550	11chr1	762590	762888	12chr17	62690436	62690659	15chr17	64463201	64463380	13chr17	65289884	65290122	10chr17	65374859	65375074	13chr17	66494221	66494379	13chr17	71188961	71189323	15chr17	7259425	7259595	13chr17	72956854	72957090	12chr17	7340342	7340538	16chr17	73408715	73408932	14chr17	73743932	73744171	11chr17	74480780	74481110	13chr17	75284895	75285170	15chr17	76250543	76250763	12chr17	76837023	76837235	13chr17	7760372	7760644	12chr17	78193351	78193551	13chr17	78228605	78228884	14chr17	79849372	79849639	10chr17	80163832	80164067	11chr1	78580137	78580351	13chr18	20143144	20143342	11chr18	23670625	23670879	9chr1	8272105	8272336	14chr18	33038947	33039135	13chr18	33767296	33767533	13chr18	3595437	3595765	14chr18	47385120	47385401	11chr18	47792764	47792992	12chr18	52901930	52902156	13chr18	52988999	52989248	15chr18	55549112	55549296	13chr18	56431591	56431794	14chr18	60152907	60153162	13chr18	61603406	61603636	13chr18	74515767	74516022	12chr18	74777477	74777716	11chr1	89990255	89990442	11chr19	10223756	10224016	12chr19	10679635	10679878	12chr19	1099553	1099753	13chr19	12792581	12792822	12chr19	13088705	13088953	12chr19	13949877	13950131	11chr19	14016882	14017072	11chr19	14550854	14551154	13chr19	14590930	14591181	13chr19	14629083	14629284	13chr1	91487661	91487919	12chr19	16449836	16450016	11chr19	1648459	1648755	11chr19	16882440	16882638	12chr19	17378133	17378388	10chr19	17530622	17530833	13chr19	17830179	17830397	8chr19	17966459	17966689	11chr19	18414293	18414527	12chr19	18631101	18631304	9chr19	20958906	20959202	11chr19	21541603	21541861	10chr19	2579457	2579733	13chr19	32864747	32864950	13chr1	9335625	9335873	15chr1	9349167	9349327	12chr19	3618555	3618804	13chr19	38734554	38734756	14chr19	38747152	38747403	11chr19	39339943	39340253	14chr19	40561984	40562283	12chr19	4058329	4058622	12chr19	41827709	41827994	11chr19	4204678	4204833	14chr19	42077044	42077285	12chr19	4233439	4233696	11chr19	42747264	42747520	11chr19	44645533	44645816	13chr19	45959022	45959240	11chr19	46498375	46498603	11chr19	46580901	46581111	12chr19	47117436	47117641	12chr19	47750705	47750894	12chr19	48210610	48210841	13chr19	49112067	49112300	11chr19	49535000	49535227	9chr19	49947023	49947236	11chr19	50167832	50168081	11chr19	50964502	50964707	11chr19	52642492	52642714	11chr19	53868851	53869140	10chr19	54527216	54527401	13chr19	56683303	56683530	12chr19	56821028	56821232	12chr19	5687176	5687390	10chr19	57702655	57702936	10chr19	58071106	58071331	12chr19	58326212	58326445	12chr1	9777704	9777899	11chr19	8008677	8009013	12chr20	10427738	10427938	13chr20	13619435	13619747	11chr20	19928611	19928794	13chr20	22672360	22672598	11chr20	25218498	25218741	10chr20	2821229	2821441	9chr20	3057468	3057673	10chr20	31109556	31109823	12chr20	32255770	32255979	13chr20	33301276	33301578	11chr20	33461935	33462189	10chr20	3691628	3691810	11chr20	414307	414580	13chr20	43540141	43540363	12chr20	43919593	43919813	10chr20	44221264	44221480	13chr20	44739904	44740143	13chr20	45039641	45039828	12chr20	45310059	45310289	12chr20	45985412	45985646	13chr20	45986284	45986584	14chr20	47131953	47132294	11chr20	47444628	47444929	13chr20	49461274	49461523	13chr20	50416734	50416985	11chr20	52259713	52259963	15chr20	52523674	52523918	11chr20	56827158	56827373	11chr20	57978817	57979008	10chr20	62127512	62127740	12chr20	8834492	8834707	13chr2	100908616	100908827	14chr2	102918333	102918609	15chr2	10633762	10634030	11chr2	109830511	109830808	13chr2	111926143	111926341	12chr2	111966629	111966920	10chr2	112456515	112456802	14chr2	113551824	113552017	12chr2	120399194	120399438	13chr2	128458142	128458414	16chr21	30391545	30391718	13chr21	31029123	31029347	12chr21	35014076	35014342	11chr21	35134047	35134265	12chr21	35320107	35320321	10chr21	35747663	35747973	10chr21	35899162	35899360	12chr21	35987589	35987817	14chr2	137181110	137181314	11chr21	37876667	37876838	10chr21	40159498	40159730	13chr21	40817692	40817975	12chr21	44847130	44847375	11chr21	44914523	44914743	13chr21	45188095	45188288	12chr21	46312769	46312947	12chr21	46369738	46369938	14chr2	146509279	146509517	15chr2	153057127	153057406	12chr2	159757836	159758064	13chr2	171092564	171092837	10chr2	17699587	17699881	13chr2	188312900	188313166	14chr2	191399283	191399624	12chr2	191746650	191746865	10chr2	201934060	201934314	11chr2	206544774	206545063	12chr2	219433266	219433588	15chr22	19958112	19958272	12chr2	220042533	220042872	13chr22	21910376	21910577	11chr22	21984251	21984555	12chr22	22549854	22550050	13chr22	24643617	24643817	11chr22	25453287	25453504	10chr22	29641581	29641783	10chr22	29866413	29866650	11chr22	30217321	30217634	12chr22	32366649	32366904	14chr2	232395115	232395358	11chr22	35979149	35979398	12chr22	36779255	36779547	16chr2	237478273	237478520	14chr22	38213724	38213920	10chr22	38902427	38902693	13chr2	239335492	239335747	10chr22	39930809	39931038	12chr2	240196936	240197142	13chr2	240866813	240867018	9chr22	40895225	40895455	12chr2	241083265	241083480	10chr2	241453356	241453548	12chr2	242036486	242036737	13chr2	242089671	242089954	11chr22	43176149	43176361	10chr22	43485341	43485622	13chr22	43781791	43781989	10chr22	45131766	45131997	10chr22	45659714	45659916	10chr22	46041498	46041729	12chr22	46390809	46391030	12chr2	27632438	27632650	15chr2	30369314	30369517	13chr2	37193649	37193911	13chr2	38342455	38342633	13chr2	39351489	39351677	11chr2	47168796	47168993	11chr2	55277522	55277832	13chr2	58468396	58468698	11chr2	61670825	61671103	14chr2	63277934	63278130	14chr2	65100609	65100863	11chr2	65414449	65414675	9chr2	69869802	69870047	11chr2	70417755	70417994	11chr2	85726289	85726505	13chr2	95539994	95540200	12chr2	9748088	9748326	12chr2	99001583	99001818	12chr2	99081429	99081672	13chr2	99953604	99953848	16chr3	107148757	107149011	13chr3	112280612	112280934	13chr3	112770174	112770364	11chr3	115538676	115538917	12chr3	119396117	119396354	15chr3	122134978	122135204	11chr3	12235069	12235223	10chr3	122398050	122398272	9chr3	129001619	129001876	14chr3	134083036	134083347	13chr3	134513780	134514070	10chr3	136732813	136733051	14chr3	137787506	137787715	12chr3	138313584	138313829	13chr3	141741246	141741485	14chr3	149530815	149531017	11chr3	16219839	16220106	12chr3	16288889	16289063	14chr3	169762740	169763025	12chr3	171847793	171848014	11chr3	17783932	17784196	9chr3	180707236	180707568	13chr3	182876329	182876582	13chr3	184063521	184063755	12chr3	195586274	195586492	13chr3	196065308	196065583	9chr3	25623766	25623975	13chr3	28282927	28283242	13chr3	32511341	32511627	11chr3	32551635	32551935	14chr3	33156692	33156941	10chr3	36954046	36954276	11chr3	38016201	38016424	11chr3	46139985	46140189	11chr3	49761396	49761745	15chr3	5054952	5055195	15chr3	50638570	50638807	11chr3	5099547	5099752	12chr3	52017379	52017616	13chr3	53254017	53254176	13chr3	63898090	63898338	11chr4	100815462	100815647	12chr4	100867663	100867864	12chr4	120134204	120134460	9chr4	128619990	128620296	13chr4	140476410	140476650	11chr4	14858073	14858308	11chr4	148788049	148788212	9chr4	151788231	151788494	11chr4	164465619	164465819	10chr4	183859302	183859561	11chr4	184580221	184580448	13chr4	1872226	1872433	11chr4	2937815	2938109	10chr4	40207138	40207320	10chr4	4109289	4109493	13chr4	47465583	47465788	12chr4	57666352	57666561	15chr4	6548393	6548587	12chr4	75397686	75397915	11chr4	76649664	76649955	13chr4	78016587	78016816	13chr4	87843621	87843828	14chr4	89300053	89300350	12chr4	90032547	90032843	13chr5	107978059	107978257	15chr5	112312286	112312634	10chr5	1174512	1174763	11chr5	131803394	131803628	13chr5	136105837	136106050	10chr5	139040736	139040976	10chr5	139523885	139524061	12chr5	142625660	142625898	11chr5	14727554	14727737	10chr5	171720551	171720744	13chr5	17317158	17317437	16chr5	175395118	175395369	11chr5	175788682	175788907	12chr5	175843497	175843709	13chr5	176005413	176005651	10chr5	176784697	176784953	13chr5	176830770	176831069	12chr5	177503145	177503394	14chr5	180611152	180611386	15chr5	34924095	34924376	13chr5	37413166	37413347	12chr5	392657	392956	17chr5	43389867	43390093	11chr5	50258839	50259025	13chr5	5351560	5351794	13chr5	54522301	54522519	12chr5	60597701	60597874	13chr5	76788209	76788497	10chr5	78429477	78429743	13chr5	90279072	90279280	12chr5	92956320	92956510	13chr5	9361965	9362244	13chr6	108395814	108396092	13chr6	109059212	109059452	13chr6	109804273	109804519	12chr6	11382422	11382649	11chr6	125513524	125513738	10chr6	138132626	138132866	13chr6	14211406	14211673	11chr6	144263338	144263563	13chr6	14711360	14711693	10chr6	150739363	150739618	12chr6	155635546	155635838	12chr6	158185556	158185854	13chr6	158641945	158642142	12chr6	16277630	16277841	12chr6	18264951	18265190	14chr6	26027220	26027506	15chr6	26066216	26066411	13chr6	26172016	26172397	13chr6	28323949	28324183	15chr6	2862984	2863302	11chr6	31105319	31105506	11chr6	31160374	31160540	11chr6	31459740	31459946	12chr6	31802394	31802635	11chr6	31830678	31830988	10chr6	3279859	3280058	14chr6	33538989	33539190	12chr6	33755913	33756201	13chr6	41559793	41560006	13chr6	41570818	41571047	12chr6	41754223	41754493	12chr6	43337380	43337641	11chr6	44205258	44205492	10chr6	44272583	44272828	12chr6	47065183	47065400	12chr6	80579382	80579567	12chr6	82462347	82462603	12chr6	84140788	84141056	12chr6	84569204	84569509	12chr6	88410510	88410803	12chr6	90007719	90007986	14chr7	100005294	100005483	12chr7	100492505	100492720	12chr7	100728612	100728880	12chr7	100835831	100836038	10chr7	101007272	101007528	11chr7	101151155	101151361	11chr7	105752896	105753141	12chr7	106183027	106183246	14chr7	107786707	107786920	10chr7	112509307	112509527	12chr7	116166185	116166406	11chr7	129041335	129041620	16chr7	131192734	131192955	11chr7	137693608	137693823	11chr7	142960435	142960649	12chr7	1472127	1472374	14chr7	157179970	157180155	12chr7	23301547	23301733	12chr7	23374252	23374532	17chr7	25219763	25220156	12chr7	25891314	25891547	13chr7	30325653	30325854	12chr7	31014470	31014699	12chr7	34787996	34788273	11chr7	36429149	36429412	16chr7	38255426	38255615	13chr7	39629384	39629620	13chr7	50245026	50245239	13chr7	50602614	50602851	11chr7	5572538	5572836	10chr7	63767663	63767923	12chr7	7139252	7139506	13chr7	7216788	7216987	11chr7	73393474	73393724	11chr7	73831819	73831999	11chr7	826104	826316	12chr7	83097648	83097901	13chr7	92271515	92271818	13chr7	98030547	98030716	11chr8	103547997	103548350	9chr8	10447717	10447939	13chr8	120879441	120879616	12chr8	121135824	121136065	12chr8	122654498	122654785	13chr8	124172720	124172936	14chr8	12516899	12517156	12chr8	126204131	126204380	13chr8	126656760	126657014	13chr8	144107750	144108029	11chr8	144360802	144361103	13chr8	145909697	145909990	12chr8	18067483	18067724	11chr8	22094870	22095070	12chr8	22446602	22446846	12chr8	27459630	27459829	12chr8	29594985	29595240	11chr8	38088770	38089095	14chr8	41386695	41386879	9chr8	56986926	56987177	14chr8	59521219	59521427	12chr8	6698272	6698455	11chr8	67974394	67974626	12chr8	96814168	96814451	13chr9	100161479	100161682	13chr9	101610877	101611118	11chr9	102581685	102581949	12chr9	108061774	108061985	14chr9	114823569	114823915	14chr9	124049343	124049540	13chr9	125667507	125667776	14chr9	129160869	129161044	11chr9	129544132	129544353	9chr9	129568137	129568344	10chr9	129728190	129728428	12chr9	130324196	130324498	10chr9	130705120	130705338	13chr9	131057658	131057947	10chr9	131397626	131397880	13chr9	131893999	131894243	14chr9	131903735	131903944	16chr9	131938150	131938401	14chr9	135545237	135545503	13chr9	136218889	136219124	11chr9	136658170	136658410	10chr9	139001568	139001772	11chr9	141121285	141121520	12chr9	19457515	19457736	11chr9	33619673	33619839	15chr9	33778829	33779038	13chr9	34074285	34074496	10chr9	34611962	34612226	12chr9	34652190	34652414	10chr9	35096462	35096701	13chr9	73178677	73178862	13chr9	90131246	90131459	12chr9	91615913	91616153	12chr9	94181562	94181785	11chr9	94896257	94896479	11chrX	100662814	100663123	11chrX	109083832	109084105	11chrX	109134191	109134417	11chrX	118925436	118925651	11chrX	122937397	122937652	15chrX	129091355	129091595	11chrX	129226100	129226436	11chrX	130947238	130947440	13chrX	134049123	134049334	10chrX	138295619	138295808	15chrX	152665659	152665841	11chrX	152736346	152736574	13chrX	153770166	153770410	11chrX	154255052	154255342	15chrX	39957119	39957347	11chrX	40126271	40126573	15chrX	41782633	41782988	8chrX	47510345	47510525	8chrX	47886125	47886347	11chrX	48774470	48774725	12chrX	49012561	49012807	13chrX	53462985	53463206	11chrX	70838261	70838497	9chrX	71238391	71238665	14chr10	101535712	101535944	11chr10	101805875	101806111	13chr10	102124649	102124859	11chr10	102728865	102729100	12chr10	103991644	103991808	10chr10	104405733	104406013	12chr10	104677261	104677499	13chr10	104991004	104991253	14chr10	105445949	105446211	11chr10	111683107	111683270	12chr10	111981659	111981943	11chr10	112259257	112259511	13chr10	112262837	112263058	11chr10	121417827	121418030	11chr10	121446066	121446380	13chr10	12307871	12308076	13chr10	126218787	126219044	13chr10	126736697	126736929	12chr10	134266702	134266910	12chr10	135207564	135207802	12chr10	23012289	23012523	12chr10	24485430	24485630	11chr10	30818408	30818780	13chr10	30823534	30823734	10chr10	35415747	35415970	13chr10	43600924	43601220	8chr10	43843978	43844181	9chr10	44882077	44882281	12chr10	48398464	48398716	11chr10	5113974	5114183	12chr10	5882323	5882605	9chr10	61385822	61386064	13chr10	6186652	6186938	10chr10	6281278	6281472	9chr10	64996232	64996429	13chr10	65389264	65389517	15chr10	71168663	71168856	13chr10	71906355	71906597	11chr10	72091532	72091852	12chr10	73144000	73144230	9chr10	74397256	74397488	14chr10	75006768	75006964	11chr10	75692153	75692362	9chr10	79731036	79731310	12chr10	80062945	80063209	11chr10	80956694	80956935	11chr10	82204464	82204714	13chr10	82218796	82219125	12chr10	85631318	85631503	11chr10	88345299	88345484	11chr10	94000298	94000611	11chr10	94194873	94195040	12chr10	95048858	95049058	10chr10	99551784	99551995	13chr1	100232035	100232294	10chr1	1004595	1004831	12chr1	100503565	100503827	9chr1	101704306	101704562	11chr1	110522320	110522587	13chr11	10681291	10681553	13chr1	110855289	110855517	12chr1	111173504	111173727	11chr1	111323545	111323788	13chr11	116147307	116147561	11chr11	117052052	117052221	11chr11	118286519	118286719	14chr11	119209078	119209296	11chr11	119227091	119227328	13chr11	122376507	122376703	13chr11	123173508	123173744	7chr11	125772953	125773184	10chr11	126350653	126350878	10chr11	131543899	131544152	11chr11	132952733	132952987	11chr11	1536738	1537003	12chr1	116012398	116012584	10chr11	16635247	16635441	11chr1	116920495	116920829	13chr11	16923774	16924015	11chr1	118364445	118364669	12chr11	18405333	18405504	12chr1	11898827	11898990	10chr1	11986525	11986710	10chr11	20000281	20000544	13chr11	2000231	2000476	14chr1	120286807	120287041	9chr1	120351629	120351870	10chr11	2555103	2555264	11chr11	2906270	2906500	13chr11	3071090	3071311	14chr11	33879459	33879673	12chr1	144533804	144534084	13chr11	44545147	44545392	12chr11	44638502	44638696	12chr11	45392775	45393018	11chr11	46330214	46330360	10chr1	147400459	147400734	12chr1	149911059	149911286	11chr1	150141250	150141482	12chr1	150592264	150592573	11chr1	151313357	151313630	13chr1	151960943	151961220	12chr11	5301912	5302092	14chr1	153630976	153631249	10chr1	15455110	15455306	11chr1	155112895	155113111	14chr1	156093474	156093778	14chr1	156458434	156458627	9chr1	156586318	156586531	13chr1	156627341	156627498	10chr1	156647902	156648084	13chr1	156859946	156860143	12chr11	568618	568911	11chr1	157347093	157347324	12chr1	158094858	158095081	12chr1	161197229	161197513	12chr11	61213703	61213947	13chr1	16121615	16121872	14chr11	61500975	61501140	12chr1	161708550	161708859	11chr11	61735189	61735369	12chr11	6374708	6374946	12chr11	64018746	64019041	13chr1	164756617	164756854	12chr11	64781563	64781940	14chr11	64972273	64972496	11chr11	64992869	64993191	9chr11	66095833	66096056	11chr11	67572655	67572850	12chr11	67723204	67723410	12chr11	68868935	68869157	13chr11	70079633	70079862	12chr11	71814434	71814642	11chr11	72125295	72125499	10chr11	72395639	72395851	11chr11	72542558	72542831	12chr11	72865237	72865540	15chr1	17338402	17338608	14chr11	73691092	73691310	12chr11	74459772	74460025	11chr11	74916047	74916237	11chr11	74925501	74925677	11chr11	75273687	75273925	11chr11	78127983	78128224	11chr1	181013670	181013961	11chr1	181394106	181394302	10chr1	182751484	182751717	12chr1	182991503	182991732	8chr11	82997000	82997303	13chr11	85955858	85956219	12chr1	19201766	19202005	11chr11	92733752	92733964	10chr1	19337648	19337841	13chr11	95745377	95745576	12chr1	201528826	201529038	11chr1	202129040	202129367	11chr1	203087000	203087250	11chr1	207226289	207226543	11chr12	101062805	101063026	10chr12	104802342	104802672	12chr12	109027527	109027768	14chr12	109128161	109128420	9chr12	109197360	109197545	11chr12	109458680	109458868	10chr12	110151287	110151531	14chr12	110433968	110434281	15chr12	111837140	111837501	10chr12	113667602	113667903	13chr1	211643441	211643661	9chr12	116930748	116930982	12chr12	117295930	117296108	11chr12	118275129	118275306	12chr12	118490341	118490547	14chr12	120339919	120340163	14chr12	120669178	120669418	11chr12	121087052	121087241	12chr12	12134144	12134428	12chr12	121581744	121581964	12chr12	122096746	122097084	12chr12	123717769	123718003	7chr12	123849790	123850061	13chr1	212407990	212408228	12chr12	125473569	125473768	10chr12	125667797	125667998	13chr12	130516207	130516388	11chr12	131232753	131232935	11chr12	133263871	133264121	15chr12	133562812	133563030	10chr12	13431182	13431383	12chr1	214434909	214435134	12chr1	214776396	214776638	13chr1	21588331	21588600	12chr12	1715191	1715509	10chr1	218550675	218550912	13chr12	1922166	1922376	11chr1	221916228	221916436	13chr1	223914440	223914670	11chr1	223963809	223964068	13chr1	22458465	22458679	12chr12	2692282	2692495	10chr12	27006780	27006999	11chr1	227975623	227975907	13chr12	31004125	31004341	12chr1	231451622	231451843	11chr12	3182636	3182826	11chr12	31885941	31886172	10chr12	32049574	32049853	10chr12	33049964	33050264	11chr1	233086165	233086416	11chr1	235416600	235416782	12chr12	38532611	38532848	11chr12	40501405	40501599	10chr1	243418655	243418892	10chr12	4417072	4417379	10chr1	244964618	244964816	11chr1	244998171	244998476	11chr1	24518453	24518667	11chr1	245206051	245206261	12chr1	246852857	246853076	13chr12	49012309	49012541	11chr12	49189670	49189905	10chr12	49390563	49390805	13chr12	4966934	4967176	13chr12	49741434	49741646	10chr12	50170214	50170429	12chr12	50260970	50261146	10chr12	51984481	51984708	10chr12	52537386	52537657	13chr12	52607348	52607552	10chr12	53268090	53268323	11chr12	53273736	53273980	11chr12	53298814	53299068	12chr12	53321160	53321462	13chr12	54426424	54426655	12chr12	54595189	54595376	12chr12	56694038	56694317	12chr12	57233720	57233941	12chr12	57487117	57487326	11chr12	57576797	57577027	11chr12	6097852	6098089	10chr12	62996958	62997215	13chr12	7070549	7070846	12chr1	27115680	27115887	12chr12	7125659	7125901	10chr1	27429269	27429456	13chr1	28441826	28442001	14chr12	89740215	89740410	13chr12	93364837	93365085	9chr13	100153553	100153799	13chr13	100568845	100569031	10chr13	100741091	100741244	11chr13	110874607	110874855	13chr13	113597054	113597275	10chr13	114427840	114428096	13chr13	114832981	114833229	10chr1	31255293	31255518	10chr1	31895587	31895842	11chr1	32166739	32166980	13chr1	32211293	32211527	10chr13	27557191	27557494	11chr13	28107266	28107510	10chr13	31998304	31998518	12chr1	33520823	33521107	14chr1	33552000	33552244	11chr13	40600346	40600664	11chr13	43294605	43294912	11chr13	45914631	45914933	11chr13	61047771	61048032	10chr1	36765548	36765838	10chr1	37041758	37041927	10chr1	37899943	37900153	10chr1	37937334	37937621	11chr1	38465295	38465517	12chr13	99137142	99137388	12chr1	40071856	40072120	12chr1	40439208	40439429	12chr14	103018452	103018757	13chr14	103995363	103995640	10chr14	104544387	104544614	10chr14	104603894	104604122	12chr14	105292743	105292983	10chr14	20883969	20884243	11chr14	23476163	23476445	12chr14	23504309	23504564	12chr14	24101260	24101480	12chr14	24483260	24483463	14chr14	24766051	24766335	10chr14	24950836	24951050	9chr14	31495992	31496256	13chr14	35891600	35891854	10chr1	44435425	44435711	10chr1	45180696	45180921	13chr14	52327187	52327411	11chr14	55556272	55556528	11chr1	45956915	45957144	11chr14	61570515	61570778	14chr14	68161525	68161785	12chr14	68868610	68868833	10chr14	75079286	75079519	9chr14	75530596	75530890	12chr14	75643211	75643511	9chr14	76027380	76027554	11chr14	77413515	77413741	12chr14	91839977	91840197	11chr15	33992988	33993187	9chr15	40602319	40602501	11chr15	40630293	40630549	12chr15	44069595	44069853	12chr15	44196069	44196244	11chr15	45016573	45016744	12chr15	45076886	45077134	14chr1	54547378	54547643	10chr15	49714223	49714425	11chr15	50295986	50296167	12chr15	50437652	50437849	9chr15	51057725	51058021	15chr15	51177488	51177707	11chr15	59430404	59430694	10chr15	60689303	60689537	11chr15	66125075	66125360	10chr15	68851988	68852252	9chr1	56991244	56991498	12chr15	78715263	78715463	11chr15	79001789	79002025	11chr15	79165068	79165279	11chr15	79471430	79471663	12chr15	80748211	80748454	10chr15	81068839	81069071	15chr15	81293117	81293362	9chr15	86029248	86029465	14chr15	89157796	89157984	11chr15	89655582	89655872	15chr15	89750122	89750345	12chr15	89954782	89954990	11chr15	90544403	90544681	10chr1	59349065	59349281	11chr15	94663221	94663410	10chr15	96870527	96870741	11chr16	11735296	11735564	11chr16	1347016	1347246	13chr16	15583419	15583641	9chr16	15766690	15766937	13chr16	18957547	18957842	9chr16	19010134	19010304	11chr16	19514066	19514280	11chr16	19897699	19897851	12chr16	21200035	21200254	11chr16	22012381	22012592	10chr16	23739155	23739447	12chr16	24528430	24528602	10chr16	27121029	27121250	10chr16	28813935	28814218	11chr16	2918436	2918646	8chr16	30381959	30382295	10chr1	63087719	63087955	10chr16	3215970	3216162	11chr16	3704326	3704539	12chr16	3740499	3740738	10chr16	441931	442157	11chr16	451924	452120	12chr16	48198883	48199051	10chr16	48492342	48492566	11chr16	53241732	53242000	10chr1	65363797	65364096	12chr16	58549242	58549488	13chr1	66277723	66277982	13chr16	66512674	66512868	10chr16	67962483	67962674	11chr16	70557280	70557614	12chr16	70772348	70772562	9chr16	70805086	70805333	11chr16	72042337	72042621	11chr16	81763897	81764218	10chr16	84674907	84675115	14chr16	88767364	88767637	14chr16	89355924	89356127	11chr16	89772420	89772604	12chr16	90014981	90015230	12chr16	9141701	9141920	10chr16	9228690	9228951	12chr17	1733025	1733246	11chr17	19417285	19417483	12chr17	1990954	1991181	12chr17	20943574	20943778	10chr17	21226312	21226555	14chr17	28690770	28691102	14chr17	32498352	32498583	10chr17	33569934	33570122	12chr17	36819441	36819650	11chr17	38771571	38771804	9chr17	39677962	39678223	13chr17	41323097	41323397	13chr17	41654071	41654306	9chr17	43176545	43176778	9chr17	43370854	43371125	11chr17	45018269	45018475	9chr17	46024356	46024597	10chr17	46080001	46080305	11chr17	46662818	46663014	14chr17	47269824	47270017	13chr17	47485458	47485634	11chr17	48157121	48157283	11chr17	48703559	48703751	10chr17	56524883	56525126	13chr17	58114492	58114741	10chr17	61996641	61996862	12chr17	65471594	65471855	12chr17	70117030	70117242	11chr1	7707340	7707564	12chr17	71224962	71225156	10chr17	72292793	72293004	12chr17	72919641	72919875	13chr17	7358774	7359008	13chr17	73629085	73629291	10chr17	73749781	73749988	9chr17	73780627	73780846	10chr17	74394178	74394390	13chr17	7589297	7589513	13chr17	77682473	77682680	11chr17	78765806	78766036	12chr17	79700856	79701053	11chr17	80009671	80009854	11chr17	80821922	80822140	9chr17	884307	884563	12chr1	8042450	8042684	14chr18	19774156	19774401	12chr18	20864034	20864294	13chr18	2972189	2972445	15chr18	3248674	3248933	12chr18	3594360	3594554	10chr18	3771447	3771631	10chr18	43684167	43684404	12chr18	43830483	43830703	13chr18	45549070	45549319	12chr18	47813978	47814265	12chr18	51915178	51915407	10chr18	56325451	56325677	12chr18	56338242	56338551	11chr1	85742153	85742391	10chr1	88646257	88646506	14chr18	9073665	9073889	12chr19	10514860	10515030	12chr19	10981962	10982184	10chr19	1102522	1102766	10chr19	11248470	11248742	9chr19	11491740	11491996	11chr19	13001754	13001967	11chr19	13973098	13973303	11chr19	14480516	14480821	13chr19	14542816	14543055	12chr19	1507801	1508094	13chr19	15740155	15740369	9chr19	16229409	16229643	12chr19	16308546	16308835	11chr19	16358505	16358652	14chr19	16695190	16695464	13chr19	17457081	17457279	15chr19	17564299	17564507	12chr19	17666215	17666425	10chr19	17877423	17877688	10chr19	18215168	18215431	8chr19	18385672	18385968	10chr19	18485200	18485459	14chr19	18589772	18590012	13chr19	19144511	19144778	13chr19	19249218	19249484	10chr19	19256900	19257075	10chr19	19475702	19475973	13chr19	2019669	2019883	13chr19	2094356	2094633	9chr19	2169025	2169202	13chr19	22018838	22019189	12chr19	23941561	23941859	14chr19	30521203	30521401	7chr19	3146625	3146867	10chr19	3429605	3429811	11chr19	35896235	35896489	11chr19	35986342	35986580	12chr19	37663476	37663768	10chr19	40283711	40283982	12chr19	4145804	4146027	12chr19	42455850	42456058	11chr1	94511140	94511427	12chr19	45227242	45227386	11chr19	45922839	45923005	12chr19	46991157	46991331	10chr19	47799467	47799666	11chr19	4803977	4804182	11chr19	4831672	4831944	12chr19	48372226	48372460	12chr19	48829221	48829495	11chr19	50073459	50073697	10chr19	50143762	50144094	12chr19	51228195	51228535	13chr19	5139269	5139540	14chr19	51399949	51400162	10chr19	51661771	51661983	11chr19	52772695	52772998	11chr19	53289935	53290127	11chr19	54975845	54976123	11chr19	55517403	55517654	11chr19	55574378	55574721	13chr19	55765485	55765748	11chr19	56189554	56189832	12chr1	956519	956763	11chr19	5805835	5806096	8chr19	59025370	59025569	12chr19	6166029	6166302	11chr19	734073	734335	12chr1	9785128	9785345	12chr19	8317946	8318127	13chr19	8420540	8420786	14chr19	8578614	8578898	14chr19	8650861	8651042	10chr19	980467	980786	13chr20	11248020	11248251	12chr20	1294252	1294457	11chr20	2295706	2296006	11chr20	2432835	2433072	13chr20	247644	247839	13chr20	31253210	31253439	10chr20	3199165	3199480	12chr20	32582167	32582460	12chr20	382595	382806	10chr20	42584142	42584316	10chr20	42846043	42846293	11chr20	43013103	43013277	12chr20	43996935	43997150	11chr20	44803206	44803423	11chr20	45318013	45318320	12chr20	45903266	45903533	15chr20	46483621	46483789	10chr20	4666759	4667053	13chr20	49262124	49262395	13chr20	49609629	49609864	10chr20	49988904	49989125	12chr20	51833674	51833873	12chr20	55956915	55957137	13chr20	57199022	57199236	12chr20	62482215	62482494	10chr20	62497151	62497409	9chr2	101033872	101034147	11chr2	101510340	101510602	13chr2	101737042	101737360	12chr2	112645363	112645609	11chr2	113384163	113384371	10chr2	113956716	113956927	12chr21	16855560	16855750	13chr2	119613205	119613481	11chr2	121070837	121071066	12chr2	121310569	121310751	10chr2	121437924	121438150	11chr2	121832680	121832927	11chr2	12633221	12633475	11chr2	128821071	128821285	11chr21	33973354	33973594	11chr21	34852046	34852332	12chr2	136288345	136288646	10chr21	36399051	36399261	13chr21	37513847	37514066	12chr21	37792288	37792536	11chr21	39084515	39084711	11chr21	43648275	43648561	14chr21	43835260	43835456	11chr21	43887437	43887676	12chr21	47065817	47066002	10chr2	152882335	152882617	11chr2	154335237	154335506	11chr2	15777486	15777728	12chr2	160778394	160778573	13chr2	166810258	166810592	14chr2	172017202	172017438	8chr2	175260310	175260578	11chr2	177419224	177419426	13chr2	178628434	178628698	13chr2	196351811	196351999	13chr2	197466210	197466459	13chr2	198063681	198063876	13chr2	201336068	201336380	14chr2	201766264	201766464	12chr2	208634391	208634676	12chr2	216658463	216658712	10chr22	18942009	18942202	11chr2	219717618	219717806	10chr22	19868747	19868965	10chr22	19897550	19897741	12chr22	19941242	19941479	11chr2	220071343	220071623	12chr22	20116824	20117024	12chr2	220363382	220363660	11chr22	23520730	23520879	14chr2	224858422	224858686	13chr22	26879739	26879926	13chr2	227700533	227700757	12chr22	28114243	28114498	12chr2	228749210	228749374	12chr22	29977084	29977309	11chr22	30195265	30195524	11chr2	232063104	232063361	10chr2	232347485	232347729	13chr2	232790014	232790199	11chr22	33445178	33445369	12chr2	236688144	236688355	11chr22	38329923	38330205	12chr22	38510588	38510768	10chr2	238767037	238767323	10chr22	39410097	39410371	10chr22	39566883	39567117	11chr22	40552045	40552319	11chr22	41465035	41465262	14chr2	242255080	242255321	10chr22	42840484	42840787	13chr22	43329519	43329726	13chr22	44278949	44279244	13chr22	45949703	45949947	9chr22	46402261	46402556	11chr22	46763623	46763836	9chr22	47000342	47000571	10chr22	47330841	47331066	12chr22	50587617	50587823	12chr22	50628847	50629093	14chr22	50780789	50781008	11chr2	25643757	25643994	10chr2	26074814	26075051	12chr2	26291515	26291744	13chr2	27543951	27544179	11chr2	37193905	37194190	10chr2	37881000	37881224	10chr2	39457044	39457276	10chr2	42313641	42313898	10chr2	44223152	44223476	10chr2	44706642	44706803	10chr2	45182830	45183055	11chr2	46165972	46166218	11chr2	64836467	64836771	10chr2	65593821	65594002	15chr2	68479756	68479981	11chr2	68668900	68669153	12chr2	70351175	70351497	14chr2	7049946	7050183	13chr2	70528406	70528649	9chr2	71299078	71299373	12chr2	74734646	74734900	10chr2	74881136	74881385	10chr2	8374969	8375170	11chr2	85779278	85779465	13chr2	85829748	85830057	10chr2	8617974	8618246	10chr2	86221129	86221437	14chr2	96823624	96823829	12chr3	100309435	100309645	11chr3	101237309	101237547	13chr3	12176584	12176851	12chr3	124480215	124480406	12chr3	12748064	12748287	11chr3	12882910	12883239	10chr3	129132629	129132926	13chr3	131748153	131748381	13chr3	141160249	141160498	10chr3	142695550	142695701	9chr3	14413327	14413657	12chr3	159706367	159706651	13chr3	160117394	160117638	13chr3	172384690	172384932	11chr3	178865702	178865940	12chr3	183706073	183706328	14chr3	183967235	183967522	14chr3	186600700	186600865	11chr3	187686764	187686949	11chr3	196377826	196378110	13chr3	197121496	197121710	13chr3	197676644	197676981	10chr3	25689611	25689818	11chr3	27525949	27526164	13chr3	33138712	33138950	12chr3	37011504	37011751	14chr3	42872807	42873038	8chr3	44277493	44277786	11chr3	49158219	49158426	12chr3	50232023	50232243	12chr3	50283608	50283846	10chr3	5068597	5068788	11chr3	51426881	51427056	11chr3	5236491	5236725	11chr3	52739924	52740290	14chr3	56792715	56792908	12chr3	75484142	75484380	11chr3	75690493	75690733	13chr3	9438694	9438931	13chr3	99721742	99722038	11chr4	100325783	100325974	12chr4	100737791	100738077	9chr4	101017201	101017437	8chr4	106392780	106393053	14chr4	113091653	113091896	7chr4	124694094	124694327	8chr4	139163454	139163739	11chr4	144695198	144695436	11chr4	185089622	185089818	11chr4	186518803	186519019	9chr4	236066	236288	12chr4	2415080	2415368	10chr4	2765890	2766083	12chr4	3482354	3482508	9chr4	39611146	39611330	13chr4	42641028	42641242	12chr4	57826514	57826714	12chr4	693230	693458	13chr4	7083770	7084050	11chr4	7803859	7804099	12chr4	78078127	78078438	11chr4	8178617	8178842	13chr4	8338270	8338476	10chr4	84457185	84457470	11chr4	87816914	87817183	12chr5	115421013	115421227	12chr5	118127767	118128002	13chr5	118747748	118748032	8chr5	123919556	123919819	14chr5	131399141	131399364	11chr5	131862456	131862745	12chr5	133913552	133913721	11chr5	134901918	134902111	11chr5	135389389	135389619	12chr5	136834909	136835170	11chr5	140900830	140901051	12chr5	141044661	141044880	11chr5	143204748	143204956	11chr5	145912957	145913120	11chr5	148110582	148110770	12chr5	148414925	148415170	12chr5	148514082	148514314	13chr5	150036044	150036331	12chr5	15008671	15008862	14chr5	158122612	158122849	14chr5	159846016	159846264	9chr5	172204498	172204739	11chr5	172205203	172205446	13chr5	17526623	17526829	12chr5	34188411	34188632	9chr5	36693320	36693591	12chr5	41856880	41857081	12chr5	42950780	42951022	12chr5	431140	431319	12chr5	52777804	52777993	12chr5	55408457	55408740	9chr5	55419625	55419933	13chr5	56205787	56206017	11chr5	58332238	58332403	11chr5	59995390	59995718	12chr5	66502832	66503029	15chr5	68485205	68485514	10chr5	76034869	76035128	10chr5	81461791	81461996	10chr5	94417249	94417500	11chr5	99870866	99871190	13chr6	104973637	104973876	12chr6	106960261	106960471	11chr6	110026476	110026690	12chr6	11921348	11921535	11chr6	122720355	122720742	8chr6	138029051	138029355	13chr6	139540413	139540691	11chr6	14144911	14145107	12chr6	143206572	143206866	14chr6	143234720	143234939	10chr6	144471330	144471589	11chr6	152416365	152416637	10chr6	155495918	155496115	10chr6	157019482	157019712	7chr6	158244194	158244374	11chr6	158652685	158652951	10chr6	159239158	159239389	12chr6	170826115	170826371	10chr6	17706131	17706458	13chr6	17907964	17908230	11chr6	20212975	20213327	13chr6	21438871	21439130	11chr6	21589535	21589719	12chr6	22025960	22026289	12chr6	25279140	25279431	10chr6	2765506	2765698	12chr6	27745561	27745729	11chr6	27759912	27760202	13chr6	28411148	28411374	13chr6	2998491	2998790	12chr6	3002127	3002360	10chr6	30687843	30688082	11chr6	31367635	31367893	14chr6	32016022	32016220	10chr6	32086636	32086883	11chr6	33257116	33257430	11chr6	36063047	36063198	10chr6	36164100	36164335	11chr6	36733975	36734244	11chr6	3732829	3733072	11chr6	41039518	41039781	9chr6	41499674	41499932	13chr6	42738866	42739103	13chr6	43968141	43968423	13chr6	52020907	52021102	13chr6	52528498	52528718	13chr6	56755509	56755683	12chr6	64308573	64308840	10chr6	6687445	6687656	12chr6	69942822	69943047	11chr6	7058701	7058887	12chr6	73839699	73839932	12chr6	76458827	76459092	10chr7	100302942	100303188	10chr7	100464851	100465067	11chr7	100926282	100926489	12chr7	101457479	101457693	12chr7	101880026	101880222	12chr7	102119287	102119572	11chr7	102632196	102632438	12chr7	105650011	105650215	12chr7	107851950	107852171	15chr7	128038701	128038882	12chr7	131339484	131339753	11chr7	137663243	137663515	12chr7	138423544	138423732	13chr7	139330739	139330999	10chr7	139702177	139702380	10chr7	142705063	142705326	13chr7	142985054	142985273	11chr7	149439283	149439585	12chr7	150018006	150018352	10chr7	150660126	150660339	10chr7	151000611	151000846	9chr7	157043394	157043683	11chr7	17448227	17448423	12chr7	20279964	20280188	11chr7	22491849	22492179	13chr7	23610324	23610486	13chr7	2749353	2749598	10chr7	29229389	29229585	12chr7	30978412	30978674	10chr7	33934988	33935245	12chr7	35749160	35749365	13chr7	37394282	37394707	12chr7	44059876	44060081	13chr7	44605156	44605351	12chr7	61752004	61752181	11chr7	6487509	6487694	11chr7	72971442	72971717	12chr7	73441183	73441448	11chr7	73497747	73498025	11chr7	73981170	73981340	12chr7	74024722	74024974	11chr7	76961262	76961484	14chr7	871407	871607	12chr7	91808245	91808506	11chr7	92053940	92054143	12chr7	97501682	97501977	13chr7	98928213	98928472	11chr7	99728596	99728856	10chr7	99844995	99845212	15chr8	101170611	101170879	13chr8	105772005	105772232	11chr8	116197759	116197979	14chr8	119755953	119756135	12chr8	123584619	123584849	10chr8	12523025	12523333	12chr8	126422852	126423078	13chr8	131761894	131762200	11chr8	135703636	135703854	10chr8	144699716	144699953	15chr8	145010704	145010959	12chr8	146024102	146024323	12chr8	15397555	15397853	12chr8	17591831	17592072	13chr8	21772930	21773149	13chr8	23331850	23332132	11chr8	26434389	26434737	11chr8	27168854	27169128	13chr8	28747698	28747963	12chr8	37248066	37248341	10chr8	41009180	41009421	13chr8	41573767	41574032	15chr8	46941160	46941325	10chr8	47868673	47868948	13chr8	56843143	56843391	12chr8	57154528	57154759	10chr8	57906315	57906498	12chr8	59307836	59308073	12chr8	59720545	59720823	10chr8	62672732	62673004	11chr8	62679781	62679985	11chr8	63951300	63951515	12chr8	6658340	6658561	11chr8	7255366	7255569	12chr8	73067483	73067674	10chr8	7771067	7771236	12chr8	81143026	81143303	11chr8	91479941	91480140	11chr8	96237748	96238042	10chr8	98905516	98905685	10chr8	99838274	99838480	10chr9	100230993	100231144	12chr9	100459351	100459678	13chr9	101337954	101338250	14chr9	102790486	102790726	13chr9	103357909	103358219	10chr9	107730448	107730670	12chr9	112730020	112730209	12chr9	116844841	116845091	12chr9	124861659	124861893	11chr9	124887522	124887719	11chr9	125590696	125590982	11chr9	126112191	126112404	10chr9	126147695	126147950	9chr9	126889180	126889372	11chr9	129263265	129263467	11chr9	129748164	129748450	13chr9	130348854	130349001	10chr9	130764096	130764304	11chr9	130797530	130797752	9chr9	130922521	130922705	9chr9	131017545	131017767	9chr9	131486273	131486551	9chr9	131549438	131549629	12chr9	131965087	131965321	14chr9	132168732	132168928	11chr9	133005497	133005697	11chr9	134211684	134211869	13chr9	134406581	134406838	12chr9	135111859	135112018	11chr9	138593855	138594059	9chr9	140356289	140356545	13chr9	140446274	140446483	10chr9	26947325	26947659	11chr9	2844072	2844290	13chr9	33081306	33081553	13chr9	33403332	33403614	12chr9	34612806	34613050	13chr9	34665977	34666237	13chr9	35116849	35117075	11chr9	5590103	5590289	12chr9	6645620	6645924	13chr9	71795651	71795884	10chr9	74653930	74654169	9chr9	86535837	86536056	10chr9	88901977	88902239	10chr9	91193434	91193659	11chr9	92164050	92164210	12chr9	93927084	93927266	11chr9	96444435	96444718	10chrX	100023787	100023993	11chrX	107334738	107335036	12chrX	128737926	128738187	10chrX	128771550	128771774	12chrX	12975071	12975290	9chrX	13003204	13003421	11chrX	134124916	134125233	11chrX	134166208	134166468	10chrX	149108516	149108766	12chrX	152110153	152110368	13chrX	153029438	153029700	11chrX	153166328	153166553	11chrX	1640551	1640786	16chrX	22003497	22003684	12chrX	2243184	2243396	12chrX	24711846	24712112	13chrX	2730424	2730753	12chrX	41193339	41193538	7chrX	41243413	41243663	10chrX	43817769	43818072	10chrX	49020043	49020347	12chrX	52949988	52950202	15chrX	68048627	68048907	9chrX	69353133	69353359	9chrX	7050489	7050730	12chrX	70842456	70842699	10chr10	104438578	104438817	11chr10	111644004	111644176	11chr10	112174292	112174618	11chr10	113899954	113900175	12chr10	114149296	114149513	10chr10	120873424	120873680	11chr10	121065657	121065868	9chr10	121485287	121485618	11chr10	124899053	124899302	13chr10	125754164	125754454	10chr10	134957473	134957767	13chr10	13820040	13820253	10chr10	21567712	21567977	12chr10	21623946	21624267	11chr10	27541486	27541733	10chr10	27649590	27649770	13chr10	30707703	30707938	9chr10	30722899	30723205	12chr10	30726063	30726307	11chr10	30799570	30799811	12chr10	32236602	32236796	12chr10	35484692	35484918	11chr10	38299393	38299656	10chr10	44167133	44167430	11chr10	45353224	45353487	11chr10	48426927	48427138	10chr10	50970264	50970538	10chr10	52178106	52178360	10chr10	61996711	61996963	13chr10	62537968	62538173	12chr10	63664563	63664845	9chr10	65462869	65463103	11chr10	72639887	72640041	9chr10	73079356	73079576	13chr10	73468044	73468219	10chr10	74020736	74020951	10chr10	75949831	75950125	11chr10	77796150	77796374	10chr10	77863105	77863353	11chr10	79671410	79671664	10chr10	81903567	81903825	11chr10	82459355	82459589	13chr10	88720267	88720451	11chr10	90692484	90692676	10chr10	93557998	93558227	11chr10	97068588	97068865	12chr10	97592099	97592298	9chr10	99092752	99092984	11chr1	109631912	109632168	13chr1	11002064	11002278	10chr1	110333372	110333724	11chr1	110572239	110572464	11chr1	110739567	110739777	11chr11	107711524	107711738	11chr11	112096838	112097077	11chr1	111415623	111415823	9chr1	111682783	111683018	11chr11	116943326	116943523	12chr1	111758706	111759035	9chr11	117778039	117778273	12chr11	119189457	119189731	10chr11	119194320	119194524	12chr11	120483729	120483969	9chr11	12065835	12066077	11chr11	126180466	126180669	12chr11	126211793	126212041	13chr11	134945661	134945902	11chr11	13894840	13895064	12chr11	1674005	1674198	10chr11	192018	192239	11chr11	20016679	20016906	10chr1	12268967	12269167	12chr1	1227269	1227496	11chr11	22851445	22851676	11chr11	2920023	2920257	13chr1	1307506	1307735	10chr11	308100	308349	12chr11	35412651	35412912	13chr11	3602421	3602683	12chr1	13992228	13992468	11chr1	14057409	14057668	9chr11	406817	407088	12chr11	44590813	44591014	12chr11	44796996	44797229	12chr11	44958091	44958250	12chr1	145457303	145457447	10chr11	45894301	45894547	10chr11	46142878	46143044	13chr11	47416362	47416533	11chr11	47475876	47476035	11chr1	147624476	147624672	11chr1	150209385	150209559	11chr1	150480356	150480631	12chr1	150532455	150532658	11chr1	151467336	151467620	11chr11	5226126	5226359	13chr1	153650258	153650439	9chr1	153721946	153722192	11chr1	154642233	154642438	10chr1	154914968	154915242	12chr1	154916295	154916496	10chr1	154943158	154943433	9chr1	155035234	155035439	12chr11	5621613	5621789	9chr1	156415687	156415852	9chr11	57092444	57092695	11chr11	57224904	57225060	11chr11	58292141	58292309	11chr11	58668302	58668551	11chr11	58912066	58912315	13chr11	59304732	59304939	8chr11	60674067	60674321	12chr11	60955081	60955285	11chr1	161085103	161085383	10chr11	61665204	61665362	10chr11	61793000	61793237	9chr11	62078673	62078894	13chr11	62361104	62361285	12chr1	16245899	16246168	12chr11	63439038	63439343	13chr11	63683899	63684153	9chr11	63949068	63949344	11chr11	64491007	64491170	10chr11	64546291	64546511	12chr11	64880039	64880223	11chr11	64918210	64918425	11chr11	65261414	65261577	9chr11	65308385	65308623	12chr11	65319565	65319797	11chr1	16570951	16571140	11chr11	66153667	66153918	11chr11	66445851	66446211	9chr1	167411478	167411662	11chr11	67980190	67980412	9chr11	69064882	69065188	12chr1	16970931	16971224	15chr11	70037712	70037913	9chr1	17004705	17004912	11chr11	71524800	71525070	13chr11	71855114	71855323	11chr11	72394850	72395042	12chr1	172715545	172715707	10chr11	73336845	73337032	12chr11	74437665	74437870	11chr11	75248150	75248397	10chr11	7542271	7542544	13chr11	76155711	76155959	13chr11	76432902	76433129	12chr11	76518551	76518728	9chr1	17674940	17675116	9chr11	77122909	77123087	12chr11	78356361	78356524	12chr1	180123451	180123775	12chr1	181007620	181007864	8chr1	181098318	181098567	13chr1	182054070	182054322	9chr1	182365910	182366088	10chr1	182570563	182570772	12chr11	8285440	8285616	12chr1	184774708	184775007	14chr11	85913903	85914165	12chr11	94356377	94356637	10chr11	94880710	94880908	11chr11	94987563	94987813	11chr11	9596921	9597128	10chr1	199241872	199242057	14chr1	200385147	200385319	11chr1	200835778	200835967	11chr1	201012108	201012308	10chr1	201683765	201684021	9chr1	203008530	203008744	11chr1	203307645	203307878	11chr1	203456965	203457139	13chr1	206976590	206976848	13chr1	20848886	20849089	13chr1	209750159	209750343	10chr1	209775445	209775696	10chr1	209981472	209981698	13chr12	104503049	104503234	10chr12	105352334	105352553	11chr1	210547748	210547955	9chr12	109011970	109012166	11chr12	109702014	109702257	11chr12	109915176	109915401	12chr12	111374974	111375119	10chr12	112213406	112213626	12chr12	112554403	112554696	12chr12	113430635	113430871	13chr12	117463396	117463623	10chr12	119499077	119499302	9chr12	120672533	120672831	11chr12	120794415	120794689	10chr12	120875719	120875978	10chr1	212113288	212113492	12chr12	121151730	121151964	13chr12	121881137	121881287	11chr12	121937787	121937987	11chr12	122249958	122250151	10chr12	122356319	122356488	12chr12	122906618	122906921	10chr12	123436938	123437143	12chr12	123611139	123611350	12chr12	123850245	123850503	10chr12	124066757	124066968	12chr12	125093640	125093879	12chr12	125389350	125389585	9chr1	212659337	212659557	12chr12	14766761	14766931	11chr12	14783166	14783294	10chr1	21695080	21695219	11chr1	217804312	217804620	12chr1	219262337	219262582	8chr12	20365653	20365895	10chr1	220906434	220906645	9chr1	22345626	22345839	10chr1	223900040	223900294	12chr12	26451986	26452278	12chr1	227545121	227545392	14chr1	227950193	227950376	10chr1	228651409	228651654	14chr12	29470820	29470988	10chr12	30960392	30960665	13chr12	31000221	31000434	12chr1	231227772	231228025	12chr12	31886713	31886972	10chr1	234445760	234445946	10chr1	235110961	235111233	12chr1	235159245	235159480	12chr1	235257197	235257396	11chr1	236666508	236666716	13chr1	238027702	238027893	10chr12	4140497	4140758	12chr12	4216618	4216865	12chr1	24243384	24243554	11chr12	46518966	46519205	10chr12	47058825	47059033	9chr12	4713933	4714181	12chr12	49389257	49389495	12chr12	53399884	53400121	11chr12	53835312	53835546	12chr12	54613956	54614133	13chr12	54867294	54867521	12chr12	56013523	56013735	10chr12	56075310	56075543	12chr12	56130986	56131247	11chr12	57674060	57674306	12chr12	57977193	57977387	12chr12	58119909	58120124	12chr12	58225288	58225494	8chr12	58258826	58259174	10chr12	59991203	59991545	9chr12	6172343	6172588	8chr12	6387926	6388123	11chr12	6432531	6432764	11chr12	64615923	64616132	10chr1	26471615	26471889	12chr12	6602348	6602655	11chr12	66501250	66501506	10chr12	6665061	6665338	12chr1	26826689	26826994	11chr12	68616945	68617181	11chr12	6862789	6863025	9chr12	7036774	7036983	9chr1	27438495	27438789	10chr1	27524506	27524712	9chr12	754710	754986	12chr1	27816806	27817019	10chr1	27986570	27986821	9chr1	27989678	27989913	11chr12	8179112	8179430	9chr1	28241195	28241461	11chr1	28471051	28471275	12chr12	861442	861629	12chr1	28718568	28718809	11chr12	9149371	9149597	15chr12	92172822	92172985	10chr12	94018768	94019110	14chr13	100077914	100078213	10chr13	100308451	100308616	12chr13	111522531	111522824	9chr13	114147699	114147938	12chr13	20701851	20702204	12chr1	32713747	32714003	12chr13	27757167	27757403	11chr13	27948849	27949077	9chr13	29282219	29282421	12chr13	31038277	31038482	10chr13	31618617	31618810	10chr1	33190833	33191006	13chr13	48611653	48611997	10chr13	50697971	50698225	10chr1	35657557	35657802	10chr1	36234943	36235273	11chr1	37169410	37169602	12chr13	76111850	76112046	11chr1	38000116	38000317	10chr13	95844081	95844313	11chr1	39724343	39724582	12chr13	99305751	99305923	11chr1	40264898	40265157	11chr14	100842663	100842899	13chr14	102438775	102439008	10chr14	102904442	102904635	11chr14	103759164	103759429	13chr14	103987507	103987736	12chr14	105536451	105536709	12chr14	106328280	106328472	8chr14	106829310	106829586	11chr14	20801347	20801617	8chr14	21093434	21093630	11chr14	24020889	24021098	11chr14	25602063	25602308	9chr14	30739946	30740146	10chr14	34318456	34318667	11chr14	38343890	38344131	9chr1	44047931	44048122	11chr14	50065417	50065652	11chr14	50319628	50319879	11chr14	50511758	50512008	10chr14	52118660	52118877	11chr14	55240767	55240975	10chr14	59742450	59742710	12chr14	61736365	61736594	10chr14	62029591	62029790	11chr14	62304357	62304605	10chr14	63158993	63159206	11chr14	65103472	65103648	10chr1	46769078	46769312	12chr14	68303844	68303993	10chr14	69162097	69162363	13chr14	69926869	69927100	11chr14	70046864	70047124	9chr14	70082300	70082605	14chr14	71791530	71791838	12chr14	77342422	77342614	12chr14	77534598	77534828	11chr14	78266268	78266665	13chr14	79032951	79033187	12chr14	81421185	81421416	11chr14	81930964	81931184	12chr1	48463392	48463636	10chr14	88480160	88480350	11chr14	93582223	93582465	9chr14	94406075	94406292	10chr14	97263486	97263716	13chr15	23086694	23087004	9chr15	29396021	29396253	14chr15	31508549	31508739	10chr1	53151937	53152142	12chr15	35370825	35370975	10chr15	37175983	37176169	10chr15	38361322	38361547	13chr15	41860699	41860921	9chr15	42426200	42426368	12chr1	54587273	54587478	8chr15	52121709	52122027	10chr1	55462095	55462303	11chr1	55743223	55743454	13chr15	64995535	64995713	9chr15	67417921	67418197	10chr1	57154212	57154437	11chr15	72448380	72448604	10chr15	72565125	72565324	9chr15	74583840	74584081	9chr15	74613085	74613280	10chr15	74731156	74731320	12chr15	74834218	74834467	14chr15	75335982	75336204	9chr15	76094433	76094728	12chr15	78400689	78400927	12chr15	81567455	81567636	12chr15	81589136	81589315	10chr15	85142314	85142532	10chr15	85855716	85855923	8chr15	90792532	90792792	10chr15	91593101	91593348	12chr1	59280907	59281196	12chr15	93363518	93363779	12chr15	95790358	95790603	12chr15	99062347	99062626	12chr16	10479688	10479888	11chr16	10965002	10965218	13chr16	11349530	11349858	9chr16	11457131	11457399	12chr16	12895651	12895855	11chr16	1381784	1382048	9chr16	13960041	13960279	9chr16	14013601	14013853	9chr16	1413507	1413721	11chr16	14404476	14404712	12chr16	14727877	14728082	8chr16	15859834	15860034	10chr16	1862752	1862983	11chr16	21531615	21531845	12chr16	2205532	2205865	12chr16	25067008	25067212	10chr16	28961887	28962208	11chr16	29655739	29655940	12chr16	30345923	30346287	12chr16	30361938	30362139	12chr16	3070138	3070510	14chr16	3174508	3174758	11chr16	3355319	3355626	12chr1	63833123	63833437	11chr16	3989180	3989364	11chr16	4321801	4322030	11chr16	49441897	49442119	9chr16	53468230	53468473	13chr16	5666144	5666386	10chr16	56985272	56985530	12chr16	57701406	57701621	14chr16	58035233	58035452	12chr16	58718583	58718764	10chr16	67302157	67302423	13chr16	67700557	67700775	11chr1	66801713	66801981	13chr16	70780668	70780912	9chr16	725173	725472	11chr16	77224859	77225170	11chr16	78036884	78037118	13chr16	83960316	83960550	10chr16	84651656	84651928	11chr16	8622972	8623162	10chr16	87179091	87179338	11chr16	87236845	87237045	10chr16	87417587	87417765	9chr16	87883691	87883905	10chr1	68808535	68808718	10chr16	88540268	88540544	10chr16	89098476	89098696	7chr1	70970131	70970335	13chr17	1090499	1090734	11chr17	1171464	1171670	8chr17	1420037	1420261	12chr17	1493349	1493581	12chr17	16424634	16424849	9chr17	19551921	19552250	9chr17	21416282	21416497	12chr17	259825	260102	9chr17	2689627	2689825	10chr17	27451160	27451347	11chr17	2752122	2752324	10chr17	28705787	28705999	9chr17	29046394	29046585	11chr17	30850285	30850494	12chr17	33914241	33914473	10chr17	34074506	34074748	12chr17	34985814	34986006	12chr17	3641065	3641246	12chr17	36831127	36831321	9chr17	38183311	38183520	11chr17	38597292	38597561	10chr17	39065650	39065888	10chr17	3907956	3908167	11chr17	39560295	39560513	9chr17	39804961	39805174	13chr17	39957855	39958064	11chr17	41324093	41324440	11chr17	42219197	42219408	11chr17	42408776	42408967	11chr17	42425586	42425795	8chr17	43303558	43303754	12chr17	43384764	43384953	11chr17	43398326	43398590	12chr17	43503132	43503355	10chr17	45000284	45000501	12chr17	47287826	47288095	13chr17	47653055	47653262	11chr17	48164861	48165100	10chr17	48556900	48557080	10chr17	53373331	53373594	11chr17	54669930	54670161	10chr17	56296648	56296834	11chr17	56310193	56310448	13chr17	57518204	57518425	13chr17	58042200	58042501	12chr17	597589	597779	12chr17	62645271	62645497	13chr1	76457255	76457532	11chr17	65370769	65371062	13chr17	66459137	66459386	12chr17	70556444	70556607	12chr17	7094899	7095064	8chr17	71366056	71366275	12chr17	72861021	72861261	10chr17	72954065	72954310	12chr17	74010361	74010589	11chr17	74620043	74620231	11chr17	74677418	74677584	7chr17	76628028	76628252	14chr17	76967414	76967634	10chr17	77083775	77083950	10chr17	77720022	77720290	11chr17	77751435	77751623	11chr17	77965505	77965692	9chr17	78235275	78235447	9chr17	79678854	79679110	11chr17	7983246	7983550	10chr17	79977904	79978152	9chr17	80258726	80258941	10chr17	8054537	8054777	10chr17	80739737	80739914	10chr17	8089515	8089722	13chr17	8093441	8093726	12chr17	81153371	81153614	11chr1	805207	805396	12chr18	14178811	14179163	10chr18	19477803	19477990	10chr18	20284346	20284594	11chr18	20711750	20711949	10chr18	21078827	21079064	10chr18	21095971	21096184	11chr18	268016	268219	11chr18	32966585	32966806	11chr18	3454716	3454936	8chr18	3652871	3653143	10chr1	8374419	8374614	10chr18	43302953	43303161	12chr18	43554142	43554332	11chr1	84569108	84569329	11chr18	45973078	45973258	11chr18	47794837	47795084	13chr18	48405326	48405503	10chr18	55789177	55789397	13chr18	57069752	57069958	9chr18	57553024	57553236	11chr18	61637641	61637958	13chr18	72921577	72921877	7chr18	77439322	77439523	12chr1	8940140	8940429	10chr1	90452827	90453028	11chr19	1042293	1042456	11chr19	10652213	10652380	11chr19	1077065	1077330	10chr19	1132143	1132411	12chr19	11354004	11354289	10chr19	11679109	11679312	11chr19	11877842	11878113	9chr19	12034472	12034760	11chr19	12170398	12170560	10chr19	12724650	12724899	11chr19	12949077	12949307	7chr19	1415469	1415743	12chr19	15408356	15408681	11chr19	16189798	16189979	14chr19	16482366	16482658	14chr19	17220868	17221118	10chr19	17371756	17371958	11chr19	17496848	17497046	10chr19	18132338	18132540	10chr19	18223852	18224069	11chr19	1864135	1864380	12chr19	18977385	18977576	10chr1	92198086	92198363	11chr1	9241409	9241704	10chr19	2524963	2525182	10chr19	2785308	2785587	11chr19	30302197	30302472	10chr19	33675145	33675373	11chr19	35224899	35225177	13chr19	36001299	36001512	9chr19	36208426	36208686	12chr19	36661682	36661876	9chr19	37407193	37407495	14chr19	37960107	37960391	12chr19	39042111	39042391	14chr19	39048013	39048194	12chr19	39452816	39453012	10chr19	40337048	40337300	10chr19	41172396	41172579	12chr19	41221577	41221804	10chr19	41955335	41955555	12chr19	42104035	42104207	11chr19	42924259	42924427	11chr19	43033486	43033667	11chr19	4310427	4310716	10chr19	44100314	44100610	13chr19	4523368	4523555	10chr19	46061668	46061921	10chr19	46386052	46386329	11chr19	46431009	46431245	11chr19	46766193	46766474	10chr19	47232104	47232328	10chr19	47987122	47987283	11chr19	4815674	4815921	9chr19	48201448	48201653	9chr19	49291455	49291667	13chr19	49298451	49298772	11chr19	49622147	49622340	10chr19	49804583	49804777	10chr19	5031642	5031853	11chr19	51059893	51060092	11chr19	51425598	51425788	11chr19	53030819	53031077	12chr19	53073537	53073863	11chr1	95320024	95320217	10chr19	5340769	5340992	8chr19	54704627	54704932	10chr19	55652611	55652871	9chr19	56111359	56111590	11chr19	56826050	56826316	11chr19	58238865	58239105	12chr19	5987395	5987684	11chr19	6460136	6460363	11chr19	8305310	8305528	11chr19	8461581	8461875	13chr19	9420325	9420630	11chr20	10015597	10015857	10chr20	10492217	10492457	12chr20	17976794	17976986	11chr20	18488103	18488318	10chr20	20432966	20433310	12chr20	21055665	21055882	12chr20	23422522	23422751	9chr20	24975560	24975751	13chr20	30147953	30148204	9chr20	30181103	30181380	9chr20	30283995	30284199	13chr20	30945763	30946001	13chr20	32241044	32241309	10chr20	33543554	33543811	11chr20	34787690	34787959	10chr20	3657338	3657569	10chr20	37037327	37037482	8chr20	37491872	37492040	13chr20	39591714	39591888	9chr20	39785789	39785983	13chr20	42843780	42843960	10chr20	43940284	43940464	9chr20	44718552	44718798	10chr20	45901662	45901924	10chr20	45960166	45960413	10chr20	46306649	46306854	10chr20	47355468	47355747	10chr20	48100788	48101007	9chr20	48895567	48895814	12chr20	49080793	49081116	11chr20	50016993	50017241	11chr20	50240813	50241081	11chr20	52195393	52195640	11chr20	52684181	52684454	12chr20	60732015	60732248	12chr20	62610141	62610364	10chr20	62701070	62701394	10chr20	799664	799912	13chr2	10054838	10055072	12chr2	101204601	101204826	12chr2	101923313	101923541	13chr2	102682281	102682534	11chr2	10410717	10411052	11chr2	10548520	10548774	8chr2	10588791	10588992	12chr2	106490284	106490495	12chr2	109210723	109210923	13chr2	113357702	113357885	12chr2	113699421	113699606	12chr2	114361559	114361823	12chr2	114652014	114652232	10chr21	19051460	19051655	12chr2	119640287	119640486	9chr2	120233916	120234132	8chr2	128407322	128407559	11chr2	13147035	13147288	11chr2	135761934	135762108	11chr21	37485131	37485406	12chr21	39047549	39047787	11chr21	43430239	43430513	12chr21	44936809	44937044	11chr21	45011606	45011804	12chr2	145275185	145275363	10chr21	45341954	45342230	14chr21	45758139	45758458	10chr21	46213276	46213539	13chr21	46548617	46548796	11chr21	47645169	47645371	11chr2	150454089	150454415	13chr2	158325945	158326182	9chr2	176032677	176032936	9chr2	176944633	176944852	10chr2	17827402	17827663	12chr2	179344987	179345279	10chr2	186603660	186603890	12chr2	190306040	190306233	10chr2	198175402	198175679	9chr2	201348481	201348732	10chr2	201828398	201828610	9chr2	210867078	210867295	9chr22	18111506	18111757	8chr22	18121211	18121442	12chr2	218578240	218578454	10chr2	219757421	219757654	13chr2	220142506	220142719	10chr2	220253136	220253357	15chr22	21983653	21983821	11chr22	22711680	22711879	12chr22	23470636	23470847	10chr22	27006314	27006576	13chr22	28122670	28122959	13chr2	228276077	228276297	12chr2	228331029	228331300	11chr22	29195323	29195606	10chr22	29663774	29664035	9chr22	30658931	30659146	12chr22	30822544	30822753	8chr22	31005383	31005613	11chr2	231084715	231084928	9chr2	232316691	232316976	13chr2	233949198	233949479	13chr2	234259441	234259685	13chr2	234620107	234620386	10chr2	235588710	235589010	11chr22	36697447	36697663	10chr22	36866360	36866566	11chr22	37963352	37963570	11chr22	38077643	38077832	11chr2	238653729	238653907	10chr22	39704833	39705070	11chr22	39712429	39712663	11chr2	239772921	239773177	10chr2	241560945	241561211	15chr22	41999934	42000221	11chr22	42765436	42765642	10chr22	42915670	42915912	11chr22	43669983	43670186	12chr22	43784406	43784664	9chr22	44208133	44208478	10chr22	45973963	45974133	11chr22	46924127	46924348	10chr2	25015695	25015942	13chr22	50431892	50432071	8chr2	26205527	26205756	12chr2	27184639	27184898	11chr2	27321543	27321751	8chr2	3307401	3307625	10chr2	37617608	37617865	11chr2	43019697	43019871	10chr2	43158639	43158889	10chr2	43354370	43354556	11chr2	43449094	43449315	10chr2	44518856	44519081	9chr2	48333744	48334027	10chr2	64558022	64558242	12chr2	65092694	65092951	11chr2	68349583	68349771	13chr2	69170703	69170891	10chr2	70418058	70418291	13chr2	73612719	73612894	11chr2	85493674	85493848	8chr2	85766234	85766428	11chr2	8685107	8685396	13chr2	8738458	8738689	10chr2	91799369	91799642	9chr2	9279938	9280171	12chr2	96804437	96804691	11chr2	9771090	9771387	12chr2	97778093	97778355	9chr2	99029382	99029631	11chr3	101655521	101655838	12chr3	106021424	106021618	13chr3	108800813	108801116	12chr3	112709884	112710136	12chr3	11684231	11684489	10chr3	121977713	121977921	9chr3	122201950	122202188	11chr3	122328949	122329269	12chr3	122785713	122785951	12chr3	127391471	127391684	10chr3	127771036	127771235	12chr3	128303363	128303549	10chr3	128327257	128327502	13chr3	128594437	128594682	10chr3	128930727	128931003	10chr3	128945292	128945466	12chr3	130611811	130612041	10chr3	13133743	13133916	11chr3	133540747	133540972	7chr3	133931832	133932074	10chr3	13526649	13526857	12chr3	139062704	139062946	12chr3	141998904	141999152	11chr3	142442851	142443085	12chr3	156393287	156393572	11chr3	170529856	170530085	11chr3	171524353	171524608	12chr3	172208000	172208208	10chr3	172428770	172428979	12chr3	183353262	183353472	10chr3	183892370	183892566	13chr3	183894279	183894535	12chr3	184135683	184135879	12chr3	184231554	184231785	12chr3	184279106	184279353	9chr3	185191338	185191590	13chr3	187457743	187457960	12chr3	187458725	187458937	11chr3	193590184	193590420	11chr3	193858605	193858873	10chr3	194871229	194871552	11chr3	195163580	195163779	10chr3	196336230	196336461	12chr3	197325996	197326229	9chr3	20081422	20081651	11chr3	37034843	37035061	13chr3	39192459	39192689	12chr3	40626099	40626328	10chr3	42485379	42485621	11chr3	46746580	46746816	10chr3	46951092	46951284	11chr3	48481505	48481762	10chr3	48701492	48701718	10chr3	4887583	4887800	13chr3	49943731	49943982	11chr3	50226808	50227030	10chr3	50275444	50275681	13chr3	5053422	5053725	8chr3	51703321	51703552	10chr3	52265589	52265833	11chr3	5468099	5468301	10chr3	61237042	61237346	11chr3	65025532	65025721	13chr3	72193970	72194189	13chr3	72806162	72806402	11chr3	73150312	73150491	11chr3	88052349	88052589	11chr3	98275165	98275381	12chr4	103352603	103352790	10chr4	108939806	108940019	11chr4	111437577	111437813	11chr4	124426846	124427129	12chr4	129349024	129349278	13chr4	147096738	147097008	14chr4	153700987	153701208	8chr4	155547963	155548289	11chr4	166397898	166398052	13chr4	171011260	171011519	11chr4	185339128	185339374	11chr4	186064200	186064452	13chr4	190200648	190200847	11chr4	25989826	25990046	12chr4	3534054	3534373	8chr4	37604742	37604973	10chr4	37688121	37688342	11chr4	37979425	37979626	9chr4	38525539	38525815	12chr4	38858389	38858568	11chr4	40910757	40910978	12chr4	41283128	41283404	11chr4	48049421	48049596	11chr4	48908699	48908970	12chr4	48911765	48911995	12chr4	55100463	55100657	12chr4	56411932	56412212	13chr4	77101093	77101300	10chr4	8596218	8596430	12chr4	88157051	88157244	10chr4	90032325	90032591	9chr5	103144216	103144474	11chr5	108992616	108992854	13chr5	123964847	123965084	12chr5	125930863	125931199	12chr5	126220055	126220302	12chr5	129103656	129103845	12chr5	130718164	130718422	12chr5	131439355	131439634	11chr5	131836925	131837231	12chr5	132142996	132143289	11chr5	132946722	132946927	10chr5	133476244	133476480	11chr5	137368495	137368841	11chr5	141229286	141229579	11chr5	14143183	14143490	13chr5	149340012	149340229	8chr5	150632462	150632705	12chr5	153451960	153452184	10chr5	158690110	158690389	10chr5	168006613	168006863	13chr5	168106536	168106730	10chr5	169694944	169695183	12chr5	173344988	173345199	11chr5	174905398	174905646	10chr5	174961166	174961353	12chr5	179498916	179499152	10chr5	317293	317503	10chr5	32329539	32329736	10chr5	34499219	34499510	11chr5	42993384	42993566	13chr5	4879915	4880113	10chr5	53606471	53606652	12chr5	54442466	54442710	11chr5	65222207	65222494	11chr5	69710997	69711235	11chr5	71078835	71079051	9chr5	71530108	71530310	11chr5	76941398	76941584	11chr5	80802841	80803066	11chr5	92917143	92917337	11chr5	95309722	95309997	14chr6	105307664	105307973	9chr6	10889715	10889958	14chr6	110360698	110360897	12chr6	112364265	112364522	14chr6	131206617	131206791	11chr6	131384287	131384505	10chr6	13328784	13329009	11chr6	13575452	13575676	11chr6	135819407	135819779	9chr6	136547393	136547614	11chr6	137143502	137143777	11chr6	1397286	1397472	10chr6	14109231	14109569	11chr6	141873784	141873941	10chr6	143265640	143265897	11chr6	146864581	146864820	10chr6	14910960	14911153	12chr6	150401854	150402121	10chr6	157542394	157542731	12chr6	157800984	157801256	12chr6	158177810	158178015	10chr6	158896976	158897147	6chr6	159460339	159460591	11chr6	160183355	160183577	12chr6	160391002	160391239	10chr6	16320251	16320568	13chr6	17702186	17702390	11chr6	20292020	20292229	13chr6	217145	217315	10chr6	27870186	27870398	13chr6	28456728	28456964	11chr6	28554982	28555212	10chr6	28984966	28985180	10chr6	2952849	2953113	10chr6	30181852	30182124	11chr6	30364741	30364895	8chr6	30524870	30525186	9chr6	30650348	30650639	13chr6	30853865	30854125	11chr6	32212793	32212969	10chr6	32557442	32557708	11chr6	32920929	32921155	12chr6	32935362	32935577	12chr6	33216960	33217231	12chr6	33553462	33553715	10chr6	35704113	35704334	12chr6	36819002	36819193	11chr6	36973325	36973559	10chr6	37282693	37282911	11chr6	37673258	37673458	12chr6	42016322	42016638	14chr6	42283832	42284040	8chr6	42629329	42629589	10chr6	42714718	42714999	15chr6	43691649	43691857	9chr6	43969105	43969393	10chr6	44235826	44236037	10chr6	4942443	4942688	12chr6	52747127	52747406	13chr6	6759475	6759681	10chr6	69678055	69678280	12chr6	7963561	7963795	10chr6	90097272	90097479	13chr7	100143306	100143554	9chr7	100202338	100202533	11chr7	101929493	101929745	10chr7	102080676	102080941	13chr7	102389216	102389462	11chr7	105292860	105293086	11chr7	12755273	12755549	14chr7	128099438	128099669	11chr7	129469977	129470203	12chr7	130796048	130796265	12chr7	134896094	134896295	9chr7	137588464	137588656	10chr7	137846350	137846548	10chr7	138571340	138571556	13chr7	139475682	139475882	10chr7	139616319	139616569	10chr7	144088908	144089140	7chr7	1500520	1500704	13chr7	20259422	20259659	14chr7	29961122	29961314	10chr7	30603972	30604150	11chr7	30839336	30839601	11chr7	33102705	33102920	10chr7	37092359	37092565	12chr7	37960065	37960305	11chr7	42909091	42909294	13chr7	43300949	43301152	11chr7	44836999	44837242	7chr7	4671363	4671561	11chr7	51230538	51230793	10chr7	55601099	55601343	12chr7	5596015	5596296	12chr7	5601673	5601893	10chr7	644120	644356	11chr7	66022629	66022817	11chr7	72385627	72385829	11chr7	73027179	73027415	12chr7	73693993	73694207	11chr7	7464109	7464368	10chr7	75272252	75272474	9chr7	75472262	75472549	12chr7	75495843	75496065	11chr7	75947380	75947690	12chr7	77168016	77168285	11chr7	8301705	8301972	10chr7	86848995	86849168	13chr7	962958	963174	12chr7	97924222	97924436	11chr7	98439435	98439631	12chr7	99730346	99730558	12chr8	10131439	10131696	10chr8	101455360	101455641	11chr8	11447013	11447288	10chr8	120667456	120667666	12chr8	123745786	123746065	10chr8	12458923	12459108	12chr8	128843942	128844132	11chr8	129252662	129252936	13chr8	129568961	129569202	12chr8	130738616	130738896	12chr8	132828709	132828906	11chr8	134387803	134388051	11chr8	134465621	134465868	11chr8	141403937	141404169	11chr8	142357061	142357250	10chr8	142414065	142414329	11chr8	143757556	143757816	12chr8	144329022	144329245	10chr8	144918631	144918852	9chr8	145703256	145703551	12chr8	145754274	145754491	9chr8	182400	182615	12chr8	22550960	22551213	10chr8	27283763	27283964	11chr8	27930326	27930583	11chr8	29522746	29522930	13chr8	30513541	30513755	12chr8	30585258	30585595	12chr8	30776507	30776715	11chr8	37002329	37002562	12chr8	37637471	37637654	12chr8	38268213	38268406	10chr8	41649980	41650209	10chr8	41852859	41853052	10chr8	42010923	42011147	11chr8	49701400	49701632	9chr8	59572307	59572578	11chr8	61911764	61911936	12chr8	62008705	62008903	12chr8	62051613	62051838	14chr8	6637145	6637383	12chr8	66860795	66861021	8chr8	67351243	67351427	11chr8	72836334	72836569	9chr8	72912646	72912883	12chr8	8204024	8204309	13chr8	86209720	86209962	10chr8	8887402	8887571	13chr8	90738117	90738323	11chr8	97345445	97345661	11chr9	100797047	100797320	10chr9	101026106	101026283	10chr9	101558729	101558997	10chr9	101761195	101761394	9chr9	111774562	111774835	13chr9	114246592	114246884	10chr9	114688893	114689119	12chr9	115607035	115607249	11chr9	117314289	117314575	10chr9	124885280	124885556	11chr9	12784542	12784751	11chr9	129467581	129467777	11chr9	129929149	129929437	12chr9	130461573	130461827	11chr9	131901506	131901758	9chr9	132386515	132386693	13chr9	132672916	132673124	15chr9	132769762	132769957	13chr9	133814516	133814713	11chr9	134128015	134128273	12chr9	134505789	134506052	12chr9	135853141	135853329	10chr9	136055382	136055582	10chr9	138018348	138018617	12chr9	139118012	139118214	12chr9	139334193	139334438	11chr9	139379527	139379712	12chr9	139383151	139383361	13chr9	139566150	139566335	10chr9	140330515	140330721	12chr9	140500168	140500512	12chr9	14322573	14322830	12chr9	19926056	19926263	11chr9	2017468	2017705	12chr9	32379354	32379519	8chr9	32384337	32384602	12chr9	33165795	33166029	11chr9	33415512	33415712	12chr9	33817808	33818050	9chr9	34591743	34591960	10chr9	35790327	35790610	10chr9	38047790	38048054	9chr9	4315696	4315871	11chr9	44070644	44070800	12chr9	44118618	44118917	13chr9	69999871	70000041	13chr9	77388849	77389097	10chr9	88555736	88555921	13chr9	94909582	94909821	10chr9	96215811	96216044	12chr9	97889988	97890150	11chr9	998674	998847	11chrX	117629425	117629702	11chrX	11777531	11777762	10chrX	118646500	118646720	10chrX	119384469	119384696	12chrX	119602907	119603203	11chrX	129535749	129536050	9chrX	130975491	130975705	11chrX	134478503	134478736	11chrX	150250835	150251133	9chrX	1587236	1587508	7chrX	24099977	24100273	12chrX	40506771	40507031	13chrX	40653372	40653555	10chrX	41112970	41113237	11chrX	46306519	46306806	9chrX	47050030	47050296	10chrX	47518395	47518694	10chrX	52950370	52950630	13chrX	56790897	56791150	11chrX	64801898	64802078	11chrX	69509689	69509955	10chrX	73834403	73834578	8chrX	77165875	77166116	13chrX	9320711	9320986	10chrX	9797663	9797890	12chr10	101419083	101419361	12chr10	102921536	102921778	11chr10	104614248	104614439	11chr10	105352551	105352756	9chr10	105456266	105456533	9chr10	112631347	112631588	11chr10	114833561	114833813	12chr10	118547612	118547843	11chr10	119412744	119412940	13chr10	119423327	119423566	11chr10	120514497	120514761	12chr10	12095593	12095813	11chr10	12306265	12306498	10chr10	123822801	123823040	13chr10	126092081	126092362	10chr10	126406768	126406962	11chr10	17272487	17272755	12chr10	18899521	18899722	12chr10	21367708	21367939	11chr10	23277829	23278075	10chr10	23357147	23357388	9chr10	24772699	24772918	8chr10	26776524	26776754	10chr10	2970504	2970759	11chr10	3085791	3085992	11chr10	31025815	31026057	10chr10	31892378	31892614	11chr10	33619523	33619687	11chr10	35507478	35507707	11chr10	38146603	38146764	9chr10	43362181	43362425	12chr10	44185553	44185827	11chr10	44796551	44796763	13chr10	49732353	49732578	10chr10	50385489	50385730	9chr10	52181128	52181313	12chr10	6019508	6019727	10chr10	63525420	63525659	13chr10	6390036	6390285	11chr10	64003089	64003282	11chr10	64564101	64564306	12chr10	73526507	73526756	12chr10	73631139	73631326	8chr10	74034546	74034766	12chr10	74055817	74056078	11chr10	76737753	76737985	12chr10	7829920	7830129	12chr10	79606520	79606687	11chr10	81080999	81081187	7chr10	81107805	81108067	11chr10	81321977	81322156	11chr10	86300368	86300541	9chr10	94516107	94516316	12chr10	95136220	95136410	11chr10	96039888	96040123	9chr10	96892786	96893021	11chr10	98346234	98346404	12chr10	98346756	98347000	12chr10	98832661	98832882	10chr10	99205676	99205888	9chr1	101754100	101754307	9chr1	109935604	109935840	10chr11	100551901	100552133	12chr11	100557950	100558194	9chr1	110438475	110438709	11chr11	107729503	107729710	9chr11	109734893	109735109	9chr11	110247542	110247720	11chr11	112090562	112090743	9chr11	114309945	114310293	9chr11	115195399	115195654	11chr1	111765574	111765776	11chr11	11820438	11820660	11chr11	118359275	118359486	11chr11	119210689	119210922	9chr11	11988462	11988647	10chr11	120105838	120105998	10chr11	120418647	120418866	11chr11	120894663	120894877	9chr11	123447744	123447932	10chr11	124065940	124066094	10chr11	124616685	124616938	8chr11	124633072	124633288	11chr11	128188827	128189016	13chr11	128342101	128342296	10chr11	1283774	1283978	10chr11	1330859	1331097	10chr1	11333219	11333448	10chr11	13484664	13484920	12chr11	14913073	14913352	13chr11	1596008	1596208	12chr11	1793799	1794054	14chr1	119829322	119829550	11chr11	20154018	20154202	12chr11	236865	237075	11chr1	1243397	1243653	14chr11	2441996	2442185	9chr1	12679037	12679305	9chr11	27180393	27180555	9chr11	27255722	27255962	11chr11	27720575	27720824	9chr1	1284672	1284876	10chr11	3131451	3131674	9chr11	3430058	3430285	10chr11	34534452	34534705	9chr11	34706868	34707118	11chr11	35061815	35062003	9chr11	35413794	35414014	13chr1	1373433	1373694	12chr11	3862857	3863117	10chr1	142890914	142891182	13chr11	44578291	44578562	11chr1	145030053	145030240	11chr1	145396739	145396962	13chr1	149272886	149273083	13chr1	149293144	149293406	11chr1	149764513	149764742	9chr1	150551068	150551292	9chr1	151762775	151762997	10chr1	151809000	151809241	10chr1	151945454	151945704	12chr1	153328036	153328232	10chr1	153755899	153756166	12chr1	154732947	154733190	10chr1	154836251	154836484	12chr1	156470965	156471304	10chr1	156783657	156783942	10chr1	156829908	156830128	12chr1	157060002	157060185	11chr1	157125895	157126069	10chr1	157459218	157459440	11chr11	57508795	57508987	12chr1	159130864	159131062	10chr1	160645423	160645692	11chr1	160967200	160967433	10chr1	161102672	161102983	12chr11	61137183	61137425	10chr1	16127001	16127203	12chr11	61849604	61849819	11chr1	162025905	162026052	10chr11	62323034	62323226	10chr11	63604789	63605005	12chr11	63953452	63953702	10chr11	6411498	6411713	13chr11	64216382	64216608	9chr1	164742002	164742222	12chr11	65405403	65405700	10chr11	66185675	66185849	9chr11	6639354	6639619	10chr1	166879744	166879922	13chr1	167013373	167013576	10chr11	67044986	67045186	11chr11	67080180	67080349	11chr11	67120763	67121047	12chr11	67147068	67147327	13chr11	67340255	67340479	11chr11	68890255	68890523	11chr1	169332647	169332864	10chr11	69455877	69456083	12chr11	69703345	69703666	8chr1	17007360	17007605	12chr1	17029725	17029904	11chr1	172788119	172788359	13chr11	72896310	72896507	10chr11	73104669	73104867	10chr11	73999270	73999475	9chr11	75258685	75258850	9chr11	765340	765553	9chr1	17732656	17732918	10chr1	17764996	17765190	9chr11	78176474	78176703	14chr1	17820750	17820977	9chr11	79193926	79194117	12chr1	180485037	180485269	10chr11	8054818	8055021	10chr1	181135785	181136011	11chr1	182758673	182758965	12chr1	183029611	183029835	14chr1	184723917	184724167	9chr1	184836178	184836361	13chr1	185139568	185139895	10chr11	85375213	85375408	9chr11	86167249	86167415	10chr11	88090698	88090983	11chr11	88217987	88218240	9chr11	9082341	9082629	13chr1	192486044	192486244	11chr11	93517904	93518168	11chr11	93654982	93655221	10chr11	9779657	9779899	9chr1	199010707	199010969	12chr1	199212661	199212848	12chr1	19986325	19986571	10chr1	201223711	201223930	9chr1	201472676	201472891	9chr1	201951764	201952020	9chr1	202091802	202092052	10chr1	202317565	202317772	11chr1	203309314	203309532	10chr1	204386786	204386981	10chr1	204910906	204911115	10chr1	205680316	205680515	9chr1	206897172	206897434	8chr1	207038373	207038567	11chr1	207483052	207483290	10chr1	207693491	207693775	13chr1	208171214	208171406	8chr12	102233603	102233824	10chr12	104319073	104319279	8chr1	210574005	210574225	10chr1	21059640	21059917	7chr12	109022220	109022458	12chr12	109870643	109870813	8chr12	110476914	110477120	11chr12	113634820	113635008	11chr12	113652856	113653130	10chr12	114400282	114400492	8chr12	116355078	116355283	9chr12	120451692	120451943	10chr12	121021265	121021474	10chr1	212102425	212102625	9chr12	122062621	122062798	13chr12	123528097	123528300	8chr12	123755656	123755874	9chr12	124457287	124457504	12chr12	125256443	125256651	11chr12	125383068	125383265	8chr12	12941756	12941975	8chr1	214601252	214601446	11chr12	15374294	15374525	12chr1	21596747	21596954	10chr1	21620898	21621180	8chr12	17092836	17093061	9chr1	220950150	220950382	9chr1	223254747	223254983	11chr1	223307049	223307298	10chr1	226033528	226033764	11chr12	27037932	27038171	10chr12	27332834	27333017	11chr1	228187126	228187347	7chr12	28283678	28283912	9chr1	230241628	230241880	11chr12	3053879	3054090	10chr12	3143364	3143584	9chr12	31806143	31806416	12chr1	234859766	234860004	12chr1	23511214	23511462	11chr1	242161755	242162112	11chr12	42876643	42876916	10chr1	243759701	243759956	13chr12	4378498	4378648	9chr1	24525844	24526083	11chr12	46603782	46603985	10chr12	46611873	46612071	12chr1	246660678	246660872	10chr1	246917320	246917474	11chr1	24691800	24691986	10chr1	24739988	24740289	10chr1	247573302	247573526	11chr1	2480234	2480469	11chr12	49437674	49437838	8chr12	50449771	50450077	9chr12	50640966	50641177	9chr12	51058249	51058478	11chr12	51566554	51566859	11chr12	52766396	52766670	9chr12	54095633	54095829	9chr12	54389289	54389550	12chr12	54601616	54601836	9chr12	56123403	56123589	11chr12	562349	562560	10chr12	56367426	56367691	11chr12	56473688	56473914	10chr12	56586846	56587049	10chr12	57481682	57481939	10chr12	57569732	57569900	11chr12	57849969	57850221	11chr12	63118756	63119032	10chr12	63193000	63193225	11chr12	6394258	6394463	12chr12	65091874	65092059	10chr1	26611302	26611508	10chr1	26713962	26714156	11chr12	6735917	6736103	10chr1	26868377	26868623	10chr12	6944936	6945103	10chr12	69464153	69464370	11chr12	71127272	71127467	11chr1	27179686	27179840	10chr1	27189346	27189627	9chr1	27337554	27337800	11chr1	27371131	27371320	11chr1	27560729	27561031	9chr12	7791309	7791533	9chr12	80580505	80580732	11chr12	8261525	8261692	8chr12	93638018	93638212	11chr12	94954683	94954930	10chr12	96336591	96336901	7chr12	99147878	99148094	12chr13	101045062	101045344	12chr13	108867875	108868041	10chr13	113241759	113241967	10chr13	113242367	113242560	10chr1	31635586	31635837	8chr1	32681302	32681482	11chr1	32701716	32701993	12chr13	27272875	27273111	9chr13	27746029	27746314	12chr13	28404171	28404365	9chr1	32866489	32866803	8chr1	32879590	32879812	12chr13	30082392	30082548	10chr13	30689143	30689351	10chr13	31735993	31736179	9chr1	3369775	3369953	10chr13	39611908	39612184	9chr1	3407867	3408030	10chr13	45945195	45945391	12chr13	47371272	47371508	10chr13	48701128	48701314	11chr1	3535645	3535886	8chr1	36181097	36181349	11chr1	36184597	36184852	11chr1	36621350	36621583	10chr1	3663810	3664042	9chr1	36653837	36654058	12chr1	36840129	36840401	11chr1	36886496	36886654	11chr13	73664643	73664910	12chr13	76450597	76450835	10chr13	77903380	77903722	11chr13	78596423	78596589	13chr1	37920559	37920738	10chr13	80025771	80026003	12chr1	38261134	38261365	9chr13	96056617	96056776	11chr13	96130829	96131028	10chr1	39657834	39658100	10chr1	39876620	39876853	9chr13	99161745	99161990	12chr14	100883883	100884093	10chr14	101539652	101539822	10chr14	102173747	102174003	9chr14	103544684	103544925	10chr14	103828221	103828442	10chr14	104013140	104013356	9chr1	41341477	41341629	9chr1	41958812	41959035	11chr14	20999200	20999419	10chr14	24559763	24560012	12chr14	24615588	24615880	8chr14	32479833	32480101	10chr1	43402307	43402512	9chr1	43407499	43407705	11chr14	34261982	34262152	11chr14	34966151	34966431	12chr14	36843259	36843541	11chr14	37445769	37446049	11chr1	43824538	43824748	11chr1	44116064	44116284	8chr1	44395299	44395520	10chr1	44502286	44502548	9chr1	44951409	44951604	12chr14	51422455	51422736	11chr1	45484378	45484557	10chr14	55118940	55119160	10chr1	45978372	45978530	11chr14	59827931	59828196	12chr14	60631364	60631613	9chr14	61788234	61788476	12chr14	61992049	61992341	13chr14	62019703	62019940	9chr14	67993377	67993655	11chr1	46993700	46993948	10chr14	71375341	71375699	9chr14	72219874	72220062	11chr14	74238470	74238757	11chr14	74319325	74319576	10chr14	74718299	74718531	9chr14	75372424	75372647	10chr14	75688961	75689220	12chr14	75955189	75955415	12chr14	77383528	77383748	11chr1	47890141	47890364	10chr1	48176246	48176432	10chr14	82000274	82000539	11chr14	89506791	89507047	12chr14	93214904	93215154	8chr14	95065493	95065793	12chr14	95067036	95067307	12chr14	95794316	95794561	12chr15	100890340	100890566	10chr1	51796987	51797240	12chr15	22511148	22511331	10chr1	53685919	53686113	10chr1	53859072	53859273	9chr15	38856298	38856490	11chr15	39420505	39420745	12chr15	39872473	39872702	10chr15	40226080	40226269	12chr15	41187079	41187343	11chr15	42500400	42500639	8chr15	44719512	44719838	12chr15	45497402	45497584	10chr15	52263698	52264026	13chr1	55416367	55416672	8chr15	56783423	56783587	11chr15	57598841	57599111	10chr15	59158528	59158715	11chr15	64338632	64338838	10chr15	64539059	64539283	12chr15	65346722	65346902	10chr15	66911600	66911796	9chr15	67356372	67356667	11chr15	72520721	72521029	8chr15	73989411	73989690	11chr15	74695334	74695592	11chr15	74726126	74726343	11chr15	75074151	75074479	10chr15	75321737	75321891	10chr15	75359532	75359684	10chr15	75744162	75744470	10chr15	75871608	75871868	9chr15	76488860	76489158	9chr15	77480247	77480476	8chr15	78600789	78601069	10chr15	79103923	79104165	10chr15	79272103	79272314	10chr15	79603436	79603638	8chr15	79888461	79888655	9chr15	83194677	83194900	11chr15	84856535	84856726	10chr15	85473250	85473426	11chr15	86338036	86338218	9chr15	90905346	90905545	9chr15	91477575	91477753	12chr15	91497987	91498266	11chr15	93573938	93574152	7chr15	94867491	94867700	10chr1	6086194	6086432	9chr16	10275470	10275695	12chr16	10969085	10969343	7chr16	11047902	11048091	10chr16	11075661	11075878	10chr16	11318121	11318335	11chr16	14051679	14051869	10chr16	1428996	1429215	9chr16	193071	193273	14chr16	19532212	19532475	11chr16	20911643	20911931	12chr16	23193694	23193907	13chr16	24053402	24053624	11chr16	27437828	27438030	10chr16	29276689	29276964	11chr16	29711904	29712103	11chr16	30043627	30043840	10chr16	30503280	30503522	13chr16	30662588	30662845	12chr16	3072376	3072600	10chr16	30934214	30934541	8chr16	3151412	3151594	8chr16	3184872	3185053	11chr16	4635532	4635714	10chr16	46431404	46431540	9chr16	4666454	4666662	8chr16	47495913	47496160	10chr16	53491668	53491887	13chr16	54963090	54963284	9chr1	6550013	6550213	12chr16	578745	579012	9chr16	58664268	58664523	8chr16	66304432	66304670	8chr16	66517663	66517889	9chr16	66623354	66623575	11chr16	66785622	66785877	9chr1	66708423	66708627	10chr16	67462109	67462365	10chr16	67548328	67548512	13chr16	67694593	67694811	11chr16	67917670	67917879	9chr16	68624467	68624705	9chr16	69563533	69563783	8chr16	71533718	71533972	11chr16	72158831	72159043	10chr16	730213	730474	12chr16	75142901	75143142	12chr16	78133128	78133450	12chr16	80061061	80061331	12chr1	68154391	68154623	12chr16	81855154	81855395	10chr16	83988146	83988342	8chr16	83990464	83990617	10chr16	84118457	84118653	10chr16	84540060	84540274	11chr16	84627768	84628136	13chr16	85286805	85287057	8chr16	85403674	85403837	10chr16	85427390	85427575	8chr16	85650283	85650516	13chr16	85922494	85922688	11chr16	87905202	87905424	10chr16	88227747	88227934	11chr16	88272427	88272696	11chr16	88717902	88718117	10chr16	88878377	88878571	11chr16	89086724	89086992	11chr16	89282931	89283196	12chr16	89307643	89307837	10chr16	89940004	89940195	11chr16	9085820	9086046	8chr17	13241162	13241423	10chr17	14205974	14206189	10chr17	1533375	1533576	11chr17	16318275	16318500	11chr17	17584439	17584742	10chr17	17794401	17794588	10chr17	19241483	19241718	11chr17	19266233	19266421	9chr17	2148119	2148288	8chr17	2157350	2157539	13chr1	7278339	7278562	11chr17	2858352	2858616	11chr17	32198758	32198982	12chr17	32747377	32747583	10chr17	32965344	32965541	9chr17	35851470	35851728	11chr17	3614266	3614532	9chr17	36665666	36665932	11chr17	36740631	36740850	9chr17	37139485	37139644	9chr17	37779711	37780027	11chr17	38109565	38109809	11chr17	38738615	38738951	13chr17	39686530	39686693	7chr17	46185002	46185230	9chr17	47592547	47592790	12chr17	48045875	48046077	10chr17	48104244	48104477	8chr17	4812344	4812612	11chr17	48716586	48716887	11chr17	4900760	4901026	11chr1	75198807	75199005	11chr17	56413276	56413540	10chr17	56665057	56665317	11chr17	58513329	58513545	10chr1	7609414	7609609	11chr17	62024112	62024355	11chr17	62658227	62658466	10chr17	62957965	62958179	11chr17	62962025	62962324	12chr17	64455985	64456223	8chr17	6569398	6569603	10chr17	68133594	68133779	13chr17	70403612	70403883	11chr17	70461732	70461926	10chr17	70553108	70553334	10chr17	70723891	70724131	9chr17	7118269	7118534	12chr17	72195803	72196066	10chr17	7227594	7227821	11chr17	72297381	72297571	9chr17	72364468	72364700	11chr17	72426777	72426999	9chr17	72957740	72957993	11chr17	73120496	73120715	12chr17	73127713	73128008	9chr17	73236801	73236990	9chr17	73857483	73857685	8chr17	74275673	74275818	8chr17	75315455	75315673	9chr17	7620538	7620819	9chr17	76861891	76862102	9chr17	7748142	7748375	11chr17	7893522	7893743	10chr17	78997602	78997832	10chr17	79068710	79068949	12chr17	79227172	79227446	13chr17	79283081	79283327	10chr17	79979050	79979318	10chr17	80231291	80231498	9chr1	7831242	7831445	12chr17	9547955	9548159	9chr1	8035422	8035644	9chr18	10629112	10629308	10chr18	10929001	10929203	12chr18	11519102	11519329	10chr18	20510165	20510381	10chr18	20938826	20939060	9chr1	8227356	8227605	11chr18	25185292	25185472	10chr18	29665078	29665398	13chr18	322631	322844	11chr18	32923917	32924118	12chr18	32957133	32957352	10chr18	3526038	3526201	11chr18	3604735	3604963	11chr18	43201006	43201217	11chr18	46065199	46065434	10chr18	46190665	46190903	11chr18	48346370	48346599	11chr1	85086425	85086732	11chr18	52443577	52443810	12chr18	55888599	55888852	8chr18	56975828	56976020	9chr18	61616323	61616645	13chr18	65183211	65183426	9chr18	74775283	74775462	10chr18	75362837	75362999	8chr18	77551576	77551833	9chr19	1001703	1001947	9chr19	10444057	10444255	6chr19	10501461	10501658	12chr19	10975137	10975346	10chr19	11404550	11404725	14chr19	11671387	11671713	12chr19	11848760	11848982	10chr19	13387972	13388198	12chr19	14306957	14307118	11chr19	1503484	1503725	10chr19	1546247	1546449	10chr19	15489327	15489524	10chr19	15947569	15947829	9chr1	9161643	9161854	11chr19	16494135	16494309	11chr19	18111835	18112003	10chr19	18155881	18156042	13chr19	18169249	18169438	9chr19	18272136	18272371	10chr19	18794451	18794615	11chr19	19296806	19297047	8chr19	19314091	19314368	12chr19	19453586	19453813	10chr19	19483857	19484111	9chr19	21665933	21666043	8chr19	2887404	2887633	8chr19	2900596	2900875	11chr19	33616877	33617046	12chr19	34286301	34286552	10chr19	35535279	35535578	9chr19	35626370	35626602	10chr19	35959650	35959870	12chr19	37808745	37808981	9chr19	3822003	3822184	11chr19	38714794	38715058	10chr19	38724505	38724710	11chr19	39050214	39050433	10chr19	40922888	40923155	11chr19	41084470	41084684	13chr19	41769510	41769764	9chr19	41829502	41829717	12chr19	42688561	42688809	10chr19	42944546	42944739	10chr19	45195415	45195625	11chr19	45505084	45505279	8chr19	45905676	45905902	9chr19	45946844	45947072	9chr19	45949585	45949793	10chr19	47165338	47165515	11chr19	47494512	47494695	11chr19	47634017	47634276	11chr1	94801498	94801693	10chr19	48111236	48111482	10chr1	9486449	9486653	10chr19	50220801	50221018	9chr19	52049984	52050246	10chr19	53141417	53141675	10chr19	53324775	53324962	11chr19	53836938	53837098	9chr19	56015330	56015612	11chr19	58446716	58446912	11chr19	58662056	58662266	12chr19	663179	663399	10chr19	6767418	6767764	12chr19	7410423	7410764	8chr19	771343	771517	12chr19	7770559	7770831	12chr1	9779232	9779467	9chr19	8680697	8680900	9chr19	8942962	8943145	11chr1	99645958	99646123	10chr1	9989530	9989768	10chr20	1946391	1946579	7chr20	22664693	22664933	11chr20	23031498	23031740	10chr20	23136787	23137045	10chr20	2332499	2332779	10chr20	30434414	30434694	11chr20	30440591	30440750	10chr20	31683845	31684012	8chr20	31886968	31887197	8chr20	32319621	32319889	8chr20	32377516	32377707	10chr20	32400062	32400257	8chr20	328284	328525	10chr20	33857142	33857359	9chr20	34580697	34580917	9chr20	35012527	35012711	9chr20	36154785	36155041	10chr20	36738308	36738558	11chr20	3996123	3996364	10chr20	4072552	4072787	11chr20	412162	412386	10chr20	42077680	42077867	11chr20	42212602	42212834	11chr20	42379914	42380187	11chr20	42381194	42381417	12chr20	42709419	42709658	10chr20	43357513	43357767	11chr20	43972941	43973192	13chr20	44600999	44601320	9chr20	44642825	44643058	11chr20	46020903	46021125	11chr20	46097149	46097377	12chr20	47237065	47237318	9chr20	47274312	47274550	9chr20	48125243	48125410	10chr20	48391856	48392090	11chr20	49204728	49204999	9chr20	49547894	49548071	8chr20	5173024	5173236	10chr20	52029727	52029953	11chr20	52209087	52209315	10chr20	55125713	55125959	9chr20	55667200	55667434	9chr20	56172061	56172311	10chr20	56273251	56273621	10chr20	58146681	58146881	12chr20	58509125	58509312	9chr20	58786265	58786486	10chr20	61922617	61922866	11chr20	62670387	62670611	11chr20	9340317	9340600	9chr2	100175633	100175820	11chr2	101958742	101958934	10chr2	105986304	105986504	12chr2	109278698	109278901	12chr2	113627106	113627409	13chr2	113642537	113642769	10chr2	114128161	114128360	9chr2	114361782	114361903	11chr21	16810321	16810494	10chr2	120460495	120460761	9chr2	120980623	120980958	13chr2	121834603	121834812	8chr2	122457046	122457295	11chr2	122494297	122494532	10chr2	12596495	12596680	10chr21	26829772	26829969	11chr21	26980651	26980888	10chr2	127415546	127415785	10chr2	128643898	128644180	7chr21	34572174	34572404	10chr21	34817613	34817803	11chr21	35916307	35916487	12chr21	36238293	36238521	9chr21	43481551	43481812	13chr21	45031834	45032087	10chr21	45395374	45395636	9chr21	46300857	46301084	11chr2	157287648	157287843	9chr2	158184155	158184365	9chr2	166810608	166810813	10chr2	16790315	16790550	10chr2	172949967	172950215	9chr2	178013715	178013882	9chr2	178589488	178589689	9chr2	182675575	182675805	9chr2	192540612	192540813	12chr2	19340600	19340796	12chr2	20776431	20776672	13chr2	208394277	208394562	12chr2	209260035	209260283	10chr2	217547204	217547448	10chr22	17565687	17565935	9chr2	218654002	218654211	9chr2	219122354	219122645	12chr2	219232477	219232649	11chr2	219844794	219845024	9chr2	219894487	219894670	9chr2	220462774	220462972	9chr22	21051870	21052073	10chr22	22676464	22676610	11chr22	23301408	23301667	11chr22	24236172	24236489	9chr2	227701074	227701280	10chr22	29427387	29427639	10chr2	231812710	231813021	12chr2	232046619	232046845	13chr2	232277116	232277339	9chr2	233476379	233476616	10chr22	36461952	36462215	11chr2	237086767	237086950	11chr22	37153726	37153900	10chr22	37545986	37546171	10chr22	37988587	37988766	10chr22	38073100	38073354	12chr22	38624800	38625033	11chr2	238683424	238683724	11chr22	39570802	39571044	11chr2	241373438	241373696	10chr22	41993387	41993586	10chr22	42565075	42565273	9chr2	242674776	242675037	9chr2	24283806	24284027	11chr2	24387332	24387583	9chr22	45216558	45216769	9chr22	46034938	46035213	11chr22	46374996	46375196	8chr22	46938277	46938450	11chr22	47154781	47154999	10chr22	50679559	50679836	12chr2	26552960	26553147	11chr2	26968672	26968885	11chr2	26991931	26992193	11chr2	28499061	28499269	10chr2	33171353	33171567	13chr2	43251748	43251929	11chr2	44396706	44396982	13chr2	46636225	46636504	9chr2	53994578	53994871	11chr2	54196481	54196729	8chr2	67691070	67691297	9chr2	6913326	6913569	8chr2	70520776	70521025	8chr2	71016742	71016971	10chr2	74730377	74730625	10chr2	74734325	74734620	10chr2	7965848	7966129	11chr2	86054615	86054816	10chr2	88650490	88650730	9chr2	97151816	97152018	10chr2	97202838	97203066	10chr2	9910575	9910779	10chr3	10334587	10334792	12chr3	106954829	106954998	9chr3	111732406	111732616	10chr3	112361835	112362037	11chr3	114425510	114425712	8chr3	115411859	115412071	12chr3	115509665	115509913	11chr3	119278341	119278632	10chr3	119319598	119319849	12chr3	124147995	124148258	12chr3	124407709	124407938	10chr3	126398180	126398386	8chr3	126422881	126423080	11chr3	127309512	127309802	7chr3	127371056	127371323	7chr3	127409798	127410043	10chr3	128564787	128565018	8chr3	130568983	130569288	12chr3	13494328	13494567	10chr3	13537831	13538035	11chr3	138655116	138655286	8chr3	141050611	141050867	12chr3	146221525	146221797	11chr3	150480953	150481240	12chr3	167098024	167098227	11chr3	171547810	171548003	11chr3	178198636	178198892	13chr3	183003667	183003849	11chr3	183100250	183100440	10chr3	18336749	18336972	9chr3	183735639	183735823	8chr3	184521109	184521319	11chr3	185293206	185293431	11chr3	185618122	185618317	12chr3	185695295	185695578	11chr3	185935655	185935871	11chr3	186258341	186258585	12chr3	187397487	187397667	7chr3	192610901	192611146	11chr3	193505986	193506197	9chr3	194722680	194723009	9chr3	194980765	194981068	11chr3	195603631	195603900	12chr3	195800196	195800368	12chr3	196705613	196705841	12chr3	23987658	23987895	13chr3	25824331	25824567	11chr3	32219470	32219693	10chr3	39680913	39681068	8chr3	40547380	40547571	10chr3	41013158	41013450	10chr3	42232727	42232929	9chr3	46908286	46908558	11chr3	50579919	50580113	10chr3	51671962	51672138	8chr3	52287324	52287557	14chr3	53304118	53304319	10chr3	57937198	57937463	9chr3	64016451	64016655	10chr3	72319227	72319457	9chr3	72572585	72572844	10chr3	9926246	9926470	10chr3	9988906	9989140	11chr4	1161018	1161235	8chr4	119274074	119274297	9chr4	119756248	119756476	9chr4	123638082	123638257	12chr4	129474047	129474291	10chr4	139951888	139952089	9chr4	141174393	141174645	10chr4	142228433	142228677	13chr4	144434362	144434685	9chr4	159651232	159651431	11chr4	1728040	1728231	10chr4	185305520	185305736	8chr4	185745720	185745927	8chr4	23335626	23335814	9chr4	25915925	25916174	8chr4	26131875	26132040	8chr4	38130634	38130871	10chr4	38761198	38761426	10chr4	47567316	47567560	11chr4	47928656	47928862	10chr4	57167727	57167911	10chr4	6785132	6785341	11chr4	69241732	69241963	10chr4	736487	736692	11chr4	80994306	80994629	11chr4	81105279	81105474	12chr4	8160560	8160785	10chr4	87857292	87857552	11chr4	89377910	89378152	10chr4	99064489	99064766	11chr5	100163816	100164066	11chr5	102090855	102091097	10chr5	110866029	110866301	12chr5	111887223	111887407	10chr5	125758741	125758992	9chr5	131343596	131343786	11chr5	131630875	131631097	9chr5	131705252	131705551	10chr5	132583660	132583823	10chr5	134761824	134762049	10chr5	134793225	134793409	10chr5	134905972	134906136	9chr5	136556250	136556500	9chr5	137404492	137404728	8chr5	137548915	137549138	10chr5	138088741	138088977	9chr5	141021528	141021721	11chr5	147237145	147237371	12chr5	148822189	148822338	11chr5	149217307	149217507	10chr5	149541267	149541440	11chr5	149730121	149730396	8chr5	149838445	149838636	10chr5	150593628	150593908	10chr5	151038573	151038778	10chr5	153900578	153900823	11chr5	156575942	156576131	8chr5	158521891	158522127	13chr5	159507869	159508143	11chr5	171601703	171601941	11chr5	172755398	172755621	8chr5	173739060	173739269	9chr5	17499660	17499809	11chr5	176297083	176297267	10chr5	176884628	176884898	7chr5	177034502	177034737	9chr5	178450599	178450833	11chr5	33850283	33850522	11chr5	34686962	34687214	11chr5	34839153	34839388	11chr5	43313587	43313929	11chr5	443124	443313	11chr5	52096563	52096896	10chr5	61519285	61519442	10chr5	68338749	68338998	10chr5	69696800	69697015	11chr5	73928039	73928285	13chr5	74616809	74617094	11chr5	76163929	76164194	7chr5	79154032	79154221	10chr5	82726295	82726483	12chr5	89561153	89561360	11chr5	89940074	89940256	10chr5	923609	923810	9chr5	95046677	95046974	13chr6	10723223	10723466	10chr6	108885509	108885709	10chr6	109156527	109156711	10chr6	112484578	112484793	11chr6	11732420	11732662	9chr6	119256180	119256472	11chr6	120583369	120583583	10chr6	127587628	127587884	10chr6	129822593	129822833	11chr6	130203761	130203911	10chr6	1382053	1382330	12chr6	14067070	14067359	12chr6	143193069	143193288	13chr6	146349443	146349701	10chr6	151186649	151186865	7chr6	152492257	152492521	10chr6	156885610	156885824	10chr6	158184461	158184673	12chr6	16131217	16131486	11chr6	161795003	161795197	9chr6	16206861	16207079	11chr6	166814363	166814554	9chr6	17815647	17815834	12chr6	24521236	24521509	10chr6	26064871	26065021	11chr6	26158285	26158501	7chr6	30187018	30187235	8chr6	30524231	30524418	10chr6	31036666	31036896	10chr6	31409405	31409660	10chr6	32164225	32164450	9chr6	32383301	32383481	10chr6	32952633	32952816	10chr6	33267448	33267726	11chr6	33558032	33558235	11chr6	33730015	33730229	9chr6	33739363	33739628	10chr6	34212336	34212528	13chr6	34495022	34495208	11chr6	34625287	34625489	12chr6	34856884	34857141	11chr6	35941548	35941739	11chr6	36095384	36095621	11chr6	36989551	36989838	11chr6	3978547	3978777	9chr6	41246815	41247011	12chr6	41673985	41674146	8chr6	43597038	43597290	10chr6	43636042	43636269	9chr6	45649684	45649922	9chr6	5030992	5031212	8chr6	52092378	52092595	10chr6	52382402	52382646	11chr6	52442039	52442280	12chr6	6557291	6557437	10chr6	6712851	6713030	11chr6	71079119	71079315	12chr6	7236552	7236768	10chr6	7698381	7698673	10chr6	86352982	86353178	12chr6	88639582	88639776	10chr6	90348251	90348546	12chr6	92192262	92192459	9chr6	97345750	97345938	11chr7	100322932	100323129	11chr7	100702726	100702888	8chr7	100787331	100787553	12chr7	101245793	101245940	9chr7	101377050	101377312	10chr7	101950294	101950501	13chr7	102516328	102516513	11chr7	104621751	104621993	10chr7	105042153	105042349	9chr7	105856963	105857184	12chr7	106358734	106358930	10chr7	106416123	106416360	11chr7	106809024	106809240	11chr7	1068494	1068771	10chr7	115851443	115851697	10chr7	117228460	117228687	8chr7	120432167	120432378	10chr7	120590490	120590803	8chr7	129650718	129650917	10chr7	129665850	129666124	11chr7	131373953	131374131	10chr7	131376878	131377099	9chr7	135196972	135197202	9chr7	138701902	138702138	11chr7	138794402	138794622	9chr7	13889800	13890045	10chr7	139876920	139877115	7chr7	140353257	140353455	10chr7	142148306	142148478	9chr7	143042555	143042744	12chr7	143599101	143599383	9chr7	148611409	148611616	9chr7	148659896	148660123	13chr7	151012269	151012420	11chr7	151329416	151329649	10chr7	154799708	154799925	11chr7	1552997	1553226	11chr7	158497623	158497882	10chr7	16779900	16780142	8chr7	22599569	22599808	9chr7	2276977	2277213	12chr7	23095296	23095483	9chr7	23513932	23514191	11chr7	2446455	2446702	10chr7	24861098	24861267	10chr7	26404785	26404967	11chr7	2760572	2760758	10chr7	28060003	28060216	9chr7	2904742	2904933	10chr7	30028109	30028285	10chr7	30174096	30174372	11chr7	30265617	30265853	12chr7	30452710	30452917	9chr7	31023318	31023468	9chr7	32545987	32546173	11chr7	32931766	32931979	9chr7	33102122	33102405	10chr7	37324389	37324576	9chr7	37723223	37723443	10chr7	38700195	38700367	8chr7	39620060	39620281	10chr7	42980920	42981111	9chr7	43556613	43556830	11chr7	43612131	43612364	9chr7	43946080	43946306	8chr7	45893928	45894161	12chr7	47709146	47709378	9chr7	51434841	51435057	11chr7	5490387	5490580	10chr7	5635760	5636013	13chr7	5896151	5896355	10chr7	66843240	66843482	11chr7	71198338	71198520	7chr7	74075360	74075589	9chr7	76009432	76009664	10chr7	77649092	77649305	11chr7	86941682	86941852	11chr7	948528	948719	10chr7	98062686	98062890	8chr7	99591858	99592032	10chr7	99818885	99819112	11chr7	99942487	99942745	12chr8	101839297	101839472	11chr8	101859416	101859624	10chr8	102110804	102111007	10chr8	103743311	103743537	10chr8	10657353	10657586	11chr8	110346484	110346700	8chr8	118057017	118057202	10chr8	121096897	121097063	9chr8	125860361	125860548	9chr8	126231574	126231790	12chr8	128209208	128209394	11chr8	12899913	12900079	10chr8	130983807	130984144	13chr8	131027547	131027731	10chr8	131206986	131207208	11chr8	134215195	134215395	10chr8	134581155	134581401	10chr8	141841288	141841484	9chr8	142129798	142129979	9chr8	143553341	143553609	10chr8	143799528	143799764	11chr8	144129673	144129908	10chr8	144212533	144212728	11chr8	145056679	145056868	8chr8	18925112	18925365	11chr8	22926549	22926725	10chr8	23315060	23315297	9chr8	23329936	23330104	9chr8	23550213	23550414	9chr8	25946154	25946407	11chr8	28221021	28221226	13chr8	28269328	28269660	12chr8	29815871	29816093	11chr8	30892207	30892469	9chr8	33412426	33412616	9chr8	37738368	37738601	12chr8	38326080	38326294	10chr8	38571920	38572152	9chr8	38578911	38579181	10chr8	59425883	59426085	12chr8	67089166	67089431	12chr8	72740257	72740407	11chr8	72743589	72743841	10chr8	74432930	74433226	13chr8	80705932	80706165	10chr8	80935855	80936114	10chr8	90771540	90771754	9chr8	95092037	95092223	10chr8	95487163	95487348	8chr9	100361007	100361262	12chr9	104292455	104292643	11chr9	104603042	104603377	13chr9	110517638	110517888	12chr9	112665932	112666106	9chr9	115875181	115875373	10chr9	116279512	116279758	11chr9	116569702	116569964	11chr9	116916093	116916352	10chr9	117249963	117250184	10chr9	117670476	117670682	12chr9	119499442	119499608	10chr9	120249895	120250135	9chr9	123493011	123493195	11chr9	124044208	124044399	13chr9	125215511	125215714	8chr9	126105745	126105982	13chr9	126803324	126803503	9chr9	127632610	127632788	9chr9	12776306	12776557	10chr9	127962621	127962891	10chr9	129949913	129950088	9chr9	131798705	131798909	10chr9	132258998	132259191	10chr9	132332995	132333176	9chr9	133710021	133710254	9chr9	133739049	133739261	10chr9	134053714	134053965	12chr9	135777321	135777518	7chr9	135978621	135978814	9chr9	136351478	136351707	11chr9	136933085	136933287	10chr9	138235838	138236054	9chr9	138495816	138495997	9chr9	138987007	138987215	10chr9	139159238	139159457	7chr9	139240348	139240589	10chr9	139655364	139655586	9chr9	139916573	139916901	9chr9	140506682	140506937	10chr9	14993162	14993395	11chr9	19999617	19999810	11chr9	22008634	22008846	13chr9	2570685	2570956	13chr9	34049376	34049654	10chr9	34548076	34548310	10chr9	34899657	34899863	10chr9	35071918	35072174	10chr9	36151919	36152117	10chr9	36765911	36766196	9chr9	37485628	37485934	9chr9	38620516	38620754	11chr9	7298184	7298381	11chr9	86264093	86264257	10chr9	86876108	86876308	9chr9	87324702	87324926	13chr9	90328317	90328639	11chr9	90407793	90407993	11chr9	91362736	91363006	11chr9	92078930	92079113	8chr9	93955332	93955544	11chr9	95397061	95397243	10chr9	95553106	95553308	11chr9	95871486	95871710	9chr9	95878406	95878576	13chr9	96725310	96725513	12chr9	99019173	99019446	10chr9	99182735	99182999	11chrX	122648287	122648534	12chrX	134477882	134478085	9chrX	140000619	140000891	10chrX	149940705	149940971	9chrX	152599560	152599727	8chrX	153059745	153060064	11chrX	154209171	154209414	10chrX	15511286	15511534	9chrX	1599822	1600085	12chrX	16141462	16141694	8chrX	2511406	2511622	13chrX	2625476	2625691	11chrX	30731379	30731565	10chrX	35908862	35909106	10chrX	37706829	37707051	11chrX	39714998	39715205	11chrX	40028841	40029107	11chrX	41135453	41135728	10chrX	43417162	43417336	9chrX	45046184	45046416	10chrX	49969290	49969502	10chrX	51546026	51546246	10chrX	55026651	55026928	13chrX	58564973	58565160	12chrX	7894017	7894165	10chrX	9963931	9964108	11chr10	100424318	100424505	7chr10	102098313	102098469	10chr10	102810031	102810252	10chr10	103227112	103227310	10chr10	103832766	103832908	9chr10	104005221	104005414	7chr10	104262486	104262705	8chr10	104401862	104402167	12chr10	104489036	104489301	8chr10	104513045	104513283	9chr10	104952338	104952550	8chr10	106113343	106113544	9chr10	112185724	112185950	9chr10	11220778	11220962	9chr10	116231203	116231383	9chr10	11720203	11720375	10chr10	121176475	121176672	9chr10	121410851	121411046	7chr10	12490750	12490986	10chr10	127584935	127585202	11chr10	129652319	129652508	10chr10	133956037	133956222	9chr10	2154206	2154460	8chr10	24906793	24906943	9chr10	25351458	25351664	11chr10	27026755	27026926	12chr10	2992180	2992398	12chr10	31996659	31996920	11chr10	32229800	32230096	8chr10	3281852	3282074	9chr10	33297718	33297936	11chr10	3524606	3524834	9chr10	35852299	35852490	11chr10	3825100	3825346	12chr10	3848092	3848289	11chr10	44069871	44070129	11chr10	51623239	51623444	11chr10	6183183	6183417	11chr10	70853101	70853302	11chr10	72237528	72237753	10chr10	7567433	7567643	10chr10	81918434	81918650	10chr10	82213566	82213777	12chr10	88280697	88280904	8chr10	88730835	88731190	10chr10	93861168	93861402	12chr10	93971099	93971333	11chr10	98135086	98135309	7chr10	98155750	98156005	11chr10	99302928	99303179	9chr1	10519270	10519454	11chr1	108478124	108478343	11chr11	101982750	101983007	9chr11	107436329	107436532	7chr1	111067194	111067346	10chr11	111797508	111797782	8chr11	113160342	113160569	9chr1	111422623	111422890	12chr11	117186894	117187142	10chr11	118481762	118482018	10chr11	119191725	119191988	9chr11	119345508	119345672	9chr11	119979001	119979209	6chr11	120215102	120215342	11chr1	112135464	112135673	10chr11	121460873	121461149	9chr11	1224302	1224536	9chr11	124543692	124543944	10chr11	125744478	125744688	8chr11	126223306	126223502	8chr11	129013304	129013554	10chr11	129716423	129716673	9chr11	130083062	130083297	10chr11	131557947	131558128	10chr11	134123273	134123487	10chr11	14929359	14929524	7chr1	115722156	115722447	9chr1	11610817	11610991	10chr1	116191662	116191827	10chr11	16807088	16807321	9chr1	11710586	11710775	9chr1	117360338	117360517	11chr1	11779920	11780094	10chr11	19681769	19681983	9chr1	120156501	120156661	9chr1	120218291	120218477	8chr1	120413597	120413834	9chr1	120510714	120510941	9chr1	120925503	120925686	8chr11	22897538	22897738	10chr11	2449519	2449768	9chr1	12509609	12509850	11chr11	27231238	27231413	8chr11	2951995	2952213	11chr11	32109636	32109898	6chr11	33966355	33966583	13chr11	34018643	34018849	8chr11	34672130	34672340	11chr1	1376383	1376564	10chr11	3941458	3941622	8chr11	44058957	44059212	10chr11	44307945	44308149	10chr11	44560055	44560262	10chr11	44883843	44884057	10chr11	44932907	44933179	9chr11	45127123	45127303	8chr11	45939328	45939631	10chr11	45986495	45986693	10chr11	46258786	46258993	9chr11	46382930	46383189	10chr11	46413435	46413690	7chr11	46639316	46639570	9chr1	146861712	146861934	11chr11	47182645	47182844	9chr11	48194495	48194696	9chr1	149204338	149204511	8chr1	150138243	150138442	9chr1	150227828	150228026	9chr1	150533508	150533732	10chr1	150895074	150895321	10chr1	151030291	151030572	11chr1	151906567	151906755	8chr11	537362	537578	9chr1	153769754	153769929	9chr1	15480588	15480902	10chr1	155058607	155058819	10chr1	155107585	155107808	9chr1	155910525	155910789	11chr1	15599037	15599277	9chr1	155995757	155995979	9chr1	156096107	156096312	10chr1	156450598	156450763	10chr1	156890266	156890418	11chr1	157076284	157076487	11chr11	57200269	57200496	11chr1	157465951	157466128	10chr1	15828010	15828177	9chr1	15911042	15911276	9chr1	160048490	160048682	9chr1	160616703	160616941	8chr11	60666889	60667096	9chr1	160696695	160696857	10chr1	16142117	16142351	11chr1	16162340	16162578	12chr11	61891120	61891445	10chr11	62092446	62092665	9chr11	64014797	64015164	10chr11	64420613	64420832	10chr11	64555130	64555306	9chr1	16499711	16499916	11chr11	65097446	65097627	9chr11	65260380	65260690	11chr11	65278265	65278481	9chr1	165362230	165362461	9chr11	65420436	65420646	9chr11	65511990	65512207	10chr11	65537328	65537561	9chr11	65835730	65835963	9chr11	66346607	66346778	11chr11	66849034	66849210	9chr11	67156987	67157207	9chr11	67325597	67325820	11chr11	67423849	67424054	10chr1	167632278	167632491	9chr1	168311997	168312212	12chr11	68856229	68856457	11chr11	69059826	69060073	10chr1	170500872	170501186	7chr11	71010409	71010637	10chr11	71781033	71781266	9chr1	172419126	172419323	8chr1	172714203	172714426	10chr1	17278233	17278417	10chr11	72953752	72953912	8chr1	174126342	174126569	8chr11	74851654	74851863	9chr11	75099821	75100049	11chr11	75434247	75434458	10chr1	17563577	17563788	7chr11	75919827	75920155	10chr11	77711286	77711446	9chr11	82774312	82774526	11chr11	85827440	85827635	5chr11	86235269	86235486	10chr1	18908000	18908163	9chr11	94226979	94227262	11chr1	198906455	198906686	10chr1	200457884	200458126	9chr1	201430352	201430554	8chr1	202046063	202046299	10chr1	20244484	20244778	11chr1	202569343	202569512	10chr1	202896099	202896360	10chr1	202928385	202928589	8chr1	203275769	203275985	10chr1	204616737	204616952	8chr1	205294616	205294814	10chr1	205626755	205626953	12chr1	205718586	205718846	12chr1	206677474	206677694	9chr1	206748529	206748764	11chr1	206831918	206832160	8chr1	207251235	207251480	8chr1	208058476	208058683	10chr1	209527733	209527955	8chr1	209866313	209866504	10chr1	21022921	21023186	10chr12	104775678	104775920	11chr1	2105803	2105967	10chr12	109124120	109124359	9chr12	110540108	110540320	8chr12	112191089	112191283	9chr12	112250974	112251178	9chr12	112825233	112825388	11chr12	114350592	114350787	9chr12	115100488	115100692	10chr12	117197638	117197772	9chr12	118620120	118620333	11chr12	120755173	120755495	7chr1	212118161	212118387	9chr12	121668164	121668378	10chr12	122120075	122120209	9chr12	122510546	122510768	9chr12	123210652	123210821	11chr1	212464880	212465128	10chr1	212629308	212629477	8chr1	212631182	212631366	9chr12	127359007	127359229	7chr12	12735908	12736151	7chr12	13023509	13023721	8chr12	131246318	131246516	8chr12	131617018	131617183	9chr1	21697131	21697294	11chr1	21996345	21996567	6chr1	221651438	221651660	10chr1	221840900	221841101	10chr1	222628569	222628726	10chr12	25054002	25054194	11chr1	225506760	225506953	10chr12	25540428	25540665	10chr12	26267354	26267668	11chr1	226814201	226814492	10chr12	28325453	28325693	7chr12	2893219	2893465	8chr1	229650436	229650674	8chr12	2994528	2994684	10chr12	32259558	32259773	11chr1	23256837	23257056	10chr12	32832018	32832226	8chr1	233926910	233927137	9chr12	34276095	34276293	10chr1	234462396	234462628	10chr1	235120170	235120405	10chr1	235492168	235492395	10chr1	236320044	236320260	7chr1	23854726	23854979	10chr1	24137328	24137521	9chr1	244624602	244624886	11chr12	45609629	45609915	11chr12	46943650	46943832	9chr12	47377878	47378020	10chr12	49525032	49525226	9chr1	25236025	25236247	10chr12	52397935	52398132	9chr12	52477253	52477483	7chr12	52513828	52514034	10chr1	25255278	25255525	8chr12	53297738	53297969	11chr12	53459808	53460043	9chr1	25348955	25349166	8chr12	54219152	54219386	12chr12	54753279	54753575	6chr12	54891333	54891504	11chr12	56323153	56323413	12chr12	57146031	57146359	12chr12	58015541	58015809	9chr12	58240418	58240657	9chr12	6444912	6445091	9chr12	6647119	6647312	10chr12	6772251	6772475	11chr12	6862150	6862364	8chr12	7000365	7000673	10chr12	71039665	71039930	10chr1	27160050	27160328	13chr1	27293433	27293645	9chr1	27339368	27339586	9chr12	76034852	76035046	10chr1	27895068	27895282	9chr12	79738727	79738912	8chr1	28157162	28157304	10chr1	28575061	28575291	11chr1	28640508	28640772	10chr12	864122	864343	10chr12	88429244	88429418	8chr12	89413437	89413598	10chr12	90103101	90103442	9chr12	93965258	93965486	12chr12	94494097	94494319	9chr12	95034359	95034587	11chr12	9917463	9917681	11chr12	9950688	9950870	12chr12	99830477	99830673	11chr13	100039808	100039983	10chr13	110438848	110439034	11chr13	111245946	111246126	10chr13	111596698	111596924	9chr13	113366097	113366339	10chr1	31181032	31181216	10chr1	31628179	31628436	11chr13	20161114	20161381	9chr13	21069347	21069514	9chr13	21517746	21517975	9chr1	32170833	32171027	11chr1	32254178	32254342	10chr13	23949550	23949733	8chr13	27521136	27521324	9chr13	27998614	27998904	12chr13	28000075	28000266	10chr1	33338284	33338532	10chr13	34302850	34303086	11chr1	33472903	33473127	10chr1	3370819	3371053	10chr1	33761166	33761347	11chr1	33815588	33815798	13chr1	33896543	33896797	8chr13	42979970	42980193	10chr13	45941549	45941785	11chr13	46895799	46896016	10chr13	47125717	47125942	9chr13	50819279	50819491	9chr1	36034625	36034838	11chr13	63217188	63217371	11chr13	78167285	78167470	11chr1	37942872	37943199	10chr1	38388542	38388808	9chr13	97793910	97794131	11chr1	40119250	40119481	11chr1	40132465	40132716	12chr1	40942730	40942985	10chr14	100872957	100873168	9chr14	101112177	101112329	11chr14	102701712	102701925	10chr14	105106387	105106597	10chr14	105491239	105491440	10chr14	106626978	106627147	10chr14	106877408	106877588	9chr1	41078072	41078249	9chr1	41239937	41240181	8chr14	21575096	21575321	11chr14	23464012	23464229	7chr14	24527532	24527761	10chr1	43388706	43388887	9chr14	34567949	34568165	10chr1	43539324	43539534	10chr14	35451998	35452294	9chr14	36295545	36295875	11chr1	43664852	43665098	10chr1	44172975	44173162	11chr14	50332126	50332336	11chr14	50527859	50528042	9chr1	45060891	45061067	9chr14	52311233	52311465	9chr1	45273411	45273600	9chr14	53173745	53174011	8chr1	46009567	46009821	11chr14	63938072	63938258	9chr1	46648893	46649171	11chr14	67880894	67881164	13chr14	67893947	67894171	10chr14	69086973	69087212	11chr14	69378374	69378594	10chr14	69596051	69596278	11chr14	70540730	70540931	11chr14	71373979	71374140	10chr1	47190967	47191160	11chr14	72887548	72887763	10chr14	74037731	74037939	9chr14	74229245	74229410	12chr14	76618031	76618326	10chr14	77519629	77519843	11chr14	77525903	77526193	11chr14	77767445	77767666	9chr14	77923945	77924173	10chr1	47800298	47800555	11chr14	78082948	78083150	10chr14	81687595	81687872	8chr14	81769727	81769983	11chr1	48192046	48192198	11chr14	94603114	94603301	10chr14	99786403	99786666	10chr1	50798732	50798960	11chr15	100468652	100468871	11chr15	101555505	101555681	9chr15	101688731	101688963	12chr15	101798426	101798645	10chr15	102192449	102192665	9chr15	29960353	29960519	9chr15	33162626	33162896	9chr15	37392503	37392749	8chr15	37393377	37393659	10chr1	53743978	53744214	9chr15	38732581	38732740	9chr1	53910341	53910513	9chr1	53935839	53936065	10chr15	39464324	39464526	11chr1	53951212	53951418	9chr15	40339962	40340181	12chr15	40636848	40636995	10chr15	40812073	40812202	10chr15	41074915	41075067	10chr15	41151427	41151625	9chr15	42351969	42352164	10chr15	51296374	51296595	10chr15	51673505	51673703	11chr15	52303182	52303359	8chr15	52393100	52393282	11chr15	55571948	55572175	12chr15	58358528	58358818	10chr15	59590565	59590755	9chr15	60878037	60878235	9chr15	66859411	66859648	10chr15	66990812	66990954	8chr15	67239120	67239337	11chr15	67442792	67443048	10chr15	68842910	68843094	11chr15	69168220	69168443	10chr15	69768184	69768413	9chr15	70006592	70006832	10chr15	71184743	71184928	10chr15	74189833	74189989	9chr15	74804481	74804732	12chr15	74890680	74890910	8chr15	75932568	75932764	10chr15	77473995	77474213	11chr15	78236803	78237019	11chr15	78286409	78286631	10chr15	78533379	78533545	11chr15	79030532	79030756	10chr15	80145922	80146137	8chr1	5805931	5806118	8chr15	81071349	81071573	10chr15	82810334	82810557	11chr15	84961516	84961713	9chr15	85590543	85590753	11chr15	85735621	85735810	10chr15	86397690	86397875	11chr15	89164424	89164639	7chr15	91073221	91073475	8chr15	91229743	91230018	13chr15	91499808	91500042	11chr1	59485918	59486160	9chr15	99322870	99323148	10chr15	99526644	99526888	10chr16	1105122	1105346	10chr16	11405637	11405847	11chr16	11450575	11450793	10chr16	12913283	12913501	10chr16	12990483	12990700	10chr16	13530607	13530907	11chr16	16090569	16090824	10chr16	17164784	17165014	7chr1	61784533	61784804	9chr16	20885877	20886188	12chr16	23812816	23812990	11chr16	24048044	24048249	10chr16	27166398	27166603	9chr16	2720145	2720296	9chr16	28328415	28328636	9chr16	2885805	2886017	10chr16	2892586	2892836	10chr16	30103222	30103480	10chr16	30968324	30968563	9chr1	63156049	63156296	10chr16	3181799	3182073	11chr16	33458965	33459142	10chr16	34187348	34187457	8chr1	63793337	63793505	9chr16	46707400	46707568	9chr16	48151454	48151670	8chr16	4818695	4818975	10chr1	6483545	6483745	9chr16	48664404	48664595	8chr16	50717918	50718167	11chr16	53552718	53552913	13chr1	65533342	65533628	9chr16	58790377	58790545	11chr1	65886351	65886556	8chr16	619832	620112	11chr1	66648308	66648558	8chr16	66556144	66556326	11chr16	66638727	66638983	9chr16	67294674	67294861	11chr16	676900	677116	10chr16	67706733	67706947	8chr16	67970693	67970930	11chr16	68108765	68109045	11chr16	69146648	69146866	10chr16	69430072	69430286	7chr16	69796085	69796257	11chr16	70415194	70415366	8chr16	70812938	70813182	9chr1	67191783	67191995	9chr16	73323165	73323371	12chr16	74141794	74142036	9chr16	75505789	75505928	9chr16	78804581	78804816	10chr16	81756817	81757034	12chr16	81831112	81831394	11chr16	82687531	82687817	11chr1	68319412	68319618	10chr16	83859760	83859940	8chr16	84788009	84788276	10chr16	84958811	84959034	9chr16	85004251	85004457	8chr16	85603425	85603716	11chr16	85648678	85648899	11chr16	86374369	86374560	10chr16	87902968	87903142	9chr16	87929251	87929470	7chr16	88850702	88850948	9chr16	89034579	89034818	9chr16	89092577	89092827	11chr16	89707661	89707857	8chr16	89767362	89767545	8chr16	9144373	9144547	9chr16	9146980	9147259	9chr16	9742037	9742258	9chr17	1101398	1101682	11chr1	71151042	71151249	10chr17	16891476	16891744	9chr17	17597558	17597845	9chr17	17763632	17763795	9chr17	18151633	18151846	9chr17	18804205	18804437	12chr17	2119228	2119419	13chr17	2285892	2286111	9chr17	25783303	25783545	8chr17	26743888	26744114	10chr17	27062347	27062617	11chr17	27070130	27070423	8chr17	27090796	27091047	11chr17	27198233	27198486	11chr17	27438922	27439100	9chr17	28431633	28431897	10chr17	29025014	29025253	11chr17	29421808	29422018	7chr17	32291462	32291668	10chr17	34959401	34959613	9chr17	36823866	36824040	9chr17	37356165	37356473	9chr17	37793293	37793582	12chr17	3791867	3792199	9chr17	38443791	38444042	7chr17	38514831	38515056	11chr17	38776296	38776480	9chr17	40464307	40464546	9chr17	40683958	40684119	8chr17	40833294	40833584	9chr17	42072337	42072500	10chr17	42264209	42264419	10chr17	43201434	43201659	10chr17	44364122	44364349	11chr17	45365958	45366174	12chr17	46555747	46555987	12chr17	46755813	46756071	9chr17	47826261	47826421	11chr17	48074016	48074212	9chr17	49538971	49539159	10chr17	5130824	5131048	11chr17	5322931	5323202	10chr17	58156000	58156232	9chr17	61484160	61484385	10chr17	62398447	62398685	10chr17	62919149	62919335	12chr17	64711907	64712107	10chr17	65989816	65990046	10chr17	66434221	66434426	9chr17	67089539	67089738	11chr17	71287649	71287852	10chr17	73500471	73500664	8chr17	73553173	73553396	11chr17	74380474	74380780	12chr17	74639694	74639946	10chr17	75118687	75118884	11chr17	75458239	75458483	9chr17	75461759	75461979	9chr17	76143786	76143994	8chr17	76172668	76172870	9chr17	76259354	76259543	9chr17	77389480	77389678	8chr17	79481767	79482012	11chr17	79482271	79482557	7chr17	80225528	80225749	10chr17	8059829	8060036	10chr17	80626507	80626740	9chr17	8191783	8192041	9chr17	8313655	8313909	9chr17	9632444	9632674	10chr18	10560539	10560756	10chr18	12237278	12237511	9chr18	12308685	12308920	12chr18	12883888	12884130	11chr18	21202304	21202445	11chr1	8257812	8258062	12chr18	29228958	29229198	12chr18	29399635	29399837	12chr18	29738056	29738244	9chr18	3153356	3153599	6chr18	32924382	32924588	10chr18	34389623	34389805	8chr18	3603540	3603750	7chr18	46394857	46395091	8chr18	47883628	47883880	9chr18	48738000	48738258	10chr18	55803068	55803300	10chr18	57568085	57568329	7chr18	60087943	60088199	11chr18	7221483	7221704	10chr1	87239965	87240223	10chr18	74110947	74111115	8chr18	74421781	74421989	10chr18	74695267	74695492	10chr18	9516536	9516726	10chr18	9913516	9913800	13chr18	9915028	9915267	9chr19	1026314	1026672	11chr19	10369774	10369927	8chr19	10969039	10969192	10chr19	11052779	11052976	10chr19	1251415	1251596	10chr19	1259273	1259493	11chr19	12871454	12871634	9chr1	91317031	91317244	9chr19	14492060	14492234	11chr19	15666723	15666930	12chr19	1590757	1590988	9chr19	16230443	16230610	10chr19	16441301	16441516	11chr19	1652749	1652935	7chr19	1652889	1653053	11chr19	16825618	16825824	8chr19	16889704	16889883	6chr1	91748357	91748531	11chr19	17559442	17559617	9chr19	17650455	17650690	9chr19	17895264	17895471	9chr19	18344027	18344201	8chr19	1837683	1837884	9chr19	18512603	18512840	9chr19	18668516	18668792	11chr19	2168382	2168569	9chr19	2182974	2183153	11chr19	22034605	22034731	10chr1	9224064	9224250	13chr19	2391352	2391558	9chr19	3065785	3065983	8chr1	9327195	9327416	10chr19	35454905	35455171	11chr19	3665924	3666086	10chr19	36869912	36870172	10chr19	36909424	36909660	9chr19	38183075	38183395	9chr19	3828488	3828696	10chr19	39125382	39125540	10chr19	39220419	39220580	10chr19	39677931	39678127	9chr19	40723289	40723534	11chr19	40939571	40939824	8chr19	4139816	4139996	9chr19	42363713	42363913	9chr19	42439103	42439272	11chr19	4267692	4267874	9chr19	44288640	44288841	10chr19	44961329	44961494	10chr1	94527092	94527248	8chr19	45492549	45492695	10chr19	4633286	4633465	11chr19	46532233	46532401	11chr19	47045981	47046217	8chr19	47120337	47120545	10chr19	47289680	47289978	12chr19	47516292	47516516	10chr19	47730277	47730547	10chr19	48018672	48018946	9chr19	4811795	4811971	9chr19	4872691	4872898	11chr19	48763443	48763654	12chr19	48794535	48794753	11chr19	49895206	49895428	10chr19	506790	506986	11chr19	50860414	50860637	11chr19	51373754	51373973	11chr19	52281173	52281357	9chr19	52800399	52800633	8chr19	52900937	52901230	11chr19	53360767	53360927	11chr19	53898337	53898516	8chr19	54345459	54345732	11chr19	5578301	5578513	9chr19	55996375	55996628	10chr19	56154782	56154973	8chr19	5720171	5720384	9chr19	5828224	5828498	10chr19	58838244	58838502	10chr19	58951585	58951760	11chr19	610790	611042	11chr19	6551995	6552223	10chr19	6947788	6947963	11chr19	8258861	8259087	9chr19	8261512	8261753	12chr20	17549713	17549978	8chr20	21216371	21216536	8chr20	25275164	25275375	9chr20	30222117	30222301	9chr20	30913704	30913887	10chr20	31490334	31490564	9chr20	322413	322641	7chr20	32262173	32262395	10chr20	33110901	33111155	9chr20	34726597	34726800	8chr20	34790823	34791019	8chr20	35490406	35490663	9chr20	35820709	35820965	10chr20	36851182	36851392	11chr20	37897664	37897856	10chr20	388781	389079	12chr20	4018714	4018974	11chr20	42876865	42877039	7chr20	43362079	43362291	10chr20	43514147	43514371	9chr20	45149735	45149930	10chr20	48315881	48316084	9chr20	48795768	48795987	8chr20	48806881	48807147	12chr20	49596814	49597011	9chr20	50278227	50278433	10chr20	50582942	50583144	10chr20	5099986	5100245	9chr20	52509917	52510112	11chr20	52720009	52720256	10chr20	56070342	56070538	9chr20	56347686	56347915	8chr20	59804270	59804464	10chr20	60471423	60471633	8chr20	61436097	61436321	11chr20	61439663	61439905	11chr20	61505116	61505328	10chr20	62184160	62184349	7chr20	62338185	62338396	11chr20	796766	796966	8chr20	8380696	8380949	11chr2	100759004	100759177	8chr2	10975480	10975723	9chr2	113388656	113388897	10chr2	114006841	114007076	10chr21	15399557	15399735	9chr2	11919366	11919561	10chr2	121010719	121010996	11chr2	127955432	127955635	9chr2	127984310	127984561	12chr2	128173481	128173740	9chr2	128785721	128785974	9chr2	131911607	131911874	10chr2	132430349	132430495	9chr2	132559399	132559618	10chr21	34814438	34814669	10chr2	136467540	136467736	10chr21	36880749	36880954	11chr21	37400847	37401029	9chr21	37470216	37470453	11chr21	40169460	40169710	10chr21	40890350	40890512	10chr21	42832601	42832810	10chr2	142943631	142943848	8chr21	43203719	43203929	9chr21	43619595	43619811	8chr21	44582970	44583181	12chr21	46847622	46847891	8chr21	46962261	46962433	10chr21	47744109	47744404	8chr2	15043959	15044160	10chr2	152145883	152146154	12chr2	1554570	1554732	10chr2	161850989	161851176	11chr2	166150281	166150487	9chr2	171181191	171181356	10chr2	173306824	173307033	10chr2	173326812	173327066	10chr2	1754638	1754848	10chr2	175613727	175613929	8chr2	17720195	17720391	10chr2	180871716	180871940	8chr2	182321392	182321657	7chr2	18360101	18360261	8chr2	188406429	188406703	10chr2	191402648	191402824	12chr2	192544964	192545234	7chr2	198318486	198318771	9chr2	200093928	200094133	10chr2	202645584	202645793	7chr2	203696343	203696566	10chr2	204400154	204400480	11chr2	208576162	208576403	9chr2	209040155	209040321	10chr22	18225289	18225502	10chr2	219221387	219221638	8chr22	19466667	19466904	7chr22	20098723	20098984	10chr22	20273365	20273525	10chr2	220324211	220324432	10chr22	21995823	21996033	6chr22	25801262	25801497	11chr22	27061543	27061723	9chr2	227514925	227515214	11chr2	232527076	232527383	11chr2	232580045	232580219	10chr2	233305358	233305527	9chr22	34317078	34317323	11chr2	23574439	23574665	10chr22	36317844	36318115	9chr2	238330265	238330449	9chr2	238600788	238601069	11chr22	38857555	38857736	8chr2	239048207	239048500	10chr2	239142970	239143261	11chr2	239172141	239172382	10chr22	39833271	39833514	10chr22	40420848	40421067	11chr22	40432720	40432964	9chr22	41042304	41042508	11chr22	41107616	41107834	11chr2	241172389	241172571	9chr22	41684326	41684596	9chr2	241790715	241790908	9chr22	41908845	41909122	10chr22	41939819	41940040	10chr22	42833561	42833757	9chr22	43364039	43364257	11chr22	43486246	43486447	9chr22	47169839	47170094	10chr22	50639264	50639530	9chr22	50689578	50689803	9chr22	51221973	51222190	12chr2	25585595	25585804	10chr2	25642571	25642823	5chr2	26578417	26578678	10chr2	27069251	27069426	9chr2	27473107	27473259	10chr2	30559961	30560182	10chr2	31479116	31479333	6chr2	37788882	37789107	8chr2	42096943	42097133	10chr2	54749898	54750119	10chr2	54787498	54787788	10chr2	61921664	61921867	7chr2	62639243	62639483	9chr2	637013	637282	7chr2	64680345	64680607	10chr2	65215451	65215651	8chr2	68612752	68612950	10chr2	7237467	7237741	8chr2	72998092	72998307	9chr2	73439712	73439871	9chr2	74776083	74776323	11chr2	75904495	75904702	8chr2	85109310	85109477	11chr2	85664745	85664952	8chr2	85969649	85969883	13chr2	86501443	86501702	9chr2	87568674	87568927	10chr2	88927044	88927315	9chr2	91757831	91758064	10chr2	95742550	95742760	8chr2	96676182	96676380	8chr2	96814257	96814509	9chr2	97426607	97426875	11chr2	9954054	9954293	9chr3	10050730	10050990	9chr3	102392460	102392673	10chr3	107843235	107843489	11chr3	10987470	10987681	8chr3	112709624	112709891	6chr3	113296735	113296936	11chr3	114958696	114958888	11chr3	11815830	11816069	8chr3	118960398	118960591	10chr3	119187685	119187922	9chr3	123371247	123371538	10chr3	125709258	125709548	8chr3	126627603	126627874	11chr3	128444822	128445014	8chr3	129824939	129825140	10chr3	13053125	13053340	10chr3	130539886	130540096	9chr3	132118227	132118466	11chr3	135794128	135794348	12chr3	137690104	137690318	10chr3	138594727	138594953	9chr3	138634820	138635030	13chr3	138642353	138642569	10chr3	139007410	139007624	13chr3	141089094	141089286	10chr3	14550486	14550660	10chr3	145878072	145878310	8chr3	14589841	14590058	10chr3	149198274	149198471	9chr3	149320238	149320490	11chr3	149526044	149526246	9chr3	150187455	150187726	12chr3	150481298	150481484	7chr3	15190236	15190421	11chr3	152939651	152939859	8chr3	169581276	169581488	10chr3	170626454	170626693	8chr3	170910165	170910402	9chr3	171146145	171146339	10chr3	171814944	171815213	10chr3	172268441	172268622	9chr3	183484882	183485103	10chr3	183613111	183613283	11chr3	186463885	186464120	9chr3	188672443	188672646	11chr3	190231561	190231850	12chr3	194040586	194040871	10chr3	194823451	194823640	8chr3	196246103	196246274	9chr3	20096162	20096393	8chr3	23851807	23852017	10chr3	29322316	29322529	9chr3	33116013	33116242	10chr3	39142671	39142903	11chr3	41309634	41309802	10chr3	42694224	42694500	8chr3	45635583	45635792	9chr3	4575494	4575707	10chr3	45906273	45906543	12chr3	46249820	46250068	11chr3	46759348	46759563	10chr3	46936750	46936936	10chr3	47018429	47018678	9chr3	48700314	48700565	9chr3	49025368	49025539	10chr3	50230133	50230315	9chr3	50362062	50362244	9chr3	50388120	50388364	10chr3	50628326	50628578	10chr3	50654224	50654518	9chr3	52086906	52087130	10chr3	52704932	52705170	12chr3	53168814	53168979	8chr3	57204249	57204451	10chr3	57955605	57955833	12chr3	58554391	58554621	10chr3	71632759	71632947	9chr3	71924633	71924889	10chr3	75834604	75834883	10chr3	81810420	81810678	10chr4	100733010	100733290	11chr4	103370981	103371181	10chr4	103422362	103422573	11chr4	112881400	112881609	12chr4	146729506	146729798	13chr4	154006347	154006616	8chr4	154671379	154671654	9chr4	165877930	165878168	10chr4	170284500	170284719	8chr4	171059884	171060066	9chr4	1731534	1731746	8chr4	175204714	175204979	9chr4	17810188	17810411	8chr4	178302193	178302445	6chr4	1786938	1787146	12chr4	182449798	182449992	10chr4	2221848	2222003	10chr4	25441630	25441835	10chr4	26030412	26030643	10chr4	2789141	2789363	9chr4	37009914	37010084	8chr4	38133975	38134199	10chr4	40228953	40229181	11chr4	4490183	4490401	11chr4	48129842	48130118	9chr4	56212667	56212906	11chr4	6182654	6182896	11chr4	6329058	6329265	9chr4	68193078	68193255	9chr4	70746967	70747171	10chr4	71570196	71570482	11chr4	76649228	76649461	9chr4	78079321	78079545	8chr4	79642278	79642545	11chr4	79696953	79697217	11chr4	81798048	81798263	11chr4	82342665	82342890	10chr4	84551398	84551582	9chr4	84565944	84566105	10chr4	8470585	8470733	9chr4	85425058	85425339	10chr5	1011279	1011493	9chr5	109024409	109024597	7chr5	116283670	116283921	9chr5	1185890	1186115	12chr5	118788022	118788229	11chr5	122847835	122848052	10chr5	123997162	123997410	9chr5	127270562	127270800	10chr5	131400455	131400686	8chr5	131515900	131516094	9chr5	132166274	132166530	8chr5	133922489	133922771	9chr5	134582677	134582878	10chr5	134896587	134896812	10chr5	135418183	135418391	10chr5	137090392	137090655	9chr5	139224066	139224279	9chr5	141081743	141081961	9chr5	141340396	141340596	11chr5	141404402	141404648	9chr5	143569459	143569684	10chr5	143571638	143571847	9chr5	146568453	146568655	9chr5	148608625	148608864	10chr5	149339056	149339271	10chr5	150138208	150138512	9chr5	150618894	150619077	9chr5	1524259	1524509	10chr5	153570236	153570404	7chr5	159200405	159200640	11chr5	160971376	160971564	9chr5	170065811	170065962	9chr5	172141923	172142182	10chr5	172337875	172338234	9chr5	173798638	173798880	11chr5	173846580	173846815	10chr5	174337303	174337470	9chr5	17517694	17517893	11chr5	17519777	17520005	11chr5	17596384	17596606	11chr5	176736778	176736940	9chr5	176797859	176798013	9chr5	177573045	177573288	9chr5	180633759	180633968	11chr5	32028218	32028402	8chr5	32531776	32532030	10chr5	34474667	34474855	7chr5	34898402	34898666	10chr5	373922	374131	8chr5	40798068	40798253	9chr5	44875830	44876032	9chr5	55232022	55232216	11chr5	55497008	55497191	8chr5	56679098	56679330	9chr5	66505021	66505281	9chr5	70363430	70363668	11chr5	71662251	71662453	10chr5	71907044	71907236	9chr5	74862741	74862958	8chr5	7755200	7755383	10chr5	81521439	81521648	11chr5	86417166	86417422	10chr5	89311370	89311641	10chr5	94980070	94980293	9chr5	95672155	95672395	10chr5	96143543	96143797	12chr6	10694758	10695019	9chr6	109103540	109103736	11chr6	109629354	109629595	7chr6	111003654	111003903	10chr6	111200350	111200621	11chr6	11144711	11144868	9chr6	11496510	11496713	10chr6	116589730	116590029	11chr6	116989800	116990028	8chr6	117923758	117923970	10chr6	122702733	122702945	11chr6	12289649	12289865	9chr6	13375668	13375871	8chr6	134159006	134159224	9chr6	137355089	137355293	11chr6	137416835	137417052	12chr6	141804860	141805044	12chr6	1424598	1424815	7chr6	143748431	143748598	9chr6	146055963	146056174	8chr6	14735042	14735326	11chr6	15245555	15245747	11chr6	157505409	157505649	9chr6	161412725	161412991	8chr6	161791935	161792100	10chr6	16215818	16216008	8chr6	16331656	16331851	8chr6	164377718	164377928	9chr6	165341086	165341295	10chr6	169357014	169357279	9chr6	169613060	169613286	11chr6	16965154	16965360	10chr6	170190253	170190554	8chr6	24581434	24581612	9chr6	27661680	27661930	11chr6	28180684	28180859	6chr6	2854008	2854191	9chr6	28918700	28918874	9chr6	2989249	2989457	10chr6	30749676	30749917	10chr6	30778696	30778931	9chr6	31165354	31165649	10chr6	31171761	31171994	9chr6	31519203	31519404	11chr6	32054545	32054737	7chr6	32096010	32096308	9chr6	32143837	32144084	9chr6	32145708	32146023	11chr6	32653428	32653631	8chr6	3324771	3325013	10chr6	33524713	33524956	10chr6	33557500	33557717	9chr6	34954599	34954866	12chr6	35295279	35295486	8chr6	35655602	35655925	11chr6	36562082	36562323	10chr6	37208030	37208307	8chr6	3849432	3849613	10chr6	41673703	41673958	9chr6	41814490	41814756	11chr6	42043355	42043523	10chr6	42847272	42847439	8chr6	49524267	49524463	9chr6	5003750	5004062	11chr6	51824605	51824817	11chr6	6697211	6697427	8chr6	6724869	6725072	9chr6	6918850	6919107	10chr6	71377432	71377596	9chr6	75918695	75918908	9chr6	90995418	90995643	9chr7	100494748	100494975	10chr7	101526233	101526410	9chr7	102045050	102045280	9chr7	102514633	102514825	10chr7	105705433	105705717	10chr7	106377037	106377222	9chr7	112538814	112539017	9chr7	113061816	113062052	10chr7	1177865	1178045	8chr7	12297364	12297547	12chr7	126336794	126336990	10chr7	127087491	127087695	10chr7	128502632	128502960	8chr7	129464736	129465012	8chr7	133977870	133978088	10chr7	134233711	134233907	13chr7	138437366	138437595	9chr7	138666114	138666356	8chr7	141233190	141233377	11chr7	141676098	141676290	9chr7	142153238	142153437	8chr7	142571111	142571306	11chr7	143077322	143077595	9chr7	148892610	148892905	12chr7	149558524	149558718	12chr7	150683181	150683417	11chr7	150691749	150691942	13chr7	151216927	151217115	10chr7	155589689	155589898	8chr7	1573554	1573741	11chr7	1576917	1577149	10chr7	17022352	17022505	10chr7	21657552	21657741	9chr7	21807021	21807240	8chr7	23125680	23125910	9chr7	23508894	23509180	8chr7	23720386	23720576	12chr7	24859905	24860106	12chr7	24865861	24866094	8chr7	25019083	25019293	8chr7	25932570	25932752	10chr7	26101354	26101553	10chr7	27219391	27219622	10chr7	28076944	28077170	10chr7	3105397	3105630	10chr7	33149009	33149260	11chr7	33972561	33972758	9chr7	37241258	37241486	12chr7	38217548	38217717	7chr7	38407855	38408033	9chr7	39559587	39559807	9chr7	43944865	43945125	9chr7	44121928	44122137	11chr7	44512983	44513216	12chr7	45018680	45018866	12chr7	45957279	45957478	9chr7	47859014	47859191	12chr7	48128043	48128259	10chr7	4923371	4923625	8chr7	50310318	50310576	8chr7	50485845	50486023	8chr7	55322588	55322787	8chr7	55602082	55602286	9chr7	56072560	56072783	11chr7	6048460	6048750	8chr7	66919058	66919286	10chr7	67090298	67090451	10chr7	70294179	70294424	10chr7	72425217	72425419	12chr7	73265903	73266102	7chr7	73476587	73476817	8chr7	73498696	73499038	7chr7	73864342	73864558	8chr7	73866701	73866945	9chr7	75649510	75649694	9chr7	75698571	75698784	12chr7	76876613	76876801	9chr7	81314315	81314539	8chr7	82201581	82201853	9chr7	88176120	88176332	11chr7	90338203	90338374	10chr7	92533707	92533945	9chr7	95064332	95064620	8chr7	95704789	95705016	10chr7	96044341	96044498	8chr7	96656985	96657224	10chr7	97557634	97557838	11chr7	97870778	97870980	7chr7	98638092	98638314	9chr7	98977754	98978040	12chr8	101506740	101506984	6chr8	101920825	101921009	10chr8	102388337	102388553	11chr8	103546882	103547132	7chr8	103664052	103664341	10chr8	103666059	103666303	10chr8	104033248	104033443	8chr8	118316437	118316644	10chr8	119974204	119974394	9chr8	121820886	121821106	9chr8	124084661	124084922	11chr8	124553410	124553651	6chr8	125452692	125452862	9chr8	125642031	125642268	9chr8	126283687	126283900	9chr8	126676745	126676951	9chr8	126711060	126711295	12chr8	13134084	13134334	11chr8	134221528	134221805	10chr8	134309346	134309514	11chr8	139739744	139739956	9chr8	139779965	139780174	10chr8	141646167	141646360	12chr8	142046232	142046409	11chr8	142164209	142164477	11chr8	142183376	142183604	9chr8	142412692	142413022	9chr8	143716103	143716290	9chr8	144110718	144110962	10chr8	144461886	144462073	11chr8	145692282	145692549	8chr8	145800890	145801098	8chr8	146228177	146228390	8chr8	17352672	17352905	11chr8	17557167	17557351	11chr8	20233798	20234000	10chr8	22562219	22562441	9chr8	23081226	23081481	9chr8	23405337	23405549	13chr8	24076295	24076518	10chr8	24749341	24749605	10chr8	2481000	2481211	10chr8	28277129	28277301	10chr8	28602645	28602826	10chr8	29208399	29208640	11chr8	30015091	30015365	10chr8	33370558	33370796	10chr8	37709027	37709212	10chr8	37853881	37854094	10chr8	38385661	38385901	10chr8	41692317	41692473	9chr8	42243019	42243221	8chr8	42645481	42645669	10chr8	48227842	48228080	11chr8	48909870	48910081	7chr8	49834463	49834657	7chr8	61824335	61824620	8chr8	72917412	72917715	12chr8	81904184	81904423	9chr8	8800159	8800383	10chr8	94834644	94834821	10chr8	97340176	97340445	9chr9	100000571	100000771	10chr9	100955321	100955596	8chr9	100968049	100968272	11chr9	101892380	101892639	9chr9	103173972	103174184	9chr9	112181132	112181325	11chr9	112232047	112232211	10chr9	114749137	114749338	9chr9	114790943	114791230	11chr9	114936865	114937124	9chr9	116169308	116169502	10chr9	117053326	117053553	10chr9	117111260	117111426	11chr9	118353908	118354075	9chr9	120480436	120480671	10chr9	125164771	125164939	9chr9	126695750	126696023	11chr9	128005657	128005831	10chr9	128508503	128508693	8chr9	129253680	129253845	10chr9	130732735	130732901	12chr9	132319766	132319978	12chr9	133587941	133588226	10chr9	133933275	133933507	9chr9	134466607	134466817	7chr9	136859608	136859791	9chr9	137979293	137979499	12chr9	139010708	139010937	9chr9	139237310	139237510	7chr9	139944343	139944515	10chr9	16192185	16192420	10chr9	178941	179195	12chr9	19174256	19174499	11chr9	32572266	32572580	10chr9	34377058	34377303	12chr9	34633947	34634174	10chr9	34646459	34646745	9chr9	35732165	35732346	11chr9	36008829	36009048	10chr9	37954134	37954358	10chr9	43133615	43133876	11chr9	5886159	5886405	10chr9	6055319	6055505	11chr9	68403283	68403451	11chr9	73028827	73029101	10chr9	74895931	74896097	11chr9	75091950	75092183	9chr9	75093135	75093385	10chr9	75172545	75172748	10chr9	75201743	75201975	7chr9	76484272	76484455	9chr9	91003424	91003665	9chr9	92217982	92218189	8chr9	94712475	94712710	9chr9	95719491	95719737	10chr9	95860931	95861225	9chrX	100352982	100353301	9chrX	106361972	106362208	8chrX	109656424	109656628	11chrX	114877335	114877499	8chrX	11551361	11551513	8chrX	117973343	117973631	8chrX	128674133	128674335	8chrX	128906899	128907190	12chrX	1331475	1331685	13chrX	13506396	13506587	10chrX	149736750	149736977	10chrX	152094855	152095148	9chrX	152933420	152933632	8chrX	153192271	153192519	9chrX	154027419	154027662	12chrX	154285707	154285920	8chrX	15693042	15693373	6chrX	16910979	16911178	9chrX	192776	193109	10chrX	197889	198112	7chrX	39968270	39968574	9chrX	46184884	46185178	11chrX	46838082	46838293	10chrX	47342554	47342784	10chrX	48755346	48755516	10chrX	49047353	49047680	10chrX	49605384	49605599	8chrX	53100805	53101078	9chrX	65382226	65382478	10chrX	69674316	69674484	9chr10	100220605	100220889	8chr10	101380983	101381311	10chr10	101769536	101769794	10chr10	102430852	102431009	8chr10	103127371	103127598	8chr10	105358617	105358841	8chr10	106089097	106089319	9chr10	1102157	1102488	8chr10	112307005	112307203	10chr10	113897224	113897393	8chr10	114139796	114140059	10chr10	1156082	1156299	9chr10	118706736	118706892	8chr10	120183395	120183614	8chr10	120969068	120969255	11chr10	121030595	121030772	8chr10	121276085	121276324	10chr10	122708486	122708715	10chr10	123902396	123902628	10chr10	124027662	124027854	9chr10	124058380	124058539	9chr10	124913553	124913752	9chr10	125755361	125755661	10chr10	126315566	126315849	8chr10	12698643	12698794	9chr10	129809675	129809832	9chr10	134257197	134257385	6chr10	13702087	13702257	9chr10	17299702	17299968	10chr10	1758026	1758282	8chr10	22919622	22919854	12chr10	24755269	24755483	7chr10	25186368	25186547	7chr10	29420973	29421227	10chr10	29922282	29922488	7chr10	30440965	30441206	12chr10	3894654	3894840	10chr10	46962574	46962778	10chr10	4857810	4858009	9chr10	50189476	50189666	8chr10	50615715	50615876	9chr10	51371297	51371494	10chr10	52191609	52191842	11chr10	5708446	5708713	9chr10	6056206	6056417	10chr10	6094651	6094843	6chr10	6150984	6151150	10chr10	61645221	61645467	10chr10	64028400	64028626	8chr10	6622295	6622510	9chr10	69834932	69835164	10chr10	72685551	72685710	9chr10	73734294	73734524	9chr10	73893388	73893613	12chr10	73930946	73931152	11chr10	74447999	74448194	10chr10	78307169	78307393	10chr10	78933673	78933890	9chr10	79110512	79110706	10chr10	80417449	80417601	8chr10	80911573	80911795	12chr10	81539167	81539360	9chr10	88726365	88726573	8chr10	91847044	91847282	12chr10	92766102	92766344	10chr10	93311684	93311909	8chr10	95218618	95218840	9chr10	97153917	97154160	9chr10	97291870	97292041	11chr10	97360967	97361177	10chr10	98004223	98004426	10chr10	98797188	98797415	9chr10	99313343	99313549	9chr1	101664899	101665144	9chr1	10567583	10567838	9chr1	1057566	1057747	10chr11	102158776	102158980	11chr1	110648825	110649004	6chr1	110750457	110750643	7chr11	108568661	108568887	10chr11	109810007	109810172	9chr11	110044990	110045189	9chr1	111038675	111038875	9chr1	11123072	11123294	12chr11	11643550	11643870	8chr11	118042353	118042590	10chr11	118272061	118272228	9chr11	118479238	118479480	10chr11	118936200	118936410	8chr11	119995725	119995973	9chr11	120796777	120796953	11chr1	112298069	112298228	9chr11	12310233	12310394	10chr11	125011635	125011875	10chr11	128392415	128392710	9chr11	128500328	128500577	10chr11	128718811	128718986	8chr11	128729838	128729989	9chr11	129201799	129201959	10chr11	129862374	129862571	7chr11	133920422	133920635	8chr11	134095250	134095615	10chr11	14322323	14322515	9chr11	1567907	1568156	7chr1	116518719	116518956	9chr11	16967569	16967819	8chr1	11702544	11702723	9chr1	117279932	117280153	9chr1	117604629	117604857	10chr1	117635901	117636103	8chr11	18137100	18137318	8chr11	19098611	19098863	9chr11	20022109	20022385	7chr11	2011157	2011417	10chr11	20132527	20132693	9chr1	12184944	12185143	9chr1	12212218	12212496	10chr11	26671421	26671606	9chr1	1280238	1280458	11chr11	29303804	29303977	9chr11	29315417	29315669	10chr11	3079952	3080198	10chr11	32395872	32396085	8chr11	33722411	33722672	9chr11	33963170	33963409	9chr11	35051559	35051810	11chr11	35099282	35099491	8chr1	1440904	1441131	12chr1	145427522	145427702	10chr11	45676882	45677051	8chr1	147071345	147071659	9chr1	147362359	147362585	7chr11	47433777	47433988	8chr1	149194465	149194726	9chr1	149678709	149678959	10chr1	150436912	150437136	9chr1	15105681	15105971	9chr1	151964880	151965140	11chr1	153467495	153467649	8chr1	154469544	154469798	9chr1	154779622	154779838	9chr1	155294977	155295163	8chr1	156355440	156355691	7chr1	156474909	156475116	12chr1	156786504	156786802	10chr11	57016576	57016758	8chr11	57117392	57117557	8chr1	15744080	15744283	11chr1	158057201	158057436	8chr11	58555935	58556161	9chr11	60598596	60598773	10chr1	160708934	160709107	10chr11	60737841	60738052	9chr11	61245190	61245397	9chr11	62308665	62308983	12chr1	162583563	162583797	7chr11	62676678	62676985	8chr11	62791624	62791842	7chr11	63342010	63342206	9chr11	63684590	63684771	7chr11	63913597	63913806	8chr11	64546472	64546657	7chr11	6460270	6460467	7chr11	64774385	64774588	9chr11	64949177	64949348	12chr11	65192787	65192986	10chr1	165227275	165227489	7chr11	65473848	65474111	10chr11	6589821	6589996	10chr11	66156582	66156724	7chr11	66197534	66197715	8chr11	66246480	66246708	10chr11	66821422	66821600	8chr11	67415685	67415892	9chr11	67976709	67976933	8chr11	68606670	68606889	8chr1	16905623	16905832	10chr1	16911919	16912128	10chr1	170043595	170043823	8chr11	70245886	70246189	10chr11	71291229	71291426	8chr11	71498456	71498677	8chr11	72296826	72297018	9chr1	172368693	172368876	9chr1	173991599	173991762	11chr11	74855585	74855738	8chr11	74991287	74991481	9chr1	175288808	175289004	9chr11	76445045	76445229	9chr1	176528983	176529248	9chr1	176918248	176918441	8chr1	179779041	179779220	9chr11	805295	805545	10chr1	183958345	183958533	11chr1	185308444	185308644	10chr11	85894164	85894411	6chr1	187460611	187460825	8chr1	18923967	18924196	8chr1	192528721	192528927	9chr1	193027612	193027876	11chr11	93275104	93275286	10chr11	94802937	94803131	9chr11	94887702	94887961	8chr1	19567787	19568032	10chr1	200772084	200772278	9chr1	201968784	201969032	8chr1	202777524	202777708	9chr1	203651913	203652186	9chr1	204288681	204288886	12chr1	204400543	204400704	10chr1	204415610	204415799	10chr1	206753341	206753619	10chr1	206757076	206757253	10chr1	207108885	207109057	11chr1	207510533	207510743	8chr1	207793709	207793939	9chr1	209739466	209739689	7chr1	209824577	209824857	9chr1	209942800	209943072	10chr1	210033725	210033915	7chr12	100548617	100548827	7chr12	101381822	101382042	9chr12	102167464	102167654	11chr12	103899220	103899503	8chr12	104856736	104856998	11chr12	105065614	105065829	9chr12	105324478	105324731	9chr12	105777158	105777446	9chr12	106617954	106618174	9chr12	110243130	110243347	8chr1	21112989	21113182	9chr12	113376050	113376298	9chr12	113623046	113623367	7chr1	211909138	211909379	12chr12	120877526	120877709	9chr12	121932082	121932246	8chr12	122227798	122228023	7chr12	122429185	122429450	9chr12	122607813	122608006	6chr12	124018402	124018598	8chr12	124196687	124196891	11chr12	125179515	125179753	10chr12	125721731	125721912	7chr12	126257698	126257893	6chr12	12627924	12628139	11chr12	12717016	12717230	9chr12	127526666	127526877	8chr12	127610446	127610761	10chr12	131589257	131589537	9chr12	15421936	15422164	7chr12	1940270	1940497	8chr1	221608803	221609064	10chr1	22231509	22231752	10chr1	222807831	222808084	10chr1	222988281	222988505	10chr1	22352338	22352593	8chr1	223888662	223888883	10chr1	223903067	223903286	8chr12	2445605	2445777	9chr1	224752939	224753172	10chr12	24992320	24992521	10chr12	25144546	25144708	9chr12	25207765	25207996	10chr12	25589292	25589519	9chr1	226251977	226252224	10chr1	226821885	226822080	10chr1	226862767	226863081	10chr12	27396923	27397184	9chr1	22747001	22747257	7chr1	228800243	228800421	11chr12	2922821	2923031	8chr1	22968591	22968764	8chr1	230446412	230446579	11chr12	31477066	31477277	8chr12	3384719	3384864	7chr1	234836002	234836189	11chr1	23515445	23515712	7chr1	236267005	236267238	10chr1	23912162	23912376	9chr1	240061502	240061673	9chr1	241803523	241803742	10chr1	242011078	242011244	9chr1	243417660	243417846	10chr1	244517121	244517344	10chr1	244815903	244816169	11chr1	244978916	244979121	10chr12	45524690	45524876	8chr12	45564319	45564557	9chr12	45628330	45628510	7chr12	46765115	46765310	11chr1	24717804	24718079	8chr12	47479602	47479865	9chr1	24842133	24842383	8chr12	48512891	48513154	8chr12	49381884	49382127	8chr12	49457753	49457950	10chr12	49524032	49524216	11chr12	50433115	50433268	8chr12	50569318	50569514	7chr12	50618249	50618412	9chr1	2509760	2510038	10chr12	51610938	51611141	9chr12	51781963	51782176	10chr12	51818887	51819087	10chr12	52637900	52638122	9chr1	25293210	25293415	8chr12	53259162	53259381	7chr1	25357338	25357582	10chr1	25360554	25360770	11chr12	53773612	53773824	10chr12	54137493	54137676	9chr1	25428027	25428168	10chr12	54730291	54730495	8chr12	54973577	54973770	8chr12	54997079	54997243	9chr12	551524	551701	10chr12	55466873	55467141	10chr12	57984848	57985089	9chr1	26123410	26123577	8chr12	6389268	6389490	8chr12	6454623	6454796	9chr1	27326070	27326276	8chr12	76328337	76328574	11chr1	27744884	27745106	9chr12	77456746	77457004	9chr1	27816977	27817235	7chr12	8123309	8123549	11chr12	8384370	8384596	9chr1	28678211	28678387	10chr12	89936758	89937046	10chr12	90320987	90321186	12chr12	92528695	92528957	7chr12	9800658	9800939	8chr12	9860831	9861080	8chr12	99037812	99038012	8chr13	101347341	101347575	9chr13	103248527	103248823	9chr13	103426158	103426337	11chr13	114035453	114035659	8chr13	114830791	114831094	10chr1	31229523	31229783	9chr1	31956319	31956518	11chr13	20138984	20139212	10chr13	21347679	21348016	11chr13	28713470	28713658	11chr13	33769488	33769747	10chr13	33854058	33854283	9chr1	33924190	33924475	10chr1	33938132	33938363	7chr1	3400448	3400697	7chr13	40690246	40690451	9chr13	40976576	40976837	9chr13	41885084	41885370	10chr13	43632531	43632768	10chr13	44833300	44833485	10chr13	46381753	46381920	6chr1	3481895	3482092	7chr13	52701917	52702161	9chr1	35667772	35667981	8chr1	36689530	36689785	10chr13	67804602	67804754	9chr13	74282600	74282765	9chr13	76270056	76270250	8chr1	3773838	3774060	9chr13	79768503	79768750	11chr1	38493710	38493972	5chr13	92058931	92059197	10chr1	39283814	39284132	7chr1	39624333	39624567	10chr13	96486506	96486704	9chr13	97912269	97912512	10chr1	40254680	40254920	12chr1	40311362	40311576	10chr1	40870515	40870746	10chr14	100497916	100498117	10chr14	100874887	100875101	8chr14	101317571	101317768	9chr14	102276042	102276288	8chr14	103230616	103230839	10chr14	103243764	103243974	8chr14	103796307	103796552	8chr14	104028861	104029057	8chr14	105189974	105190221	7chr14	105669375	105669586	9chr14	106993650	106993820	9chr1	42217015	42217198	7chr14	22942066	22942224	11chr1	42335862	42336037	9chr14	23849183	23849406	7chr14	24299433	24299588	10chr14	24766890	24767076	8chr14	24777354	24777563	7chr14	32728974	32729161	8chr1	43282849	43283006	12chr14	35825272	35825613	10chr14	35860260	35860474	9chr14	36539751	36539945	9chr1	43692934	43693191	8chr1	43703817	43704065	9chr14	39336800	39337027	10chr1	43988844	43989065	9chr1	43994896	43995057	10chr1	44300850	44301056	9chr1	44501582	44501862	11chr14	50529359	50529576	7chr1	45194968	45195158	7chr14	52083783	52084022	8chr1	45480191	45480468	11chr14	54863408	54863649	11chr14	54955700	54955974	10chr14	55368862	55369101	9chr1	46035806	46036019	8chr14	65290044	65290197	8chr1	46872014	46872273	9chr14	69260060	69260260	9chr1	4712741	4712929	9chr14	72322172	72322442	9chr14	74129219	74129454	9chr14	74684402	74684627	8chr14	74926346	74926527	9chr14	75760832	75761024	10chr14	76178236	76178469	8chr14	77371384	77371565	9chr14	78328619	78328799	6chr14	81630546	81630769	9chr14	91696160	91696372	12chr14	91708319	91708502	11chr14	92724886	92725098	8chr14	93418337	93418544	8chr14	93842174	93842409	8chr14	95908929	95909098	8chr15	101547266	101547457	9chr15	20398349	20398502	10chr15	22893559	22893788	7chr15	31047354	31047533	10chr15	32270087	32270362	8chr15	34354192	34354410	8chr15	35838117	35838333	9chr1	53647753	53648015	9chr15	38982975	38983214	9chr15	39917041	39917225	10chr15	40571673	40571856	9chr15	41139620	41139806	9chr15	41165844	41166073	8chr15	41551445	41551623	8chr15	42873642	42873911	7chr15	43542016	43542242	10chr15	43577797	43578070	9chr15	45021043	45021247	11chr15	45749830	45750005	10chr15	45997907	45998184	8chr15	50736901	50737104	11chr1	55137779	55137987	10chr15	51455134	51455355	9chr15	51484936	51485187	9chr15	52861235	52861465	9chr15	53027753	53027923	10chr15	55700620	55700822	8chr15	55818786	55818973	7chr15	57852136	57852288	8chr15	58474795	58474994	9chr15	60663675	60663833	9chr15	60700695	60700901	9chr15	60800106	60800292	9chr15	62126395	62126584	7chr15	64359814	64359991	9chr15	65023577	65023789	8chr15	65102162	65102370	9chr15	65133645	65133946	8chr15	65184437	65184620	11chr15	65611219	65611416	9chr15	65930983	65931217	10chr15	67749339	67749548	8chr15	69335976	69336157	8chr15	70563821	70564036	8chr15	70780454	70780645	9chr15	73992675	73992882	10chr15	74200674	74200893	9chr15	74667645	74667808	11chr15	74677446	74677765	11chr15	75061247	75061427	9chr15	75748423	75748668	7chr15	76474010	76474281	10chr15	77316736	77316900	9chr15	78202865	78203072	10chr15	80325110	80325343	10chr15	81288694	81288878	8chr15	81558252	81558478	8chr15	81773004	81773194	8chr15	82728046	82728265	10chr15	82752574	82752754	9chr15	82987086	82987263	9chr15	83011723	83011922	10chr15	83104571	83104790	10chr15	83129160	83129340	9chr15	83209150	83209356	7chr15	83349209	83349399	7chr15	83501353	83501632	9chr15	83679684	83679860	11chr15	84910818	84911039	10chr15	85861713	85861974	10chr15	88998310	88998547	9chr15	89421998	89422187	7chr15	90581547	90581784	11chr15	90776534	90776722	10chr1	59118353	59118566	10chr15	91191427	91191621	10chr15	91208802	91209023	11chr15	91576411	91576591	9chr15	92396387	92396580	9chr15	92620602	92620816	7chr15	93160828	93161049	12chr15	98985701	98985890	9chr15	99640833	99641023	9chr16	1156335	1156541	8chr16	11891175	11891328	9chr16	11904002	11904285	7chr16	1380625	1380804	9chr16	14595446	14595682	9chr16	15915807	15915999	9chr16	15982483	15982744	8chr16	163508	163731	12chr16	17510530	17510800	10chr16	19125230	19125466	10chr16	20311865	20312073	11chr1	6217843	6218092	10chr16	2186003	2186250	12chr16	2190698	2190879	10chr16	22332664	22332844	8chr16	23446785	23447002	10chr16	23690784	23691010	10chr16	24510619	24510777	10chr16	24516143	24516327	10chr16	28437583	28437869	9chr16	28505472	28505658	7chr16	28699751	28700036	10chr16	28761534	28761785	8chr16	28884929	28885203	8chr16	29168442	29168627	8chr16	29652976	29653233	10chr16	29703351	29703533	9chr16	29721691	29721920	9chr16	3019165	3019459	8chr16	30411527	30411881	8chr16	30461176	30461344	10chr16	30547012	30547242	9chr16	3068141	3068425	9chr16	30705255	30705479	10chr16	3124976	3125218	12chr16	31345063	31345311	10chr16	31891850	31892137	9chr16	3221921	3222179	7chr16	402975	403242	11chr16	4311631	4311854	6chr16	4409168	4409377	7chr1	6453767	6453985	10chr16	47538621	47538799	9chr16	48222428	48222700	9chr16	50699352	50699517	8chr16	53132907	53133088	9chr16	53544407	53544689	7chr1	6535605	6535824	10chr16	56611063	56611200	8chr16	57079766	57080000	9chr16	57338162	57338391	7chr16	57625136	57625447	10chr16	57634380	57634586	8chr16	60888	61104	10chr16	66929188	66929361	9chr16	67138117	67138330	7chr16	67278316	67278535	9chr16	68176271	68176465	10chr16	686266	686485	10chr16	69511083	69511342	11chr16	69597971	69598229	7chr16	69830440	69830692	9chr16	70429379	70429567	7chr16	74729837	74730038	9chr16	75339449	75339661	9chr16	80500384	80500562	9chr16	81439430	81439650	11chr1	68231857	68232095	9chr16	84652509	84652790	9chr16	84985888	84986049	7chr16	85330653	85330893	9chr16	85461905	85462109	8chr16	85641003	85641181	11chr16	85722460	85722596	7chr16	87840445	87840674	9chr16	87843169	87843412	10chr16	88814371	88814576	7chr16	88848926	88849095	9chr16	88991680	88991901	9chr16	89181692	89181957	8chr16	90148440	90148637	10chr17	1026352	1026555	7chr17	1162513	1162721	7chr17	12159803	12159999	10chr17	1268808	1269008	9chr17	1316636	1316866	11chr17	15903016	15903228	7chr17	16319872	16320124	7chr17	16905164	16905348	9chr17	17723064	17723243	8chr17	17822601	17822810	9chr17	18864183	18864421	8chr17	20058925	20059216	10chr17	21187782	21188057	7chr17	235959	236171	8chr17	25926944	25927135	11chr17	2628095	2628262	11chr17	26898456	26898688	8chr17	27444221	27444417	8chr17	28420528	28420759	8chr17	28667236	28667500	9chr17	33325307	33325464	9chr17	33581450	33581659	11chr17	34296619	34296809	9chr17	34819826	34820073	8chr17	35405005	35405219	11chr17	3767037	3767244	9chr17	37810176	37810349	6chr17	3793383	3793618	9chr17	38256870	38257183	12chr17	3838429	3838618	9chr17	38470919	38471137	10chr17	3877605	3877819	8chr17	39835421	39835659	11chr17	40219368	40219640	7chr17	40268825	40268991	11chr17	40540745	40540901	9chr17	41446492	41446736	12chr17	41869086	41869293	9chr17	42428198	42428455	8chr17	42852938	42853173	6chr17	43309079	43309340	10chr17	43662445	43662612	6chr17	43862384	43862589	8chr17	44368640	44368926	11chr17	44899759	44899941	9chr17	45393066	45393270	8chr17	4624867	4625116	9chr17	47392966	47393142	9chr17	47772894	47773129	8chr17	4890757	4890939	11chr17	48929520	48929695	9chr17	49010944	49011169	11chr17	5095008	5095270	10chr17	5100335	5100497	10chr17	55082469	55082697	10chr17	55292773	55292972	9chr17	57078854	57079068	12chr17	60498850	60499017	10chr17	61538229	61538470	7chr17	62833224	62833529	8chr17	63290394	63290595	10chr17	64271829	64271988	9chr17	64948206	64948444	8chr17	655510	655650	8chr17	65646985	65647162	11chr17	65780538	65780695	10chr17	66770496	66770673	11chr17	6955426	6955644	10chr17	70419063	70419287	8chr17	71187691	71187942	10chr17	71301242	71301451	10chr17	7155222	7155475	7chr1	7729574	7729810	8chr17	7297913	7298162	10chr17	74497352	74497509	10chr17	74637704	74637886	8chr17	75052424	75052586	9chr17	75441528	75441737	6chr1	7764598	7764776	6chr17	78056518	78056759	9chr17	78623873	78624135	9chr17	78638838	78639090	10chr17	79827276	79827458	10chr1	77999479	77999646	9chr17	80297063	80297249	8chr17	80415910	80416171	9chr17	80843804	80844014	12chr17	8204016	8204201	9chr1	8075120	8075308	8chr18	13577361	13577546	10chr1	8157555	8157828	9chr18	21599682	21599861	8chr18	23476191	23476418	8chr18	28810597	28810848	9chr18	29265125	29265366	11chr18	3013347	3013662	10chr18	32440560	32440776	11chr18	33058579	33058832	9chr18	35024781	35024995	10chr18	35358550	35358797	8chr18	3995576	3995790	8chr18	42419220	42419380	10chr18	42792805	42792994	9chr1	84326198	84326387	10chr18	43460011	43460198	8chr18	45299119	45299270	9chr18	45550145	45550401	9chr18	46459467	46459703	6chr18	46543578	46543786	9chr18	47037086	47037324	9chr18	51673905	51674102	12chr18	55513559	55513783	9chr18	56219320	56219509	9chr1	85744636	85744837	9chr18	57620325	57620575	8chr18	57655417	57655616	10chr1	85794697	85794942	9chr18	6004783	6004953	10chr18	60382414	60382647	9chr18	61106595	61106791	10chr18	68159031	68159254	10chr18	71863709	71863883	10chr18	74514079	74514276	7chr1	87490957	87491156	11chr18	77905676	77905878	10chr18	77960227	77960366	8chr1	87800876	87801182	10chr18	812763	813011	8chr1	88419560	88419744	9chr18	8730497	8730689	10chr1	8933740	8933963	7chr1	90355773	90356024	11chr19	10522971	10523251	10chr19	10538856	10539093	11chr19	1064967	1065159	9chr19	10947014	10947272	10chr19	1205636	1205843	6chr19	12074745	12074965	11chr19	12098347	12098580	9chr19	1275414	1275716	9chr19	12787203	12787430	10chr19	12903343	12903599	9chr19	13278584	13278765	8chr19	13283243	13283444	9chr19	13306612	13306793	8chr19	13700134	13700325	10chr19	13972643	13972898	10chr19	14247925	14248120	9chr19	14258965	14259187	9chr19	14399707	14399904	9chr19	14897094	14897371	9chr19	15667303	15667479	9chr19	15695536	15695725	9chr19	16189639	16189841	9chr19	16293609	16293821	9chr19	16933978	16934147	10chr19	17190883	17191125	9chr19	17577101	17577297	10chr19	17684982	17685164	7chr19	1775014	1775215	8chr19	18228574	18228749	10chr19	18402423	18402636	11chr19	18521487	18521651	8chr19	18547821	18548104	10chr19	1855408	1855686	9chr19	18561813	18561979	10chr19	19471973	19472171	9chr19	19976516	19976776	9chr1	9202384	9202565	9chr19	20748499	20748807	8chr19	21105916	21106195	9chr19	21205935	21206166	8chr19	2156086	2156292	11chr19	2263733	2263936	11chr19	23257945	23258137	8chr19	2507206	2507422	10chr19	30185602	30185767	9chr1	9310114	9310343	9chr1	93249707	93249915	9chr19	33726210	33726462	10chr19	33897848	33898008	8chr19	33921630	33921842	8chr19	34262558	34262733	10chr1	93441214	93441403	10chr19	3485085	3485254	8chr19	35417725	35418010	8chr19	35738684	35738979	11chr19	36236242	36236494	8chr19	39348030	39348280	10chr19	39900718	39901010	10chr19	40324176	40324443	11chr19	4065910	4066139	11chr19	40697205	40697396	9chr19	41770048	41770293	9chr19	41873779	41873993	9chr19	42289572	42289808	10chr19	42461989	42462172	11chr19	42538984	42539166	8chr19	42772787	42773050	9chr19	42905533	42905690	8chr19	4455249	4455468	11chr19	45160558	45160800	7chr19	45348882	45349047	8chr19	45690849	45691037	9chr19	45946135	45946335	11chr19	46273517	46273733	8chr19	46520165	46520325	9chr19	47260632	47260838	8chr19	47343759	47344018	9chr19	47714057	47714233	7chr19	4809690	4809874	10chr19	48269257	48269465	10chr19	48673641	48673863	11chr19	48993062	48993304	9chr19	49148106	49148319	11chr19	49395235	49395428	10chr19	49649168	49649394	9chr19	507294	507560	8chr1	95113853	95114074	9chr19	52692993	52693277	7chr19	53439683	53439853	11chr19	53696446	53696671	9chr19	53928355	53928512	9chr19	54249575	54249803	9chr1	954916	955181	9chr19	55128419	55128650	10chr19	55690639	55690875	11chr19	5794165	5794470	10chr19	57988901	57989130	9chr19	59092597	59092810	11chr19	6460589	6460823	8chr19	662169	662396	10chr19	6737322	6737479	6chr19	6772627	6772830	8chr19	7616308	7616534	11chr19	770333	770558	9chr19	7953315	7953531	10chr19	8151074	8151241	10chr19	8477840	8478126	7chr19	951577	951803	11chr20	10982457	10982725	9chr20	12905889	12906098	8chr20	17806883	17807041	11chr20	18257460	18257649	7chr20	19643103	19643335	9chr20	20693457	20693699	8chr20	20805677	20805873	10chr20	23133096	23133252	11chr20	30249451	30249670	10chr20	31377931	31378138	8chr20	32890732	32890994	11chr20	33917459	33917651	8chr20	34673705	34673986	10chr20	35093963	35094149	11chr20	36685973	36686196	11chr20	36722793	36722974	11chr20	3767319	3767542	10chr20	39574674	39574888	10chr20	3994620	3994875	10chr20	43948185	43948432	8chr20	44844610	44844804	11chr20	45181735	45181938	7chr20	45834348	45834634	8chr20	4587947	4588136	9chr20	46354146	46354390	8chr20	47113449	47113718	10chr20	47895052	47895273	9chr20	48291318	48291517	9chr20	49175203	49175423	9chr20	52565914	52566115	9chr20	5510345	5510535	9chr20	56008052	56008238	10chr20	5626151	5626361	12chr20	57056614	57056871	9chr20	58071082	58071235	8chr20	61940952	61941137	8chr20	62212208	62212411	8chr20	62359758	62360006	10chr20	719739	720009	9chr20	741682	741873	8chr2	101601408	101601600	7chr2	101767965	101768235	10chr2	10590177	10590364	11chr2	10638039	10638259	10chr2	106731832	106732009	9chr2	110267783	110267995	10chr2	11270035	11270336	9chr2	113403428	113403612	9chr2	113463891	113464098	11chr2	113507274	113507538	10chr2	113578868	113579122	10chr2	113961589	113961781	8chr2	114195263	114195474	9chr2	11483818	11484120	10chr2	11586874	11587048	6chr2	11618146	11618317	9chr2	121453284	121453452	8chr2	121852936	121853149	7chr2	12434071	12434298	12chr21	25341032	25341234	7chr2	129513056	129513269	8chr21	30117450	30117621	8chr21	30118099	30118275	10chr21	33158294	33158460	8chr21	33238470	33238691	10chr21	34602818	34603095	9chr2	134644280	134644548	9chr21	34681265	34681528	9chr21	35402488	35402688	11chr21	35720269	35720490	9chr21	35756353	35756547	11chr21	35815909	35816106	8chr21	36143908	36144089	7chr2	136555156	136555376	8chr21	36772801	36773017	9chr21	37528710	37528984	10chr21	37670531	37670725	11chr21	38593525	38593757	10chr21	39073059	39073236	11chr21	39591052	39591240	11chr21	39728008	39728193	10chr21	40685677	40685888	10chr21	41160334	41160571	8chr21	43678721	43678954	10chr21	44775823	44776021	10chr21	45079199	45079422	9chr21	46572291	46572495	8chr21	47016708	47016871	9chr21	47390629	47390846	8chr21	47567407	47567597	7chr21	47714781	47715076	10chr2	148601958	148602223	10chr2	149571350	149571542	9chr2	152144799	152145001	9chr2	158159087	158159313	10chr2	16938543	16938778	9chr2	169658953	169659218	8chr2	171943356	171943561	8chr2	173330526	173330705	8chr2	174888859	174889139	7chr2	175442792	175443035	10chr2	175636381	175636629	8chr2	178848027	178848227	10chr2	17935105	17935371	9chr2	180871989	180872198	10chr2	182321925	182322174	11chr2	190007918	190008158	8chr2	192109873	192110108	9chr2	192731773	192731994	10chr2	197107102	197107319	9chr2	198570013	198570231	9chr2	198669164	198669388	11chr21	9968440	9968735	9chr2	200604601	200604790	8chr2	201270735	201270958	8chr2	201676142	201676463	10chr2	201827925	201828185	8chr2	202645835	202646065	10chr2	203453438	203453648	9chr2	203776267	203776491	9chr2	20525936	20526187	7chr2	206750378	206750602	9chr2	208489308	208489580	9chr2	208610870	208611037	9chr2	211036176	211036352	9chr2	213727943	213728220	11chr2	214018451	214018690	10chr2	214092226	214092438	9chr2	217639870	217640118	9chr22	19384661	19384858	11chr22	19435102	19435353	9chr22	19617511	19617695	10chr22	19949840	19950039	8chr22	20012990	20013301	12chr2	220341041	220341298	8chr22	21132042	21132264	11chr22	21265344	21265535	9chr22	21369048	21369319	8chr22	21986946	21987158	10chr22	22697308	22697481	9chr22	23053289	23053487	9chr2	224761565	224761736	11chr22	24899170	24899413	10chr22	24917757	24918001	8chr2	225266733	225266983	8chr22	25758175	25758353	9chr22	28200735	28200938	8chr22	30234309	30234547	7chr22	30602981	30603187	8chr22	30608652	30608846	7chr22	30787189	30787398	8chr22	30881444	30881651	10chr2	231729525	231729838	9chr22	32056981	32057217	9chr2	232466406	232466623	9chr22	33056273	33056541	9chr2	233470604	233470792	9chr2	233470891	233471096	9chr22	33695647	33695847	12chr2	233924917	233925140	9chr2	234352224	234352472	8chr2	235197688	235197902	9chr2	236402373	236402641	9chr22	37562631	37562848	10chr22	37853455	37853614	7chr22	38749237	38749411	8chr22	39096869	39097113	11chr2	239892241	239892471	9chr2	240189171	240189401	9chr22	40306189	40306389	12chr22	41929279	41929497	9chr2	242314096	242314290	12chr22	42665613	42665838	9chr22	44389107	44389308	9chr22	46001203	46001456	9chr22	46481693	46481930	9chr22	50323258	50323453	8chr22	50648343	50648520	9chr2	26800744	26800971	9chr2	27165556	27165757	8chr2	27239740	27239961	10chr2	28619669	28619924	9chr2	31456802	31457079	10chr2	38059820	38060001	7chr2	38374049	38374261	7chr2	38834930	38835141	11chr2	39355233	39355487	12chr2	39719320	39719547	11chr2	42068006	42068253	8chr2	42316534	42316726	8chr2	42890356	42890528	9chr2	43056014	43056225	7chr2	44105114	44105298	9chr2	46456731	46456959	10chr2	46769401	46769696	10chr2	47403602	47403771	9chr2	47562273	47562494	8chr2	47615529	47615697	10chr2	47629915	47630170	10chr2	48348007	48348262	8chr2	48571397	48571635	9chr2	54308800	54309049	10chr2	54816799	54817004	11chr2	55278357	55278567	9chr2	58269775	58269990	7chr2	60666632	60666828	10chr2	60901776	60901943	9chr2	6485770	6485977	9chr2	7057319	7057510	8chr2	74055901	74056129	10chr2	75280703	75280913	10chr2	80162946	80163156	8chr2	85147029	85147268	9chr2	86161322	86161518	10chr2	86477244	86477455	11chr2	86790118	86790471	8chr2	88301418	88301595	9chr2	96079825	96080015	8chr2	96808058	96808290	8chr2	96840309	96840539	10chr2	97466763	97466993	10chr2	9893392	9893626	11chr3	100789160	100789379	10chr3	101232007	101232279	9chr3	111830756	111830902	10chr3	113006979	113007179	9chr3	120499866	120500117	10chr3	121468630	121468938	9chr3	122233677	122233858	9chr3	124620281	124620420	10chr3	124826947	124827131	7chr3	125231021	125231264	9chr3	125853109	125853271	9chr3	125917161	125917358	9chr3	127263128	127263333	8chr3	127309831	127310118	10chr3	127814087	127814286	9chr3	127945716	127945920	8chr3	128134180	128134413	11chr3	128513931	128514165	9chr3	128943943	128944128	9chr3	13025807	13026020	8chr3	133142483	133142673	9chr3	133293276	133293577	9chr3	13394292	13394485	8chr3	134204456	134204735	10chr3	137771123	137771302	8chr3	140661415	140661679	8chr3	141173856	141174074	10chr3	14167272	14167560	10chr3	142804015	142804224	10chr3	142877496	142877791	7chr3	148897911	148898116	9chr3	148963544	148963778	8chr3	150505665	150505820	11chr3	150583204	150583376	7chr3	152192493	152192688	6chr3	152760934	152761138	9chr3	15310979	15311214	10chr3	15352223	15352438	10chr3	155761760	155761979	8chr3	157324481	157324699	10chr3	15900451	15900643	9chr3	16101860	16102113	9chr3	169939728	169940026	9chr3	171860197	171860390	9chr3	176656887	176657078	10chr3	181418280	181418496	10chr3	183959676	183959883	9chr3	184007231	184007503	10chr3	185276821	185277037	10chr3	185972598	185972869	12chr3	186759633	186759885	8chr3	190284300	190284491	11chr3	192185078	192185297	7chr3	195749017	195749234	9chr3	196006253	196006487	7chr3	197226392	197226624	8chr3	197381672	197381938	9chr3	197520207	197520508	8chr3	23727323	23727596	11chr3	25634057	25634302	7chr3	27722788	27722958	8chr3	32461886	32462104	10chr3	33414384	33414607	9chr3	37254608	37254812	9chr3	37821106	37821319	10chr3	38008886	38009120	10chr3	39041556	39041722	9chr3	39072415	39072570	9chr3	42220993	42221212	10chr3	4462901	4463102	7chr3	44753983	44754170	10chr3	45082278	45082465	6chr3	45224737	45225023	8chr3	46580592	46580851	11chr3	4763817	4764048	11chr3	48885036	48885365	9chr3	49208553	49208728	8chr3	4930949	4931107	9chr3	49876721	49876913	9chr3	50388425	50388623	11chr3	5058338	5058510	11chr3	51534190	51534426	9chr3	53148034	53148238	9chr3	56952366	56952532	9chr3	61880781	61881010	10chr3	63329383	63329662	9chr3	6759166	6759343	11chr3	69788366	69788625	11chr3	77087657	77087880	10chr3	81810117	81810354	7chr3	81959479	81959689	8chr3	8486112	8486314	12chr3	9394933	9395128	9chr4	100644799	100645047	8chr4	100729495	100729688	10chr4	10159452	10159667	9chr4	1124463	1124614	9chr4	112563327	112563556	9chr4	114315964	114316193	6chr4	144257134	144257422	8chr4	152038588	152038770	8chr4	15480689	15480950	9chr4	156810663	156810844	9chr4	159131490	159131718	10chr4	15957975	15958138	10chr4	159748026	159748292	11chr4	164415521	164415791	9chr4	169075099	169075253	9chr4	17338339	17338577	9chr4	174291849	174292143	11chr4	175041668	175041894	8chr4	17638519	17638664	10chr4	184183663	184183859	9chr4	185235406	185235623	12chr4	185570769	185571024	10chr4	185667011	185667260	9chr4	190714238	190714442	9chr4	2757722	2757951	9chr4	27693782	27694010	9chr4	3049177	3049403	10chr4	37803781	37803966	9chr4	38063759	38063925	7chr4	39033937	39034139	11chr4	3944229	3944407	10chr4	39625900	39626181	12chr4	40533384	40533617	9chr4	40843072	40843278	9chr4	40899711	40899890	9chr4	41694917	41695136	9chr4	52762045	52762244	9chr4	6690699	6690938	9chr4	6875767	6875957	7chr4	72978377	72978542	9chr4	74401765	74401970	7chr4	75585090	75585331	9chr4	76498665	76498949	11chr4	76769480	76769675	7chr4	77008361	77008588	8chr4	77155556	77155804	8chr4	77507121	77507350	11chr4	78508092	78508357	12chr4	80274470	80274588	10chr4	86478476	86478688	10chr4	86647048	86647310	9chr4	9705133	9705377	9chr5	10595962	10596169	9chr5	10962296	10962456	6chr5	112299528	112299814	9chr5	112714753	112714906	9chr5	113785633	113785866	9chr5	118406335	118406601	7chr5	118676334	118676518	6chr5	118703258	118703497	11chr5	120609861	120610096	7chr5	126409027	126409248	10chr5	133801540	133801751	6chr5	133984228	133984492	12chr5	135547928	135548118	10chr5	139145289	139145515	12chr5	13988063	13988262	9chr5	142035348	142035512	8chr5	14352297	14352456	10chr5	145234299	145234543	11chr5	148494317	148494515	9chr5	149370396	149370635	11chr5	149509602	149509777	7chr5	149737030	149737330	11chr5	150494950	150495116	11chr5	151204985	151205278	10chr5	154029747	154030009	9chr5	154062397	154062650	9chr5	1568552	1568702	7chr5	157502327	157502573	8chr5	158560202	158560401	9chr5	158606120	158606313	10chr5	162887129	162887391	9chr5	169611126	169611378	9chr5	169862102	169862283	7chr5	17118849	17119017	9chr5	172197632	172197818	9chr5	172295602	172295793	11chr5	173019153	173019378	10chr5	17413467	17413689	7chr5	17523189	17523395	11chr5	175815532	175815707	9chr5	17584371	17584563	11chr5	176037171	176037384	8chr5	176498948	176499146	9chr5	176543944	176544145	9chr5	176988359	176988544	11chr5	179228859	179229097	9chr5	179755729	179755910	7chr5	179898052	179898264	10chr5	30040691	30040939	10chr5	31809240	31809421	9chr5	32093364	32093602	9chr5	34523311	34523532	9chr5	35617806	35617992	7chr5	37723023	37723263	10chr5	38383052	38383270	7chr5	38441937	38442138	10chr5	39077069	39077323	10chr5	39949079	39949303	7chr5	49963498	49963833	10chr5	54038266	54038520	10chr5	55507425	55507634	9chr5	56328099	56328314	10chr5	56433990	56434225	8chr5	64777626	64777837	8chr5	6484021	6484222	8chr5	65038473	65038698	9chr5	65220982	65221166	10chr5	67639298	67639577	7chr5	73927476	73927719	12chr5	73944654	73944928	7chr5	76028915	76029116	12chr5	78320881	78321066	6chr5	7878710	7878926	12chr5	80461403	80461628	10chr5	87976303	87976503	9chr5	88123076	88123278	11chr5	90568825	90568997	10chr5	90675994	90676242	8chr5	95144526	95144766	10chr5	96270888	96271187	11chr5	97173987	97174233	8chr5	98368213	98368453	8chr6	106340826	106341061	9chr6	106545714	106545874	9chr6	106583101	106583331	8chr6	106967557	106967830	9chr6	10747684	10747920	8chr6	107639395	107639596	8chr6	107780592	107780787	10chr6	108887119	108887371	9chr6	111799992	111800217	10chr6	113953562	113953831	10chr6	114180115	114180357	9chr6	116422194	116422496	9chr6	119121761	119121959	8chr6	119450578	119450860	9chr6	119582539	119582701	9chr6	12008761	12008920	8chr6	133144220	133144416	9chr6	13360982	13361180	8chr6	136871637	136871902	8chr6	136904257	136904457	8chr6	137289349	137289512	8chr6	138428535	138428755	12chr6	142310513	142310730	10chr6	1437016	1437207	9chr6	144472443	144472700	8chr6	151996934	151997107	9chr6	1524305	1524463	8chr6	155545274	155545490	11chr6	15662613	15662818	8chr6	156983187	156983377	9chr6	157309444	157309761	8chr6	157738266	157738449	8chr6	158182430	158182606	7chr6	158402561	158402843	11chr6	158939620	158939852	10chr6	164006941	164007217	9chr6	16410041	16410199	8chr6	166689245	166689448	8chr6	167631916	167632125	7chr6	167764900	167765142	10chr6	17993790	17993951	9chr6	20127058	20127247	8chr6	21022637	21022901	8chr6	21264209	21264440	8chr6	2246538	2246756	8chr6	24856321	24856496	9chr6	25138424	25138702	9chr6	27198221	27198409	10chr6	27280516	27280751	9chr6	27447364	27447518	9chr6	27862916	27863189	7chr6	28652066	28652240	9chr6	3056292	3056496	10chr6	3118776	3119116	10chr6	3160524	3160738	9chr6	31702588	31702744	9chr6	3209530	3209808	10chr6	33640701	33640887	10chr6	34241152	34241318	11chr6	35420253	35420428	8chr6	38288710	38288906	10chr6	39082615	39082827	8chr6	39277768	39277973	9chr6	39315672	39315876	10chr6	4008662	4008894	8chr6	40276470	40276750	11chr6	4156150	4156349	9chr6	44030646	44030798	9chr6	44201907	44202150	9chr6	44306316	44306512	9chr6	45888606	45888868	11chr6	4688313	4688553	8chr6	52027777	52027993	10chr6	52151694	52151924	9chr6	52516947	52517088	10chr6	5303583	5303765	8chr6	6803910	6804127	9chr6	71276534	71276695	8chr6	73332815	73333048	8chr6	74225457	74225673	10chr6	76206040	76206314	12chr6	7975337	7975594	10chr6	80577924	80578133	8chr6	83073239	83073421	8chr6	86192908	86193096	10chr6	87646783	87647073	10chr6	88632679	88632875	5chr6	90081868	90082033	10chr7	100026827	100026995	8chr7	100042130	100042293	6chr7	100224277	100224479	9chr7	100399885	100400112	12chr7	100817249	100817447	9chr7	101625304	101625450	6chr7	101922857	101923062	9chr7	102179813	102180026	9chr7	102278921	102279134	9chr7	105229986	105230164	9chr7	106511584	106511774	12chr7	111381877	111382117	9chr7	112190605	112190771	10chr7	112579879	112580147	10chr7	11372232	11372411	9chr7	119912306	119912492	9chr7	120446060	120446219	9chr7	121184938	121185116	9chr7	123197799	123197942	9chr7	1265200	1265409	7chr7	127775735	127775947	8chr7	128027795	128027995	9chr7	128574613	128574834	8chr7	129882466	129882656	9chr7	130645137	130645420	11chr7	130920504	130920763	9chr7	131630247	131630382	9chr7	132003616	132003770	8chr7	133826094	133826320	9chr7	134197987	134198193	7chr7	135357614	135357829	9chr7	137567022	137567245	9chr7	139365623	139365838	7chr7	139555358	139555598	9chr7	140143845	140144053	8chr7	141349772	141349970	10chr7	142115704	142115918	9chr7	149580590	149580767	7chr7	150685657	150685873	8chr7	151201061	151201301	9chr7	154585941	154586201	9chr7	1560355	1560604	10chr7	20256981	20257179	9chr7	20369374	20369584	9chr7	22390480	22390674	12chr7	22484207	22484442	10chr7	2249230	2249421	10chr7	2353939	2354217	8chr7	23584620	23584818	10chr7	25138129	25138312	9chr7	2996419	2996665	6chr7	30780167	30780323	8chr7	31162284	31162558	9chr7	362115	362314	9chr7	36331180	36331345	9chr7	37026810	37026987	9chr7	372666	372842	11chr7	40938869	40939042	9chr7	43479468	43479658	8chr7	43966230	43966478	11chr7	44025561	44025742	9chr7	44111962	44112208	8chr7	45013429	45013647	9chr7	45921634	45921823	8chr7	46017849	46018050	9chr7	47140512	47140682	8chr7	50132166	50132370	10chr7	50306624	50306810	9chr7	50894529	50894742	8chr7	51228822	51229011	10chr7	5436835	5437039	9chr7	5467640	5467901	9chr7	55141743	55142058	10chr7	5593053	5593287	9chr7	6425009	6425279	9chr7	64254770	64255034	11chr7	6488090	6488255	11chr7	75892340	75892520	8chr7	76067291	76067451	11chr7	7758975	7759167	8chr7	7805170	7805364	9chr7	7835402	7835681	10chr7	8174058	8174256	8chr7	8335768	8335951	10chr7	86099068	86099257	9chr7	87872694	87872911	10chr7	90446143	90446350	8chr7	92262789	92262982	10chr7	92264275	92264513	9chr7	92463248	92463500	9chr7	93717096	93717328	10chr7	95950636	95950882	9chr7	97464903	97465157	9chr7	97642819	97643063	9chr7	97762562	97762780	11chr7	98709719	98709987	10chr7	98963226	98963461	9chr8	100872127	100872267	10chr8	101733062	101733267	8chr8	101962458	101962749	9chr8	101965583	101965794	9chr8	10282704	10282938	8chr8	103669564	103669767	9chr8	10538534	10538689	10chr8	107670608	107670807	9chr8	107868814	107868999	9chr8	11325093	11325327	9chr8	116231208	116231486	10chr8	11816414	11816627	9chr8	124054017	124054234	8chr8	124428601	124428838	11chr8	124642671	124642882	8chr8	12624235	12624480	8chr8	130832423	130832664	8chr8	131141908	131142085	9chr8	13224319	13224495	10chr8	134511578	134511791	8chr8	134581957	134582209	9chr8	134898393	134898597	9chr8	142045540	142045785	10chr8	144371666	144371922	10chr8	144635678	144635861	11chr8	145150942	145151169	10chr8	17623934	17624087	10chr8	1840614	1840881	8chr8	20898709	20898909	9chr8	22530900	22531139	7chr8	26315443	26315668	9chr8	27472186	27472378	9chr8	27918668	27918893	8chr8	29083246	29083464	9chr8	29386991	29387254	10chr8	3142158	3142349	11chr8	36716515	36716658	10chr8	37595179	37595441	9chr8	39891583	39891809	10chr8	427582	427785	10chr8	49231377	49231573	8chr8	52438712	52438919	11chr8	54798523	54798778	8chr8	56015404	56015701	10chr8	56793100	56793367	8chr8	580949	581159	9chr8	58909608	58909796	10chr8	59634273	59634499	9chr8	59754677	59754865	10chr8	60964177	60964388	9chr8	65294696	65294901	8chr8	6673854	6674042	9chr8	67579341	67579548	7chr8	68492371	68492591	10chr8	7212755	7212976	10chr8	72751290	72751468	4chr8	72756039	72756309	10chr8	74268649	74268872	7chr8	74332222	74332418	9chr8	77912177	77912371	9chr8	81595232	81595494	9chr8	81913743	81913982	9chr8	8229356	8229609	10chr8	82692841	82692995	7chr8	8608306	8608515	7chr8	95834965	95835140	8chr8	95961668	95961863	7chr8	96249446	96249652	9chr8	96261034	96261196	10chr8	99370411	99370575	9chr8	99956370	99956571	11chr9	100686020	100686265	10chr9	100716914	100717095	9chr9	100836765	100836941	8chr9	103364636	103364939	8chr9	109975840	109976081	10chr9	114061671	114061862	8chr9	114783051	114783285	8chr9	114837095	114837349	11chr9	116354016	116354185	9chr9	116417707	116417995	8chr9	117167928	117168096	10chr9	117247272	117247545	11chr9	117415883	117416062	9chr9	12329445	12329610	10chr9	123748102	123748364	10chr9	126692279	126692532	9chr9	126973051	126973311	9chr9	127016623	127016810	11chr9	129373944	129374136	8chr9	129702954	129703174	9chr9	130665842	130666100	11chr9	131636848	131637042	8chr9	131690273	131690473	7chr9	132949528	132949694	11chr9	134510738	134511016	8chr9	134637954	134638104	8chr9	135128028	135128262	10chr9	135674484	135674722	10chr9	135916404	135916602	9chr9	135991113	135991299	10chr9	136647364	136647534	11chr9	136964332	136964569	10chr9	137670756	137670986	9chr9	138366854	138367100	9chr9	139233761	139233949	8chr9	139267070	139267310	8chr9	139538917	139539135	6chr9	139894706	139894945	11chr9	14693266	14693556	11chr9	2012420	2012614	12chr9	21813006	21813281	8chr9	27333250	27333479	10chr9	2857626	2857834	11chr9	31120027	31120227	10chr9	32378593	32378771	10chr9	33447012	33447241	9chr9	33624039	33624238	9chr9	34158435	34158718	9chr9	3441283	3441479	7chr9	34664380	34664549	8chr9	3467723	3467883	9chr9	3526287	3526591	8chr9	35477335	35477485	7chr9	36572710	36572962	8chr9	37594547	37594717	8chr9	38035889	38036137	8chr9	69989517	69989647	10chr9	71735607	71735795	9chr9	73013090	73013353	10chr9	74639975	74640197	7chr9	75637932	75638192	9chr9	78990126	78990316	10chr9	85949241	85949417	9chr9	86159348	86159557	8chr9	86237829	86238027	10chr9	86571557	86571755	9chr9	88896927	88897172	8chr9	893881	894142	8chr9	90788410	90788584	8chr9	92208849	92209045	8chr9	97431219	97431385	8chr9	97768611	97768840	9chr9	99616792	99616967	10chrX	100249507	100249704	8chrX	100306759	100306975	9chrX	102942852	102943113	10chrX	106961364	106961623	8chrX	108956330	108956529	10chrX	119115735	119115930	9chrX	119347601	119347825	8chrX	12789668	12789841	8chrX	129243124	129243332	9chrX	13119899	13120112	10chrX	133594168	133594432	9chrX	135967058	135967270	10chrX	13665866	13666116	11chrX	150921911	150922097	7chrX	152773061	152773235	9chrX	153189623	153189814	9chrX	153389416	153389631	10chrX	153977071	153977249	8chrX	15489194	15489435	10chrX	16790032	16790280	4chrX	22544511	22544726	9chrX	2309988	2310240	8chrX	23799558	23799738	11chrX	23925704	23925939	9chrX	23926137	23926347	7chrX	29573664	29573914	11chrX	39055913	39056072	6chrX	45684001	45684254	8chrX	48432706	48432931	8chrX	49028861	49029118	9chrX	49643750	49644069	9chrX	53449064	53449315	8chrX	54208832	54209052	9chrX	71840425	71840650	9chrX	73235307	73235512	10chrX	74376041	74376232	6chrX	74493737	74494005	9chrX	77320507	77320706	10chrX	9645331	9645543	6chrX	99891692	99891936	9chrY	10037714	10037916	11chr10	102632064	102632292	10chr10	102672563	102672779	6chr10	102801078	102801233	7chr10	102819807	102819978	9chr10	103329973	103330215	7chr10	104261844	104262025	8chr10	104956966	104957298	8chr10	105236142	105236455	7chr10	111612266	111612509	8chr10	112201555	112201854	7chr10	112506200	112506428	9chr10	115323293	115323498	7chr10	118927705	118927942	8chr10	120966329	120966506	8chr10	121204956	121205194	8chr10	122475	122683	9chr10	124067536	124067717	8chr10	133977020	133977253	9chr10	134272122	134272294	8chr10	13578845	13579019	7chr10	15251949	15252128	10chr10	16885307	16885550	9chr10	17553408	17553585	9chr10	18273065	18273342	7chr10	22177962	22178177	10chr10	22972193	22972486	9chr10	27847517	27847725	8chr10	29737964	29738174	8chr10	29785379	29785570	7chr10	30876441	30876754	9chr10	30971446	30971618	9chr10	30976674	30976885	9chr10	33422973	33423224	9chr10	35924518	35924732	9chr10	3847452	3847676	6chr10	471065	471244	9chr10	50372685	50372916	9chr10	52337552	52337765	11chr10	5426040	5426231	9chr10	5671891	5672065	9chr10	5701883	5702044	8chr10	60082287	60082508	10chr10	62174589	62174884	8chr10	63656809	63656967	9chr10	64004843	64005034	9chr10	70231608	70231822	7chr10	70848738	70848918	9chr10	70884675	70884822	8chr10	71075398	71075592	9chr10	71275505	71275654	7chr10	71404395	71404574	7chr10	71455872	71456076	7chr10	71867030	71867325	8chr10	72056335	72056591	8chr10	72663121	72663330	8chr10	7306526	7306723	8chr10	73254928	73255168	8chr10	73369033	73369230	8chr10	73596012	73596210	8chr10	73857563	73857791	8chr10	74008508	74008751	9chr10	74020276	74020499	8chr10	75289217	75289443	10chr10	75654308	75654545	9chr10	75668821	75669022	9chr10	76498867	76499159	10chr10	76990445	76990616	8chr10	80724799	80725084	11chr10	81004744	81004997	7chr10	81247412	81247668	7chr10	81838920	81839147	6chr10	81923484	81923798	9chr10	82423884	82424081	7chr10	86016898	86017051	8chr10	86134749	86134991	8chr10	86475708	86475901	8chr10	88648755	88648989	9chr10	89825444	89825719	11chr10	90694678	90694846	7chr10	92079333	92079570	8chr10	92671424	92671634	9chr10	94002957	94003278	8chr10	96163426	96163648	9chr10	99167025	99167257	10chr10	99599939	99600156	11chr1	100065326	100065507	9chr1	101823022	101823214	9chr1	101836848	101837067	9chr1	107538665	107538931	9chr1	107973325	107973529	8chr1	108479160	108479371	10chr1	109104407	109104606	9chr1	10927236	10927413	7chr1	109740050	109740277	11chr11	102323677	102323887	9chr11	102424693	102424960	9chr11	102470440	102470674	8chr11	102906783	102907005	9chr11	10344534	10344811	7chr1	110448890	110449078	11chr1	110596563	110596716	8chr11	10678966	10679242	7chr11	109870866	109871043	8chr1	111003010	111003158	10chr11	111101171	111101422	7chr11	112151341	112151526	7chr11	116562439	116562670	7chr11	117015826	117016085	6chr11	117680085	117680266	9chr11	117688490	117688708	9chr11	117714782	117714967	9chr11	11809411	11809588	8chr11	118766436	118766719	9chr11	118783074	118783253	8chr11	118971029	118971213	8chr11	119600012	119600240	6chr11	120177343	120177589	8chr11	12087857	12088001	8chr11	12188690	12188876	7chr11	122430564	122430749	7chr11	122925078	122925283	8chr1	112312244	112312437	6chr11	123132282	123132466	6chr11	123451367	123451565	8chr11	124272324	124272533	5chr11	124539086	124539284	9chr11	125274778	125275024	9chr11	125811993	125812260	6chr11	12621081	12621303	10chr11	130717592	130717789	8chr11	131564802	131564976	8chr11	131737690	131737843	11chr11	1320414	1320633	10chr11	133797614	133797771	7chr1	113446674	113446959	10chr11	14665252	14665427	7chr1	11538794	11539057	8chr11	15962817	15963009	9chr11	15990999	15991174	10chr11	1650095	1650294	8chr1	117610522	117610679	9chr11	17791636	17791807	9chr11	1903752	1903945	9chr11	1974828	1975039	9chr11	19764332	19764504	9chr11	19973171	19973384	10chr11	20149848	20150050	7chr1	120204888	120205088	10chr1	12039886	12040173	6chr1	12040203	12040434	10chr1	12128531	12128758	4chr1	12178742	12178933	8chr1	12203701	12203971	9chr11	268803	268997	10chr1	12719067	12719267	8chr11	305010	305216	9chr11	32073763	32073968	9chr11	32113254	32113448	9chr11	32334513	32334744	5chr11	33934357	33934588	10chr11	34066571	34066778	8chr11	34607831	34608049	7chr11	35929708	35929934	8chr11	35949356	35949563	9chr11	364455	364697	8chr11	36724286	36724506	8chr11	37525701	37525898	8chr11	3915522	3915728	10chr11	41330815	41331047	10chr1	14135156	14135441	11chr1	143467619	143467774	9chr11	44581003	44581251	8chr1	144621439	144621710	9chr1	145299689	145299945	10chr1	145421672	145421899	8chr1	146082677	146082845	8chr11	46134612	46134816	8chr11	46270242	46270536	11chr11	4647506	4647693	7chr1	146714650	146714879	10chr1	147112647	147112792	8chr11	47198952	47199149	7chr1	14748692	14748894	11chr11	47638649	47638837	7chr11	47664251	47664467	8chr1	147727194	147727442	9chr11	47883444	47883618	8chr11	48028304	48028496	8chr1	148176290	148176465	10chr1	148531981	148532164	9chr1	149224471	149224673	10chr1	150577286	150577446	11chr11	5145371	5145657	9chr1	151803279	151803498	9chr1	153325036	153325170	6chr1	153581373	153581567	8chr11	5402041	5402235	7chr1	154158635	154158863	10chr1	154379913	154380118	8chr1	154392116	154392283	10chr1	155149214	155149352	7chr1	155294102	155294297	9chr1	156265305	156265580	9chr1	156352829	156353029	8chr1	15650143	15650344	7chr1	156561325	156561511	6chr1	1565763	1565916	8chr1	156675867	156676103	8chr1	156897620	156897819	8chr11	57247867	57248058	9chr1	157475558	157475734	8chr11	58335456	58335681	9chr11	58345446	58345625	9chr1	15850586	15850768	7chr11	59544028	59544216	7chr1	159768722	159768966	8chr11	59961209	59961358	8chr11	60145912	60146140	8chr1	160221827	160221999	6chr1	161039632	161039838	7chr1	161041737	161041906	7chr1	161155110	161155296	9chr11	61250095	61250268	7chr1	161275867	161276090	9chr11	61284467	61284632	7chr1	161369107	161369337	9chr1	161591411	161591620	10chr11	62148326	62148497	8chr11	62323976	62324246	10chr1	162824135	162824368	6chr11	63275605	63275749	8chr11	63606166	63606369	9chr11	63688637	63688827	7chr11	63862011	63862146	9chr11	63916166	63916415	8chr1	16402601	16402819	11chr11	64038463	64038770	8chr1	16405477	16405646	9chr1	16408922	16409095	8chr11	64692012	64692229	8chr11	64781049	64781235	8chr11	65356264	65356412	9chr1	165601148	165601367	7chr11	66717569	66717850	8chr11	66839259	66839435	8chr11	67141692	67142044	10chr11	67169089	67169303	9chr11	67171232	67171435	10chr11	67231481	67231670	11chr1	167525448	167525651	7chr1	167657233	167657401	8chr1	168290326	168290540	9chr1	168515287	168515539	7chr1	168606742	168606903	7chr1	168679238	168679495	7chr11	69259200	69259429	8chr1	169281537	169281747	6chr11	6947502	6947673	7chr11	70023471	70023681	7chr11	70952410	70952601	10chr11	72680986	72681308	8chr1	172697232	172697455	7chr11	74160448	74160640	8chr1	174768732	174768973	9chr1	174933706	174933913	10chr1	175121270	175121425	9chr11	7555249	7555499	7chr1	17662658	17662841	8chr11	7678598	7678867	7chr11	76838255	76838439	7chr11	76899469	76899658	7chr1	177150918	177151121	7chr1	17802349	17802587	9chr1	178994734	178994940	9chr11	79204655	79204872	9chr1	179783986	179784124	8chr1	17988138	17988339	8chr11	8027986	8028169	9chr1	180991098	180991346	9chr1	181905904	181906154	7chr1	181999030	181999310	7chr11	8247216	8247439	8chr11	82719042	82719272	9chr1	183573427	183573727	10chr1	183925833	183926035	9chr1	185287067	185287247	7chr1	185702906	185703177	7chr11	85780868	85781052	9chr11	87836910	87837129	8chr11	88831143	88831367	9chr1	19254300	19254575	10chr11	92788143	92788338	10chr1	192955305	192955497	10chr1	19670959	19671126	7chr1	198526134	198526406	10chr11	9883775	9883951	9chr1	200638834	200639020	10chr1	200941186	200941335	8chr1	201458389	201458680	9chr1	201499979	201500140	7chr1	201682633	201682840	9chr1	202104423	202104578	6chr1	203185390	203185600	6chr1	203238269	203238439	8chr1	203274589	203274768	8chr1	203481988	203482209	8chr1	204098217	204098446	7chr1	20427517	20427669	8chr1	204534949	204535190	7chr1	204944872	204945084	7chr1	205030777	205030985	8chr1	205457291	205457551	7chr1	206718220	206718359	7chr1	206939796	206939978	9chr1	207970746	207970922	9chr1	209761238	209761450	8chr12	102333641	102333878	9chr12	10364802	10365040	5chr12	104235715	104235991	9chr12	104271775	104271996	9chr12	104997921	104998181	10chr12	108130770	108131013	10chr12	108297465	108297716	10chr12	108727973	108728238	8chr12	108754326	108754521	8chr12	111040073	111040249	7chr12	111332268	111332513	7chr12	111742475	111742614	9chr12	111967330	111967517	9chr12	113338201	113338446	9chr12	11639577	11639780	8chr12	117141091	117141299	8chr12	118025556	118025741	9chr12	118815747	118815972	8chr12	119417681	119417833	8chr12	11951825	11952107	8chr12	119553189	119553361	10chr12	121397744	121397973	8chr12	121990635	121990823	8chr12	122230137	122230335	8chr12	124296513	124296743	7chr12	12480791	12481009	10chr12	125110931	125111113	8chr12	125235390	125235684	8chr1	212541418	212541588	8chr12	125815562	125815716	7chr12	127392594	127392798	10chr12	12849018	12849299	10chr12	12992964	12993157	8chr12	133464255	133464492	9chr12	15743366	15743531	7chr12	15782110	15782344	7chr1	21651235	21651495	8chr1	21655910	21656145	6chr1	2187305	2187474	10chr1	21965187	21965473	8chr1	221886544	221886724	10chr1	222013234	222013429	8chr1	222060582	222060785	7chr1	222990957	222991180	8chr1	223705169	223705375	7chr1	225654872	225655046	10chr1	226298289	226298528	10chr1	226384798	226384999	9chr1	227100412	227100639	9chr1	227422751	227422987	9chr1	227505989	227506207	7chr12	27536992	27537176	7chr1	227915754	227915963	9chr12	28343213	28343419	8chr1	228966927	228967123	8chr1	229038859	229039096	10chr1	229363261	229363465	8chr1	229393821	229394026	9chr12	298364	298545	7chr1	229850847	229851034	9chr1	230202682	230202945	8chr1	230220463	230220628	8chr1	230303171	230303384	9chr1	230844449	230844669	8chr1	230933690	230933897	7chr12	31424873	31425059	10chr12	32173942	32174181	11chr1	23290746	23290960	8chr12	33741102	33741361	8chr1	2345857	2346056	8chr1	235147554	235147782	9chr1	236073993	236074201	8chr1	236695062	236695297	8chr1	240637426	240637616	8chr12	42417998	42418241	9chr12	42693839	42694042	10chr1	244482968	244483191	8chr1	244763264	244763460	8chr1	244766814	244766999	8chr12	45586193	45586413	8chr12	45626754	45627021	8chr1	246166520	246166709	9chr1	246187976	246188292	10chr12	46502572	46502804	10chr12	46751329	46751586	9chr12	47609297	47609477	8chr12	48109147	48109358	7chr12	48169329	48169522	7chr12	48222781	48223008	9chr12	49371775	49371975	7chr12	49658518	49658814	10chr12	49716977	49717213	7chr12	49725450	49725662	8chr12	49752528	49752717	8chr1	25010227	25010445	7chr12	50172004	50172194	9chr12	50983657	50983914	11chr12	51180715	51180912	8chr12	51219172	51219376	7chr12	51663405	51663568	6chr12	52515653	52515888	9chr12	53301013	53301215	10chr12	53440587	53440878	8chr12	53645858	53646061	9chr12	53886847	53887051	9chr12	53937367	53937504	9chr12	54069599	54069851	7chr12	54788149	54788346	9chr12	54812919	54813122	8chr12	56349953	56350134	7chr12	56600334	56600581	8chr1	25664890	25665047	9chr12	57505476	57505717	6chr12	57518121	57518330	7chr12	57608150	57608349	7chr12	57636830	57636991	8chr12	57846757	57846938	8chr12	57916500	57916765	7chr1	25935174	25935323	7chr12	59507633	59507878	8chr12	59543891	59544093	7chr12	6147327	6147539	8chr1	26202122	26202349	7chr1	26232866	26233081	9chr12	63814024	63814238	10chr12	64311824	64311999	8chr1	26431534	26431724	8chr12	65196081	65196271	8chr12	66025431	66025660	8chr12	6807079	6807259	7chr12	70082897	70083116	10chr1	27204510	27204695	9chr12	7342025	7342234	7chr12	7372352	7372570	9chr12	76160582	76160815	7chr12	76864444	76864645	8chr12	8043644	8043922	8chr12	83044481	83044676	8chr1	28526566	28526702	6chr12	85862515	85862733	9chr12	8797607	8797819	7chr1	28827583	28827857	11chr1	28995062	28995298	10chr12	90250285	90250516	9chr12	93484042	93484224	9chr12	93771341	93771595	10chr12	94179081	94179254	7chr12	96094238	96094476	8chr12	99092750	99092926	8chr1	30265965	30266147	8chr13	101167164	101167356	9chr13	101326076	101326293	10chr13	107185431	107185640	10chr13	109814608	109814813	10chr13	110443690	110443943	8chr13	111868690	111868893	8chr13	113344012	113344212	8chr13	113929733	113929953	9chr1	31252487	31252682	8chr13	20392472	20392679	8chr1	32071517	32071742	10chr1	3229773	3229970	10chr13	25302239	25302390	8chr13	27504519	27504735	6chr13	28051413	28051687	8chr13	32738902	32739103	9chr13	33802786	33802951	8chr13	34116512	34116730	6chr13	36992205	36992378	8chr13	41582562	41582730	8chr13	41859043	41859268	6chr13	42490794	42490992	9chr13	42986952	42987157	7chr13	49154403	49154636	10chr13	50569771	50569990	6chr13	50798724	50798921	8chr13	52123267	52123515	9chr1	35252169	35252325	9chr13	59965845	59966051	7chr13	62867283	62867499	8chr1	36982044	36982225	9chr13	71865347	71865563	10chr13	75793967	75794145	6chr13	77047795	77048029	8chr1	38495753	38496003	8chr13	92050684	92050948	10chr1	39491816	39492016	10chr13	95923419	95923709	9chr13	96146401	96146615	7chr13	98507846	98508028	7chr1	39864357	39864564	10chr13	99492491	99492663	8chr1	40004931	40005118	9chr1	40282539	40282739	8chr1	40550465	40550647	9chr1	40840257	40840448	7chr14	100413334	100413514	9chr14	100542563	100542774	9chr14	100713281	100713503	7chr14	101159562	101159786	9chr14	103241323	103241512	9chr14	103384244	103384450	8chr14	103966559	103966762	9chr14	104376701	104376906	7chr14	105030452	105030610	7chr14	105818597	105818800	7chr14	105915001	105915243	8chr14	107238960	107239148	9chr1	41157500	41157788	9chr1	41414637	41414830	8chr1	41733922	41734101	8chr14	19038092	19038195	8chr14	21482943	21483118	7chr14	21852067	21852259	7chr14	22855858	22856139	9chr14	23169368	23169615	8chr14	23369538	23369696	7chr14	24583859	24584172	9chr14	25501737	25501922	9chr14	30692668	30692909	8chr14	31739409	31739691	7chr14	33045395	33045590	8chr1	43432837	43433078	8chr14	35834800	35835034	9chr1	43672634	43672836	9chr14	45722275	45722446	9chr1	44997816	44998065	11chr14	50465925	50466123	9chr14	50697418	50697711	9chr14	50779114	50779333	9chr14	50863375	50863701	9chr14	52001382	52001582	10chr1	45251854	45252076	8chr14	55196895	55197161	9chr14	55221513	55221742	11chr14	55921286	55921495	7chr14	56269857	56270078	9chr14	56666001	56666191	8chr14	56958113	56958301	7chr1	45732143	45732295	6chr14	57459106	57459325	9chr14	58764546	58764786	9chr14	64331084	64331281	10chr1	46769267	46769478	10chr14	68286365	68286578	10chr14	69149637	69149837	8chr14	69152276	69152523	8chr14	69261150	69261388	8chr14	70092145	70092376	11chr14	70883700	70883906	8chr14	74079330	74079576	8chr14	74296822	74297039	9chr14	75229900	75230214	10chr14	75412003	75412213	8chr14	75469444	75469643	9chr14	76044116	76044361	9chr14	76669822	76670108	10chr14	77385044	77385367	10chr14	77591288	77591498	10chr14	77770122	77770374	8chr14	78121427	78121579	7chr14	79064487	79064631	8chr1	48035540	48035771	9chr14	81453861	81454049	9chr1	48204339	48204518	8chr14	86400629	86400876	9chr14	89466924	89467167	8chr14	91270169	91270341	11chr14	91893645	91893893	9chr14	93147480	93147667	9chr14	93312864	93313083	8chr14	93836060	93836341	10chr14	94952630	94952820	8chr14	95155933	95156074	7chr14	96741950	96742103	6chr15	101795888	101796054	8chr1	51655174	51655343	10chr1	52088178	52088407	9chr15	21927356	21927556	6chr15	22977324	22977509	11chr15	23378298	23378532	8chr15	32160105	32160323	9chr15	34339746	34339959	9chr15	34658478	34658717	8chr15	37426943	37427130	7chr15	40559076	40559343	10chr15	42281824	42281993	8chr15	42942712	42942905	8chr1	54344256	54344486	8chr15	44067723	44067882	7chr1	54482162	54482410	7chr15	45906517	45906703	9chr15	45926823	45927133	11chr15	50474306	50474485	8chr15	50550229	50550414	7chr15	51057365	51057614	6chr15	51520675	51520931	10chr15	51939430	51939618	10chr1	55606301	55606469	8chr15	56664045	56664275	10chr1	5570014	5570179	10chr1	55754008	55754197	11chr15	57853295	57853463	9chr15	58024813	58024982	6chr15	60884662	60884830	7chr15	64296898	64297152	7chr15	64303016	64303222	10chr15	65117689	65117920	9chr15	66155058	66155236	8chr15	66194753	66194974	9chr15	66896025	66896195	8chr15	69770632	69770822	8chr15	69849786	69849992	8chr15	69884311	69884496	9chr15	70265569	70265756	8chr15	73997211	73997433	7chr15	74340723	74340971	6chr15	75080187	75080377	9chr15	75246880	75247151	10chr15	76440319	76440536	7chr15	77197648	77197925	11chr15	78162876	78163078	8chr15	78504991	78505245	11chr15	80285819	80285979	8chr15	80534579	80534796	8chr15	81316105	81316313	8chr15	81398903	81399091	10chr15	84864443	84864651	9chr15	85144054	85144281	11chr15	85743528	85743734	10chr15	85901310	85901518	11chr1	58628702	58628893	7chr15	86402103	86402308	7chr15	89448056	89448239	9chr15	90099978	90100155	8chr15	90574629	90574884	9chr15	93631114	93631338	7chr15	98835792	98835991	8chr15	99202547	99202801	9chr1	60437140	60437291	7chr16	10591104	10591303	11chr16	10831030	10831237	9chr16	11194241	11194473	10chr16	11402763	11402970	10chr16	11890244	11890494	8chr16	1238812	1238980	6chr16	14383653	14383855	6chr16	14724081	14724307	10chr16	14991181	14991409	9chr16	16389829	16390063	9chr16	1973643	1973911	8chr16	20786157	20786339	8chr1	62107451	62107641	10chr16	21168152	21168347	9chr16	21719911	21720143	7chr16	22330064	22330286	8chr16	2244895	2245141	8chr16	22910158	22910432	7chr16	23912662	23912875	9chr16	24264516	24264767	6chr16	24674312	24674521	11chr16	24712386	24712575	8chr16	24850124	24850338	9chr16	25288050	25288241	7chr16	27246401	27246555	9chr16	27379490	27379685	8chr16	27879022	27879231	10chr16	28229540	28229724	9chr16	28333104	28333336	7chr16	28376121	28376369	8chr16	28891179	28891396	6chr16	29239514	29239696	7chr16	30066423	30066590	8chr16	30107535	30107712	8chr16	30198668	30198861	8chr16	30908433	30908603	10chr16	31008358	31008537	8chr16	33647096	33647257	10chr16	34625805	34625996	9chr16	4033094	4033296	9chr1	64239452	64239703	9chr16	4340324	4340530	7chr16	4349151	4349365	9chr16	4357601	4357828	8chr16	4700541	4700738	8chr16	47007642	47007829	9chr16	48157086	48157342	8chr16	48190744	48190917	7chr16	49891598	49891803	8chr16	49910652	49910803	7chr16	5106452	5106616	8chr16	51796056	51796267	8chr16	56574175	56574353	8chr16	57452825	57453081	9chr16	57573104	57573305	11chr16	57728607	57728848	9chr16	57850309	57850500	10chr16	58035130	58035280	8chr16	58695185	58695397	9chr16	67204116	67204425	8chr16	67433351	67433584	9chr16	67465134	67465371	7chr1	66748084	66748340	10chr16	67580903	67581141	8chr16	67596534	67596798	10chr16	68295503	68295703	9chr16	70687713	70687876	8chr16	74464017	74464159	8chr16	74896674	74896924	8chr16	75419512	75419720	7chr16	75540551	75540784	8chr16	79636119	79636403	9chr16	81027017	81027173	9chr16	81214914	81215141	8chr16	81529383	81529610	10chr16	81907580	81907739	8chr16	84178629	84178849	9chr1	6844527	6844732	10chr16	85028494	85028659	9chr16	85436688	85436895	11chr16	85604435	85604729	10chr16	85645878	85646104	9chr16	85852869	85853035	8chr1	68639825	68640062	8chr16	86551368	86551526	8chr16	87358356	87358555	6chr16	87970129	87970388	8chr16	88077800	88078027	9chr16	88752830	88753141	9chr16	88923332	88923560	9chr16	89988145	89988417	8chr16	90276484	90276718	9chr16	9254063	9254250	7chr17	1179542	1179739	8chr17	12719140	12719414	8chr17	1389302	1389535	9chr17	14204245	14204488	9chr17	15394041	15394275	10chr17	1553154	1553351	9chr17	15901844	15902047	8chr17	1619411	1619642	7chr17	1624174	1624347	9chr17	16267146	16267368	7chr17	17589247	17589448	10chr17	17598049	17598301	6chr17	1812123	1812293	7chr17	18965425	18965627	10chr17	19669852	19670062	8chr17	1973807	1973987	11chr17	25798774	25798991	9chr17	25905834	25905987	10chr17	26132586	26132757	7chr17	26207116	26207279	9chr17	27068456	27068720	8chr17	2731826	2731999	9chr17	27467223	27467483	8chr17	2833420	2833585	8chr17	28977954	28978199	10chr17	2916343	2916556	9chr17	29295640	29295855	8chr17	30466001	30466168	8chr17	31155528	31155698	7chr17	31204307	31204480	8chr17	31298255	31298477	10chr17	33866485	33866655	10chr17	34476405	34476605	8chr17	3461956	3462215	8chr17	34948046	34948313	10chr17	3505884	3506073	9chr17	35848369	35848641	9chr17	36192923	36193126	8chr17	36489605	36489849	7chr17	36505049	36505260	8chr17	36580013	36580176	8chr17	36641201	36641422	8chr17	37320931	37321136	8chr17	37910668	37910888	11chr17	3905710	3905926	9chr17	39705553	39705757	8chr17	39844867	39845156	10chr17	40333351	40333590	10chr17	40335205	40335425	9chr17	40472027	40472203	11chr17	40558117	40558351	9chr17	40559589	40559796	8chr17	4064596	4064823	9chr17	40670558	40670807	9chr17	40819567	40819795	8chr17	41751248	41751396	7chr17	42021421	42021604	8chr17	42160891	42161108	8chr17	42186109	42186348	8chr17	42365857	42366133	9chr17	42422468	42422729	6chr17	43096584	43096817	7chr17	43313111	43313413	7chr17	43430491	43430696	8chr17	44121741	44121957	9chr17	45726551	45726766	7chr17	46208674	46208901	9chr17	46985684	46985910	8chr17	46986339	46986546	9chr17	47109637	47109795	9chr17	47300838	47301125	9chr17	47585855	47586052	7chr17	48439360	48439631	7chr17	48758721	48758890	11chr17	4965792	4965983	7chr17	53591461	53591656	9chr17	54993264	54993509	10chr17	55860712	55860941	8chr17	56065862	56066091	8chr17	56190419	56190654	8chr17	56394871	56395076	8chr17	58469498	58469756	10chr17	58716162	58716337	7chr17	59467394	59467556	8chr17	60143329	60143528	7chr17	60170936	60171111	8chr17	60902227	60902423	8chr17	62703291	62703544	8chr17	62935077	62935270	8chr17	63225208	63225440	10chr17	63552588	63552759	8chr17	63911000	63911210	10chr17	6410194	6410376	8chr17	64188293	64188481	7chr17	64344811	64344991	7chr17	64563432	64563590	9chr17	65775194	65775446	7chr17	65796634	65796849	7chr17	66007310	66007557	8chr17	66086160	66086336	7chr17	67231695	67231854	9chr17	67853590	67853826	7chr17	6916635	6916907	10chr17	70610214	70610416	9chr17	71267328	71267524	9chr17	7140982	7141281	7chr17	72489578	72489732	9chr17	7255189	7255390	9chr17	72766949	72767094	7chr17	74001706	74001937	8chr17	74351059	74351299	9chr17	74404674	74404895	9chr17	7487230	7487472	8chr17	74998355	74998563	7chr17	75400976	75401179	10chr17	75456225	75456396	10chr17	76081136	76081342	9chr17	76352428	76352635	10chr17	76879307	76879517	8chr17	7745582	7745780	8chr17	7747233	7747511	9chr17	77879642	77879893	8chr17	78169932	78170134	9chr17	78475876	78476099	9chr17	79030969	79031169	9chr17	79319419	79319679	6chr17	79323686	79324037	10chr17	79486453	79486746	10chr17	79504639	79504867	6chr17	79519300	79519474	7chr17	79643046	79643199	10chr17	79919132	79919325	6chr17	79988425	79988627	10chr17	8013598	8013805	8chr17	80196726	80196961	9chr17	80376297	80376499	9chr17	80408479	80408821	11chr1	78149094	78149296	9chr1	8002436	8002672	7chr1	8100705	8100891	8chr1	8137331	8137585	8chr18	19180865	19181045	7chr18	19315584	19315808	10chr18	20046077	20046316	7chr18	23887258	23887468	9chr18	23963768	23963941	8chr18	29004415	29004630	5chr18	29070406	29070621	8chr18	3262016	3262260	9chr18	32935111	32935338	8chr18	34218286	34218432	7chr18	3453938	3454226	8chr18	39878765	39878997	9chr18	4106457	4106664	7chr18	44626730	44626884	9chr18	46067643	46067875	9chr18	46548230	46548444	7chr18	5073328	5073527	8chr1	85219309	85219501	7chr18	55746228	55746423	9chr18	55758634	55758816	8chr18	56282999	56283217	10chr18	56435715	56435981	7chr1	85804886	85805110	7chr18	60118385	60118597	8chr18	60253499	60253750	8chr18	60384620	60384869	9chr1	86042653	86042883	9chr18	6482604	6482825	8chr18	6521883	6522066	8chr18	67602703	67602893	8chr1	87299357	87299571	10chr18	741968	742154	6chr18	74533841	74534152	9chr18	75612137	75612301	8chr18	77165763	77166028	10chr18	8696833	8697025	8chr18	9117305	9117577	8chr1	89458195	89458402	9chr18	9913897	9914155	8chr1	90011235	90011426	9chr1	90158408	90158646	10chr1	90418852	90419053	8chr19	10110992	10111156	8chr19	10499660	10499852	9chr19	10531726	10531880	8chr19	10541969	10542144	9chr19	10764434	10764704	6chr19	10828007	10828234	10chr19	11047433	11047618	8chr19	11071420	11071711	10chr19	12550863	12551096	9chr19	12876861	12877065	8chr19	13358732	13358938	9chr19	1354650	1354839	8chr19	1407286	1407522	10chr19	14134429	14134653	7chr19	14153931	14154081	9chr19	14224955	14225153	10chr19	14693773	14693986	11chr19	15334265	15334510	8chr19	15574639	15574863	8chr19	16254336	16254586	5chr19	16435391	16435654	8chr19	16999491	16999833	9chr19	17316164	17316306	7chr19	17908326	17908600	10chr19	18133053	18133244	9chr19	18572723	18572898	8chr19	18580492	18580686	8chr19	18960748	18960946	4chr19	1905664	1905870	9chr19	19106649	19106872	9chr19	19145130	19145352	9chr19	19216285	19216486	9chr19	19702009	19702229	8chr19	19779575	19779835	11chr19	2163611	2163809	10chr19	21949914	21950256	10chr19	2598002	2598188	10chr19	2621820	2622007	11chr19	2637060	2637229	8chr19	30163970	30164214	9chr19	30221471	30221665	8chr19	31173911	31174112	7chr19	3185448	3185734	7chr19	34258851	34259036	8chr19	35760590	35760833	11chr19	3580234	3580436	9chr19	35942737	35942879	8chr19	36231869	36232201	11chr19	36310937	36311105	9chr19	36394588	36394765	8chr19	38826902	38827181	9chr19	39468610	39468936	11chr19	39842195	39842440	9chr19	40671539	40671713	7chr19	40727892	40728128	8chr19	41208580	41208752	10chr19	42502748	42502916	6chr19	43246280	43246477	10chr19	43588435	43588678	9chr19	43775341	43775508	9chr19	44952554	44952797	9chr19	4525205	4525420	7chr19	45562812	45562993	8chr19	4558430	4558681	6chr19	45841971	45842147	8chr19	45907453	45907692	7chr19	45930954	45931120	7chr19	4636351	4636554	7chr19	46377151	46377389	9chr19	4732707	4732879	9chr19	47731020	47731284	8chr19	48292138	48292320	9chr19	48366763	48366941	8chr19	48707079	48707313	8chr19	48833402	48833682	9chr19	48901902	48902031	10chr19	49376268	49376450	6chr19	49482199	49482408	9chr19	49601114	49601308	8chr19	49729379	49729543	8chr19	50000557	50000793	6chr19	50380732	50380920	8chr19	50427591	50427830	8chr19	51843321	51843546	8chr19	51911105	51911300	8chr19	51971352	51971536	8chr19	52302649	52302862	9chr19	52430565	52430758	11chr19	53662206	53662402	9chr19	55030060	55030254	8chr19	55580792	55581002	9chr19	55803392	55803617	8chr19	55897198	55897381	9chr19	55929612	55929841	6chr19	56616597	56616812	8chr19	56861908	56862109	9chr19	58011177	58011373	8chr19	5968062	5968285	9chr19	6218576	6218740	9chr1	9653563	9653754	7chr19	6616073	6616244	8chr1	96598	96818	9chr19	703667	703875	9chr19	7572905	7573138	9chr19	7985096	7985267	8chr1	97991206	97991418	7chr19	8333861	8334079	8chr19	8412713	8412874	7chr19	8455121	8455383	11chr19	8488908	8489077	8chr19	8764119	8764303	8chr1	9885568	9885801	6chr19	9434871	9435072	6chr19	9736594	9736755	10chr1	999215	999514	8chr20	1193590	1193837	7chr20	1396712	1396892	6chr20	14966869	14967106	10chr20	19796239	19796442	8chr20	22814635	22814822	9chr20	2327895	2328055	5chr20	23427337	23427511	5chr20	23587287	23587444	8chr20	26188898	26189093	7chr20	278811	279052	8chr20	2832774	2832998	9chr20	30190718	30190946	8chr20	30292397	30292607	10chr20	30424031	30424271	10chr20	305074	305300	10chr20	32778487	32778670	9chr20	33893593	33893769	11chr20	34129729	34130090	10chr20	34330387	34330649	11chr20	35471842	35472051	10chr20	37501840	37502071	9chr20	3792317	3792565	7chr20	3800764	3801047	11chr20	39763889	39764127	8chr20	39801831	39802017	7chr20	39969421	39969583	7chr20	41816504	41816658	6chr20	43313913	43314122	6chr20	43700003	43700191	8chr20	44992901	44993138	9chr20	47252425	47252576	7chr20	4742293	4742524	9chr20	47470495	47470723	9chr20	47833729	47833953	7chr20	48334307	48334528	7chr20	48381735	48381921	8chr20	48392877	48393070	10chr20	48532991	48533259	9chr20	48646535	48646781	8chr20	48720335	48720586	8chr20	49039787	49039989	9chr20	49242713	49242859	8chr20	50366781	50366957	9chr20	5509641	5509817	7chr20	56089357	56089610	9chr20	5700596	5700817	10chr20	5741426	5741591	9chr20	61187598	61187817	8chr20	62013258	62013420	7chr20	62313945	62314165	8chr20	9959162	9959325	7chr2	100106879	100107145	8chr2	101741496	101741679	9chr2	101822073	101822313	8chr2	10260402	10260623	8chr2	10260682	10260990	9chr2	10438127	10438361	10chr2	10545599	10545852	11chr2	105654458	105654672	9chr2	106162242	106162508	8chr2	107203925	107204129	8chr2	10952732	10952951	7chr2	11040399	11040677	8chr2	111874702	111874864	6chr2	112505325	112505502	8chr2	113938028	113938252	9chr21	15352558	15352779	9chr2	118870528	118870703	9chr2	119496187	119496432	8chr2	11969367	11969661	10chr2	119932107	119932291	6chr2	120584004	120584192	8chr2	12214408	12214602	10chr21	26548979	26549187	8chr2	127751993	127752193	10chr2	127825681	127825856	5chr2	128211026	128211240	8chr2	128459745	128460014	7chr2	130345048	130345284	7chr2	130913718	130913883	8chr2	131631364	131631564	10chr21	32926865	32927107	9chr21	32953208	32953365	8chr21	33104256	33104506	8chr21	34499685	34499915	6chr2	134601895	134602094	7chr21	36421060	36421311	6chr2	136742912	136743112	10chr2	136993683	136993892	8chr21	37794553	37794788	8chr21	42655274	42655435	8chr21	42738648	42738929	9chr21	43099124	43099412	8chr21	43518021	43518187	8chr21	43537647	43537783	8chr21	43608437	43608642	11chr21	43880965	43881219	10chr21	44395500	44395753	9chr21	44537339	44537529	9chr21	44597225	44597472	7chr21	44922396	44922653	9chr21	45148289	45148576	10chr21	45595117	45595375	9chr21	46676892	46677116	11chr21	46738793	46739026	10chr21	46970879	46971062	6chr21	47649093	47649323	8chr2	148386584	148386743	3chr2	150990206	150990453	8chr2	153574865	153575059	7chr2	158866821	158867082	9chr2	159071049	159071218	9chr2	161067210	161067403	8chr2	163077520	163077752	9chr2	169907294	169907556	8chr2	171217897	171218154	8chr2	171265635	171265837	9chr2	174075370	174075576	6chr2	175562018	175562207	8chr2	175584636	175584870	9chr2	177796638	177796860	8chr2	178199782	178199953	6chr2	178483494	178483685	9chr2	178770909	178771146	8chr2	181845379	181845562	9chr2	1821327	1821529	7chr2	190953646	190953871	9chr2	191217663	191217896	9chr2	191269386	191269569	10chr2	192542459	192542791	10chr2	19549943	19550160	7chr2	196409356	196409582	6chr2	197040894	197041147	10chr2	197852730	19785
[truncated: 1,200,000 more chars]
