# Supplementary material for: Non-targeted transcription factors motifs are a systemic component of ChIP-seq datasets
Source: Genome Biol. 2014 Jul 29;15(7):412. doi: 10.1186/s13059-014-0412-4 (PMC4165360; doi:10.1186/s13059-014-0412-4)
Supplement: Additional file 13: Table S1. — The top 20 motifs from motif over-representation analysis on HOT regions. [file 13059_2014_412_MOESM13_ESM.pdf]

Table S1

Enriched TF motif	TF matrix Information content	Fisher log score	Fisher p-value	Zinger motif
CTCF	17.205	-Inf	0	CTCF-like
CTCFL	15.983	478.563	1.4e-208	CTCF-like
CTCF::SMC3	15.886	456.433	5.9e-199	CTCF-like
CTCF::RAD21	14.398	239.965	6.1e-105	CTCF-like
HLF	11.147	171.034	5.3e-75	
CEBPB	12.334	142.261	1.6e-62	JUN-like
JUND	14.840	128.454	1.6e-56	JUN-like
ELK4	13.433	122.643	5.5e-54	ETS-like
IRF1-1	16.008	120.295	5.7e-53	
GABPA	13.821	111.216	5.0e-49	ETS-like
PRRX2	9.063	105.924	9.9e-47	
HIF1A::ARNT	9.740	102.951	1.9e-45	
ARNT	10.992	101.559	7.8e-45	
JUN	13.308	101.034	1.3e-44	JUN-like
E2F4	11.346	95.011	5.5e-42	
MIZF	13.197	89.491	1.4e-39	
THAP11::SIX5	23.317	81.275	5.0e-36	THAP11
BATF	13.028	79.320	3.6e-35	JUN-like
FOSL2	13.999	78.750	6.3e-35	JUN-like
CREB1	10.139	73.909	8.0e-33	JUN-like
