# Supplementary material for: Non-targeted transcription factors motifs are a systemic component of ChIP-seq datasets
Source: Genome Biol. 2014 Jul 29;15(7):412. doi: 10.1186/s13059-014-0412-4 (PMC4165360; doi:10.1186/s13059-014-0412-4)
Supplement: Additional file 14: Dataset S2. — Position frequency matrices. [file 13059_2014_412_MOESM14_ESM.rtf]

#Position Frequency Matrices#>AP1A  [304 559   0   0 887  69   0  63 887  33 335 ]C  [212  63   0   0   0 367   0 824   0 338 292 ]G  [245 234   0 887   0 388   0   0   0 292  37 ]T  [126  31 887   0   0  63 887   0   0 224 223 ]>ARID3AA  [27  0  1 27 27 20 ]C  [ 0  0  9  0  0  0 ]G  [ 0  0  0  0  0  1 ]T  [ 0 27 17  0  0  6 ]>ArA  [ 9  9 11 16  0 12 21  0 15  4  5  6  3  0  4 11  1  3  6  6 10  5 ]C  [ 7  2  3  1  0  6  2 24  0  9 11  9  5  0  0  5 22 16  7  5 11 11 ]G  [ 2  3  3  7 22  1  0  0  8  2  2  9  1 24  0  1  1  1  5  9  0  6 ]T  [ 6 10  7  0  2  5  1  0  1  9  6  0 15  0 20  7  0  4  6  4  3  2 ]>Arnt::AhrA  [ 3  0  0  0  0  0 ]C  [ 8  0 23  0  0  0 ]G  [ 2 23  0 23  0 24 ]T  [11  1  1  1 24  0 ]>ArntA  [ 4 19  0  0  0  0 ]C  [16  0 20  0  0  0 ]G  [ 0  1  0 20  0 20 ]T  [ 0  0  0  0 20  0 ]>Atf3A  [ 92  20   0 495   0  12   0   0 472   0  53  85 ]C  [153 201 498   0 408   1   0   0  10 460 257 175 ]G  [233  83   0   0  12 492   0 505  23   1  88 154 ]T  [ 27 201   7  10  85   0 505   0   0  44 107  91 ]>BatfA  [247 463   0   0 651  41   0 150 651 154 224 ]C  [  5  81   0   0   0 304   0 501   0 124 170 ]G  [147  34   0 644   0 306   0   0   0 201 110 ]T  [252  73 651   7   0   0 651   0   0 172 147 ]>Bcl11aA  [ 57   9  12   6 434   0  22   0   0   8  37  28 ]C  [ 56 173  96 500  19 241   5   0 439 378 160 303 ]G  [ 48  45   9   0  13 182   0   5   0   0  44  52 ]T  [356 290 400  11  51  94 490 512  78 131 276 134 ]>BHLHE40A  [ 117    5    0  915    1   55   11    0  223  218 ]C  [ 178   51 1000   31  921   50   20    3  479  243 ]G  [ 583  363    0   15   28  886   39  884   62  293 ]T  [ 122  581    0   39   50    9  930  113  236  237 ]>BRCA1A  [144 100   7  43   1   0   7   0   0 388   4 321  90  38 ]C  [ 89 131  10 390  39 434   2 429   0   4  29   2 108 171 ]G  [157 103  30   1  17   0 425   1 434  34 332 107 131  80 ]T  [ 43 100 387   0 377   0   0   4   0   8  69   4 105 145 ]>CEBPAA  [ 3  1  4  2  4  2 18 18  0 ]C  [ 0  1  1  9  2 15  0  0  6 ]G  [ 0  4  6  2 10  0  0  0  2 ]T  [15 12  7  5  2  1  0  0 10 ]>CEBPBA  [ 253  181  591    1    0   73    0  588  163  679  999   13  330  240 ]C  [ 223  153  125    1    0    1 1000   86  464  321    0  411  310  259 ]G  [ 257  314  276    0    7  799    0  201    2    0    1  105  110  213 ]T  [ 267  352    8  998  993  127    0  125  371    0    0  471  250  288 ]>CREB1A  [ 0  0 11  0  1  0  2  8 ]C  [ 1  1  0  9  0  3  7  0 ]G  [ 1 10  0  2 10  0  1  1 ]T  [ 9  0  0  0  0  8  1  2 ]>CTCFLA  [176 161  44  19 341  79 114 805   0 192  62  14  20  84 134  55 133 202 ]C  [299 221 785 919  97 522 532  47   0   6  54   0  23 868  48 646 452 307 ]G  [379 408 109  13 396 349 107  70 962 764 731 939 884   1 740 178 124 350 ]T  [102 168  24  11 128  12 209  40   0   0 115   9  35   9  40  83 253  99 ]>CTCFA  [ 87 167 281  56   8 744  40 107 851   5 333  54  12  56 104 372  82 117 402 ]C  [291 145  49 800 903  13 528 433  11   0   3  12   0   8 733  13 482 322 181 ]G  [ 76 414 449  21   0  65 334  48  32 903 566 504 890 775   5 507 307  73 266 ]T  [459 187 134  36   2  91  11 324  18   3   9 341   8  71  67  17  37 396  59 ]>Ddit3::CebpaA  [14 11 18  0  0  4 38 36  0 14  4  0 ]C  [ 7  7  3  1  0 33  1  2  6 17 23 26 ]G  [12 14 15  0 38  0  0  1  0  5  9  6 ]T  [ 6  7  3 38  1  2  0  0 33  3  3  7 ]>E2f1-HeLaA  [ 259  218  144    0    0    0    0    0 1059  508  305 ]C  [ 317    0  274    0 1059    0  337  286    0    0  269 ]G  [ 280  628  641 1059    0 1059  722  773    0  551  485 ]T  [ 203  213    0    0    0    0    0    0    0    0    0 ]>E2f4-HeLasA [ 110  65  43  64 149 214 190  60  17  16   0   0   3   0   0   2]C [ 133 150 162 193 147 118  89 134 124  46 430 389 490   0 472 321]G [ 132  96  88  81 147  83  76  58  21  39  57 111   0 485  26 128]T [ 125 189 207 162  57  85 145 248 338 399  13   0   7  15   2  49]>E2f6-HeLaA [  98  78  48   0  41   0   0   0 300 209 108  75]C [  69  24  53   0 240   0   0   0   0   0  57  87]G [  75 140 198 300   0 300 300 300   0  90 108 101]T [  56  57   0   0  18   0   0   0   0   0  24  36]>EBF1A  [ 63  41   0   6   0 381 161  16  81   0 736 177 182 186 ]C  [217 304 858 624 849   8   5   0   0   0  37 351 289 183 ]G  [388  45   0   0   0  39 592 842 777 858  78 233  76 181 ]T  [190 468   0 228   9 430 100   0   0   0   7  97 311 308 ]>Egr1A  [177 143  63 136   0   0  58   0   0 113   4  53 212 237 ]C  [278 172  72 712   0   1   0   0   0 644   3  27 184 130 ]G  [412 340 845  15 995 547 937 988 995   0 966 726 456 495 ]T  [128 340  15 132   0 447   0   7   0 238  22 189 143 133 ]>ELF5A  [12 29  2 14  0  0  0  4  4 ]C  [ 9  3 20  0  1 44 44  3  8 ]G  [ 3  7  5  1  0  0  0 11 11 ]T  [20  5 17 29 43  0  0 26 21 ]>ELK1A  [ 7 10  9  5  2  0  1 27 21 13 ]C  [ 7  6  4 19 24  0  0  0  5  0 ]G  [10  6 10  1  1 24 27  1  0 14 ]T  [ 4  6  5  3  1  4  0  0  2  1 ]>ELK4A  [ 222  350   23   88    5    0 1000  973  190   34  147  218  205 ]C  [ 276  190  846  903    0    0    0    0   22  304  135  244  330 ]G  [ 396  375  129    1  995 1000    0    0  788   34  593  459  341 ]T  [ 106   85    2    8    0    0    0   27    0  628  125   79  124 ]>ESR1A  [122 107  64  83 134 308  36  19  33   4 398  58  63  64  32  21 305  10  59  26 ]C  [120  80 173 229 232  28   8  18  41 394  13 250 276 258  19  22 106 436 353 165 ]G  [154 164 149  65  47  89 387 420  91  53  27 107  53  97   8 426  31  12   2  22 ]T  [ 71 117  82  93  57  48  43  18 310  24  37  60  83  56 416   6  33  17  61 262 ]>ESR2A  [ 78 158 186  27  18  13   0 337  49  64  52  21  63 178  34  46  27  60 ]C  [161  51  15   0  20  19 357   1 123  63  61  33  25  99 268 289  90  94 ]G  [ 63  41 154 275 319  33   0   0 113 149 147  24 265  19   2   0   0 136 ]T  [ 55 107   2  55   0 292   0  19  72  81  97 279   4  61  53  22 240  67 ]>EsrraA  [122  68   8   6 479  14   4   1   4   0 190  87 ]C  [126 167  27  29  96 630 633  43  61  78  53 235 ]G  [150 312  18 610  18   1   0   0  36 447 340 254 ]T  [247  98 592   0  52   0   8 601 544 120  62  69 ]>EsrrbA  [1055  673  403  260  312 3347 3590   33   12  180    9 3478 ]C  [ 801  832 1212 1230 2653   32    2    2   11   43 3394   21 ]G  [ 984 1352 1212  449  630  248   60 3597 3622  243  167  128 ]T  [ 773  767  807 1702   51   27    6   29   16 3192   85   26 ]>Evi1A  [14 20  0 27  1 27 26  0 27  0 24 23  6 15 ]C  [ 2  1  1  0 10  0  0  0  0  3  1  0  7  6 ]G  [ 6  2 25  0  0  0  1 27  0  0  0  4  7  3 ]T  [ 5  4  1  0 16  0  0  0  0 24  2  0  7  3 ]>EWSR1-FLI1A  [  0   2 104 104   1   2 103 102   0   0  99 105   0   0 100 102   5   3 ]C  [  0   0   0   0   0   0   0   0   0   2   4   0   0   2   3   0   0   3 ]G  [105 103   1   1 104 102   2   3 104 103   2   0 105 103   0   2  97  97 ]T  [  0   0   0   0   0   1   0   0   1   0   0   0   0   0   2   1   3   2 ]>FEVA  [ 2  9  0  0 13 12  7  0 ]C  [ 9  3  0  0  0  0  0  0 ]G  [ 1  1 13 13  0  0  6  0 ]T  [ 1  0  0  0  0  1  0 13 ]>FOSL1A  [219 389   0   0 965   4   0   0 962   8 136 ]C  [261 274   6   0   9 404   5 995  22 480 624 ]G  [229 323   3 975  16 577  11   5   0  65  66 ]T  [288  14 991  25  10  15 984   0  16 447 174 ]>Fosl2A  [223 280   0   0 967  15   0  22 967  13 102 ]C  [ 48 238   0   0   0 500   0 945   0 218 372 ]G  [432 433   0 962   0 410   0   0   0  81 262 ]T  [264  16 967   5   0  42 967   0   0 655 231 ]>FOXA1A  [ 13 108   1   4   6 478   4 289  42 379  89 ]C  [  1   7   7   7   3  12 808 106 369   7 125 ]G  [  2 772   4  24  77 368   5   5  69  24 467 ]T  [876   7 885 862 811  38  79 496 414 482 211 ]>Foxa2A  [  1 221   1   1   1 736   2 338  50 417 131 129 ]C  [  1   3   1   3   1   4 725  77 265   6  53 195 ]G  [  1 582   1  36 175  67   2   3  58  24 482 290 ]T  [805   3 806 769 634   3  81 391 436 360 140 189 ]>FOXD1A  [ 1  0 19 20 18  1 20  7 ]C  [ 1  0  1  0  1 18  0  2 ]G  [17  0  0  0  1  0  0  3 ]T  [ 1 20  0  0  0  1  0  8 ]>Foxd3A  [11 30 24  1 12  0  1  5 14  6  1  0 ]C  [ 3  2  1  4  0  0  0  0  0  7  2 12 ]G  [26  0  0  0 34  0  0 10 21  0  4  0 ]T  [ 7 15 22 42  1 47 46 32 12 34 40 35 ]>FOXF2A  [ 1 10 17 13  3  7  0 27 27 27  0 27 16  7 ]C  [10  7  4  5 11  0  0  0  0  0 25  0  4  4 ]G  [ 7  5  2  5  8 20  0  0  0  0  0  0  2  6 ]T  [ 9  5  4  5  0  0 27  0  0  0  2  0  5 10 ]>FOXI1A  [ 6  4 11  0 13  0  0  0 15  0  8  2 ]C  [ 1  8  4  0  0  0  0  0  0  0  1  1 ]G  [14 12  8  0 18  0  0  0 16  7  4  4 ]T  [10  7  8 31  0 31 31 31  0 24 18 24 ]>FOXL1A  [ 7 10  6 13  4 21  0 22 ]C  [ 1  4  3  4 10  0  2  1 ]G  [ 4  2  6  4  2  2  0  0 ]T  [11  7  8  2  7  0 21  0 ]>FOXO3A  [ 0  1  3 13 12 13  0 12 ]C  [ 0  2  0  0  1  0 12  0 ]G  [ 5 10  1  0  0  0  0  0 ]T  [ 8  0  9  0  0  0  1  1 ]>Foxq1A  [ 4 13  5  3  0  0  0  0 17  0  6 ]C  [ 4  1  2  0  0  0  0  0  0  1  0 ]G  [ 3  3  0  0 18  0  0  0  1  4  3 ]T  [ 7  1 11 15  0 18 18 18  0 13  9 ]>GABPAA  [307 396  55 102   0   0 994 992  75  40 127 221 ]C  [320 179 754 892   0   0   0   0  15 227 155 292 ]G  [262 379 185   0 994 994   0   0 904  21 595 382 ]T  [105  40   0   0   0   0   0   2   0 706 117  99 ]>Gata1A  [ 339  196  838    0  969  169  926  858  222  415 ]C  [ 176  454    0    0    0    3    1    0   49  123 ]G  [ 257  343    0 1000   31  190   73  141  726  458 ]T  [ 227    7  162    0    0  638    0    1    3    4 ]>GATA2A  [ 377  179  718    0 1000  100  863  897  242  447 ]C  [ 122  487    0    0    0    2   13    0  150  186 ]G  [ 286  316    0 1000    0  113  122   98  580  293 ]T  [ 215   18  282    0    0  785    2    5   28   74 ]>GATA3A  [ 44  86   0 195   0  26  18  14 195   0   0  54  43 ]C  [ 62  10   0   0   0  37  55  44   0   0 195   0  50 ]G  [ 45  16 195   0   0  55  30   0   0   0   0   0  50 ]T  [ 44  83   0   0 195  77  92 137   0 195   0 141  52 ]>GfiA  [ 9 28 53 53  1  1 31  8 19  7 ]C  [20 16  0  0  0 52  7 28 11 13 ]G  [ 9  7  0  0  1  0  2 11  2 28 ]T  [15  2  0  0 51  0 13  6 21  5 ]>Hand1::Tcfe2aA  [ 4 10  2  0  0  0  0  9 16  5 ]C  [ 8  0  2 28  0  0  3 14  0  4 ]G  [10 15  1  0  0 29 25  1  3  4 ]T  [ 7  4 24  1 29  0  1  5 10 16 ]>HIF1A::ARNTA  [ 27  10  78   0   0   0   0  18 ]C  [ 28  29   2 103   0   0   0  51 ]G  [ 49  34  23   0 104   0 104  20 ]T  [  0  31   1   1   0 104   0  15 ]>HLFA  [ 1  6  1  0 13  0  6  0 13 15  2  5 ]C  [ 4  0  0  0  1 15  0  9  4  0  3  5 ]G  [ 8 12  0  3  2  1 12  0  1  1  1  3 ]T  [ 5  0 17 15  2  2  0  9  0  2 12  5 ]>HNF1AA  [ 5  1  1  1 20 16  1  8 14  2  0 13  8  5 ]C  [ 0  0  0  0  0  2  0  2  0  0  4  1  8 13 ]G  [14 20  0  0  0  1  0  4  1  0  0  3  3  0 ]T  [ 2  0 20 20  1  2 20  7  6 19 17  4  2  3 ]>HNF1BA  [0 0 8 7 0 5 3 0 0 8 9 0 ]C  [3 3 1 1 0 0 0 0 0 0 0 8 ]G  [0 0 0 1 0 4 1 1 0 1 0 0 ]T  [6 6 0 0 9 0 5 8 9 0 0 1 ]>HNF4AA  [113 165 163  10 959 785 893   4  50  40  12 789 ]C  [ 38  97 375 913  15   8   0   8  11 535 939  73 ]G  [753 308 195   6  26 181 101 967 186 113   2  45 ]T  [ 96 430 267  71   0  26   6  21 753 312  47  93 ]>Hnf4gA  [361 316 475 113 165 161   9 965 829 907  10  19  39  26 712 234 277 ]C  [225 197  46  28  78 373 918  12   7   0   9   9 476 853  95 248 204 ]G  [217 352 369 775 345 216   5  17 148  82 957 337 123  10  83 310 267 ]T  [197 135 110  84 412 250  68   6  16  11  24 635 362 111 110 205 249 ]>HOXA5A  [ 2  7  0  6 14 14  0  0 ]C  [13  0  7  0  0  0  0  1 ]G  [ 0  5  5  1  0  2  0  6 ]T  [ 0  4  4  9  2  0 16  9 ]>Hsf1A  [ 53 103  57 171   3 309 283  96  70  32  20   0   3 226   0 297 272  88 ]C  [117  98 103  12   2  17  15  90 101  20   4 336 141   7   3  16  12  93 ]G  [ 95  88  73 143 331   1  21  99  93  21  39   0   9 102 333  19  42 108 ]T  [ 70  46 103  10   0   9  17  51  72 263 273   0 183   1   0   4  10  47 ]>INSM1A  [ 1  0  0  6 16  0  0  0  0  0  3 10 ]C  [ 0  0  8 15  0  0  1  0  0  2 16  0 ]G  [ 4 20  3  0  0 24 23 24 24 16  0 12 ]T  [19  4 13  3  8  0  0  0  0  6  5  2 ]>IRF1-1A  [ 6 19 19 20  5  0  1 20 19 20  1  1 ]C  [ 4  0  0  0  3 10  1  0  1  0 13 13 ]G  [10  1  0  0 11  0 18  0  0  0  6  1 ]T  [ 0  0  1  0  1 10  0  0  0  0  0  5 ]>IRF1-2A  [ 51 250 183   8   1 533 543   0  86 ]C  [206  41  49 533 539   0   2  10 248 ]G  [113 239 267   0   0   0   0   9 188 ]T  [175  15  46   4   5  12   0 526  23 ]>IRF2A  [ 0  2 12 11 12  2  0  0 12 12 12  0  0  5  6  6  5  3 ]C  [ 4  0  0  0  0  0  6  0  0  0  0  6  7  2  0  1  2  6 ]G  [ 7 10  0  0  0 10  0 12  0  0  0  6  2  3  2  1  1  1 ]T  [ 1  0  0  1  0  0  6  0  0  0  0  0  3  2  4  4  4  2 ]>IRF3A  [453 248   0   0 893 893   0 128 554 ]C  [ 57  60 893 893   0   0   0 440  20 ]G  [363 478   0   0   0   0   0 298 317 ]T  [ 20 107   0   0   0   0 893  27   2 ]>Irf4A  [326  87 159  32  26   1 750  26  84   0   1  53 119  76 282 217 ]C  [155 322 115 345 158 938  60 420  46  58 767 642 300 529 252 192 ]G  [233 353  54  95  28   0  46 374   2   0  39   0 135 106  62 123 ]T  [282 238 672 528 788  61 144 180 868 942 193 305 446 289 404 468 ]>JUNDA  [146 429   0   0 818   0   0  24 818  13 178 ]C  [121 110   0   0   0 785   0 794   0 217 340 ]G  [363 279   0 818   0   2   0   0   0 157 132 ]T  [188   0 818   0   0  31 818   0   0 431 168 ]>JUNA  [302 425   0   0 833  48   0  79 833  41 279 ]C  [ 72  68   0   0   0 337   0 754   0 206 241 ]G  [337 334   0 833   0 407   0   0   0 161 174 ]T  [122   6 833   0   0  41 833   0   0 425 139 ]>Klf4A  [1468   88   14   14  277   22   42  264  123  254 ]C  [  81    9   13   36 1922   15   80   46  130 2858 ]G  [1022 4237 4304 4281   11 4273 2179 3902 3789  280 ]T  [1765    6   13   16 2139   34 2042  125  289  932 ]>Lhx3A  [ 9 16 19  0  0 19 20  2  0 16  9  2  2 ]C  [ 0  2  0  0  0  0  0  2  1  1  0  8  9 ]G  [ 3  2  0  0  0  0  0  0  0  0  3  0  3 ]T  [ 8  0  1 20 20  1  0 16 19  3  8 10  6 ]>MafbA  [ 0  1  0  1 12  3  3  2 ]C  [ 0 12  3  0  2  8  1  5 ]G  [15  1  1 12  0  1  7  5 ]T  [ 0  1 11  2  1  3  4  3 ]>MaffA  [673 100   4  21 995   1   1 920 359 414 ]C  [ 61 299   2 979   0   4 998   4 159  82 ]G  [ 97 564   2   0   5 879   1  22 176  58 ]T  [169  37 992   0   0 116   0  54 306 446 ]>MAFKA  [652 107   9  36 996   0   0 909 375 418 ]C  [ 71 305   2 962   0   3 998   0 148  70 ]G  [ 89 531   0   0   2 899   0  20 166  57 ]T  [186  55 987   0   0  96   0  69 309 452 ]>MAXA  [356 142   0 873   0 186   0   0  59 187 ]C  [ 66 229 873   0 873   0   0   0 213 264 ]G  [423 261   0   0   0 687   0 873 462 247 ]T  [ 28 241   0   0   0   0 873   0 139 172 ]>MEF2AA  [306 107  40   0 935  74  76  22 175 150 580 167 379 349 ]C  [ 51  71 642  70   0  11   0  45 104  48   0  52 356 238 ]G  [221 408  70   0  24   1   0   1  14  95 420 628  82  91 ]T  [422 414 248 930  41 914 924 932 707 707   0 153 183 322 ]>MIZFA  [ 2 13 20  2  0  0  0  0  0  2 ]C  [ 6  1  0 17  0  1 19 18  0 13 ]G  [ 5  0  0  1 19  0  0  2 19  1 ]T  [ 7  6  0  0  1 19  1  0  1  4 ]>MXI1A  [112  55   0 577   0  86   0   0  21  19 ]C  [178 277 577   0 577   0   0   0  82 246 ]G  [ 91 173   0   0   0 491   0 577 304 144 ]T  [196  72   0   0   0   0 577   0 170 168 ]>MybA  [ 8 22  2 16  1  2  0  6 ]C  [ 4  1 48 17  0  1  0  0 ]G  [34 25  1 16 50  0  0 44 ]T  [ 5  3  0  2  0 48 51  1 ]>MycA  [259  77   0 638   0 192   0   0  17  73 ]C  [ 71 147 638   0 578   0   0   0  88 275 ]G  [120 380   0   0  47 446   0 638 411 188 ]T  [188  34   0   0  13   0 638   0 122 102 ]>MYC::MAXA  [ 7 15  2  1 21  0  1  0  0  1  3 ]C  [ 1  1  9 20  0 20  0  1  0  1  5 ]G  [ 9  4  9  0  0  0 20  0 21 18  0 ]T  [ 4  1  1  0  0  1  0 20  0  1 13 ]>MycnA  [153  39   7 414   0  31  24   0  27  61 ]C  [159 170 431   0 421   1  97   0  49 265 ]G  [ 63 196   0  18   8 405   2 411 324  40 ]T  [ 63  33   0   6   9   1 315  27  38  72 ]>MyfA  [ 7  9  4  0 16  7  0  6  0  0  6  0 ]C  [ 8  0  2 15  0  0 15  0  0 10  0  0 ]G  [ 1  7 10  1  0  9  1  0 16  6  0 16 ]T  [ 0  0  0  0  0  0  0 10  0  0 10  0 ]>MYODA [ 102   0 452   1   0   0   0   5   5  99  46  61]C [ 123 474   2 119 474   0   0 174 160 130 214 151]G [ 181   0  15 324   0   0 474  28  59 150  76  86]T [  68   0   1  28   0 474   0 265 248  93 137 174]>MYOGENINA [  53 138  52   0 484   0   0   0   0   0   0 100  36  68]C [ 221 120 123 484   0  51 484   0   0 198 265 169 233 181]G [ 117  91 247   0   0 432   0   0 484   6  34 110 103 101]T [  92 134  60   0   0   0   0 484   0 279 184 104 109 132]>MZF1A  [ 3  0  2  0  0 18 ]C  [ 5  0  0  0  0  0 ]G  [ 4 19 18 19 20  2 ]T  [ 8  1  0  1  0  0 ]>MZF1A  [ 1  2 15  0  0  0  0  3 10  8 ]C  [ 4  0  1  0  0  2  0  1  0  2 ]G  [ 7  7  0 11 15 14 14  8  4  4 ]T  [ 4  7  0  5  1  0  2  4  2  2 ]>NANOGA  [111  68   0   0 172   0   0   0   2   0  44  41  78 ]C  [ 96 111 241 329   0   0   0  74   0 164 167 170 162 ]G  [ 98 136 103   0   0   0   0 382   0  60   1  45  77 ]T  [147 140 112 127 284 456 456   0 454 232 244 200 139 ]>NFATC2A  [ 3  1  1  0  1  0 18 ]C  [ 1  2  1  0 25 26  3 ]G  [ 2  2  0  0  0  0  1 ]T  [20 21 24 26  0  0  4 ]>NFE2L2A  [10  0  0 20  0  6  5 16  0  0 15 ]C  [ 1  0  0  0 17  2 10  0  0 20  2 ]G  [ 9  0 19  0  1  1  1  2 20  0  2 ]T  [ 0 20  1  0  2 11  4  2  0  0  1 ]>NFE2A  [160  46   1 695   0  21   0   0 641   0  88 115 ]C  [185 285 700   0 541  13   0   0  23 603 330 258 ]G  [316  85   0   1  10 666   0 701  32   9 101 192 ]T  [ 40 285   0   5 150   1 701   0   5  89 182 136 ]>NFICA  [ 358   81   81   91 1226 3298 ]C  [1832   67   67   88 5364  981 ]G  [ 176  300 6713 6643  160 1186 ]T  [4546 6464   51   90  162 1447 ]>NFIL3A  [ 1  0 22  0  2  0 23 22  0  7  5 ]C  [ 0  0  0  8  0  0  0  0 11  5  5 ]G  [ 0  2  0  0 21  0  0  1  4  7  3 ]T  [22 21  1 15  0 23  0  0  8  4 10 ]>NF-kappaBA  [ 0  0  1 25 19  7  1  2  2  0 ]C  [ 0  0  0  0 13  1  2 17 35 36 ]G  [38 38 37 13  1  3  2  0  0  0 ]T  [ 0  0  0  0  5 27 33 19  1  2 ]>NFKB1A  [238 367   5   0   0 339 358 115   0   7   0  27 313 318 ]C  [257  51   9   0   0   9 275  29  22 173 835 791 351 191 ]G  [126 217 814 874 773 479 140   1  11  19   0   0  57 241 ]T  [253 239  46   0 101  47 101 729 841 675  39  56 153 124 ]>NFYAA  [364 433   0   0 985 985   0  58 720 ]C  [159  22 985 985   0   0   4 637  16 ]G  [413 418   0   0   0   0   0 279 249 ]T  [ 49 112   0   0   0   0 981  11   0 ]>NfybA  [ 92  82 467 264   0   3 994 989   0  81 663 116 508 366 ]C  [393 394  54  38 998 994   0   4  11 572  27 129 243  80 ]G  [170 193 457 585   1   2   0   6   0 325 309 724 197 412 ]T  [344 330  21 112   0   0   5   0 988  21   0  30  51 141 ]>NHLH1A  [13 13  3  1 54  1  1  1  0  3  2  5 ]C  [13 39  5 53  0  1 50  1  0 37  0 17 ]G  [17  2 37  0  0 52  3  0 53  8 37 12 ]T  [11  0  9  0  0  0  0 52  1  6 15 20 ]>Nkx2-5A  [ 7  0 17 17  0  4  2 ]C  [ 0  4  0  0  0  2  1 ]G  [ 1  0  0  0  7  0 11 ]T  [ 9 13  0  0 10 11  3 ]>NKX3-1A  [13  0 20  0  1  1 19 ]C  [ 1  1  0 19  0  0  0 ]G  [ 3  0  0  0  0  0  1 ]T  [ 3 19  0  1 19 19  0 ]>Nkx3-2A  [ 4  1 13 24  0  0  6  4  9 ]C  [ 7  4  1  0  0  0  0  6  7 ]G  [ 4  5  7  0 24  0 18 12  5 ]T  [ 9 14  3  0  0 24  0  2  3 ]>NoboxA  [ 0 36 38  1  2 15  4  2 ]C  [ 1  0  0  1  0  0 12 13 ]G  [ 0  0  0  2  4 22 18  6 ]T  [37  2  0 34 32  1  4 17 ]>NR1H2::RXRAA  [17 17 20  0  0  0  0 25 24 25  0  0  0  0 25 20  0 ]C  [ 5  1  0  0  0  0 25  0  0  0  0  0  0 25  0  1 15 ]G  [ 0  5  5 25 24  0  0  0  0  0 25 25  0  0  0  2  6 ]T  [ 3  2  0  0  1 25  0  0  1  0  0  0 25  0  0  2  4 ]>Nr2e3A  [ 4 23 23  0  0  0  0 ]C  [12  0  0  1 23  0  0 ]G  [ 2  0  0 22  0  0  0 ]T  [ 5  0  0  0  0 23 23 ]>NR2F1A  [ 0  1 12  6  0  0  0  1  2  6  6  1  3  0 ]C  [ 0  0  0  7 13  3  2  0  0  4  5 10  6  3 ]G  [ 2 12  1  0  0  0  0  0 11  3  1  1  0  3 ]T  [11  0  0  0  0 10 11 12  0  0  1  1  4  7 ]>Nr3c1A  [3 4 3 7 6 1 8 2 3 5 1 0 1 2 0 1 4 3 ]C  [2 0 0 0 1 6 0 0 2 3 3 0 0 4 8 2 0 1 ]G  [4 5 6 1 1 2 1 2 0 0 0 8 0 0 1 1 4 2 ]T  [0 0 0 1 1 0 0 5 4 1 5 1 8 3 0 5 1 3 ]>NR4A2A  [ 8 13  0  3  2  0 14  3 ]C  [ 1  0  0  0  2 13  0  8 ]G  [ 3  1 13 11  0  0  0  2 ]T  [ 1  0  1  0 10  1  0  0 ]>Pax4A  [ 7 20 16 11 13 11  6  9  5  5  6  7  8  6 10  4  3  7  4  9  5  5  7  6  3  7  3  1  6  3 ]C  [ 2  0  2  1  1  3  1  1  3 11 11  7  7  5  5  8  6  8  9  6  7  6 11  9 12  9 12 11 11 13 ]G  [11  1  1  1  3  1  2  1  6  1  4  5  2  1  4  4  4  3  3  2  5  2  0  1  2  2  2  3  1  2 ]T  [ 1  0  2  8  4  6 12 10  7  4  0  2  4  9  2  5  8  3  5  4  4  8  3  5  4  3  4  6  3  3 ]>Pax5A  [ 4  4  4  1  2  7  2  0  1  6  6  0  2  3  1  6  5  2  2  5 ]C  [ 1  0  3  2  7  2  5  3  2  1  0  0  8  0  0  1  1 10  5  0 ]G  [ 4  8  3  5  1  1  3  2  8  3  2 12  1  9  4  5  5  0  5  6 ]T  [ 3  0  2  4  2  2  2  7  1  2  4  0  1  0  7  0  1  0  0  1 ]>Pax6A  [ 2  2  4 39  3  1  1 21  1  2 36 11  1  1 ]C  [ 4  2 26  2 34  0 37  2  4 14  0 11  5  0 ]G  [ 4  0  1  1  1 41  4  2  1 25  6 13  3 17 ]T  [33 39 12  1  5  1  1 18 37  2  1  8 34 25 ]>PBX1A  [ 5  3 16  1  0 17 17  0  0 16 12  8 ]C  [ 6  9  1  1 18  1  0  0 18  1  0  2 ]G  [ 2  3  1  0  0  0  0  1  0  0  1  2 ]T  [ 5  3  0 16  0  0  1 17  0  1  5  6 ]>Pbx3A  [377 183   0   3 862 849   0 108 505 ]C  [ 72  50 793 859   0   0   1 450  30 ]G  [309 509   0   0   0  13   0 278 327 ]T  [104 120  69   0   0   0 861  26   0 ]>Pdx1A  [ 1  0 31 31  0  1 ]C  [19  0  0  0  0  1 ]G  [ 5  0  0  0  1 10 ]T  [ 6 31  0  0 30 19 ]>PLAG1A  [ 0  3  0  0  0  0  4 12 11  2  0  0  0  2 ]C  [ 0  0  0  0 14 15 10  0  5  0  0  0  0  0 ]G  [18 14 18 17  4  1  1  0  2 14 18 18 16 16 ]T  [ 0  1  0  1  0  2  3  6  0  2  0  0  2  0 ]>Pou2f2A  [316 111 701   0   0   0 685 621 701  63 165 274 ]C  [ 87 144   0   0   0 671   0   0   0  38 159 149 ]G  [129  34   0   0 682   0   0  21   0  60 199  96 ]T  [169 412   0 701  19  30  16  59   0 540 178 182 ]>Pou5f1A  [282 494  69 130 138 161  90 925  25 446 662  29 868 898 481 288 254 284 271 ]C  [281 118  91 246  27  91 852   8   9  93  14 566  43  20  21  53 171 251 236 ]G  [170 126  52  48  13 679   0   0   0 278   8 227   0  17  28 527 461 250 213 ]T  [206 202 730 518 764  11   0   9 908 125 258 120  31   7 412  74  56 157 220 ]>PPARGA  [ 3  3 19  0  1  0  2 26  5  5  4  1  2 22  1  0  3 22  5  7 ]C  [ 8  0  0  1  0  1 23  1 15  7  2  0  5  5 27 25 12  5 12  0 ]G  [14  0  9 27 26  4  3  0  4 10 18  2 20  0  0  0  0  0  6  1 ]T  [ 3 25  0  0  1 23  0  1  4  6  4 25  1  1  0  3 13  1  5 20 ]>PPARG::RXRAA  [ 94 101 390 100 139 145  71 819 522 713  82  41  22  54 676 ]C  [317 166  23   3  15 129 547  21  48   5   2   9  99 555  58 ]G  [320 127 368 671 674 395 179  15 270 137 767 693 263 144  47 ]T  [126 464  79  87  34 193  66   8  23   9  12 120 479 109  81 ]>Prdm1A [  92 112 406  21   9   0   0   0 312  10   6  31  72  50]C [ 112 230  39 451   4  34   0 472  80 456  53  46  65 215]G [  86  51  42  13   0   0   3   0   9   0   0  54  45  56]T [ 200  96   1   4 477 456 487  18  89  24 431 357 309 169]>Prrx2A  [52 59  0  0 58 ]C  [ 2  0  0  0  0 ]G  [ 4  0  1  0  1 ]T  [ 1  0 58 59  0 ]>CTCF::RAD21A  [234 278 110   0 825  24 173 857   3 493  27  26  34 140 479 ]C  [174 106 757 999   8 618 369   8   0   4  13   0  30 695  50 ]G  [360 425  58   0  63 336  50  78 995 494 470 973 862  45 427 ]T  [231 190  74   0 103  21 407  56   1   8 489   0  73 119  41 ]>RELAA  [ 0  0  0 11 10  2  0  0  0  0 ]C  [ 4  0  0  0  3  0  0  2 18 18 ]G  [11 17 18  7  4  0  0  0  0  0 ]T  [ 3  1  0  0  1 16 18 16  0  0 ]>RELA  [ 0  0  1  5  6  5  1  2  0  1 ]C  [ 5  1  0  1  5  1  0  0 15 16 ]G  [ 8 15 15  9  3  1  0  0  0  0 ]T  [ 4  1  1  2  3 10 16 15  2  0 ]>RESTA  [ 211   58   76 1452   34  122 1575    3   35  913  219   39   20 1406   13 1574   42  205  366  213  179 ]C  [ 174  269 1366   30   44  978    7 1586 1481  201  375    7    5  112 1280    9   13 1007   31  933 1114 ]G  [ 366  146   50   94 1516  323   12   12   20  162  124 1551 1577   34  233    7 1530  183  688  319   37 ]T  [ 840 1124  105   26   10  182   12    5   70  329  886    7    2   51   74   10    9  198  507  125  260 ]>RORAA  [15  9  6 11 21  0  0  0  0 25 ]C  [ 1  1 12  2  0  0  0  0 25  0 ]G  [ 2  0  4  5  4 25 25  0  0  0 ]T  [ 7 15  3  7  0  0  0 25  0  0 ]>RORAA  [ 9 17 15 35 23  2  0 28  0  0  0  0 36 15 ]C  [ 8  2  0  1  0 12  0  0  0  0  0 36  0  6 ]G  [ 8  7  3  0  0 13  0  8 36 36  0  0  0 10 ]T  [11 10 18  0 13  9 36  0  0  0 36  0  0  5 ]>RREB1A  [ 3  1  3  0  7  9  8  4  0 11  4  1  3  4  2  4  4  4  1  4 ]C  [ 8 10  8 11  4  2  3  6 11  0  7 10  8  6  9  5  5  6  7  4 ]G  [ 0  0  0  0  0  0  0  0  0  0  0  0  0  1  0  0  1  0  3  2 ]T  [ 0  0  0  0  0  0  0  1  0  0  0  0  0  0  0  2  1  1  0  1 ]>RUNX1A  [ 287  234  123   57    0   87    0   17   10  131  500 ]C  [ 496  485 1072    0   75  127    0   42  400  463  158 ]G  [ 696  467  149    7 1872   70 1987 1848  251   81  289 ]T  [ 521  814  656 1936   53 1716   13   93 1339 1325 1053 ]>RxraA  [243 112  52  58 600 220   0   0  24   9  99 277 174  89 168 223 ]C  [286 446   4 139 166 669 934 216 363  42 192 397 533 646 329 304 ]G  [202 227  34 766 152  41   0   0  67 188 595 170  59  61  86 212 ]T  [267 213 908  35  80  68  64 782 544 759 112 154 232 202 415 254 ]>RXRA::VDRA  [ 3  0  0  0  0  9  4  2  2  5  0  0  1  0  7 ]C  [ 0  0  0  0  9  0  2  4  0  0  0  0  0  9  1 ]G  [ 7 10  9  0  0  1  0  2  8  5 10  0  0  0  2 ]T  [ 0  0  1 10  1  0  4  2  0  0  0 10  9  1  0 ]>RXR::RARA  [12  0  1  0  0 22  4  5  5 13  5 17  1  0  2  1 21 ]C  [ 0  0  0  0 18  0  7  8  4  1  6  3  1  1  1 17  0 ]G  [11 23 13  1  3  1  5  9 11  7 12  3 20 16  3  3  1 ]T  [ 0  0  9 22  2  0  7  1  3  2  0  0  1  6 17  2  1 ]>ZNF143-like::Six5A  [890   1   0 826   0 805 447  62  43   4   0   8 715  78 311 573  92  62  36 ]C  [  5 895  20  32 893  22  59 287  35 878 896 868   9  76 357  86  69 228 795 ]G  [  1   0   0  26   0  61  53  84  11   1   0   4 158 642 191 203 338 532  33 ]T  [  0   0 876  12   3   8 337 463 807  13   0  16  14 100  37  34 397  74  32 ]>CTCF::SMC3A  [247  76   9 829  21 153 886   5 443  15  12  28 116 477  91 ]C  [100 819 985  14 630 505  22   0   0  17   0  17 791  21 500 ]G  [475  51   0  95 333  73  55 988 551 494 984 873  19 475 353 ]T  [173  50   2  58  12 265  33   3   2 470   0  78  70  23  52 ]>Sox17A  [ 7  8  3 30  0  0  0  0  0 ]C  [ 9  8 18  0  1  0  0  0 17 ]G  [ 6  4  1  0  0  0 31  2 10 ]T  [ 9 11  9  1 30 31  0 29  4 ]>Sox2A  [ 27  20 329   2  14  36  85  63 407  26  26  31 426 367 485 ]C  [446 454   6  10   0  83   4 195 125  37  77 423  42  67  60 ]G  [ 89  22   3   6   6 531   8  76  38  26 445  84  37 156  59 ]T  [107 173 331 651 649  19 571 334  96 577 117 127 159  74  58 ]>Sox5A  [ 8 21 22  0 23 22  0 ]C  [ 4  0  0 22  0  1  0 ]G  [ 3  1  1  0  0  0  0 ]T  [ 8  1  0  1  0  0 23 ]>SOX9A  [24 54 59  0 65 71  4 24  9 ]C  [ 7  6  4 72  4  2  0  6  9 ]G  [31  7  0  2  0  1  1 38 55 ]T  [14  9 13  2  7  2 71  8  3 ]>SP1A  [ 53  39   0   6   5  12  69   0   0   0   9   5  87  78 ]C  [118  99 309 337 376 342   0 384 374 243 344 267 133 141 ]G  [137 176  22   0   0   0 222   1   0   0   2   3  65 101 ]T  [ 80  74  57  45   7  34  97   3  14 145  33 113 103  67 ]>Sp2A  [299 177   0   0 602 602   1 108 320 ]C  [ 30  35 602 602   0   0   0 241  10 ]G  [260 331   0   0   0   0   0 237 272 ]T  [ 13  59   0   0   0   0 601  16   0 ]>SPI1A  [ 145   59    2  910    6   12    0    0    0    8    7  223  104  107  106  169 ]C  [ 292  244  920   10  703    0    0  998  894  259  746   90  102  134  304  248 ]G  [ 149  128   64   22  246    0    0    0    0   75   83    6   34   38  107  171 ]T  [ 414  569   14   58   45  988 1000    2  106  658  164  681  760  721  483  410 ]>SPIBA  [31  4 24  2  1 48 47 ]C  [ 0 14 16  0  0  0  0 ]G  [ 0 29  7 47 47  0  0 ]T  [18  2  2  0  1  1  2 ]>Spz1A  [ 9  0  2  0  1  8  6  1 10  0  2 ]C  [ 0  2  0  0  0  0  0  9  0  1  8 ]G  [ 3  9 10 11  1  0  2  2  2  9  2 ]T  [ 0  1  0  1 10  4  4  0  0  2  0 ]>SREBF1A [  56   0   0 191   0  18   0   0 191   0  26]C [  63   0 181   0 142 107 116 131   0 114  74]G [  69   0   9   0  48  64   0  59   0  23  41]T [   1 191   0   0   0   0  74   0   0  54  48]>SRFA  [ 61 147   1   0 170  46 515  11 375 114  42  13 147 185 402 ]C  [ 88 111 724 717  55   9   7  19  11   9   0   9 167 371  56 ]G  [172 264   2   0   7   5  36   0   1  24 686 700 221  75  92 ]T  [408 207   2  12 497 669 171 699 342 582   1   7 194  98 179 ]>SRYA  [ 5  8 15 18 25  0 28 27  7 ]C  [ 5  3  0  3  0 26  0  0  0 ]G  [10  4  3  3  0  0  0  1  2 ]T  [ 8 13 10  4  3  2  0  0 19 ]>STAT1A  [382 129   0   0  43   4 124 308   5 827 891 484 242 ]C  [255 207   0   0 836 813 437   8   8  51   0 139 271 ]G  [142  55   0   0   0   1  10 559 774  13   6 201 130 ]T  [119 507 898 898  19  80 327  23 111   7   1  74 255 ]>Stat3A  [420 148   0   0  97   0 208 451 100 659 877 227 266 ]C  [213 285   0   0 893 893 452   0  46 293  17 204 247 ]G  [124  97   0  37   0   0   0 498 716   0  37 327 189 ]T  [242 469 999 962   9 106 339  50 137  47  68 241 297 ]>Tal1::Gata1A  [ 400  261  131  265  669  701  664  713  734  996  527 1959    3 2928   16 2770 2382  547 ]C  [1329  444  182  746  635  687  745  699  662  407 1172   31    7    7   26    6   51  550 ]G  [ 618  520 1921 1228  775  903  938  891  862  940  901   37 2936    2   30   23  376 1601 ]T  [ 595 1717  710  706  866  654  599  644  691  603  345  920    0   10 2873  144  134  242 ]>TAL1::TCF3A  [13  9 39 20  0 44  0 12  0  0  0  4 ]C  [14 10  0 24 43  0  1 32  0  0  3  4 ]G  [ 8 20  3  0  1  0 11  0  0 43 20  2 ]T  [ 9  5  2  0  0  0 32  0 44  1 21 34 ]>TBPA  [ 61  16 352   3 354 268 360 222 155  56  83  82  82  68  77 ]C  [145  46   0  10   0   0   3   2  44 135 147 127 118 107 101 ]G  [152  18   2   2   5   0  20  44 157 150 128 128 128 139 140 ]T  [ 31 309  35 374  30 121   6 121  33  48  31  52  61  75  71 ]>TCF12A  [412 162  17 998  10  59  42   0  44 243 ]C  [152 337 968   0   0 425   4   1 339 262 ]G  [250 481   7   0 989 516  13 991 230 275 ]T  [186  20   8   2   1   0 941   8 387 218 ]>TCF7L2-HeLaA [  39   3   0   0   0   5 409 152  25  49  35]C [ 209 397  19   3   0  69   0   0 203  67 132]G [ 116  17   0   0   0 369   3   3 213  21  96]T [  87  33 433 449 453   9  41 296   9 314 189]>Tcfcp2l1A  [   8  285 2425   24  717  402  711 1461 2583 1612   15  252 2187   49 ]C  [3763 3289   21   97 1374 1182 1624  920  285  210 3815 3316   30  118 ]G  [ 255   22 1125 3945  105  254  618 1032  781 1269  224   12 1247 3886 ]T  [  40  477  508   13 1890 2248 1135  677  441  996   34  503  612   17 ]>TEAD1A  [ 1  9  0 12  0  0  0  0  5  1  2  0 ]C  [ 6  0 12  0  0  0 12 11  0  7  4  2 ]G  [ 1  3  0  0  0  0  0  0  0  4  3  8 ]T  [ 4  0  0  0 12 12  0  1  7  0  3  2 ]>TFAP2A-AP2AA  [164 266  16   0   0   0  51 800  16   0  16 125 530 ]C  [304 135 304 999 870 494 743  31   0   0 366 625 174 ]G  [147 294 651   0   0  47 161 112 983 999 608 124  83 ]T  [384 304  28   0 129 458  44  56   0   0   9 125 212 ]>Thap1A  [ 37  62  32  14  13  15  11   1  61  56  32 ]C  [118  73  64 116  59   0 232 247 187  25  88 ]G  [ 54  38  55  26   7 210   0   0   0  81  43 ]T  [ 40  76  98  93 170  24   6   1   1  87  86 ]>TLX1::NFICA  [ 0  0  0  0 14  2  2  7  4  0  0  0 16 14 ]C  [ 0  0  0 16  1  8  8  1  3  0 16 16  0  0 ]G  [ 0 16 16  0  0  5  4  5  2 16  0  0  0  1 ]T  [16  0  0  0  1  1  2  3  7  0  0  0  0  1 ]>TP53A  [ 5  3  4  5 13  0 17  0  0  0  0  0  1  1  4  1 15  2  1  1 ]C  [ 8  7  0  0  0 17  0  0  0 11 16 16  0  0  0 14  0  0  1  2 ]G  [ 2  6 13 12  4  0  0  0 17  0  0  0 15 14 13  2  0  0 14  1 ]T  [ 2  1  0  0  0  0  0 17  0  6  1  1  1  2  0  0  2 15  1 13 ]>USF1A  [ 227   73    0  976    0   53    0    0  903    0   73  223 ]C  [ 241  433 1000    8  719   20    0    0   39  864  533  323 ]G  [ 462  172    0    8    8  927    0 1000   51   12  114  261 ]T  [  70  322    0    8  273    0 1000    0    7  124  280  192 ]>USF2A  [ 247   74    0  979    0   26    0    0  905    0  110  167 ]C  [ 231  421 1000    8  795    9    0    0   48  844  491  348 ]G  [ 467  136    0    8    6  965    0 1000   36   16  123  272 ]T  [  55  369    0    5  199    0 1000    0   11  140  276  213 ]>YY1A  [ 52  94  24   8   0 867   0  31  25   0  79 144 ]C  [626 602   0 859 867   0   0 479   9  26 132 216 ]G  [109 133 822   0   0   0   0 126  21   0 503 354 ]T  [ 80  38  21   0   0   0 867 231 812 841 153 153 ]>Zbtb33A  [194  72   3  78  10   0 123   0   6 594   3 574  92   4 ]C  [176 229 106 670  56 765   0 746   0  46  42  55 174 424 ]G  [300 211  26  11   8   0 644   4 761 100 593  90 133  47 ]T  [ 97 255 632   8 693   2   0  17   0  27 129  48 368 292 ]>Zbtb7aA  [167 123  34  91  26 149 109  41  19  25  10  21 115   0   8   0  53 167 161 ]C  [368 403 681 571 763 533 211 515 782 686 859 774   1 805 810 704 619 327 358 ]G  [306 299  74  87 105  82 432 261   0  32   8   0 304  87   0  43  81 326 320 ]T  [136 161 204 244  99 229 241 176 192 250 116 198 573 101 175 246 240 166 140 ]>ZEB1A  [114 204   0 919   0   0   0 193 421 163 124 ]C  [301 314 906   0   0   0   0   0 170 102 259 ]G  [323 288  13   0 919 919   0 726 127 586 377 ]T  [170 106   0   0   0   0 919   0 201  68 159 ]>Zfp423A  [ 7  0 16 17  0  0  1 24 13  0  0  0  0  0 11 ]C  [ 4 16 17 16 33 33 17  0  0  0  0  0  0  8 22 ]G  [22 17  0  0  0  0  0  0 16 33 33 17 16 16  0 ]T  [ 0  0  0  0  0  0 15  9  4  0  0 16 17  9  0 ]>ZfxA  [ 50  60  91  72  10   6  30 191   9   0   1   0   0  84 ]C  [177 170 151  49 297 362 125 154   2   3 480 480   2 122 ]G  [179 172 199 298 144   2 182 121 469 477   0   0   0 219 ]T  [ 70  75  37  60  30 111 144  15   1   1   0   1 479  56 ]>ZNF143-LIKEA  [199 258 311 435 874   0   2 792   1 779 478  57  41   6   0   0 712  77 325 ]C  [251 168  26  26   8 881  38  41 876  20  69 337  39 862 882 877   1  73 336 ]G  [319 310 527 412   0   0   1  25   0  74  64 103  15   0   0   0 162 636 190 ]T  [113 146  18   9   0   1 841  24   5   9 271 385 787  14   0   5   7  96  31 ]>Znf263A  [ 130  412  204   49    4 1000    7    9  939   50  147 ]C  [ 283  117    0    0   44    0    0  326    0  481  289 ]G  [ 476  311  762  925  952    0  991  665   61  453  292 ]T  [ 111  160   34   26    0    0    2    0    0   16  271 ]>Znf274A  [ 98  94  40 418  45  28 354   2   0 565 201   0   0   1   0   0 181  48 ]C  [ 65 350 482  20  28  39   2   0   0   4  10   0  86 592   0 536  51 163 ]G  [113  88  40  41 510   4 222  34 593  16 382   3   0   0   1   0  17 212 ]T  [317  61  31 114  10 522  15 557   0   8   0 590 507   0 592  57 343 168 ]>ZNF354CA  [ 7  3  0  0 16  0 ]C  [ 6  2 16 16  0 15 ]G  [ 3  0  0  0  0  1 ]T  [ 0 11  0  0  0  0 ]
